# National situation, trends, and predictions of disease burden of atopic dermatitis in Chinese children and adolescents

**DOI:** 10.3389/fmicb.2023.1161969

**Published:** 2023-06-15

**Authors:** Yang Guo, Kao-Yuan Zhang, Yan-Fen Zou, Bo Yu

**Affiliations:** Department of Dermatology, Institute of Dermatology, Peking University Shenzhen Hospital, Shenzhen Peking University-The Hong Kong University of Science and Technology Medical Center, Shenzhen, China

**Keywords:** atopic dermatitis, children, China, disease burden, dermatology

## Abstract

**Background:**

Atopic dermatitis (AD) is an important global health problem affecting children and adolescents and detailed national information of disease burden in China is lacking. We aimed to evaluate the national disease burden of AD in Chinese children and adolescent, to provide the temporal trends over the past 30 years and to predict the burden for the next 10 years.

**Methods:**

The data of AD in China, including incidence, prevalence, and DALY, and population data were obtained from the Global Burden of Disease Study 2019 (GBD study 2019), which were estimated using the DisMod-MR 2.1. We analyzed the three measures by age and sex; the age groups were <5 years, 5–9 years, 10–14 years, and 15–19 years. The joinpoint regression analyses was conducted to assess the temporal trends from 1990 to 2019. The Bayesian age-period cohort (BAPC) model was used to predict measures from 2020 to 2030.

**Results:**

In 2019, the highest incidence case and rate were observed in <5 years group; for prevalence and disability adjusted life year (DALY), the groups of <5 years and 5–9 years showed similar higher levels and the groups of 10–14 years and 15–19 years had similar relatively lower levels. Overall, the male-to-female ratios were >1 in <5 years group and <1 in 10–14 and 15–19 age groups. The trend analyses found an overall trend of decrease in cases of the three measures; in recent about 3 years, slight increase trends were shown in cases and rates of the three measures in <5 years group. The prediction analyses found a slight decreasing trend for cases of these measures and a slight increasing trend for rates of these measures in the <5 years group in the next 10 years; the 5–9 years group was predicted to increase slightly in rates of the three measures.

**Conclusion:**

In conclusion, the groups of <5 years and 5–9 years are two important populations that need targeted measures to reduce disease burden of AD in China. Regarding sex disparity, we should pay more attention to males in <5 years group and to females in 10–19 years group.

## Introduction

Atopic dermatitis (AD) is a chronic inflammatory skin condition characterized by recurrent eczematous lesions and intense itch (Weidinger et al., 2018; Langan et al., 2020). The pathogenesis of AD is multifactorial, including genetic susceptibility, epidermal barrier dysfunction, immunological dysregulation, skin microbiota dysbiosis, etc. (Malik et al., 2017). Globally, in terms of disease burden, AD is a major type of dermatitis, which ranks first in the disease burden caused by skin diseases (Xue et al., 2022). It has been estimated that at least 230 million people worldwide are suffering from AD (Weidinger et al., 2018). For the relationship between age and disease manifestation of AD, compared with adults, children have higher incidence and prevalence than AD (Laughter et al., 2021). Thus, children are the key population for disease prevention and treatment of AD. However, the detailed national information on children in China is lacking. The most available studies focusing on Chinese children were mainly regional surveys (Li et al., 2004; Xu et al., 2012; Zhang et al., 2017) and comprehensive reports for China at the national level are needed.

The Global Burden of Disease Study 2019 (GBD study 2019) (GBD 2019 Diseases and Injuries Collaborators, 2020) provided comprehensive epidemiology data of the disease burden of 369 diseases and injuries for different age groups, sexes, and 204 countries and territories from 1990 to 2019. The GBD datasets have been considered a powerful tool that has been widely used in disease burden research (Armocida et al., 2022; Liu et al., 2022). Therefore, we conducted the current analyses based on data from GBD study 2019. In the present study, we aimed to evaluate the national disease burden of AD in Chinese children and adolescents, including incidence, prevalence, and disability adjusted life year (DALY). We also provided the temporal trends over the past 30 years and the predictions for the next 10 years for the three measures.

## Methods

### Data sources

For the data of disease burden, we used data from GBD study 2019 (Diseases and Injuries Collaborators, 2020). The detailed descriptions of the methodology used by GBD study 2019 have been described in the previous publication (Diseases and Injuries Collaborators, 2020; GBD 2019 Viewpoint Collaborators, 2020). Briefly, the data from GBD study 2019 were from national censuses, disease surveillance point systems, systematically reviewed published literature, etc. Further, the data from these sources were input into DisMod-MR 2.1 to estimate measures, by age, sex, year, and geography. The DisMod-MR is a Bayesian meta-regression tool and could ensure consistency between epidemiological parameters. In addition, the measures were reported with the 95% uncertainty intervals (UIs), including the value of a parameter with 95% probability, which were based on sequential percentages of 2.5 and 97.5 of 1,000 draws of the uncertainty distribution assessed in simulation testing (Diseases and Injuries Collaborators, 2020).

In the prediction analyses, the population data from 1990 to 2019 and the forecast data of next 10 years were needed. For our study, the population data from 1990 to 2019 were obtained from the GBD repository webpage (Institute for Health Metrics and Evaluation (IHME). (n.d.-a)). The population forecast data from 2020 to 2030 were obtained from another GBD repository webpage (Institute for Health Metrics and Evaluation (IHME). (n.d.-b)).

### Measures of disease burden

We extracted data of incidence cases, incidence rates, prevalence cases, prevalence rates, DALY cases, and DALY rates of AD cases under 19 years in China from 1990 to 2019, through the GBD Results tool (IHME. (n.d.)). Age- and sex-specific cases and rates were described in four GBD age groups, including <5 years, 5–9 years, 10–14 years, and 15–19 years. In detail, the incidence case refers to the number of new cases in the population; the incidence rate refers to the new cases per 100,000 population. The prevalence case refers to the total number of cases in the population; the prevalence rate refers to the total cases per 100,000 population. In addition, one DALY represents the loss of the equivalent of 1 year of full health; the DALY case refers to the number of DALYs in the population; the DALY rate refers to the DALYs per 100,000 population.

### Joinpoint regression analysis

To assess the magnitude and direction of trends in the incidence, prevalence, and DALY, we conducted joinpoint regression analysis (Kim et al., 2000) to calculate the average annual percentage change (AAPC) and annual percentage change (APC) and the corresponding 95% CIs, and to compare the value and 0 to determine whether the fluctuation trend in different parts was statistically significant. A two-sided *p*-value of less than 0.05 were deemed statistically significant. For the significance tests, Monte Carlo methods were used, and the overall asymptotic significance level was obtained through a Bonferroni correction. We used the Joinpoint software (version 4.9.1.0; National Cancer Institute, Rockville, MD, United States) to conduct joinpoint regression analysis and construct figures for joinpoint regression.

### Bayesian age-period cohort model

To predict the measures from 2020 to 2030, the Bayesian age-period cohort (BAPC) model (Riebler and Held, 2017) was used. The BAPC model is based on an integrated nested Laplacian approximation to approximate marginal posterior distributions, avoiding some of the mixing and convergence problems introduced by the Markov Chain Monte Carlo sampling technique traditionally used for Bayesian methods (Huang et al., 2022). We used the BAPC and INLA packages in R program (version 4.2.2) for BAPC analyses.

## Results

### The disease burden in 2019

#### Incidence cases and rates

**Figure 1:**
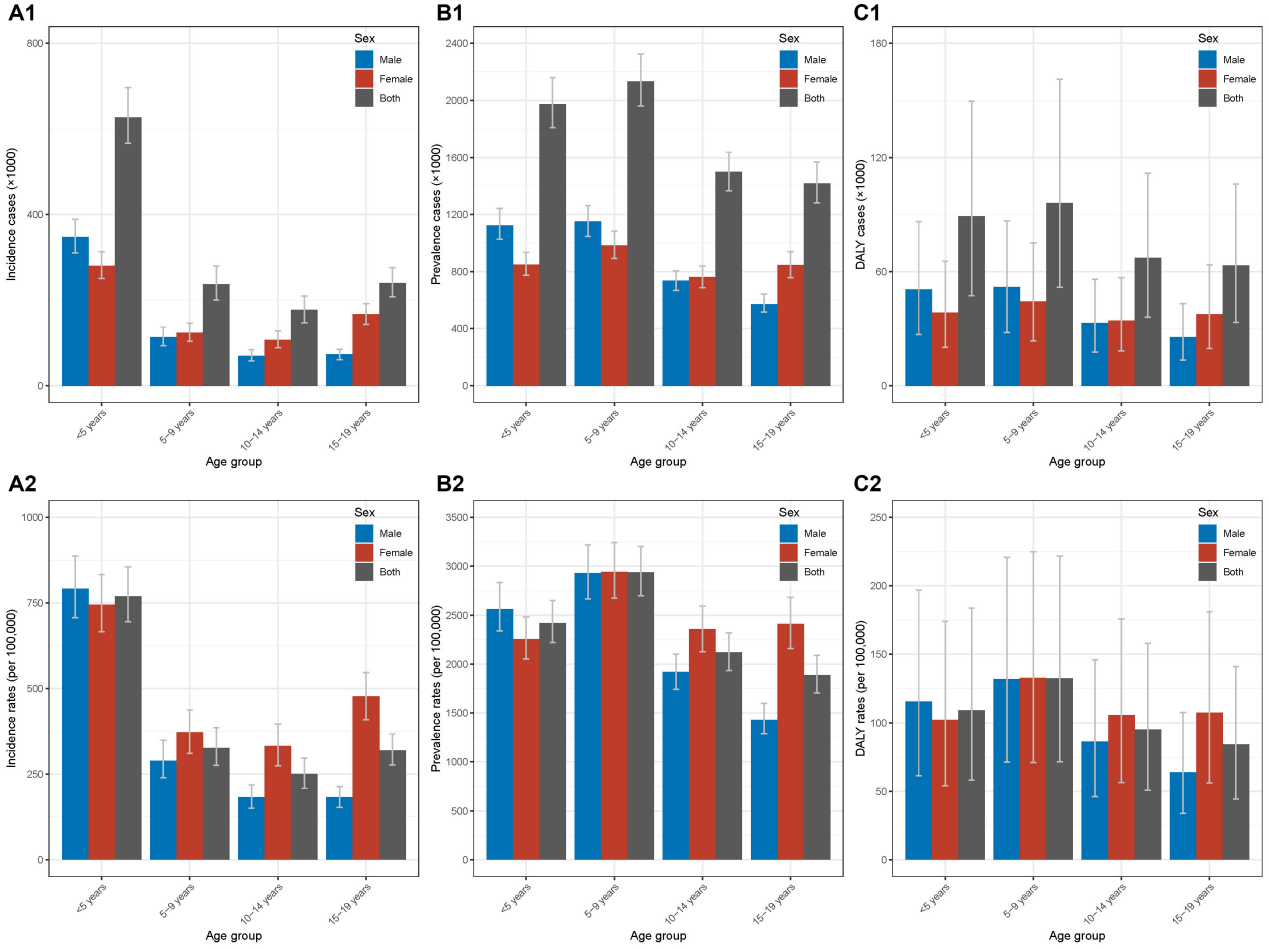
Incidence, prevalence, and DALY of Chinese children and adolescents with AD in different age groups in 2019 **(A1)** incidence cases in different age groups in 2019; **(A2)** incidence rates in different age groups in 2019; **(B1)** prevalence cases different age groups in 2019; **(B2)** prevalence rates in different age groups in 2019; **(C1)** DALY cases in different age groups in 2019; **(C2)** DALY rates in different age groups in 2019. AD, atopic dermatitis; DALY, disability-adjusted life year.

In 2019, the incidence cases of Chinese AD were 627.34 (566.2–696.56) thousand, 237.48 (199.93–279.82) thousand, 177.11 (147.22–209.69) thousand, and 240.3 (207.7–275.97) thousand in groups of <5 years, 5–9 years, 10–14 years, and 15–19 years, respectively (Figure 1A1; Table 1). For the incidence rate, the four age groups showed the incidence rates of 769.83 (694.81–854.78) per 100,000, 326.97 (275.26–385.25) per 100,000, 250.67 (208.37–296.79) per 100,000, 319.82 (276.43–367.29) per 100,000, respectively (Figure 1A2; Table 1). The highest incidence case and rate were seen in <5 years group; the male-to-female ratios in different age groups were 1.0–1.5 (<5 years), 0.5–1.0 (5–9 years), 0.5–1.0 (10–14 years), and 0–0.5 (15–19 years) for cases and rates (Supplementary Figures S1A1–A4,B1–B4).

**Table 1:** Incidence, prevalence, and DALY of Chinese children and adolescents with AD in 1990 and 2019 and the average annual percent changes.

	Cases (× 1,000) (95% UI)			Rate (per 100,000) (95% UI)		
	1990	2019	AAPC (%)	1990	2019	AAPC (%)
**Incidence**						
**Male**						
<5 years	469.58 (419.92–527.91)	347.09 (310.11–389.18)	−1.04^*^ (−1.14 to −0.94)	770.67 (689.18–866.41)	791.13 (706.85–887.07)	0.09^*^ (0.00–0.18)
5–9 years	158.69 (132.93–188.23)	113.26 (93.78–136.98)	−1.16^*^ (−1.26 to −1.06)	291.33 (244.05–345.56)	288.44 (238.83–348.86)	−0.04^*^ (−0.06 to −0.02)
10–14 years	95.72 (78.37–115.37)	69.86 (57.58–83.86)	−1.11^*^ (−1.33 to −0.88)	180.43 (147.72–217.47)	182.31 (150.25–218.83)	0.03^*^ (0.02–0.05)
15–19 years	117.13 (99.05–136.68)	73.18 (61.11–85.44)	−1.62^*^ (−1.79 to −1.45)	179.76 (152.01–209.76)	182.5 (152.41–213.09)	0.06^*^ (0.03–0.08)
**Female**						
<5 years	394.31 (353.45–437.81)	280.25 (250.32–313.23)	−1.18^*^ (−1.31 to −1.05)	724.02 (648.99–803.9)	744.99 (665.43–832.64)	0.10 (−0.01 to 0.21)
5–9 years	189.85 (159.16–220.17)	124.22 (103.48–145.68)	−1.47^*^ (−1.59 to −1.36)	377.64 (316.6–437.96)	372.3 (310.13–436.61)	−0.06^*^ (−0.09 to −0.02)
10–14 years	162.89 (132.85–194.95)	107.25 (88.78–128.14)	−1.44^*^ (−1.67 to −1.20)	327.59 (267.17–392.06)	331.7 (274.57–396.3)	0.04^*^ (0.03–0.05)
15–19 years	285.13 (243.9–328.54)	167.13 (143.22–191.43)	−1.84^*^ (−2.00 to −1.68)	461.96 (395.15–532.29)	476.95 (408.73–546.32)	0.11^*^ (0.10–0.13)
**Both**						
<5 years	863.89 (776.36–963.09)	627.34 (566.2–696.56)	−1.10^*^ (−1.23 to −0.96)	748.65 (672.8–834.62)	769.83 (694.81–854.78)	0.11^*^ (0.01–0.20)
5–9 years	348.53 (294.98–406.03)	237.48 (199.93–279.82)	−1.34^*^ (−1.44 to −1.24)	332.76 (281.63–387.64)	326.97 (275.26–385.25)	−0.07^*^ (−0.09 to −0.05)
10–14 years	258.61 (213.62–306.72)	177.11 (147.22–209.69)	−1.30^*^ (−1.53 to −1.06)	251.63 (207.85–298.44)	250.67 (208.37–296.79)	−0.02^*^ (−0.03 to −0.01)
15–19 years	402.26 (346.87–464.69)	240.3 (207.7–275.97)	−1.77^*^ (−1.93 to −1.61)	317.04 (273.38–366.24)	319.82 (276.43–367.29)	0.03^*^ (0.01–0.06)
**Prevalence**						
**Male**						
<5 years	1518.13 (1385.5–1665.18)	1123.48 (1026.47–1242.48)	−1.04^*^ (−1.16 to −0.92)	2491.54 (2273.88–2732.89)	2560.79 (2339.66–2832.03)	0.10^*^ (0.01–0.19)
5–9 years	1594.92 (1456.15–1750.64)	1150.4 (1046.25–1262.98)	−1.15^*^ (−1.24 to −1.06)	2928.07 (2673.3–3213.94)	2929.76 (2664.5–3216.47)	0.00 (−0.02 to 0.01)
10–14 years	1018.14 (913.96–1114.44)	736.21 (667.32–805.85)	−1.15^*^ (−1.50 to −0.79)	1919.16 (1722.78–2100.69)	1921.15 (1741.39–2102.89)	0.00 (−0.04 to 0.03)
15–19 years	924.56 (828.57–1032.76)	572.24 (516.82–641.66)	−1.67^*^ (−1.94 to −1.41)	1418.9 (1271.59–1584.96)	1427.19 (1288.96–1600.32)	0.02^*^ (0.00–0.03)
**Female**						
<5 years	1189.61 (1079.02–1309.72)	848.62 (772.85–932.98)	−1.16^*^ (−1.34 to −0.97)	2184.3 (1981.25–2404.84)	2255.86 (2054.45–2480.13)	0.13^*^ (0.04–0.21)
5–9 years	1477.43 (1345.01–1623.57)	982.19 (892.26–1081.69)	−1.42^*^ (−1.52 to −1.32)	2938.89 (2675.48–3229.57)	2943.62 (2674.09–3241.82)	0.00 (−0.02 to 0.01)
10–14 years	1165.81 (1059.72–1286.02)	762.16 (687.56–837.8)	−1.48^*^ (−1.80 to −1.16)	2344.58 (2131.23–2586.35)	2357.21 (2126.49–2591.17)	0.01 (−0.01 to 0.04)
15–19 years	1450.72 (1300.21–1622.79)	844.9 (756.68–940.11)	−1.88^*^ (−2.11 to −1.66)	2350.4 (2106.55–2629.18)	2411.22 (2159.45–2682.92)	0.09^*^ (0.07–0.10)
**Both**						
<5 years	2707.73 (2488.21–2951.71)	1972.1 (1807.76–2159.4)	−1.09^*^ (−1.23 to −0.95)	2346.53 (2156.29–2557.97)	2420.03 (2218.36–2649.87)	0.11^*^ (0.02–0.21)
5–9 years	3072.36 (2817.66–3342.57)	2132.6 (1959.99–2324.86)	−1.28^*^ (−1.37 to −1.19)	2933.26 (2690.09–3191.24)	2936.13 (2698.49–3200.83)	0.00 (−0.02 to 0.01)
10–14 years	2183.95 (1987.29–2386.67)	1498.36 (1366.34–1636.64)	−1.32^*^ (−1.64 to −0.99)	2124.98 (1933.63–2322.23)	2120.7 (1933.84–2316.41)	−0.01 (−0.04 to 0.01)
15–19 years	2375.28 (2146.87–2643.83)	1417.14 (1281.57–1568.99)	−1.80^*^ (−2.04 to −1.56)	1872.03 (1692.02–2083.69)	1886.1 (1705.67–2088.2)	0.02^*^ (0.01–0.04)
**DALY**						
**Male**						
<5 years	68.24 (37.03–115.31)	50.65 (26.89–86.34)	−1.02^*^ (−1.11 to −0.92)	111.99 (60.77–189.24)	115.44 (61.29–196.81)	0.11^*^ (0.02–0.20)
5–9 years	71.66 (37.75–121.9)	51.81 (27.93–86.66)	−1.15^*^ (−1.23 to −1.06)	131.55 (69.3–223.79)	131.94 (71.12–220.69)	0.00 (−0.02 to 0.02)
10–14 years	45.57 (24.09–77.18)	33.01 (17.66–55.96)	−1.17^*^ (−1.53 to −0.80)	85.89 (45.4–145.49)	86.15 (46.07–146.04)	0.00 (−0.04 to 0.05)
15–19 years	41.33 (21.54–68.83)	25.59 (13.56–43.06)	−1.67^*^ (−1.95 to −1.40)	63.43 (33.06–105.64)	63.82 (33.81–107.39)	0.02 (−0.01 to 0.05)
**Female**						
<5 years	53.53 (28.62–90.88)	38.35 (20.26–65.44)	−1.14^*^ (−1.28 to −1.00)	98.3 (52.56–166.86)	101.94 (53.85–173.97)	0.13^*^ (0.04–0.22)
5–9 years	66.33 (35.58–112.33)	44.28 (23.62–75.03)	−1.41^*^ (−1.50 to −1.32)	131.95 (70.77–223.45)	132.72 (70.79–224.86)	0.01 (−0.01 to 0.04)
10–14 years	52.09 (27.78–88.5)	34.15 (18.21–56.88)	−1.47^*^ (−1.80 to −1.14)	104.76 (55.87–177.99)	105.62 (56.33–175.92)	0.02 (0.00–0.05)
15–19 years	64.44 (33.37–108.76)	37.61 (19.59–63.47)	−1.87^*^ (−2.10 to −1.65)	104.4 (54.07–176.21)	107.34 (55.91–181.13)	0.10^*^ (0.07–0.12)
**Both**						
<5 years	121.77 (65.24–205.66)	88.99 (47.35–149.61)	−1.08^*^ (−1.22 to −0.94)	105.53 (56.54–178.23)	109.2 (58.11–183.59)	0.13^*^ (0.04–0.21)
5–9 years	137.99 (73.54–234.42)	96.09 (51.85–161.07)	−1.27^*^ (−1.36 to −1.18)	131.74 (70.21–223.81)	132.3 (71.39–221.76)	0.01 (−0.01 to 0.03)
10–14 years	97.66 (52.01–166.83)	67.17 (35.93–111.65)	−1.31^*^ (−1.68 to −0.94)	95.02 (50.61–162.33)	95.06 (50.85–158.03)	0.00 (−0.03 to 0.02)
15–19 years	105.77 (55.29–178.29)	63.2 (33.28–106.05)	−1.79^*^ (−2.03 to −1.55)	83.36 (43.57–140.52)	84.12 (44.29–141.15)	0.03^*^ (0.01–0.05)

AAPC, average annual percent change; AD, atopic dermatitis; DALY, disability-adjusted life year; UI, uncertainty interval. ^*^p < 0.05.

#### Prevalence cases and rates

The prevalence cases of Chinese AD in 2019 were 1.9721 (1.80776–2.1594) million, 2.1326 (1.95999–2.32486) million, 1.49836 (1.36634–1.63664) million, and 1.41714 (1.28157–1.56899) million in the four age groups (Figure 1B1; Table 1). The prevalence rates in the four age groups were 2420.03 (2218.36–2649.87) per 100,000, 2936.13 (2698.49–3200.83) per 100,000, 2120.7 (1933.84–2316.41) per 100,000, and 1886.1 (1705.67–2088.2) per 100,000 (Figure 1B2; Table 1). The groups of <5 years and 5–9 years showed relatively higher prevalence. For the sex disparity, the male-to-female ratios were 1.0–1.5 (<5 years), 0.5–1.0 (5–9 years), 0.5–1.0 (10–14 years), and 0–0.5 (15–19 years; Supplementary Figures S2A1–A4,B1–B4).

#### DALY cases and rates

For DALY in 2019, the DALY cases in the four age groups were 88.99 (47.35–149.61) thousand, 96.09 (51.85–161.07) thousand, 67.17 (35.93–111.65) thousand, and 63.2 (33.28–106.05) thousand, respectively (Figure 1C1; Table 1). The DALY rates in the four age groups were 109.2 (58.11–183.59) per 100,000, 132.3 (71.39–221.76) per 100,000, 95.06 (50.85–158.03) per 100,000, and 84.12 (44.29–141.15) per 100,000 (Figure 1C2; Table 1). The groups of <5 years and 5–9 years had relatively higher values of DALY. Similar distributions of male-to-female ratios in the four age groups were seen (Supplementary Figures S3A1–A4,B1–B4).

### Trends in the disease burden from 1990 to 2019

#### Incidence cases and rates

**Figure 2:**
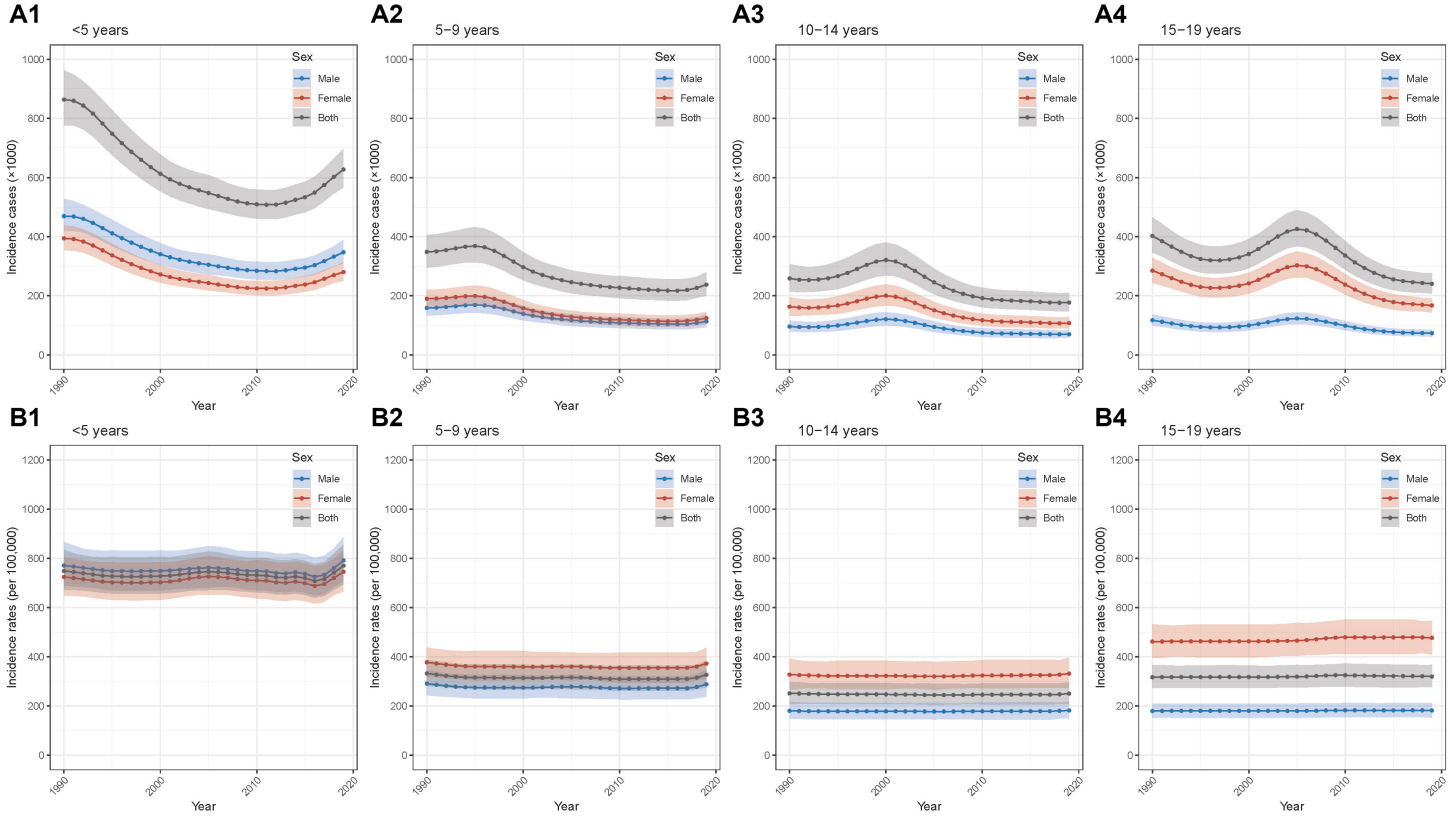
Temporal trend of incidence of Chinese children and adolescents with AD in different age groups from 1990 to 2019 **(A1)** temporal trend of incidence cases for aged <5 years; **(A2)** temporal trend of incidence cases for aged 5–9 years; **(A3)** temporal trend of incidence cases for aged 10–14 years; **(A4)** temporal trend of incidence cases for aged 15–19 years; **(B1)** temporal trend of incidence rates for aged <5 years; **(B2)** temporal trend of incidence rates for aged 5–9 years; **(B3)** temporal trend of incidence rates for aged 10–14 years; **(B4)** temporal trend of incidence rates for aged 15–19 years. AD, atopic dermatitis.

The detailed data of incidence cases and rates from 1990 to 2019 in different age groups were presented in Supplementary Tables S1, S2. As shown in the Table 1, overall decreases trend in incidence cases from 1990 to 2019 were observed with AAPCs of −1.10% (−1.23% to −0.96%), −1.34% (−1.44% to −1.24%), −1.30% (−1.53% to −1.06%), and − 1.77% (−1.93% to −1.61%) in the four groups (all *p*-values <0.05). In the group of <5 years, according to the joinpoint regression analyses, the incidence cases decreased from 1990 to 2012 and then increased from 2012 to 2019, with the same trends of males and females; the largest APC was found in the period of 2016–2019 (4.76, 4.29%–5.24%; Figure 2A1; Supplementary Figure S4A1; Supplementary Table S7). In the groups of 5–9 years and 10–14 years, the incidence cases increased slightly and then decreased slightly with similar trends of males and females (Figures 2A2,A3; Supplementary Figures S4A2,A3; Supplementary Table S7). For the group of 15–19 years, an overall decrease trend with fluctuation was seen; the trend for females was more fluctuated (Figure 2A4; Supplementary Figure S4A4; Supplementary Table S7). In addition, the male-to-female ratios remained relatively stable from 1990 to 2019 (Supplementary Figures S1A1–A4).

For incidence rates, the AAPCs between 1990 and 2019 were 0.11% (0.01%–0.20%), −0.07% (−0.09% to −0.05%), −0.02% (−0.03% to −0.01%), 0.03% (0.01%–0.06%) (all *p*-values <0.05) in the four age groups (Table 1). In the group of <5 years, it was relatively stable and increased slightly in the recent years from 2017 to 2019 (Figure 2B1; Supplementary Figure S4B1; Supplementary Table S8); incidence rates in other three groups remained stable between 1990 and 2019 (Figures 2B2–B4; Supplementary Figures S4B2–B4 and Supplementary Table S8). Furthermore, the patterns of male-to-female ratio stayed relatively stable from 1990 to 2019 (Supplementary Figures S1B1–B4).

#### Prevalence cases and rates

**Figure 3:**
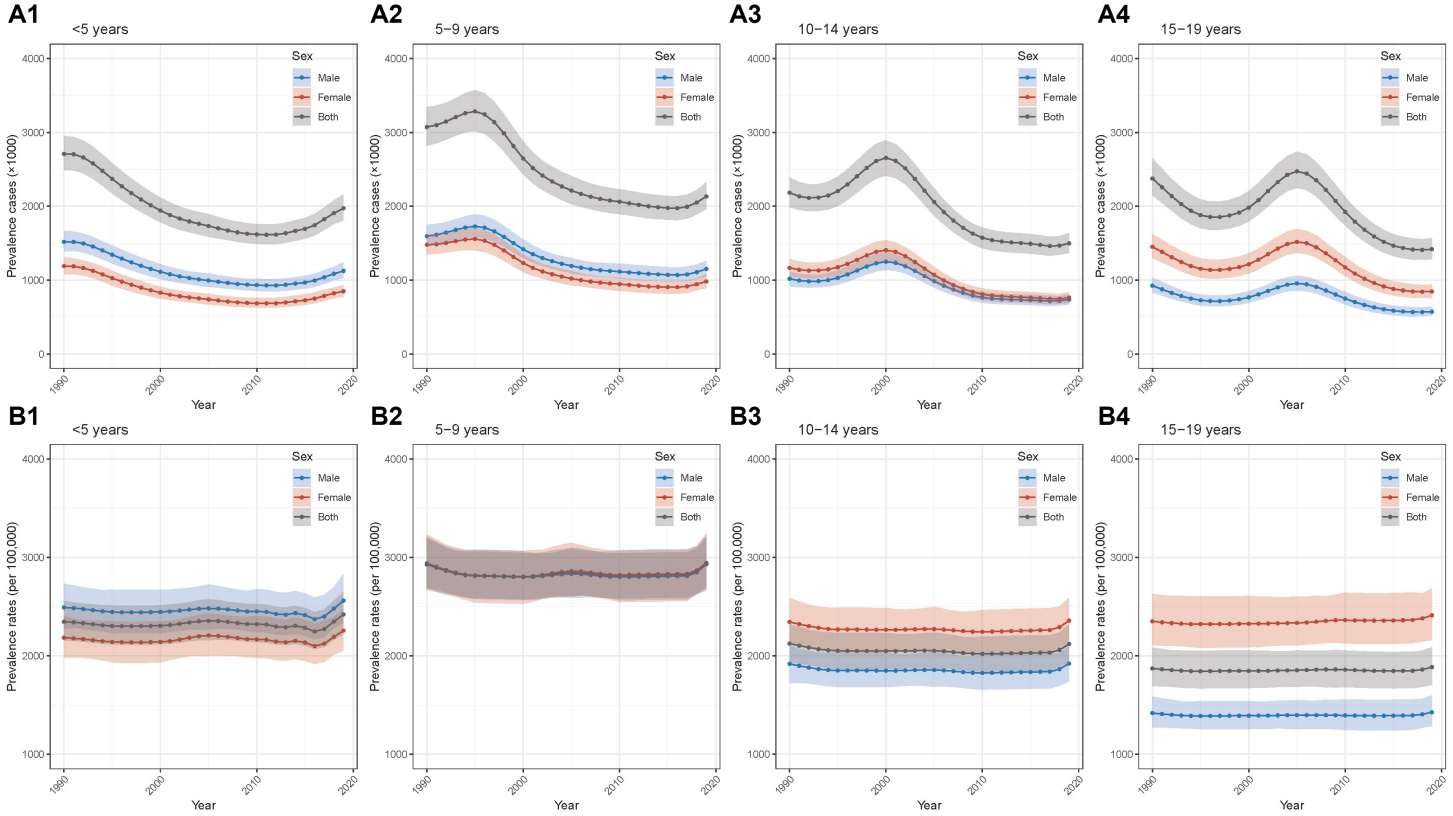
Temporal trend of prevalence of Chinese children and adolescents with AD in different age groups from 1990 to 2019 **(A1)** temporal trend of prevalence cases for aged <5 years; **(A2)** temporal trend of prevalence cases for aged 5–9 years; **(A3)** temporal trend of prevalence cases for aged 10–14 years; **(A4)** temporal trend of prevalence cases for aged 15–19 years; **(B1)** temporal trend of prevalence rates for aged <5 years; **(B2)** temporal trend of prevalence rates for aged 5–9 years; **(B3)** temporal trend of prevalence rates for aged 10–14 years; **(B4)** temporal trend of prevalence rates for aged 15–19 years. AD, atopic dermatitis.

The detailed data of prevalence cases and rates from 1990 to 2019 in different age groups were shown in Supplementary Table S3, S4. For prevalence cases, from 1990 to 2019, the overall decease trends were observed and the AAPCs in the four age groups were −1.09% (−1.23% to −0.95%), −1.28% (−1.37% to −1.19%), −1.32% (−1.64% to −0.99%), and −1.80% (−2.04% to −1.56%), respectively (all *p*-values <0.05; Table 1). Furthermore, the joinpoint regression analyses found that in the group of <5 years, the prevalence cases decreased from 1990 to 2012 and then increased from 2012 to 2019; the largest APC was seen in the period of 2015–2019 (3.99, 3.67%–4.30%; Figure 3A1; Supplementary Figure S5A1; Supplementary Table S9). The groups of 5–9 years and 10–14 years showed similar trends of increase slightly and then decrease slightly (Figures 3A2,A3; Supplementary Figures S5A2,A3; Supplementary Table S9). The group of 15–19 years had an overall decrease trend with fluctuations (Figure 3A4; Supplementary Figure S5A4; Supplementary Table S9). Additionally, for sex disparity, the patterns of male-to-female ratio remained relatively stable over the past 30 years (Supplementary Figures S2A1–A4).

Regarding prevalence rates, the AAPCs were 0.11% (0.02%–0.21%, *p* < 0.05), 0.00% (−0.02% to 0.01%), −0.01% (−0.04% to 0.01%), 0.02% (0.01%–0.04%, *p* < 0.05), respectively (Table 1). The four age groups displayed similar overall trends of general stability and slight increases over the last 2–4 years (Figures 3B1–B4; Supplementary Figures S5B1–B4; Supplementary Table S10). In terms of sex difference, relatively stable trends of male-to-female ratio patterns were seen between 1990 and 2019 (Supplementary Figures S2B1–B4).

#### DALY cases and rates

**Figure 4:**
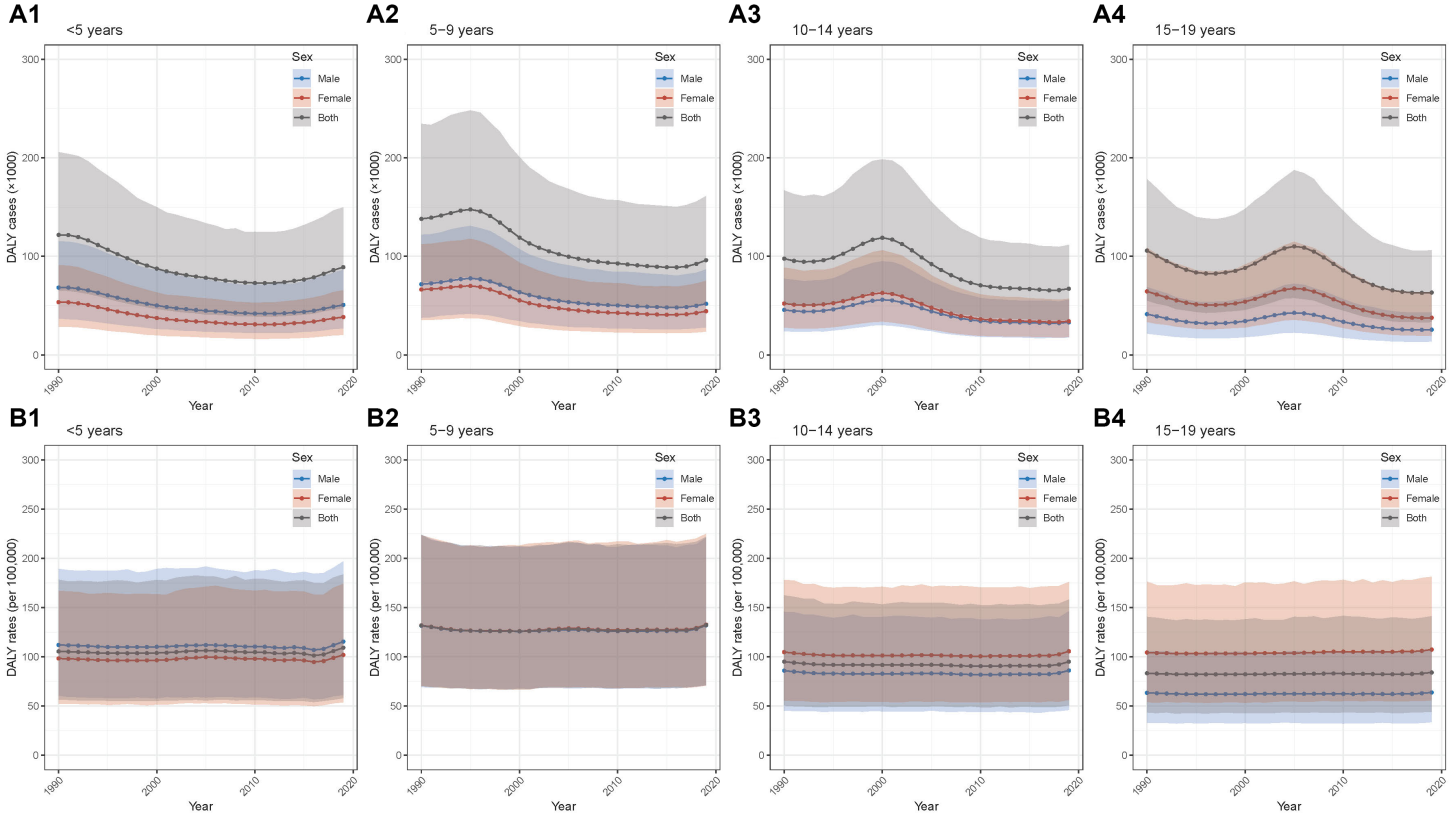
Temporal trend of DALY of Chinese children and adolescents with AD in different age groups from 1990 to 2019 **(A1)** temporal trend of DALY cases for aged <5 years; **(A2)** temporal trend of DALY cases for aged 5–9 years; **(A3)** temporal trend of DALY cases for aged 10–14 years; **(A4)** temporal trend of DALY cases for aged 15–19 years; **(B1)** temporal trend of DALY rates for aged <5 years; **(B2)** temporal trend of DALY rates for aged 5–9 years; **(B3)** temporal trend of DALY rates for aged 10–14 years; **(B4)** temporal trend of DALY rates for aged 15–19 years. AD, atopic dermatitis; DALY, disability-adjusted life year.

The detailed data of DALY cases and rates from 1990 to 2019 in different age groups were shown in Supplementary Tables S5, S6. Between 1990 and 2019, the DALY cases showed overall decrease trends with AAPCs of −1.08% (−1.22% to −0.94%), −1.27% (−1.36% to −1.18%), −1.31% (−1.68% to −0.94%), −1.79% (−2.03% to −1.55%) in the four age groups (all *p*-values for AAPC were < 0.05) (Table 1). In the group of <5 years, the DALY cases decreased from 1990 to 2012 and then increased from 2012 to 2019, with the largest APC in the period of 2015–2019 (3.99, 3.68%–4.31%; Figure 4A1; Supplementary Figure S6A1; Supplementary Table S11). The other three groups displayed overall decrease trends with fluctuations (Figures 4A2–A4; Supplementary Figures S6A2–A4; Supplementary Table S11). Moreover, for sex disparity, the patterns of male-to-female ratio stayed relatively stable during the past 30 years (Supplementary Figures S3A1–A4).

The AAPCs for DALY rates in the four age groups were 0.13% (0.04%–0.21%, *p* < 0.05), 0.01% (−0.01% to 0.03%), 0.00% (−0.03% to 0.02%), 0.03% (0.01%–0.05%, *p* < 0.05; Table 1). The similar overall trends of general stability and slight increases over the recent 2–3 years were observed in the four age groups (Figures 4B1–B4; Supplementary Figures S6B1–B4 and Supplementary Table S12). In addition, the patterns of male-to-female ratio remained relatively stable from 1990 to 2019 (Supplementary Figures S3B1–B4).

### Predictions in yhe disease burden from 2020 to 2030

#### Incidence cases and rates

**Figure 5:**
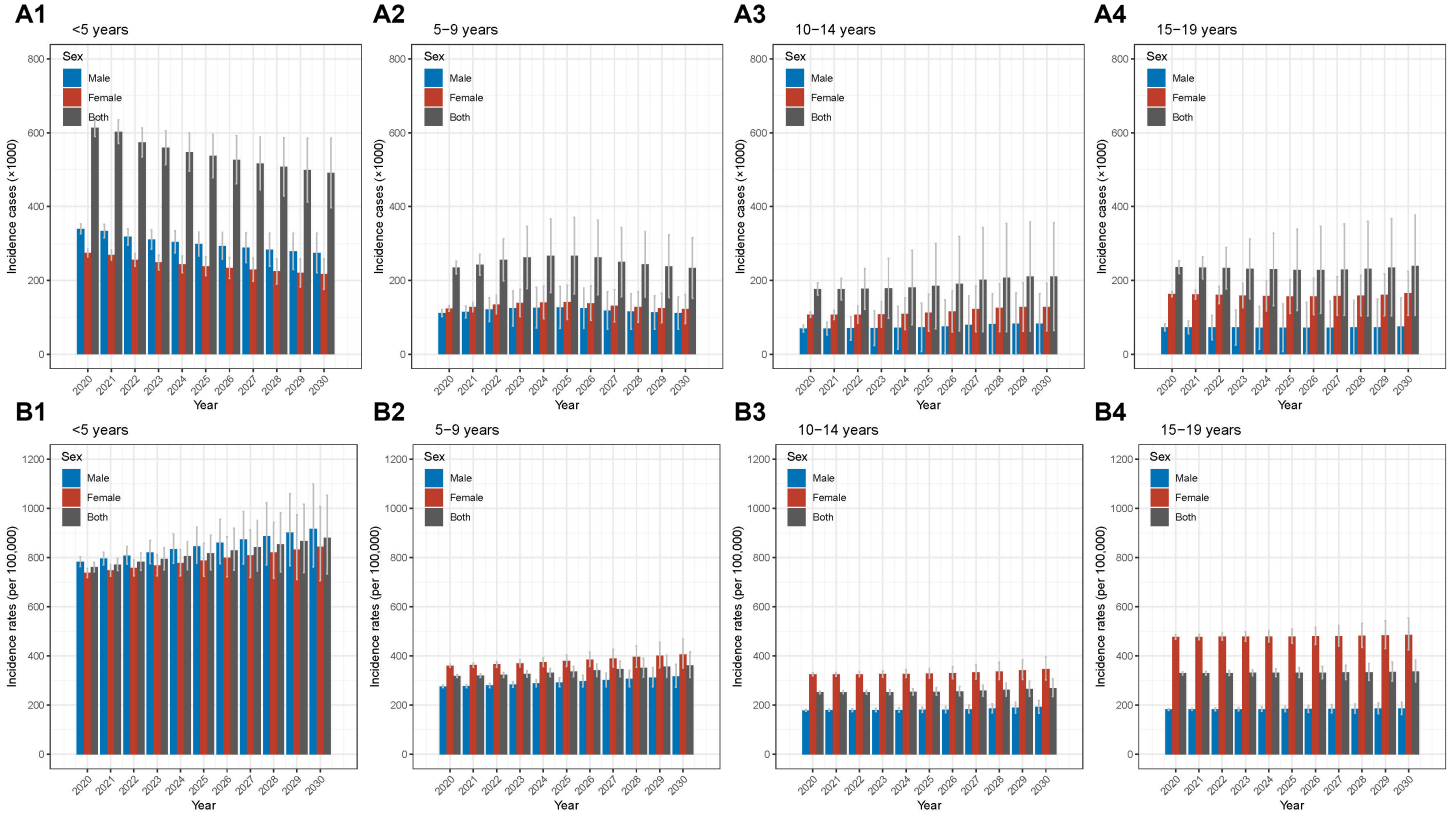
Predicted incidence of Chinese children and adolescents with AD in different age groups from 2020 to 2030 **(A1)** predicted incidence cases for aged <5 years; **(A2)** predicted incidence cases for aged 5–9 years; **(A3)** predicted incidence cases for aged 10–14 years; **(A4)** predicted incidence cases for aged 15–19 years; **(B1)** predicted incidence rates for aged <5 years; **(B2)** predicted incidence rates for aged 5–9 years; **(B3)** predicted incidence rates for aged 10–14 years; **(B4)** predicted incidence rates for aged 15–19 years. AD, atopic dermatitis.

The results of BAPC model for incidence cases and rates prediction were presented in the Supplementary Tables S13, S14. We found that in the population of <5 years, the incidence case will show a decreasing trend for both sexes in the upcoming 10 years and it was predicted to be about 491.0 thousand by 2030 (Figure 5A1; Supplementary Table S13). The other three groups were predicted to stay relatively stable in males and females from 2020 to 2030 (Figures 5A2–A4).

By contrast, the incidence rates were forecast to increase slightly in the next 10 years in <5 years and 5–9 years age groups (Figures 5B1,B2). The 10–14 and 15–19 age groups were predicted to show relatively stable trends in males and females for the period from 2020 to 2030 (Figures 5B3,B4).

#### Prevalence cases and rates

**Figure 6:**
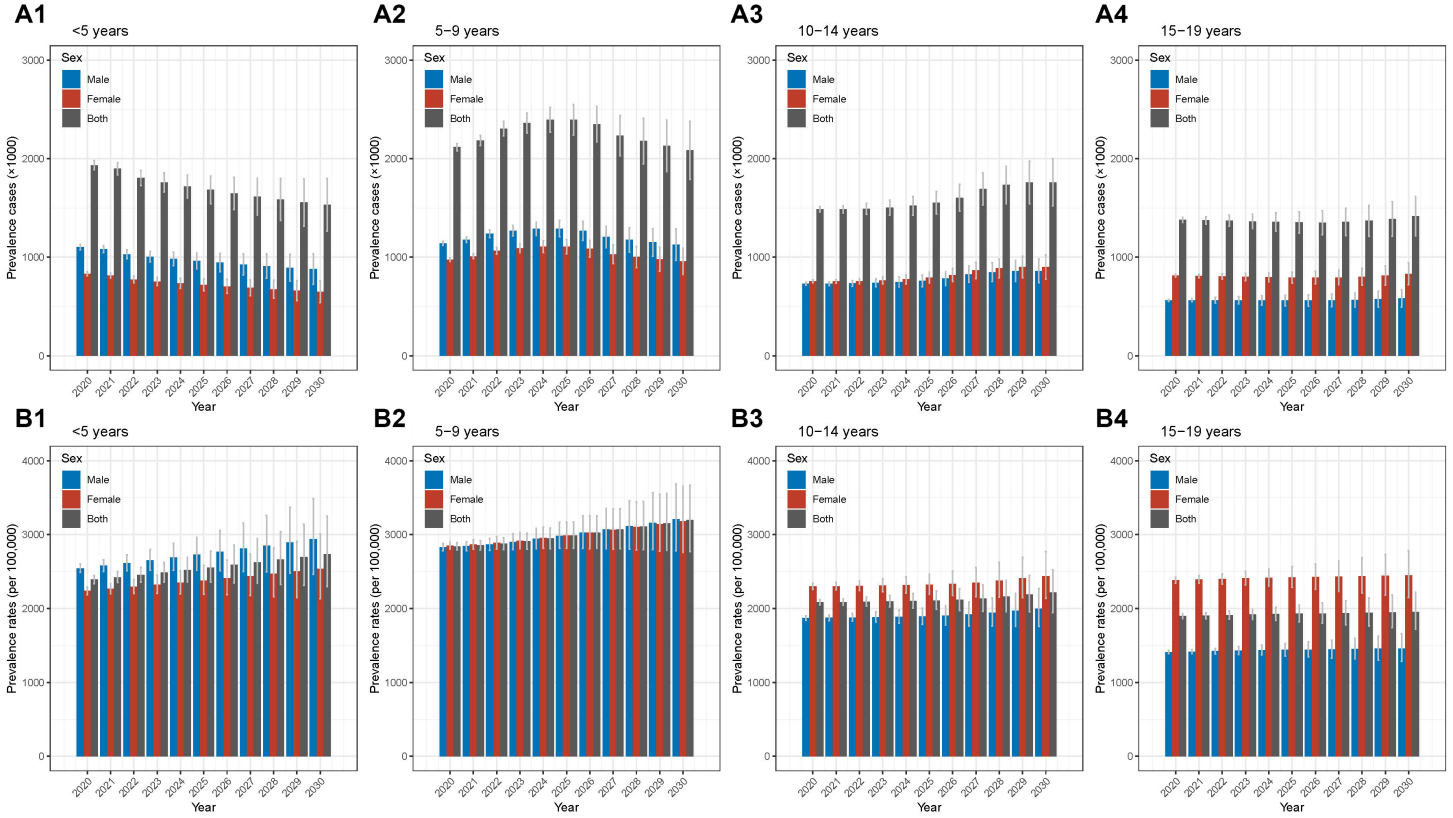
Predicted prevalence of Chinese children and adolescents with AD in different age groups from 2020 to 2030 **(A1)** predicted prevalence cases for aged <5 years; **(A2)** predicted prevalence cases for aged 5–9 years; **(A3)** predicted prevalence cases for aged 10–14 years; **(A4)** predicted prevalence cases for aged 15–19 years; **(B1)** predicted prevalence rates for aged <5 years; **(B2)** predicted prevalence rates for aged 5–9 years; **(B3)** predicted prevalence rates for aged 10–14 years; **(B4)** predicted prevalence rates for aged 15–19 years. AD, atopic dermatitis.

As the Figure 6A1 demonstrated, BAPC prediction analyses found a decreasing trend of prevalence cases for both sexes in the <5 years age group; by 2030, the prevalence cases were predicted to decrease to 1.53 million (Supplementary Table S15). In the 5–9 years age group, it was projected to increase slightly from 2020 to 2025 and then decline slightly from 2020 to 2030 (Figure 6A2). In terms of the group of 10–14 years, from 2020 to 2030, it was projected to stay stable in the first 5 years and then increase slightly in the last 5 years (Figure 6A3). Regarding the 15–19 years group, the prevalence cases was predicted to remain relatively stable in the upcoming 10 years (Figure 6A4).

For the prevalence rates, the age groups of <5 years and 5–9 years were forecast to increase slightly for both males and females in the next 10 years (Figures 6B1,B2). In the 10–14 and 15–19 age groups, the prevalence rates were projected to remain relatively stable for both sexes (Figure 6B3,B4). The detailed results of BAPC model for prediction of prevalence rates were presented in the Supplementary Table S16.

#### DALY cases and rates

**Figure 7:**
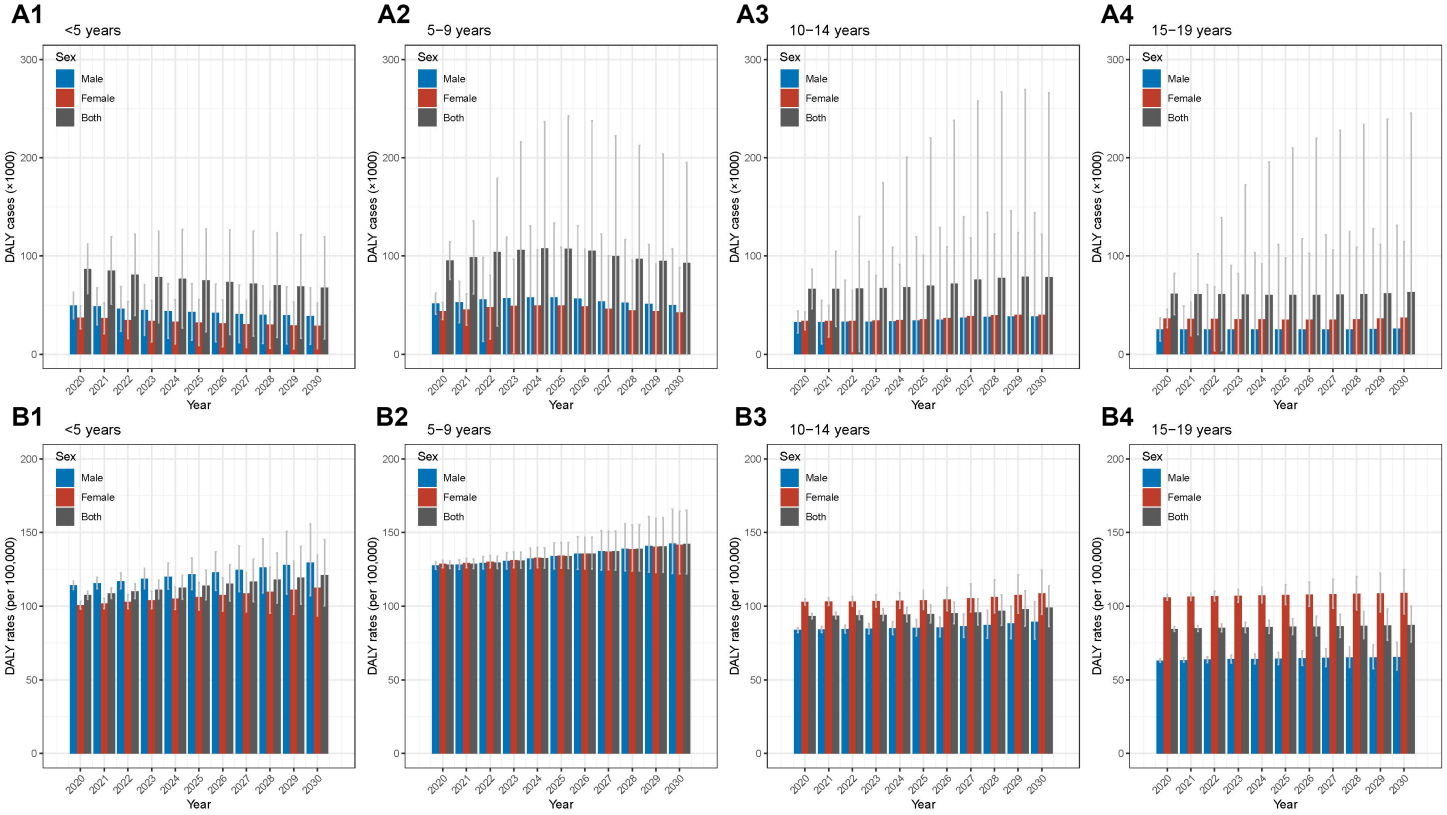
Predicted DALY of Chinese children and adolescents with AD in different age groups from 2020 to 2030 **(A1)** predicted DALY cases for aged <5 years; **(A2)** predicted DALY cases for aged 5–9 years; **(A3)** predicted DALY cases for aged 10–14 years; **(A4)** predicted DALY cases for aged 15–19 years; **(B1)** predicted DALY rates for aged <5 years; **(B2)** predicted DALY rates for aged 5–9 years; **(B3)** predicted DALY rates for aged 10–14 years; **(B4)** predicted DALY rates for aged 15–19 years. AD, atopic dermatitis; DALY, disability-adjusted life year.

As shown in the Figure 7A1, the DALY cases were projected to decrease from 2020 to 2030 for both sexes in the <5 years group; by 2030, it was predicted to be 67.6 thousand (Supplementary Table S17). In the age group of 5–9 years, it was projected to increase slightly between 2020 and 2025 and then decrease between 2026 and 2030 (Figure 7A2). For the 10–14 years group, the DALY cases will show a slight increasing trend for both sexes in the next 10 years (Figure 7A3). In terms of the 15–19 years group, it was predicted to stay relatively stable in males and females (Figure 7A4).

For the DALY rates, the prediction analyses suggested a gradual increase in trends for both males and females in <5 years and 5–9 years age groups (Figures 7B1,B2). The other two groups were forecast to stay relatively stable for both sexes (Figure 7B3,B4). The detailed results of BAPC model for prediction of DALY rates were presented in the Supplementary Table S18.

## Discussion

In our study, we present a comprehensive report of the national disease burden of AD in Chinese children and adolescents, including incidence, prevalence, and DALY. The current situations in 2019, the temporal trends during the past 30 years, and the predictions of the disease burden of AD have been reported, with particular attention paid to age- and sex-specific patterns. Our findings provided insights into strategies to reduce the disease burden of AD in China.

Among Chinese children and adolescents, we observed the peak of incidence cases and rates mainly in the age group <5 years, indicating that this population is considered a high-risk group of onsets of AD in China. Similar pattern was also observed in other regions. A cohort study conducted in Denmark reported that the highest incidence of AD during the first 18 months of life (Nissen et al., 2013). For the disease onset, approximately up to 80% of children with AD demonstrate the onset of AD during infancy in UK (Williams and Strachan, 1998; Bylund et al., 2020). Compared with the group of <5 years, the group of 5–9 years showed similar and even higher values of prevalence and DALY although the incidence cases and rates were lower than those aged <5 years. This finding suggested that, in addition to the group of <5 years, attentions should also be paid for the population of 5–9 years. Another study conducted in Shanghai, a city in east-central China, found that the prevalence rates in different age groups (3 ~ years, 4 ~ years, 5 ~ years, and 6 ~ years) were similar (Xu et al., 2012). In line with our findings, a Sweden study (Ballardini et al., 2012) reported similar prevalence rates in <5 years and 5–9 years group. Therefore, the groups of <5 years and 5–9 years are two important high-risk populations that need targeted measures to reduce incidence and prevalence in China.

The sex disparity with a marked shift in different age groups for the burden of AD in China was identified. Specifically, the male-to-female ratios were >1 in <5 years group and <1 in 10–14 and 15–19 age groups. Similar patterns in sex difference were reported in European population (Böhme et al., 2001; Dirven-Meijer et al., 2008); however, the results remained controversial across studies (Lee et al., 2007; Peroni et al., 2008). The modulation effects of sex hormones on immune responses might partly explain the phenomenon (Kanda et al., 2019). Moreover, females aged 10–19 years may have an increased risk of skin barrier damage (Lin, 2009), which might result from higher usage of cosmetics and skin care products than males; however, further investigations focusing on Chinese population are needed to confirm such hypothesis. Based on our findings in China, we should pay more attention to males in the <5 years group and to females in 10–19 years group. Therefore, age- and sex-specific measures are needed to reduce disease burden of AD in China.

AD is an important part of atopic march (Paller et al., 2019), which is generally characterized by the progression of AD to other atopic conditions, including allergic rhinitis and asthma. AD is considered the first step of atopic march (Bantz et al., 2014) and children with AD have increased risk of developing allergic rhinitis and asthma (von Kobyletzki et al., 2012). With the progression of atopic march, the prevalence rates of allergic rhinitis increase with age and the prevalence rates of AD and wheeze decrease with age (Belgrave et al., 2014). In our present study focusing on China, we provided the manifestation of atopic march in Chinese children and adolescents in the aspect of AD disease burden with detailed prevalence data reported in different age groups.

In terms of temporal trends, we did joinpoint regression analyses and found that over the past 30 years for this age group, we found an overall trend of decrease in cases of incidence, prevalence, and DALY; however, in recent years, slight increase trends were shown in cases and rates of these measures. For the groups of <5 years, the increases of cases in incidence, prevalence, and DALY started around the year of 2013 (APCs’ range: 1.60%–1.97%) followed by a larger increase of rates in incidence, prevalence, and DALY from 2016 (APCs’ range: 3.43%–3.99%). In China, the one-child policy was replaced by a selective two-child policy in 2013 and then replaced by a universal two-child policy officially in 2016 (Zeng and Hesketh, 2016). The population of <5 years increased and the changes of temporal trends in 2013 and 2016 may be resulted from the new policies in China.

The previous studies on national disease burden of AD in China were mainly surveys focusing on prevalence rates. Three national epidemiologic surveys focusing on prevalence rates have been conducted in 1998 (Gu et al., 2000), 2002 (Gu et al., 2004), and 2014 (Guo et al., 2016); the prevalence rates of AD reported in the above surveys were 0.69% (1998) (Gu et al., 2000), 3.07% (2002) (Gu et al., 2004), and 12.94% (2014) (Guo et al., 2016). The study carried out in 2002 reported similar results as our present study. Based on the above studies, an increase trend of prevalence rates in China was seen. Of note, however, the participants in the 1998 study (Gu et al., 2000) were aged 6–20 years from 11 cities in China and the diagnosis information was obtained from questionnaire with the criteria of Williams et al. (1996) used; for the 2002 study (Gu et al., 2004), the participants were aged 1–7 years from 10 cities in China and questionnaire method was also used based on the criteria of Williams et al. (1996). But in the 2014 study (Guo et al., 2016), besides the method of questionnaire, clinical diagnosis made by experienced dermatologists was considered with the criteria of Williams et al. (1996) used; 12 cities in China were enrolled. The discrepancies in age groups, regions, diagnosis, etc. may explain the difference of the above studies and our study.

### Strengths and limitations

To the best of our knowledge, our study is the most up-to-date and comprehensive report to evaluate the national disease burden of AD in Chinese children and adolescents, which is a common disease affecting children in China. We provided detailed analyses of the current situation in 2019, the temporal trends over the past 30 years, and the predictions for the next 10 years evaluated by age and sex, with three major measures of burden reported, including incidence, prevalence, and DALY. For the implications of our study, we provided insights into strategies to prevent and control AD in Chinese children and adolescents. Based on our findings, age- and sex-specific strategies targeting high-risk populations could be considered to reduce the burden of AD in China.

There are some limitations of our study. First, the GBD data were from national censuses, disease surveillance point systems, systematically reviewed published literature, etc. (Diseases and Injuries Collaborators, 2020). Moreover, the diagnostic criteria for AD may differ in these data sources (Urban et al., 2021). Accordingly, the quality and heterogeneity of the original data could influence the accuracy of estimates in the database of GBD. In addition, we cannot obtain the detailed data on disease burden of AD at the provincial level in China. Thus, the further analyses of the geographic distribution of the disease burden of AD in China cannot be conducted.

## Conclusion

In conclusion, the groups of <5 years and 5–9 years are two important populations that need targeted measures to reduce disease burden of AD in China. Regarding the sex disparity, we should pay more attention to males in the <5 years group and to females in 10–19 years group. Our findings provided insights into age- and sex-specific strategies to reduce disease burden of AD in China.

## Data availability statement

The datasets presented in this study can be found in online repositories. The names of the repository/repositories and accession number(s) can be found at: https://vizhub.healthdata.org/gbd-results/.

## Author contributions

YG and BY structured and designed the study. YG and K-YZ conducted data analyses. YG wrote the first draft of the manuscript. BY and Y-FZ critically reviewed the manuscript. All authors contributed to the article and approved the submitted version.

## Funding

This work was supported by the National Natural Science Foundation of China (no. 82103727), the fellowship of China Postdoctoral Science Foundation (no. 2021M702221), Guangdong Basic and Applied Basic Research Foundation (nos. 2022A1515010957 and 2021A1515011558), Shenzhen Sanming Project (no. SZSM201812059), Shenzhen Key Medical Discipline Construction Fund (no. SZXK040), and Shenzhen Science and Technology Program (no. RCBS20210706092408008).

## Conflict of interest

The authors declare that the research was conducted in the absence of any commercial or financial relationships that could be construed as a potential conflict of interest.

## Publisher’s note

All claims expressed in this article are solely those of the authors and do not necessarily represent those of their affiliated organizations, or those of the publisher, the editors and the reviewers. Any product that may be evaluated in this article, or claim that may be made by its manufacturer, is not guaranteed or endorsed by the publisher.
