# Supplementary material for: National situation, trends, and predictions of disease burden of atopic dermatitis in Chinese children and adolescents
Source: Front Microbiol. 2023 Jun 15;14:1161969. doi: 10.3389/fmicb.2023.1161969 (PMC10308015; doi:10.3389/fmicb.2023.1161969)
Supplement: Supplementary file 1 [file Data_Sheet_1.docx]

Supplementary Material

## Supplementary Table 1. Incidence cases of Chinese children and adolescents with AD in different age groups from 1990 to 2019

| **Age group** | **Year** | **Sex** | **Value** | **Lower** | **Upper** |
| --- | --- | --- | --- | --- | --- |
| <5 years | 1990 | Male | 469578.9 | 419924.2 | 527912.8 |
| <5 years | 1990 | Female | 394313.8 | 353452.6 | 437814.9 |
| <5 years | 1990 | Both | 863892.7 | 776359.1 | 963089.8 |
| <5 years | 1991 | Male | 468121.8 | 419518.7 | 520412.5 |
| <5 years | 1991 | Female | 391738.8 | 352822.1 | 434262.1 |
| <5 years | 1991 | Both | 859860.6 | 776334.7 | 947591.6 |
| <5 years | 1992 | Male | 460085 | 413535.4 | 506826.4 |
| <5 years | 1992 | Female | 383501.2 | 345998.1 | 424170.2 |
| <5 years | 1992 | Both | 843586.2 | 763294.4 | 925219.9 |
| <5 years | 1993 | Male | 446336 | 401303.7 | 492021.3 |
| <5 years | 1993 | Female | 370139.2 | 333360.5 | 410154.7 |
| <5 years | 1993 | Both | 816475.2 | 739012.9 | 895919.3 |
| <5 years | 1994 | Male | 429080.4 | 385129.1 | 474269.1 |
| <5 years | 1994 | Female | 353682.1 | 318398.6 | 392846.6 |
| <5 years | 1994 | Both | 782762.5 | 707706.9 | 862401.1 |
| <5 years | 1995 | Male | 411267.6 | 368009.9 | 456934.7 |
| <5 years | 1995 | Female | 336743.2 | 302467.2 | 375613.3 |
| <5 years | 1995 | Both | 748010.7 | 676079.9 | 828435 |
| <5 years | 1996 | Male | 394952 | 353368.9 | 438734.9 |
| <5 years | 1996 | Female | 321310.1 | 288577.1 | 358469 |
| <5 years | 1996 | Both | 716262.2 | 647387.4 | 793264.4 |
| <5 years | 1997 | Male | 379683.8 | 339674.6 | 421712.2 |
| <5 years | 1997 | Female | 307152.1 | 275838.8 | 342728.2 |
| <5 years | 1997 | Both | 686836 | 620793.8 | 760659.1 |
| <5 years | 1998 | Male | 365561.6 | 327016 | 405976.9 |
| <5 years | 1998 | Female | 294304.9 | 264284.8 | 328430.8 |
| <5 years | 1998 | Both | 659866.4 | 596421.4 | 730769.7 |
| <5 years | 1999 | Male | 352527.3 | 315340.1 | 391465.3 |
| <5 years | 1999 | Female | 282615.1 | 253776.8 | 315407.1 |
| <5 years | 1999 | Both | 635142.3 | 574079.5 | 703362 |
| <5 years | 2000 | Male | 340583.4 | 304648.2 | 378178.2 |
| <5 years | 2000 | Female | 272026.9 | 244263.5 | 303597 |
| <5 years | 2000 | Both | 612610.3 | 553719.6 | 678377 |
| <5 years | 2001 | Male | 330278.7 | 295889.2 | 365124.6 |
| <5 years | 2001 | Female | 263286.2 | 237444.5 | 292626 |
| <5 years | 2001 | Both | 593564.9 | 536473 | 654139.1 |
| <5 years | 2002 | Male | 321954.6 | 288739.6 | 355130.6 |
| <5 years | 2002 | Female | 256752 | 231708.6 | 284979.9 |
| <5 years | 2002 | Both | 578706.7 | 522281.9 | 638072.1 |
| <5 years | 2003 | Male | 315230.1 | 282102.1 | 348569.6 |
| <5 years | 2003 | Female | 251697.1 | 227093.5 | 278687 |
| <5 years | 2003 | Both | 566927.2 | 512276.4 | 625543.5 |
| <5 years | 2004 | Male | 309718.1 | 277048 | 343778.8 |
| <5 years | 2004 | Female | 247399 | 222776.9 | 274378.6 |
| <5 years | 2004 | Both | 557117.2 | 502118.3 | 615717.1 |
| <5 years | 2005 | Male | 304757.1 | 272238 | 339796.5 |
| <5 years | 2005 | Female | 242987.5 | 218329.7 | 270705.4 |
| <5 years | 2005 | Both | 547744.6 | 493262.5 | 608042.3 |
| <5 years | 2006 | Male | 299581.3 | 270000.8 | 333817.3 |
| <5 years | 2006 | Female | 237998.5 | 214377.8 | 264231.8 |
| <5 years | 2006 | Both | 537579.8 | 486047.7 | 595608.9 |
| <5 years | 2007 | Male | 294490.4 | 266209.4 | 327126.9 |
| <5 years | 2007 | Female | 233170.4 | 211105.6 | 257701.3 |
| <5 years | 2007 | Both | 527660.8 | 478322.7 | 580433.4 |
| <5 years | 2008 | Male | 289942.1 | 262028.6 | 320123.7 |
| <5 years | 2008 | Female | 229021.2 | 206665.4 | 253295.1 |
| <5 years | 2008 | Both | 518963.3 | 470489.3 | 570194.6 |
| <5 years | 2009 | Male | 286534.9 | 257736.3 | 317174.2 |
| <5 years | 2009 | Female | 226107.8 | 204166.5 | 250571.9 |
| <5 years | 2009 | Both | 512642.7 | 465479.6 | 562486.1 |
| <5 years | 2010 | Male | 284654.4 | 255122.1 | 315094.4 |
| <5 years | 2010 | Female | 224790.4 | 201320.5 | 250761.3 |
| <5 years | 2010 | Both | 509444.8 | 460741.5 | 559292.4 |
| <5 years | 2011 | Male | 283485.9 | 254125.3 | 313767.1 |
| <5 years | 2011 | Female | 224444.2 | 201012.8 | 250362.5 |
| <5 years | 2011 | Both | 507930 | 459419.5 | 557606.2 |
| <5 years | 2012 | Male | 283224.1 | 253911.6 | 313450.2 |
| <5 years | 2012 | Female | 225106.1 | 201607.6 | 251090.8 |
| <5 years | 2012 | Both | 508330.3 | 459837 | 558029.6 |
| <5 years | 2013 | Male | 286204.6 | 256594.3 | 316736.5 |
| <5 years | 2013 | Female | 228518.5 | 204664 | 254892.5 |
| <5 years | 2013 | Both | 514723.2 | 465681.4 | 565027.8 |
| <5 years | 2014 | Male | 291054.7 | 260942.7 | 322106.3 |
| <5 years | 2014 | Female | 233498.8 | 209122.7 | 260448.7 |
| <5 years | 2014 | Both | 524553.5 | 474583.4 | 575793.2 |
| <5 years | 2015 | Male | 295351.7 | 264792.6 | 326863.8 |
| <5 years | 2015 | Female | 237984.6 | 213138.9 | 265455.1 |
| <5 years | 2015 | Both | 533336.3 | 482510 | 585430.9 |
| <5 years | 2016 | Male | 303500.2 | 272096.7 | 335876.9 |
| <5 years | 2016 | Female | 245518 | 219885.5 | 273861 |
| <5 years | 2016 | Both | 549018.2 | 496676.1 | 602685.9 |
| <5 years | 2017 | Male | 317135.9 | 284319.9 | 350957.7 |
| <5 years | 2017 | Female | 257453.1 | 230575 | 287177.5 |
| <5 years | 2017 | Both | 574589 | 519786 | 630796.7 |
| <5 years | 2018 | Male | 332497.8 | 299655.4 | 366970.7 |
| <5 years | 2018 | Female | 269799 | 243942.6 | 298552.5 |
| <5 years | 2018 | Both | 602296.7 | 546115.3 | 664537.4 |
| <5 years | 2019 | Male | 347088 | 310112.4 | 389180.4 |
| <5 years | 2019 | Female | 280252.2 | 250322.6 | 313225.9 |
| <5 years | 2019 | Both | 627340.1 | 566203.5 | 696564.2 |
| 5-9 years | 1990 | Male | 158685.5 | 132933.9 | 188227.8 |
| 5-9 years | 1990 | Female | 189849 | 159160.9 | 220173.2 |
| 5-9 years | 1990 | Both | 348534.5 | 294980.9 | 406026.8 |
| 5-9 years | 1991 | Male | 159756.3 | 134234.7 | 189770.3 |
| 5-9 years | 1991 | Female | 190532.9 | 162005.6 | 220833.8 |
| 5-9 years | 1991 | Both | 350289.1 | 297996 | 408307.3 |
| 5-9 years | 1992 | Male | 162012.2 | 136667.6 | 190742.9 |
| 5-9 years | 1992 | Female | 192577.2 | 163672.7 | 223487.7 |
| 5-9 years | 1992 | Both | 354589.4 | 303137.4 | 413497.8 |
| 5-9 years | 1993 | Male | 164915.3 | 139558 | 194355.8 |
| 5-9 years | 1993 | Female | 195491.3 | 166804.8 | 227679.2 |
| 5-9 years | 1993 | Both | 360406.6 | 307930.8 | 419829.3 |
| 5-9 years | 1994 | Male | 167630.3 | 141736 | 198909.4 |
| 5-9 years | 1994 | Female | 198211.8 | 168951.2 | 231729.7 |
| 5-9 years | 1994 | Both | 365842.1 | 313143.6 | 426936.6 |
| 5-9 years | 1995 | Male | 169078.8 | 142061.5 | 201070.5 |
| 5-9 years | 1995 | Female | 199253.4 | 167979.6 | 234555.9 |
| 5-9 years | 1995 | Both | 368332.1 | 312952.6 | 432261.6 |
| 5-9 years | 1996 | Male | 167475.1 | 140712 | 199150.1 |
| 5-9 years | 1996 | Female | 196352.1 | 165537.4 | 231145.1 |
| 5-9 years | 1996 | Both | 363827.2 | 309151.4 | 426976.4 |
| 5-9 years | 1997 | Male | 162617 | 136629.9 | 193348 |
| 5-9 years | 1997 | Female | 189412.6 | 159700.1 | 222979.7 |
| 5-9 years | 1997 | Both | 352029.6 | 299167.3 | 413126.7 |
| 5-9 years | 1998 | Male | 155401.3 | 130568.4 | 184735.1 |
| 5-9 years | 1998 | Female | 179568.2 | 151419.4 | 211394 |
| 5-9 years | 1998 | Both | 334969.5 | 284685.2 | 393064.5 |
| 5-9 years | 1999 | Male | 147048.4 | 123557.8 | 174767.7 |
| 5-9 years | 1999 | Female | 168436.7 | 142056.1 | 198292.1 |
| 5-9 years | 1999 | Both | 315485 | 268141.2 | 370055.7 |
| 5-9 years | 2000 | Male | 138913 | 116760.9 | 165063.9 |
| 5-9 years | 2000 | Female | 157756.9 | 133072.3 | 185721.2 |
| 5-9 years | 2000 | Both | 296669.9 | 252187.2 | 347854.7 |
| 5-9 years | 2001 | Male | 132497.5 | 112579.2 | 156285.5 |
| 5-9 years | 2001 | Female | 149240.9 | 126860.9 | 174273.8 |
| 5-9 years | 2001 | Both | 281738.4 | 240508.9 | 328836.1 |
| 5-9 years | 2002 | Male | 127550.1 | 108538.3 | 149738.9 |
| 5-9 years | 2002 | Female | 142445.2 | 121689.2 | 165792.9 |
| 5-9 years | 2002 | Both | 269995.3 | 231420.2 | 314263.4 |
| 5-9 years | 2003 | Male | 123576.5 | 104636.1 | 144750.4 |
| 5-9 years | 2003 | Female | 136891.2 | 117134.1 | 159506.7 |
| 5-9 years | 2003 | Both | 260467.7 | 223014.9 | 303820.4 |
| 5-9 years | 2004 | Male | 120232.5 | 100529.9 | 140927.1 |
| 5-9 years | 2004 | Female | 132302.2 | 113206.5 | 153613.4 |
| 5-9 years | 2004 | Both | 252534.7 | 214719.8 | 294802.8 |
| 5-9 years | 2005 | Male | 117363.2 | 97458.77 | 138113.6 |
| 5-9 years | 2005 | Female | 128625.3 | 108124.3 | 148856.3 |
| 5-9 years | 2005 | Both | 245988.5 | 208474.6 | 285846 |
| 5-9 years | 2006 | Male | 114845.6 | 95359.26 | 134496.6 |
| 5-9 years | 2006 | Female | 125750.1 | 107485.9 | 145437.7 |
| 5-9 years | 2006 | Both | 240595.7 | 204236.6 | 280227.1 |
| 5-9 years | 2007 | Male | 112586.5 | 93787.46 | 131659.9 |
| 5-9 years | 2007 | Female | 123385.5 | 105822.2 | 143096.6 |
| 5-9 years | 2007 | Both | 235972 | 200945.9 | 275220.8 |
| 5-9 years | 2008 | Male | 110707.4 | 92633.66 | 129170 |
| 5-9 years | 2008 | Female | 121514.2 | 103078.1 | 141538.1 |
| 5-9 years | 2008 | Both | 232221.7 | 198566.5 | 271357.1 |
| 5-9 years | 2009 | Male | 109285.3 | 91800.13 | 127669.5 |
| 5-9 years | 2009 | Female | 120075.7 | 100797.3 | 140237.9 |
| 5-9 years | 2009 | Both | 229361 | 194909.8 | 267918.8 |
| 5-9 years | 2010 | Male | 108311.2 | 90261.31 | 127763.8 |
| 5-9 years | 2010 | Female | 118910.1 | 99127.48 | 139887.7 |
| 5-9 years | 2010 | Both | 227221.3 | 191896.6 | 267316.1 |
| 5-9 years | 2011 | Male | 107322.6 | 89436.28 | 126606.6 |
| 5-9 years | 2011 | Female | 117526.5 | 97970.1 | 138268.3 |
| 5-9 years | 2011 | Both | 224849.1 | 189875.6 | 264522 |
| 5-9 years | 2012 | Male | 106253.9 | 88541.91 | 125361.2 |
| 5-9 years | 2012 | Female | 116076.9 | 96763.44 | 136562.6 |
| 5-9 years | 2012 | Both | 222330.8 | 187745.3 | 261557.9 |
| 5-9 years | 2013 | Male | 105313.9 | 87753.54 | 124271.1 |
| 5-9 years | 2013 | Female | 114874.9 | 95766.76 | 135142.2 |
| 5-9 years | 2013 | Both | 220188.8 | 185940.3 | 259039 |
| 5-9 years | 2014 | Male | 104585.2 | 87141.27 | 123430.5 |
| 5-9 years | 2014 | Female | 114055.9 | 95090.67 | 134169.9 |
| 5-9 years | 2014 | Both | 218641.1 | 184639.7 | 257222.6 |
| 5-9 years | 2015 | Male | 103923.8 | 86585.9 | 122666.6 |
| 5-9 years | 2015 | Female | 113484.6 | 94620.41 | 133489.4 |
| 5-9 years | 2015 | Both | 217408.3 | 183604.3 | 255780.2 |
| 5-9 years | 2016 | Male | 103644.8 | 86350.17 | 122350.8 |
| 5-9 years | 2016 | Female | 113487.7 | 94628.09 | 133486.3 |
| 5-9 years | 2016 | Both | 217132.6 | 183375.5 | 255467.2 |
| 5-9 years | 2017 | Male | 104461 | 87027.08 | 123324.3 |
| 5-9 years | 2017 | Female | 114800.9 | 95727.1 | 135025.8 |
| 5-9 years | 2017 | Both | 219261.9 | 185178.9 | 257987.4 |
| 5-9 years | 2018 | Male | 107826.1 | 90072.49 | 127168.6 |
| 5-9 years | 2018 | Female | 118595.5 | 100433.3 | 138002.1 |
| 5-9 years | 2018 | Both | 226421.6 | 193330.2 | 263453.7 |
| 5-9 years | 2019 | Male | 113260.7 | 93780.15 | 136984.1 |
| 5-9 years | 2019 | Female | 124223.9 | 103480.6 | 145681.6 |
| 5-9 years | 2019 | Both | 237484.6 | 199927.4 | 279816.2 |
| 10-14 years | 1990 | Male | 95719.12 | 78365.78 | 115369.3 |
| 10-14 years | 1990 | Female | 162889.4 | 132847 | 194947.5 |
| 10-14 years | 1990 | Both | 258608.5 | 213620.4 | 306718.1 |
| 10-14 years | 1991 | Male | 94189.01 | 77480.67 | 112663.1 |
| 10-14 years | 1991 | Female | 159997.7 | 132619.2 | 188902.6 |
| 10-14 years | 1991 | Both | 254186.7 | 212232.1 | 300727.1 |
| 10-14 years | 1992 | Male | 93885.18 | 77129.19 | 111565 |
| 10-14 years | 1992 | Female | 159291.3 | 133883.7 | 187181.2 |
| 10-14 years | 1992 | Both | 253176.5 | 212867 | 296574.8 |
| 10-14 years | 1993 | Male | 94640.27 | 77633.22 | 112448.6 |
| 10-14 years | 1993 | Female | 160225.4 | 135165.3 | 188776.5 |
| 10-14 years | 1993 | Both | 254865.7 | 214905.5 | 299718.7 |
| 10-14 years | 1994 | Male | 96443.05 | 79123.88 | 114418.8 |
| 10-14 years | 1994 | Female | 162677.1 | 136582 | 192128.8 |
| 10-14 years | 1994 | Both | 259120.1 | 217991.2 | 305786.3 |
| 10-14 years | 1995 | Male | 99439.6 | 81425.96 | 118776.8 |
| 10-14 years | 1995 | Female | 166971 | 138969.8 | 198830.7 |
| 10-14 years | 1995 | Both | 266410.6 | 222978.4 | 315817.3 |
| 10-14 years | 1996 | Male | 103788.3 | 84986.59 | 123966.7 |
| 10-14 years | 1996 | Female | 173492.7 | 144399 | 206585.3 |
| 10-14 years | 1996 | Both | 277280.9 | 232079.6 | 328723.2 |
| 10-14 years | 1997 | Male | 109024.8 | 89274.69 | 130212 |
| 10-14 years | 1997 | Female | 181587.1 | 151142.8 | 216192.7 |
| 10-14 years | 1997 | Both | 290612 | 243253.7 | 344556.2 |
| 10-14 years | 1998 | Male | 114355.6 | 93640.11 | 136569.2 |
| 10-14 years | 1998 | Female | 190065.8 | 158209.9 | 226246.7 |
| 10-14 years | 1998 | Both | 304421.4 | 254822.8 | 360957.3 |
| 10-14 years | 1999 | Male | 118678.6 | 97171.57 | 141728 |
| 10-14 years | 1999 | Female | 197030.7 | 164016.7 | 234500.9 |
| 10-14 years | 1999 | Both | 315709.3 | 264262.7 | 374361.4 |
| 10-14 years | 2000 | Male | 120671.6 | 98810.68 | 144116.3 |
| 10-14 years | 2000 | Female | 200079.8 | 166559.3 | 238112 |
| 10-14 years | 2000 | Both | 320751.4 | 268487.3 | 380344.3 |
| 10-14 years | 2001 | Male | 119013 | 97568.64 | 141034.1 |
| 10-14 years | 2001 | Female | 196595.3 | 165393.5 | 233607.9 |
| 10-14 years | 2001 | Both | 315608.3 | 265888.8 | 373158.8 |
| 10-14 years | 2002 | Male | 114484.9 | 94295 | 136196.7 |
| 10-14 years | 2002 | Female | 187932.4 | 158602.5 | 222691 |
| 10-14 years | 2002 | Both | 302417.2 | 254757.4 | 357012.6 |
| 10-14 years | 2003 | Male | 108156.8 | 89478.67 | 128561 |
| 10-14 years | 2003 | Female | 176065.4 | 149018.2 | 208457.7 |
| 10-14 years | 2003 | Both | 284222.2 | 239257 | 336364.4 |
| 10-14 years | 2004 | Male | 101140.7 | 83609.06 | 120592.5 |
| 10-14 years | 2004 | Female | 163093.4 | 137240.5 | 194543 |
| 10-14 years | 2004 | Both | 264234.1 | 221265.2 | 314203.8 |
| 10-14 years | 2005 | Male | 94437.93 | 77158.98 | 113298.1 |
| 10-14 years | 2005 | Female | 150852.6 | 125202.8 | 178915.3 |
| 10-14 years | 2005 | Both | 245290.5 | 205452.9 | 291337.3 |
| 10-14 years | 2006 | Male | 88834.93 | 73295.08 | 105665.2 |
| 10-14 years | 2006 | Female | 140856.6 | 118200.2 | 167297.8 |
| 10-14 years | 2006 | Both | 229691.6 | 192786 | 272683 |
| 10-14 years | 2007 | Male | 84196.93 | 69592.78 | 99629.46 |
| 10-14 years | 2007 | Female | 132803.9 | 112462.3 | 157757.6 |
| 10-14 years | 2007 | Both | 217000.9 | 182487.5 | 256991.9 |
| 10-14 years | 2008 | Male | 80354.94 | 66021.96 | 95500.85 |
| 10-14 years | 2008 | Female | 126238.3 | 106739.6 | 149722 |
| 10-14 years | 2008 | Both | 206593.2 | 174557.6 | 243509.3 |
| 10-14 years | 2009 | Male | 77227.85 | 63207.77 | 91956.18 |
| 10-14 years | 2009 | Female | 120921.4 | 102191.5 | 143100.7 |
| 10-14 years | 2009 | Both | 198149.2 | 167345.4 | 233639.9 |
| 10-14 years | 2010 | Male | 74851.25 | 60677.31 | 89986.45 |
| 10-14 years | 2010 | Female | 116836.1 | 98233.4 | 139408.1 |
| 10-14 years | 2010 | Both | 191687.3 | 161054.1 | 227258.6 |
| 10-14 years | 2011 | Male | 73355.49 | 59445.3 | 88199.6 |
| 10-14 years | 2011 | Female | 114231.3 | 96019.2 | 136308.2 |
| 10-14 years | 2011 | Both | 187586.8 | 157621.4 | 222356.6 |
| 10-14 years | 2012 | Male | 72459.5 | 58694.12 | 87136.91 |
| 10-14 years | 2012 | Female | 112648.1 | 94658.85 | 134429.2 |
| 10-14 years | 2012 | Both | 185107.6 | 155555.2 | 219424.2 |
| 10-14 years | 2013 | Male | 71920.36 | 58231.81 | 86503.48 |
| 10-14 years | 2013 | Female | 111637.8 | 93781.01 | 133232.9 |
| 10-14 years | 2013 | Both | 183558.1 | 154236.5 | 217625.4 |
| 10-14 years | 2014 | Male | 71538.73 | 57901.02 | 86057.09 |
| 10-14 years | 2014 | Female | 110842.2 | 93109.32 | 132289 |
| 10-14 years | 2014 | Both | 182381 | 153210.8 | 216257.3 |
| 10-14 years | 2015 | Male | 71116.32 | 57544.19 | 85557.54 |
| 10-14 years | 2015 | Female | 109922.7 | 92336.68 | 131192.2 |
| 10-14 years | 2015 | Both | 181039 | 152056 | 214678.4 |
| 10-14 years | 2016 | Male | 70479.17 | 57022.44 | 84794.67 |
| 10-14 years | 2016 | Female | 108599.2 | 91226.49 | 129609.1 |
| 10-14 years | 2016 | Both | 179078.4 | 150393 | 212356.7 |
| 10-14 years | 2017 | Male | 69788.12 | 56462.83 | 83963.72 |
| 10-14 years | 2017 | Female | 107213.9 | 90065.38 | 127949.9 |
| 10-14 years | 2017 | Both | 177002 | 148640.8 | 209886.9 |
| 10-14 years | 2018 | Male | 69543.07 | 57585.08 | 82674.17 |
| 10-14 years | 2018 | Female | 106683.4 | 89493.81 | 125779.6 |
| 10-14 years | 2018 | Both | 176226.5 | 147445.1 | 207925.8 |
| 10-14 years | 2019 | Male | 69861.99 | 57578.17 | 83858.83 |
| 10-14 years | 2019 | Female | 107247.1 | 88775.08 | 128136.1 |
| 10-14 years | 2019 | Both | 177109.1 | 147220.9 | 209694.3 |
| 15-19 years | 1990 | Male | 117131 | 99047.88 | 136680.2 |
| 15-19 years | 1990 | Female | 285131.1 | 243895.4 | 328541.5 |
| 15-19 years | 1990 | Both | 402262 | 346870.3 | 464690.2 |
| 15-19 years | 1991 | Male | 111782 | 95145.59 | 130086.5 |
| 15-19 years | 1991 | Female | 272759.9 | 234129.7 | 312363.6 |
| 15-19 years | 1991 | Both | 384541.9 | 332659.4 | 443535.1 |
| 15-19 years | 1992 | Male | 106321.5 | 89849.68 | 123384.3 |
| 15-19 years | 1992 | Female | 259704.6 | 224951.9 | 295853.2 |
| 15-19 years | 1992 | Both | 366026.1 | 316310.6 | 419290.9 |
| 15-19 years | 1993 | Male | 101347.7 | 84923.9 | 117692.1 |
| 15-19 years | 1993 | Female | 247368.4 | 214200 | 282456.6 |
| 15-19 years | 1993 | Both | 348716.1 | 301022.9 | 397820.3 |
| 15-19 years | 1994 | Male | 97279.45 | 81477.18 | 113028.7 |
| 15-19 years | 1994 | Female | 236963 | 204660.9 | 271639.4 |
| 15-19 years | 1994 | Both | 334242.4 | 287618.6 | 383724.4 |
| 15-19 years | 1995 | Male | 94423.56 | 78854.04 | 109898.3 |
| 15-19 years | 1995 | Female | 229692 | 197127.4 | 263948 |
| 15-19 years | 1995 | Both | 324115.6 | 278695.8 | 372239.8 |
| 15-19 years | 1996 | Male | 93044.95 | 77724.41 | 108273.1 |
| 15-19 years | 1996 | Female | 226753.9 | 194563.2 | 260544.4 |
| 15-19 years | 1996 | Both | 319798.9 | 274902.1 | 367276.9 |
| 15-19 years | 1997 | Male | 92897.3 | 77619.27 | 108083.3 |
| 15-19 years | 1997 | Female | 226817.3 | 194581.6 | 260592.6 |
| 15-19 years | 1997 | Both | 319714.6 | 274756.3 | 367186.5 |
| 15-19 years | 1998 | Male | 93856.62 | 78434.43 | 109185.5 |
| 15-19 years | 1998 | Female | 229198.5 | 196594.9 | 263320.1 |
| 15-19 years | 1998 | Both | 323055.1 | 277560.2 | 371033.8 |
| 15-19 years | 1999 | Male | 95964.34 | 80203.72 | 111628.4 |
| 15-19 years | 1999 | Female | 233919 | 200624 | 268757.5 |
| 15-19 years | 1999 | Both | 329883.3 | 283380.5 | 378895.2 |
| 15-19 years | 2000 | Male | 99433.74 | 83103.65 | 115660.7 |
| 15-19 years | 2000 | Female | 241736.4 | 207337.7 | 277745.2 |
| 15-19 years | 2000 | Both | 341170.2 | 293063.9 | 391885.2 |
| 15-19 years | 2001 | Male | 104512.4 | 88269.68 | 121433 |
| 15-19 years | 2001 | Female | 253832.3 | 218469.1 | 291685.8 |
| 15-19 years | 2001 | Both | 358344.8 | 309153.6 | 409667.1 |
| 15-19 years | 2002 | Male | 110475.4 | 93058.91 | 127898.6 |
| 15-19 years | 2002 | Female | 268735.4 | 231957.7 | 307558.5 |
| 15-19 years | 2002 | Both | 379210.9 | 326884.9 | 431917.7 |
| 15-19 years | 2003 | Male | 116324.8 | 98576.13 | 134908.6 |
| 15-19 years | 2003 | Female | 284030.8 | 246768.2 | 326054.3 |
| 15-19 years | 2003 | Both | 400355.6 | 345444.3 | 458130.8 |
| 15-19 years | 2004 | Male | 120889.6 | 102625.9 | 141146.9 |
| 15-19 years | 2004 | Female | 296526 | 257900 | 340520.1 |
| 15-19 years | 2004 | Both | 417415.6 | 360531.2 | 478718.5 |
| 15-19 years | 2005 | Male | 122947 | 103878.4 | 143645.2 |
| 15-19 years | 2005 | Female | 302645.5 | 262182.8 | 349608.2 |
| 15-19 years | 2005 | Both | 425592.5 | 367538.7 | 489563.7 |
| 15-19 years | 2006 | Male | 121603.4 | 103192.2 | 142342 |
| 15-19 years | 2006 | Female | 299727.4 | 261082.2 | 343606 |
| 15-19 years | 2006 | Both | 421330.8 | 363269.7 | 481837.1 |
| 15-19 years | 2007 | Male | 117661 | 99541.62 | 136713.4 |
| 15-19 years | 2007 | Female | 289853.9 | 250499.9 | 331837.3 |
| 15-19 years | 2007 | Both | 407514.9 | 351271.9 | 464622.3 |
| 15-19 years | 2008 | Male | 111892.2 | 95125.39 | 129299.3 |
| 15-19 years | 2008 | Female | 274777.3 | 236807.2 | 314036 |
| 15-19 years | 2008 | Both | 386669.5 | 332530 | 440148 |
| 15-19 years | 2009 | Male | 105163.1 | 89367.65 | 121746.9 |
| 15-19 years | 2009 | Female | 256719.1 | 221439 | 293796.1 |
| 15-19 years | 2009 | Both | 361882.3 | 310723.7 | 412303 |
| 15-19 years | 2010 | Male | 98304.77 | 83315.15 | 114085.7 |
| 15-19 years | 2010 | Female | 237873.5 | 203762.8 | 273470.8 |
| 15-19 years | 2010 | Both | 336178.3 | 288190.2 | 385281.4 |
| 15-19 years | 2011 | Male | 92201.98 | 78155.51 | 106996.8 |
| 15-19 years | 2011 | Female | 220918.2 | 189303.3 | 254062.1 |
| 15-19 years | 2011 | Both | 313120.1 | 268432.6 | 358836.3 |
| 15-19 years | 2012 | Male | 87051.41 | 73798.11 | 101015.3 |
| 15-19 years | 2012 | Female | 206636.2 | 177104.8 | 237690.4 |
| 15-19 years | 2012 | Both | 293687.6 | 251779.5 | 336544.4 |
| 15-19 years | 2013 | Male | 82792.75 | 70191.78 | 96071.38 |
| 15-19 years | 2013 | Female | 194843.7 | 167012.5 | 224146.3 |
| 15-19 years | 2013 | Both | 277636.4 | 238022 | 318128.1 |
| 15-19 years | 2014 | Male | 79393.03 | 67308.75 | 92126.64 |
| 15-19 years | 2014 | Female | 185459.7 | 158959.5 | 213339.5 |
| 15-19 years | 2014 | Both | 264852.7 | 227063 | 303458 |
| 15-19 years | 2015 | Male | 76892.85 | 65184.28 | 89227.88 |
| 15-19 years | 2015 | Female | 178578.5 | 153028.6 | 205382 |
| 15-19 years | 2015 | Both | 255471.4 | 219018 | 292688.3 |
| 15-19 years | 2016 | Male | 75344.28 | 63863.01 | 87435.35 |
| 15-19 years | 2016 | Female | 174273.7 | 149284.9 | 200361.1 |
| 15-19 years | 2016 | Both | 249617.9 | 213994.7 | 285962.3 |
| 15-19 years | 2017 | Male | 74412.98 | 63063.31 | 86360.31 |
| 15-19 years | 2017 | Female | 171569.1 | 146904.5 | 197169.4 |
| 15-19 years | 2017 | Both | 245982.1 | 210871.3 | 281776.9 |
| 15-19 years | 2018 | Male | 73763.09 | 62559.3 | 85496.86 |
| 15-19 years | 2018 | Female | 169394.2 | 146984.6 | 192402.3 |
| 15-19 years | 2018 | Both | 243157.2 | 209822 | 277331.3 |
| 15-19 years | 2019 | Male | 73175.43 | 61108.12 | 85441.51 |
| 15-19 years | 2019 | Female | 167126.6 | 143220.8 | 191434.7 |
| 15-19 years | 2019 | Both | 240302.1 | 207701.4 | 275970.1 |

## Supplementary Table 2. Incidence rates of Chinese children and adolescents with AD in different age groups from 1990 to 2019

| **Age group** | **Year** | **Sex** | **Value*** | **Lower*** | **Upper*** |
| --- | --- | --- | --- | --- | --- |
| <5 years | 1990 | Male | 770.6701 | 689.177 | 866.4074 |
| <5 years | 1990 | Female | 724.0213 | 648.9937 | 803.8961 |
| <5 years | 1990 | Both | 748.6534 | 672.7964 | 834.6181 |
| <5 years | 1991 | Male | 766.3392 | 686.7736 | 851.9419 |
| <5 years | 1991 | Female | 719.7667 | 648.2626 | 797.8975 |
| <5 years | 1991 | Both | 744.3955 | 672.0858 | 820.3458 |
| <5 years | 1992 | Male | 761.3688 | 684.3364 | 838.7186 |
| <5 years | 1992 | Female | 715.0521 | 645.1262 | 790.881 |
| <5 years | 1992 | Both | 739.5903 | 669.1968 | 811.1603 |
| <5 years | 1993 | Male | 756.0078 | 679.7316 | 833.3898 |
| <5 years | 1993 | Female | 709.8939 | 639.3557 | 786.64 |
| <5 years | 1993 | Both | 734.3815 | 664.7078 | 805.8378 |
| <5 years | 1994 | Male | 751.2397 | 674.2891 | 830.3567 |
| <5 years | 1994 | Female | 705.2363 | 634.8815 | 783.3297 |
| <5 years | 1994 | Both | 729.7316 | 659.7609 | 803.9748 |
| <5 years | 1995 | Male | 748.6056 | 669.8663 | 831.7307 |
| <5 years | 1995 | Female | 702.493 | 630.9887 | 783.5815 |
| <5 years | 1995 | Both | 727.1187 | 657.1969 | 805.2967 |
| <5 years | 1996 | Male | 747.9219 | 669.1758 | 830.8337 |
| <5 years | 1996 | Female | 701.4895 | 630.0261 | 782.6154 |
| <5 years | 1996 | Both | 726.3544 | 656.5092 | 804.4416 |
| <5 years | 1997 | Male | 747.6304 | 668.8487 | 830.3879 |
| <5 years | 1997 | Female | 701.0007 | 629.5356 | 782.1945 |
| <5 years | 1997 | Both | 726.033 | 656.2219 | 804.0692 |
| <5 years | 1998 | Male | 747.7765 | 668.9293 | 830.4483 |
| <5 years | 1998 | Female | 701.1832 | 629.6602 | 782.4886 |
| <5 years | 1998 | Both | 726.2526 | 656.4246 | 804.2891 |
| <5 years | 1999 | Male | 748.2746 | 669.3411 | 830.9245 |
| <5 years | 1999 | Female | 701.7795 | 630.1692 | 783.2074 |
| <5 years | 1999 | Both | 726.847 | 656.9676 | 804.9165 |
| <5 years | 2000 | Male | 748.8738 | 669.8596 | 831.5372 |
| <5 years | 2000 | Female | 702.4752 | 630.7797 | 784.0009 |
| <5 years | 2000 | Both | 727.5357 | 657.5972 | 805.6402 |
| <5 years | 2001 | Male | 750.5361 | 672.3883 | 829.7211 |
| <5 years | 2001 | Female | 705.2546 | 636.0336 | 783.846 |
| <5 years | 2001 | Both | 729.753 | 659.5619 | 804.2254 |
| <5 years | 2002 | Male | 753.7506 | 675.9886 | 831.4211 |
| <5 years | 2002 | Female | 711.1336 | 641.77 | 789.3172 |
| <5 years | 2002 | Both | 734.2289 | 662.6404 | 809.5482 |
| <5 years | 2003 | Male | 757.2968 | 677.7114 | 837.3902 |
| <5 years | 2003 | Female | 717.9061 | 647.7301 | 794.8885 |
| <5 years | 2003 | Both | 739.2878 | 668.0218 | 815.725 |
| <5 years | 2004 | Male | 760.2193 | 680.0289 | 843.8231 |
| <5 years | 2004 | Female | 723.4823 | 651.4783 | 802.38 |
| <5 years | 2004 | Both | 743.4551 | 670.0609 | 821.6548 |
| <5 years | 2005 | Male | 761.508 | 680.2512 | 849.0622 |
| <5 years | 2005 | Female | 725.8189 | 652.1647 | 808.6141 |
| <5 years | 2005 | Both | 745.2519 | 671.1245 | 827.2919 |
| <5 years | 2006 | Male | 760.2692 | 685.2007 | 847.1525 |
| <5 years | 2006 | Female | 724.3648 | 652.4734 | 804.2076 |
| <5 years | 2006 | Both | 743.9439 | 672.6298 | 824.2489 |
| <5 years | 2007 | Male | 756.9687 | 684.2742 | 840.8589 |
| <5 years | 2007 | Female | 720.6368 | 652.4431 | 796.452 |
| <5 years | 2007 | Both | 740.4719 | 671.2352 | 814.5283 |
| <5 years | 2008 | Male | 752.5374 | 680.0886 | 830.8731 |
| <5 years | 2008 | Female | 715.6776 | 645.817 | 791.532 |
| <5 years | 2008 | Both | 735.8133 | 667.0844 | 808.4518 |
| <5 years | 2009 | Male | 748.8357 | 673.5729 | 828.9089 |
| <5 years | 2009 | Female | 711.3873 | 642.3551 | 788.3569 |
| <5 years | 2009 | Both | 731.8436 | 664.514 | 802.9995 |
| <5 years | 2010 | Male | 748.449 | 670.799 | 828.4857 |
| <5 years | 2010 | Female | 710.3651 | 636.1973 | 792.4362 |
| <5 years | 2010 | Both | 731.1529 | 661.2541 | 802.6939 |
| <5 years | 2011 | Male | 747.203 | 669.8154 | 827.0173 |
| <5 years | 2011 | Female | 708.845 | 634.8434 | 790.7011 |
| <5 years | 2011 | Both | 729.7534 | 660.0574 | 801.1242 |
| <5 years | 2012 | Male | 740.5675 | 663.9218 | 819.6017 |
| <5 years | 2012 | Female | 702.4596 | 629.1309 | 783.5467 |
| <5 years | 2012 | Both | 723.1939 | 654.2033 | 793.9004 |
| <5 years | 2013 | Male | 738.3387 | 661.9513 | 817.1035 |
| <5 years | 2013 | Female | 700.3819 | 627.2705 | 781.215 |
| <5 years | 2013 | Both | 720.9914 | 652.2969 | 791.4549 |
| <5 years | 2014 | Male | 743.2876 | 666.3886 | 822.5865 |
| <5 years | 2014 | Female | 705.213 | 631.5924 | 786.6071 |
| <5 years | 2014 | Both | 725.8433 | 656.698 | 796.7456 |
| <5 years | 2015 | Male | 736.9996 | 660.7448 | 815.6328 |
| <5 years | 2015 | Female | 699.3538 | 626.3408 | 780.0801 |
| <5 years | 2015 | Both | 719.7123 | 651.1247 | 790.0116 |
| <5 years | 2016 | Male | 724.6253 | 649.6475 | 801.9267 |
| <5 years | 2016 | Female | 687.7849 | 615.9789 | 767.1837 |
| <5 years | 2016 | Both | 707.674 | 640.206 | 776.8506 |
| <5 years | 2017 | Male | 732.4204 | 656.6324 | 810.5314 |
| <5 years | 2017 | Female | 695.6121 | 622.9901 | 775.9243 |
| <5 years | 2017 | Both | 715.4574 | 647.2187 | 785.4451 |
| <5 years | 2018 | Male | 760.0504 | 684.9766 | 838.8515 |
| <5 years | 2018 | Female | 720.0991 | 651.0879 | 796.8428 |
| <5 years | 2018 | Both | 741.6194 | 672.4422 | 818.2574 |
| <5 years | 2019 | Male | 791.129 | 706.8495 | 887.0717 |
| <5 years | 2019 | Female | 744.9875 | 665.4265 | 832.6408 |
| <5 years | 2019 | Both | 769.8288 | 694.8061 | 854.7759 |
| 5-9 years | 1990 | Male | 291.3255 | 244.049 | 345.5612 |
| 5-9 years | 1990 | Female | 377.6445 | 316.6003 | 437.9649 |
| 5-9 years | 1990 | Both | 332.7551 | 281.626 | 387.6445 |
| 5-9 years | 1991 | Male | 286.586 | 240.8029 | 340.428 |
| 5-9 years | 1991 | Female | 372.6559 | 316.8604 | 431.9204 |
| 5-9 years | 1991 | Both | 327.7621 | 278.832 | 382.0491 |
| 5-9 years | 1992 | Male | 282.2329 | 238.0814 | 332.2832 |
| 5-9 years | 1992 | Female | 368.066 | 312.8219 | 427.1442 |
| 5-9 years | 1992 | Both | 323.1616 | 276.2699 | 376.8489 |
| 5-9 years | 1993 | Male | 278.644 | 235.7999 | 328.3874 |
| 5-9 years | 1993 | Female | 364.276 | 310.8219 | 424.2544 |
| 5-9 years | 1993 | Both | 319.366 | 272.8657 | 372.0221 |
| 5-9 years | 1994 | Male | 276.1928 | 233.5286 | 327.7292 |
| 5-9 years | 1994 | Female | 361.6772 | 308.2855 | 422.8374 |
| 5-9 years | 1994 | Both | 316.7554 | 271.1276 | 369.6525 |
| 5-9 years | 1995 | Male | 275.2483 | 231.2662 | 327.3287 |
| 5-9 years | 1995 | Female | 360.6525 | 304.0464 | 424.5508 |
| 5-9 years | 1995 | Both | 315.6887 | 268.2242 | 370.4812 |
| 5-9 years | 1996 | Male | 275.1579 | 231.1867 | 327.1992 |
| 5-9 years | 1996 | Female | 360.5095 | 303.9325 | 424.3906 |
| 5-9 years | 1996 | Both | 315.4655 | 268.0575 | 370.2206 |
| 5-9 years | 1997 | Male | 275.0318 | 231.0801 | 327.0065 |
| 5-9 years | 1997 | Female | 360.3176 | 303.7959 | 424.172 |
| 5-9 years | 1997 | Both | 315.1708 | 267.8434 | 369.8708 |
| 5-9 years | 1998 | Male | 274.8766 | 230.9516 | 326.7626 |
| 5-9 years | 1998 | Female | 360.0871 | 303.6405 | 423.9071 |
| 5-9 years | 1998 | Both | 314.8123 | 267.5539 | 369.4113 |
| 5-9 years | 1999 | Male | 274.7071 | 230.8234 | 326.4908 |
| 5-9 years | 1999 | Female | 359.8398 | 303.4818 | 423.6216 |
| 5-9 years | 1999 | Both | 314.4225 | 267.2381 | 368.8094 |
| 5-9 years | 2000 | Male | 274.5479 | 230.7665 | 326.2327 |
| 5-9 years | 2000 | Female | 359.6123 | 303.3428 | 423.3577 |
| 5-9 years | 2000 | Both | 314.0507 | 266.962 | 368.2343 |
| 5-9 years | 2001 | Male | 274.8986 | 233.5731 | 324.2525 |
| 5-9 years | 2001 | Female | 359.5814 | 305.659 | 419.8956 |
| 5-9 years | 2001 | Both | 314.08 | 268.1176 | 366.5841 |
| 5-9 years | 2002 | Male | 275.937 | 234.8076 | 323.9394 |
| 5-9 years | 2002 | Female | 359.7869 | 307.3615 | 418.7583 |
| 5-9 years | 2002 | Both | 314.6215 | 269.6704 | 366.2064 |
| 5-9 years | 2003 | Male | 277.2317 | 234.7409 | 324.7334 |
| 5-9 years | 2003 | Female | 360.0884 | 308.1178 | 419.5778 |
| 5-9 years | 2003 | Both | 315.3699 | 270.0227 | 367.8607 |
| 5-9 years | 2004 | Male | 278.3543 | 232.7403 | 326.2652 |
| 5-9 years | 2004 | Female | 360.3446 | 308.3346 | 418.3887 |
| 5-9 years | 2004 | Both | 316.0258 | 268.7037 | 368.9209 |
| 5-9 years | 2005 | Male | 278.8783 | 231.5813 | 328.1852 |
| 5-9 years | 2005 | Female | 360.4208 | 302.9751 | 417.11 |
| 5-9 years | 2005 | Both | 316.2962 | 268.0601 | 367.5456 |
| 5-9 years | 2006 | Male | 278.2164 | 231.0103 | 325.8214 |
| 5-9 years | 2006 | Female | 359.7739 | 307.5197 | 416.1007 |
| 5-9 years | 2006 | Both | 315.6108 | 267.9154 | 367.5989 |
| 5-9 years | 2007 | Male | 276.5197 | 230.348 | 323.3651 |
| 5-9 years | 2007 | Female | 358.3213 | 307.3161 | 415.5637 |
| 5-9 years | 2007 | Both | 314.0019 | 267.3935 | 366.2292 |
| 5-9 years | 2008 | Male | 274.4766 | 229.6664 | 320.2508 |
| 5-9 years | 2008 | Female | 356.6315 | 302.5232 | 415.3993 |
| 5-9 years | 2008 | Both | 312.0974 | 266.8661 | 364.694 |
| 5-9 years | 2009 | Male | 272.7804 | 229.1366 | 318.6679 |
| 5-9 years | 2009 | Female | 355.2711 | 298.2317 | 414.9256 |
| 5-9 years | 2009 | Both | 310.5272 | 263.8844 | 362.7298 |
| 5-9 years | 2010 | Male | 272.126 | 226.7766 | 320.9996 |
| 5-9 years | 2010 | Female | 354.7996 | 295.773 | 417.3919 |
| 5-9 years | 2010 | Both | 309.918 | 261.737 | 364.6052 |
| 5-9 years | 2011 | Male | 272.2002 | 226.8355 | 321.11 |
| 5-9 years | 2011 | Female | 354.9247 | 295.8653 | 417.5642 |
| 5-9 years | 2011 | Both | 309.9619 | 261.7498 | 364.6522 |
| 5-9 years | 2012 | Male | 272.2458 | 226.8637 | 321.2028 |
| 5-9 years | 2012 | Female | 355.0091 | 295.9408 | 417.6624 |
| 5-9 years | 2012 | Both | 309.9743 | 261.7551 | 364.6648 |
| 5-9 years | 2013 | Male | 272.2759 | 226.8758 | 321.2874 |
| 5-9 years | 2013 | Female | 355.0696 | 296.0078 | 417.7142 |
| 5-9 years | 2013 | Both | 309.9858 | 261.7702 | 364.6798 |
| 5-9 years | 2014 | Male | 272.299 | 226.8819 | 321.3648 |
| 5-9 years | 2014 | Female | 355.1167 | 296.0678 | 417.7421 |
| 5-9 years | 2014 | Both | 310.0146 | 261.8035 | 364.7197 |
| 5-9 years | 2015 | Male | 272.3196 | 226.8878 | 321.4328 |
| 5-9 years | 2015 | Female | 355.1556 | 296.1193 | 417.7618 |
| 5-9 years | 2015 | Both | 310.0699 | 261.8582 | 364.7963 |
| 5-9 years | 2016 | Male | 272.3386 | 226.895 | 321.4908 |
| 5-9 years | 2016 | Female | 355.1884 | 296.1624 | 417.7788 |
| 5-9 years | 2016 | Both | 310.1506 | 261.9322 | 364.9076 |
| 5-9 years | 2017 | Male | 272.3478 | 226.8946 | 321.5277 |
| 5-9 years | 2017 | Female | 355.2047 | 296.1886 | 417.7823 |
| 5-9 years | 2017 | Both | 310.238 | 262.0134 | 365.0315 |
| 5-9 years | 2018 | Male | 277.3778 | 231.7075 | 327.1356 |
| 5-9 years | 2018 | Female | 360.5472 | 305.3315 | 419.5462 |
| 5-9 years | 2018 | Both | 315.4973 | 269.3876 | 367.0982 |
| 5-9 years | 2019 | Male | 288.4435 | 238.8321 | 348.8606 |
| 5-9 years | 2019 | Female | 372.2973 | 310.1299 | 436.6056 |
| 5-9 years | 2019 | Both | 326.9651 | 275.257 | 385.2465 |
| 10-14 years | 1990 | Male | 180.4273 | 147.7168 | 217.4671 |
| 10-14 years | 1990 | Female | 327.5895 | 267.1708 | 392.0621 |
| 10-14 years | 1990 | Both | 251.6259 | 207.8526 | 298.4366 |
| 10-14 years | 1991 | Male | 179.9134 | 147.9983 | 215.2013 |
| 10-14 years | 1991 | Female | 326.156 | 270.345 | 385.0789 |
| 10-14 years | 1991 | Both | 250.6575 | 209.2854 | 296.5518 |
| 10-14 years | 1992 | Male | 179.4375 | 147.4127 | 213.228 |
| 10-14 years | 1992 | Female | 324.819 | 273.009 | 381.6908 |
| 10-14 years | 1992 | Both | 249.7747 | 210.0067 | 292.5899 |
| 10-14 years | 1993 | Male | 179.0412 | 146.8671 | 212.7313 |
| 10-14 years | 1993 | Female | 323.6946 | 273.0669 | 381.3747 |
| 10-14 years | 1993 | Both | 248.9933 | 209.9538 | 292.8129 |
| 10-14 years | 1994 | Male | 178.7683 | 146.6653 | 212.0884 |
| 10-14 years | 1994 | Female | 322.912 | 271.1136 | 381.3734 |
| 10-14 years | 1994 | Both | 248.3736 | 208.9504 | 293.1043 |
| 10-14 years | 1995 | Male | 178.6648 | 146.2994 | 213.4084 |
| 10-14 years | 1995 | Female | 322.6083 | 268.5066 | 384.1652 |
| 10-14 years | 1995 | Both | 248.0231 | 207.5885 | 294.0198 |
| 10-14 years | 1996 | Male | 178.6679 | 146.3015 | 213.4044 |
| 10-14 years | 1996 | Female | 322.6084 | 268.509 | 384.1439 |
| 10-14 years | 1996 | Both | 247.8641 | 207.4582 | 293.8488 |
| 10-14 years | 1997 | Male | 178.6751 | 146.3076 | 213.3977 |
| 10-14 years | 1997 | Female | 322.6267 | 268.5361 | 384.1105 |
| 10-14 years | 1997 | Both | 247.7459 | 207.3731 | 293.7331 |
| 10-14 years | 1998 | Male | 178.6783 | 146.3108 | 213.3867 |
| 10-14 years | 1998 | Female | 322.6459 | 268.5689 | 384.0646 |
| 10-14 years | 1998 | Both | 247.6797 | 207.3259 | 293.6778 |
| 10-14 years | 1999 | Male | 178.6688 | 146.2903 | 213.3694 |
| 10-14 years | 1999 | Female | 322.6458 | 268.5839 | 384.0046 |
| 10-14 years | 1999 | Both | 247.6326 | 207.2795 | 293.6375 |
| 10-14 years | 2000 | Male | 178.6366 | 146.2747 | 213.3431 |
| 10-14 years | 2000 | Female | 322.6032 | 268.5556 | 383.9253 |
| 10-14 years | 2000 | Both | 247.5471 | 207.2111 | 293.5393 |
| 10-14 years | 2001 | Male | 178.5478 | 146.3762 | 211.5847 |
| 10-14 years | 2001 | Female | 322.3238 | 271.1675 | 383.0071 |
| 10-14 years | 2001 | Both | 247.2466 | 208.2965 | 292.3315 |
| 10-14 years | 2002 | Male | 178.4022 | 146.9402 | 212.2358 |
| 10-14 years | 2002 | Female | 321.7609 | 271.5449 | 381.2715 |
| 10-14 years | 2002 | Both | 246.7105 | 207.8298 | 291.2491 |
| 10-14 years | 2003 | Male | 178.2311 | 147.4514 | 211.855 |
| 10-14 years | 2003 | Female | 321.0867 | 271.7613 | 380.1598 |
| 10-14 years | 2003 | Both | 246.0421 | 207.1172 | 291.18 |
| 10-14 years | 2004 | Male | 178.0686 | 147.2024 | 212.3156 |
| 10-14 years | 2004 | Female | 320.4794 | 269.6782 | 382.2779 |
| 10-14 years | 2004 | Both | 245.3674 | 205.4665 | 291.7692 |
| 10-14 years | 2005 | Male | 177.9518 | 145.3927 | 213.4904 |
| 10-14 years | 2005 | Female | 320.1219 | 265.691 | 379.6734 |
| 10-14 years | 2005 | Both | 244.8183 | 205.0574 | 290.7766 |
| 10-14 years | 2006 | Male | 177.9755 | 146.8423 | 211.694 |
| 10-14 years | 2006 | Female | 320.4275 | 268.8875 | 380.5771 |
| 10-14 years | 2006 | Both | 244.683 | 205.3687 | 290.4804 |
| 10-14 years | 2007 | Male | 178.149 | 147.2486 | 210.8021 |
| 10-14 years | 2007 | Female | 321.4064 | 272.1765 | 381.7983 |
| 10-14 years | 2007 | Both | 244.9725 | 206.0104 | 290.1185 |
| 10-14 years | 2008 | Male | 178.3892 | 146.5698 | 212.0134 |
| 10-14 years | 2008 | Female | 322.6477 | 272.8118 | 382.6688 |
| 10-14 years | 2008 | Both | 245.4463 | 207.3858 | 289.3049 |
| 10-14 years | 2009 | Male | 178.6141 | 146.1882 | 212.678 |
| 10-14 years | 2009 | Female | 323.74 | 273.595 | 383.1203 |
| 10-14 years | 2009 | Both | 245.8773 | 207.6539 | 289.9167 |
| 10-14 years | 2010 | Male | 178.7432 | 144.8961 | 214.8857 |
| 10-14 years | 2010 | Female | 324.2768 | 272.6453 | 386.9251 |
| 10-14 years | 2010 | Both | 246.049 | 206.7284 | 291.7081 |
| 10-14 years | 2011 | Male | 178.8173 | 144.9087 | 215.0025 |
| 10-14 years | 2011 | Female | 324.4386 | 272.7127 | 387.1412 |
| 10-14 years | 2011 | Both | 246.0752 | 206.7668 | 291.6859 |
| 10-14 years | 2012 | Male | 178.913 | 144.9242 | 215.1536 |
| 10-14 years | 2012 | Female | 324.6412 | 272.7979 | 387.4122 |
| 10-14 years | 2012 | Both | 246.1566 | 206.8577 | 291.7909 |
| 10-14 years | 2013 | Male | 179.0148 | 144.9431 | 215.3133 |
| 10-14 years | 2013 | Female | 324.8555 | 272.894 | 387.6952 |
| 10-14 years | 2013 | Both | 246.2512 | 206.915 | 291.954 |
| 10-14 years | 2014 | Male | 179.1109 | 144.9663 | 215.4604 |
| 10-14 years | 2014 | Female | 325.0598 | 273.0556 | 387.9552 |
| 10-14 years | 2014 | Both | 246.3274 | 206.9295 | 292.0815 |
| 10-14 years | 2015 | Male | 179.1897 | 144.9924 | 215.5768 |
| 10-14 years | 2015 | Female | 325.2329 | 273.2004 | 388.1638 |
| 10-14 years | 2015 | Both | 246.3589 | 206.9187 | 292.1355 |
| 10-14 years | 2016 | Male | 179.2406 | 145.0178 | 215.6474 |
| 10-14 years | 2016 | Female | 325.3542 | 273.307 | 388.298 |
| 10-14 years | 2016 | Both | 246.326 | 206.8687 | 292.1011 |
| 10-14 years | 2017 | Male | 179.2716 | 145.0416 | 215.6859 |
| 10-14 years | 2017 | Female | 325.4368 | 273.3843 | 388.379 |
| 10-14 years | 2017 | Both | 246.2694 | 206.8095 | 292.0235 |
| 10-14 years | 2018 | Male | 180.229 | 149.2384 | 214.2597 |
| 10-14 years | 2018 | Female | 327.4157 | 274.6602 | 386.0227 |
| 10-14 years | 2018 | Both | 247.6154 | 207.1748 | 292.1561 |
| 10-14 years | 2019 | Male | 182.3067 | 150.2518 | 218.8319 |
| 10-14 years | 2019 | Female | 331.696 | 274.5655 | 396.302 |
| 10-14 years | 2019 | Both | 250.6707 | 208.3686 | 296.7901 |
| 15-19 years | 1990 | Male | 179.759 | 152.0072 | 209.7609 |
| 15-19 years | 1990 | Female | 461.9587 | 395.1502 | 532.2906 |
| 15-19 years | 1990 | Both | 317.0358 | 273.3798 | 366.2375 |
| 15-19 years | 1991 | Male | 180.0339 | 153.2396 | 209.5148 |
| 15-19 years | 1991 | Female | 462.1791 | 396.7219 | 529.2857 |
| 15-19 years | 1991 | Both | 317.5264 | 274.6856 | 366.2386 |
| 15-19 years | 1992 | Male | 180.2845 | 152.3539 | 209.2171 |
| 15-19 years | 1992 | Female | 462.3848 | 400.5102 | 526.7447 |
| 15-19 years | 1992 | Both | 317.8948 | 274.7167 | 364.1554 |
| 15-19 years | 1993 | Male | 180.4891 | 151.2401 | 209.5966 |
| 15-19 years | 1993 | Female | 462.5649 | 400.5419 | 528.1778 |
| 15-19 years | 1993 | Both | 318.0867 | 274.5825 | 362.8778 |
| 15-19 years | 1994 | Male | 180.625 | 151.2839 | 209.8677 |
| 15-19 years | 1994 | Female | 462.6928 | 399.62 | 530.4019 |
| 15-19 years | 1994 | Both | 318.1109 | 273.7373 | 365.2048 |
| 15-19 years | 1995 | Male | 180.6679 | 150.8775 | 210.2768 |
| 15-19 years | 1995 | Female | 462.7421 | 397.1367 | 531.7548 |
| 15-19 years | 1995 | Both | 318.0698 | 273.4972 | 365.2964 |
| 15-19 years | 1996 | Male | 180.6571 | 150.9105 | 210.2243 |
| 15-19 years | 1996 | Female | 462.7497 | 397.0562 | 531.7077 |
| 15-19 years | 1996 | Both | 318.1918 | 273.5207 | 365.4312 |
| 15-19 years | 1997 | Male | 180.6508 | 150.9407 | 210.182 |
| 15-19 years | 1997 | Female | 462.7623 | 396.9936 | 531.6721 |
| 15-19 years | 1997 | Both | 318.322 | 273.5596 | 365.5871 |
| 15-19 years | 1998 | Male | 180.6474 | 150.964 | 210.1512 |
| 15-19 years | 1998 | Female | 462.773 | 396.9433 | 531.6676 |
| 15-19 years | 1998 | Both | 318.3345 | 273.5044 | 365.6121 |
| 15-19 years | 1999 | Male | 180.6483 | 150.9797 | 210.1352 |
| 15-19 years | 1999 | Female | 462.801 | 396.9279 | 531.7278 |
| 15-19 years | 1999 | Both | 318.2163 | 273.3582 | 365.4948 |
| 15-19 years | 2000 | Male | 180.6574 | 150.9879 | 210.1396 |
| 15-19 years | 2000 | Female | 462.8762 | 397.0097 | 531.8257 |
| 15-19 years | 2000 | Both | 318.0635 | 273.2154 | 365.3437 |
| 15-19 years | 2001 | Male | 180.6014 | 152.5333 | 209.8408 |
| 15-19 years | 2001 | Female | 463.1191 | 398.5986 | 532.1831 |
| 15-19 years | 2001 | Both | 318.0243 | 274.3681 | 363.5719 |
| 15-19 years | 2002 | Male | 180.4398 | 151.9934 | 208.8971 |
| 15-19 years | 2002 | Female | 463.5719 | 400.1298 | 530.5422 |
| 15-19 years | 2002 | Both | 318.1399 | 274.2409 | 362.3584 |
| 15-19 years | 2003 | Male | 180.2367 | 152.7365 | 209.031 |
| 15-19 years | 2003 | Female | 464.1871 | 403.2893 | 532.8654 |
| 15-19 years | 2003 | Both | 318.4278 | 274.7533 | 364.38 |
| 15-19 years | 2004 | Male | 180.0591 | 152.8562 | 210.2314 |
| 15-19 years | 2004 | Female | 464.9209 | 404.3594 | 533.8988 |
| 15-19 years | 2004 | Both | 318.8357 | 275.3855 | 365.6608 |
| 15-19 years | 2005 | Male | 179.9743 | 152.0609 | 210.273 |
| 15-19 years | 2005 | Female | 465.7253 | 403.4594 | 537.9937 |
| 15-19 years | 2005 | Both | 319.2806 | 275.7285 | 367.2719 |
| 15-19 years | 2006 | Male | 180.2914 | 152.9946 | 211.0387 |
| 15-19 years | 2006 | Female | 467.7119 | 407.4077 | 536.1826 |
| 15-19 years | 2006 | Both | 320.3256 | 276.1834 | 366.3268 |
| 15-19 years | 2007 | Male | 181.0615 | 153.1786 | 210.38 |
| 15-19 years | 2007 | Female | 471.2477 | 407.2656 | 539.5048 |
| 15-19 years | 2007 | Both | 322.1671 | 277.7034 | 367.3142 |
| 15-19 years | 2008 | Male | 181.9598 | 154.6934 | 210.2673 |
| 15-19 years | 2008 | Female | 475.1341 | 409.4778 | 543.0187 |
| 15-19 years | 2008 | Both | 324.0491 | 278.6774 | 368.8668 |
| 15-19 years | 2009 | Male | 182.6704 | 155.2334 | 211.4768 |
| 15-19 years | 2009 | Female | 478.187 | 412.4712 | 547.2496 |
| 15-19 years | 2009 | Both | 325.2705 | 279.2876 | 370.5901 |
| 15-19 years | 2010 | Male | 182.8882 | 155.0012 | 212.2474 |
| 15-19 years | 2010 | Female | 479.2366 | 410.5148 | 550.9533 |
| 15-19 years | 2010 | Both | 325.1642 | 278.7484 | 372.6586 |
| 15-19 years | 2011 | Male | 182.7755 | 154.9307 | 212.1039 |
| 15-19 years | 2011 | Female | 478.9363 | 410.3973 | 550.7902 |
| 15-19 years | 2011 | Both | 324.2339 | 277.9602 | 371.5726 |
| 15-19 years | 2012 | Male | 182.6969 | 154.8819 | 212.0034 |
| 15-19 years | 2012 | Female | 478.7284 | 410.311 | 550.6739 |
| 15-19 years | 2012 | Both | 323.4034 | 277.2549 | 370.5964 |
| 15-19 years | 2013 | Male | 182.6572 | 154.857 | 211.9525 |
| 15-19 years | 2013 | Female | 478.6317 | 410.2646 | 550.6135 |
| 15-19 years | 2013 | Both | 322.7005 | 276.6562 | 369.7646 |
| 15-19 years | 2014 | Male | 182.6601 | 154.8577 | 211.9564 |
| 15-19 years | 2014 | Female | 478.6599 | 410.2644 | 550.616 |
| 15-19 years | 2014 | Both | 322.1641 | 276.1971 | 369.1231 |
| 15-19 years | 2015 | Male | 182.7094 | 154.888 | 212.0193 |
| 15-19 years | 2015 | Female | 478.8279 | 410.3201 | 550.6968 |
| 15-19 years | 2015 | Both | 321.8346 | 275.9118 | 368.7193 |
| 15-19 years | 2016 | Male | 182.8049 | 154.9484 | 212.141 |
| 15-19 years | 2016 | Female | 479.1381 | 410.4354 | 550.8614 |
| 15-19 years | 2016 | Both | 321.7224 | 275.809 | 368.5651 |
| 15-19 years | 2017 | Male | 182.9256 | 155.0253 | 212.2951 |
| 15-19 years | 2017 | Female | 479.5281 | 410.5916 | 551.0798 |
| 15-19 years | 2017 | Both | 321.7214 | 275.7997 | 368.5375 |
| 15-19 years | 2018 | Male | 182.8423 | 155.0706 | 211.9277 |
| 15-19 years | 2018 | Female | 478.8876 | 415.5345 | 543.9331 |
| 15-19 years | 2018 | Both | 321.1485 | 277.1212 | 366.2837 |
| 15-19 years | 2019 | Male | 182.5017 | 152.4055 | 213.0937 |
| 15-19 years | 2019 | Female | 476.9532 | 408.7296 | 546.3244 |
| 15-19 years | 2019 | Both | 319.8219 | 276.4331 | 367.2931 |

*Per 100,000

## Supplementary Table 3. Prevalence cases of Chinese children and adolescents with AD in different age groups from 1990 to 2019

| **Age group** | **Year** | **Sex** | **Value** | **Lower** | **Upper** |
| --- | --- | --- | --- | --- | --- |
| <5 years | 1990 | Male | 1518126 | 1385501 | 1665181 |
| <5 years | 1990 | Female | 1189607 | 1079023 | 1309715 |
| <5 years | 1990 | Both | 2707733 | 2488209 | 2951710 |
| <5 years | 1991 | Male | 1517952 | 1395878 | 1658280 |
| <5 years | 1991 | Female | 1185478 | 1078361 | 1293000 |
| <5 years | 1991 | Both | 2703430 | 2485042 | 2937880 |
| <5 years | 1992 | Male | 1496064 | 1373814 | 1628587 |
| <5 years | 1992 | Female | 1163892 | 1062076 | 1266946 |
| <5 years | 1992 | Both | 2659956 | 2443579 | 2888362 |
| <5 years | 1993 | Male | 1454740 | 1333102 | 1583841 |
| <5 years | 1993 | Female | 1126037 | 1027312 | 1228941 |
| <5 years | 1993 | Both | 2580777 | 2371760 | 2805675 |
| <5 years | 1994 | Male | 1400718 | 1283557 | 1522201 |
| <5 years | 1994 | Female | 1077727 | 979622.2 | 1180902 |
| <5 years | 1994 | Both | 2478445 | 2275866 | 2697777 |
| <5 years | 1995 | Male | 1343322 | 1225892 | 1468425 |
| <5 years | 1995 | Female | 1026693 | 926489.9 | 1126879 |
| <5 years | 1995 | Both | 2370014 | 2176281 | 2584028 |
| <5 years | 1996 | Male | 1289978 | 1177078 | 1410307 |
| <5 years | 1996 | Female | 979584.7 | 883940.9 | 1075291 |
| <5 years | 1996 | Both | 2269563 | 2084193 | 2474372 |
| <5 years | 1997 | Male | 1240073 | 1131432 | 1355687 |
| <5 years | 1997 | Female | 936383 | 844932.5 | 1027956 |
| <5 years | 1997 | Both | 2176456 | 1998821 | 2372748 |
| <5 years | 1998 | Male | 1193928 | 1089247 | 1305184 |
| <5 years | 1998 | Female | 897195.9 | 809560.9 | 984991.3 |
| <5 years | 1998 | Both | 2091124 | 1920558 | 2279631 |
| <5 years | 1999 | Male | 1151352 | 1050350 | 1258591 |
| <5 years | 1999 | Female | 861554.3 | 777402.8 | 945886 |
| <5 years | 1999 | Both | 2012907 | 1848802 | 2194297 |
| <5 years | 2000 | Male | 1112352 | 1014745 | 1215915 |
| <5 years | 2000 | Female | 829286.1 | 748302.4 | 910456.9 |
| <5 years | 2000 | Both | 1941638 | 1783303 | 2116560 |
| <5 years | 2001 | Male | 1078460 | 987418.5 | 1176104 |
| <5 years | 2001 | Female | 802371.5 | 731026.3 | 878423.9 |
| <5 years | 2001 | Both | 1880831 | 1726861 | 2050772 |
| <5 years | 2002 | Male | 1050712 | 963356.1 | 1145345 |
| <5 years | 2002 | Female | 781823.7 | 714538 | 854745.4 |
| <5 years | 2002 | Both | 1832536 | 1681749 | 1995559 |
| <5 years | 2003 | Male | 1028112 | 943356.5 | 1119408 |
| <5 years | 2003 | Female | 765709.9 | 700062.6 | 840136 |
| <5 years | 2003 | Both | 1793822 | 1647417 | 1950747 |
| <5 years | 2004 | Male | 1009613 | 924477.3 | 1101733 |
| <5 years | 2004 | Female | 752073.5 | 684114.3 | 826372.6 |
| <5 years | 2004 | Both | 1761687 | 1617649 | 1921808 |
| <5 years | 2005 | Male | 993257.6 | 906966.5 | 1090607 |
| <5 years | 2005 | Female | 738483.6 | 669606.6 | 814309.9 |
| <5 years | 2005 | Both | 1731741 | 1586658 | 1892447 |
| <5 years | 2006 | Male | 976888.7 | 897020.1 | 1067014 |
| <5 years | 2006 | Female | 723678 | 658199.9 | 795132 |
| <5 years | 2006 | Both | 1700567 | 1563642 | 1855212 |
| <5 years | 2007 | Male | 961396.3 | 883895.1 | 1046457 |
| <5 years | 2007 | Female | 709724.1 | 646252.3 | 780001.2 |
| <5 years | 2007 | Both | 1671120 | 1542917 | 1815948 |
| <5 years | 2008 | Male | 947849 | 873940.7 | 1029455 |
| <5 years | 2008 | Female | 697934 | 639008.8 | 768247.2 |
| <5 years | 2008 | Both | 1645783 | 1518785 | 1790177 |
| <5 years | 2009 | Male | 937801.3 | 861762.6 | 1018191 |
| <5 years | 2009 | Female | 689759.3 | 631836.1 | 761170 |
| <5 years | 2009 | Both | 1627561 | 1501885 | 1770263 |
| <5 years | 2010 | Male | 932125.3 | 851937.8 | 1017880 |
| <5 years | 2010 | Female | 686060.7 | 626478.2 | 760914 |
| <5 years | 2010 | Both | 1618186 | 1489750 | 1759349 |
| <5 years | 2011 | Male | 928335 | 848450.7 | 1013716 |
| <5 years | 2011 | Female | 685045.3 | 625575.8 | 759722.9 |
| <5 years | 2011 | Both | 1613380 | 1485335 | 1754157 |
| <5 years | 2012 | Male | 927507.1 | 847677.4 | 1012793 |
| <5 years | 2012 | Female | 687098.7 | 627470.9 | 761948 |
| <5 years | 2012 | Both | 1614606 | 1486488 | 1755552 |
| <5 years | 2013 | Male | 937287.1 | 856599.2 | 1023459 |
| <5 years | 2013 | Female | 697536.6 | 637014.4 | 773493.7 |
| <5 years | 2013 | Both | 1634824 | 1505102 | 1777633 |
| <5 years | 2014 | Male | 953179.2 | 871105.2 | 1040804 |
| <5 years | 2014 | Female | 712749.1 | 650908.3 | 790357.5 |
| <5 years | 2014 | Both | 1665928 | 1533776 | 1811580 |
| <5 years | 2015 | Male | 967252.7 | 883942.7 | 1056157 |
| <5 years | 2015 | Female | 726444.8 | 663409.8 | 805547.2 |
| <5 years | 2015 | Both | 1693698 | 1559436 | 1841903 |
| <5 years | 2016 | Male | 993935.3 | 908292.7 | 1085322 |
| <5 years | 2016 | Female | 749440 | 684398 | 831049.8 |
| <5 years | 2016 | Both | 1743375 | 1605272 | 1896052 |
| <5 years | 2017 | Male | 1038582 | 949047.7 | 1134135 |
| <5 years | 2017 | Female | 785866.1 | 717644.2 | 871450.2 |
| <5 years | 2017 | Both | 1824449 | 1680022 | 1984352 |
| <5 years | 2018 | Male | 1084830 | 998868.3 | 1179384 |
| <5 years | 2018 | Female | 821448.3 | 753384.2 | 897423.9 |
| <5 years | 2018 | Both | 1906278 | 1761052 | 2078187 |
| <5 years | 2019 | Male | 1123482 | 1026468 | 1242483 |
| <5 years | 2019 | Female | 848617.4 | 772852.1 | 932983.9 |
| <5 years | 2019 | Both | 1972099 | 1807762 | 2159404 |
| 5-9 years | 1990 | Male | 1594924 | 1456149 | 1750641 |
| 5-9 years | 1990 | Female | 1477435 | 1345012 | 1623567 |
| 5-9 years | 1990 | Both | 3072359 | 2817657 | 3342573 |
| 5-9 years | 1991 | Male | 1613511 | 1479333 | 1761490 |
| 5-9 years | 1991 | Female | 1484819 | 1354775 | 1626841 |
| 5-9 years | 1991 | Both | 3098330 | 2853886 | 3362763 |
| 5-9 years | 1992 | Male | 1643649 | 1509559 | 1784345 |
| 5-9 years | 1992 | Female | 1502502 | 1372401 | 1637041 |
| 5-9 years | 1992 | Both | 3146151 | 2900941 | 3397657 |
| 5-9 years | 1993 | Male | 1679257 | 1544161 | 1823576 |
| 5-9 years | 1993 | Female | 1526517 | 1395034 | 1663479 |
| 5-9 years | 1993 | Both | 3205774 | 2956227 | 3460490 |
| 5-9 years | 1994 | Male | 1711008 | 1574441 | 1863456 |
| 5-9 years | 1994 | Female | 1548402 | 1407060 | 1685525 |
| 5-9 years | 1994 | Both | 3259410 | 2998529 | 3532243 |
| 5-9 years | 1995 | Male | 1726943 | 1582149 | 1890322 |
| 5-9 years | 1995 | Female | 1556372 | 1403025 | 1700886 |
| 5-9 years | 1995 | Both | 3283315 | 3016062 | 3572121 |
| 5-9 years | 1996 | Male | 1709955 | 1566681 | 1871608 |
| 5-9 years | 1996 | Female | 1533110 | 1382155 | 1675563 |
| 5-9 years | 1996 | Both | 3243065 | 2979358 | 3527981 |
| 5-9 years | 1997 | Male | 1659759 | 1520843 | 1816422 |
| 5-9 years | 1997 | Female | 1478381 | 1332774 | 1616039 |
| 5-9 years | 1997 | Both | 3138140 | 2883300 | 3413243 |
| 5-9 years | 1998 | Male | 1585554 | 1453042 | 1734878 |
| 5-9 years | 1998 | Female | 1401065 | 1263055 | 1531944 |
| 5-9 years | 1998 | Both | 2986619 | 2744466 | 3247669 |
| 5-9 years | 1999 | Male | 1499839 | 1374699 | 1640712 |
| 5-9 years | 1999 | Female | 1313813 | 1184399 | 1436976 |
| 5-9 years | 1999 | Both | 2813653 | 2585339 | 3058748 |
| 5-9 years | 2000 | Male | 1416471 | 1298476 | 1549160 |
| 5-9 years | 2000 | Female | 1230204 | 1109036 | 1345908 |
| 5-9 years | 2000 | Both | 2646675 | 2431330 | 2876467 |
| 5-9 years | 2001 | Male | 1350191 | 1238129 | 1466927 |
| 5-9 years | 2001 | Female | 1165632 | 1058948 | 1275160 |
| 5-9 years | 2001 | Both | 2515823 | 2311065 | 2728224 |
| 5-9 years | 2002 | Male | 1298276 | 1192033 | 1405374 |
| 5-9 years | 2002 | Female | 1117134 | 1021115 | 1218352 |
| 5-9 years | 2002 | Both | 2415410 | 2220669 | 2615103 |
| 5-9 years | 2003 | Male | 1256259 | 1155860 | 1355848 |
| 5-9 years | 2003 | Female | 1079025 | 985635.7 | 1180323 |
| 5-9 years | 2003 | Both | 2335283 | 2148764 | 2533312 |
| 5-9 years | 2004 | Male | 1221111 | 1120033 | 1324019 |
| 5-9 years | 2004 | Female | 1047533 | 954512 | 1149011 |
| 5-9 years | 2004 | Both | 2268644 | 2082225 | 2463917 |
| 5-9 years | 2005 | Male | 1191691 | 1088739 | 1298578 |
| 5-9 years | 2005 | Female | 1020862 | 927930.2 | 1123237 |
| 5-9 years | 2005 | Both | 2212554 | 2028977 | 2408783 |
| 5-9 years | 2006 | Male | 1167809 | 1069222 | 1267334 |
| 5-9 years | 2006 | Female | 998678.6 | 914123.9 | 1091382 |
| 5-9 years | 2006 | Both | 2166487 | 1992484 | 2353409 |
| 5-9 years | 2007 | Male | 1148411 | 1056150 | 1245990 |
| 5-9 years | 2007 | Female | 980205.4 | 897241.9 | 1066736 |
| 5-9 years | 2007 | Both | 2128616 | 1958394 | 2306962 |
| 5-9 years | 2008 | Male | 1133491 | 1044676 | 1233112 |
| 5-9 years | 2008 | Female | 965501.3 | 882436 | 1050631 |
| 5-9 years | 2008 | Both | 2098993 | 1931689 | 2271045 |
| 5-9 years | 2009 | Male | 1122627 | 1035853 | 1226479 |
| 5-9 years | 2009 | Female | 954268.8 | 868474.4 | 1037803 |
| 5-9 years | 2009 | Both | 2076896 | 1909105 | 2247434 |
| 5-9 years | 2010 | Male | 1114495 | 1020191 | 1223312 |
| 5-9 years | 2010 | Female | 945410 | 852997.6 | 1028107 |
| 5-9 years | 2010 | Both | 2059905 | 1893389 | 2230105 |
| 5-9 years | 2011 | Male | 1104819 | 1011373 | 1212632 |
| 5-9 years | 2011 | Female | 934940.6 | 843566.5 | 1016663 |
| 5-9 years | 2011 | Both | 2039759 | 1874759 | 2208239 |
| 5-9 years | 2012 | Male | 1094258 | 1001758 | 1200954 |
| 5-9 years | 2012 | Female | 923891.1 | 833636.7 | 1004578 |
| 5-9 years | 2012 | Both | 2018149 | 1854789 | 2184798 |
| 5-9 years | 2013 | Male | 1084972 | 993315.9 | 1190661 |
| 5-9 years | 2013 | Female | 914761 | 825450.3 | 994572.8 |
| 5-9 years | 2013 | Both | 1999733 | 1837759 | 2164840 |
| 5-9 years | 2014 | Male | 1077812 | 986819.9 | 1182763 |
| 5-9 years | 2014 | Female | 908625.5 | 819966.5 | 987824.5 |
| 5-9 years | 2014 | Both | 1986437 | 1825440 | 2150458 |
| 5-9 years | 2015 | Male | 1071289 | 980899.1 | 1175597 |
| 5-9 years | 2015 | Female | 904398.5 | 816197.6 | 983161.1 |
| 5-9 years | 2015 | Both | 1975688 | 1815468 | 2138864 |
| 5-9 years | 2016 | Male | 1068658 | 978531.9 | 1172705 |
| 5-9 years | 2016 | Female | 904691 | 816498.6 | 983422.7 |
| 5-9 years | 2016 | Both | 1973349 | 1813231 | 2136360 |
| 5-9 years | 2017 | Male | 1077262 | 986441.2 | 1182142 |
| 5-9 years | 2017 | Female | 915365.2 | 826166.2 | 994986.8 |
| 5-9 years | 2017 | Both | 1992627 | 1830859 | 2157240 |
| 5-9 years | 2018 | Male | 1106583 | 1019746 | 1208437 |
| 5-9 years | 2018 | Female | 943133.2 | 864866.7 | 1025058 |
| 5-9 years | 2018 | Both | 2049716 | 1888110 | 2217954 |
| 5-9 years | 2019 | Male | 1150404 | 1046246 | 1262985 |
| 5-9 years | 2019 | Female | 982193.7 | 892261.6 | 1081695 |
| 5-9 years | 2019 | Both | 2132598 | 1959992 | 2324859 |
| 10-14 years | 1990 | Male | 1018139 | 913955.8 | 1114444 |
| 10-14 years | 1990 | Female | 1165809 | 1059723 | 1286024 |
| 10-14 years | 1990 | Both | 2183948 | 1987291 | 2386672 |
| 10-14 years | 1991 | Male | 994318.8 | 903905.4 | 1083626 |
| 10-14 years | 1991 | Female | 1139209 | 1035909 | 1251947 |
| 10-14 years | 1991 | Both | 2133528 | 1951765 | 2325921 |
| 10-14 years | 1992 | Male | 984132.1 | 899470.9 | 1072143 |
| 10-14 years | 1992 | Female | 1128684 | 1021597 | 1235588 |
| 10-14 years | 1992 | Both | 2112816 | 1931773 | 2295863 |
| 10-14 years | 1993 | Male | 986175.9 | 901532.2 | 1074628 |
| 10-14 years | 1993 | Female | 1130646 | 1027078 | 1236698 |
| 10-14 years | 1993 | Both | 2116822 | 1937113 | 2300724 |
| 10-14 years | 1994 | Male | 1000842 | 913467.6 | 1093839 |
| 10-14 years | 1994 | Female | 1144661 | 1035954 | 1254894 |
| 10-14 years | 1994 | Both | 2145503 | 1954980 | 2334271 |
| 10-14 years | 1995 | Male | 1030329 | 938097.5 | 1133414 |
| 10-14 years | 1995 | Female | 1173583 | 1055173 | 1288546 |
| 10-14 years | 1995 | Both | 2203912 | 2003437 | 2399677 |
| 10-14 years | 1996 | Male | 1075300 | 979002.6 | 1182898 |
| 10-14 years | 1996 | Female | 1219312 | 1096240 | 1338712 |
| 10-14 years | 1996 | Both | 2294612 | 2085900 | 2498560 |
| 10-14 years | 1997 | Male | 1129374 | 1028167 | 1242414 |
| 10-14 years | 1997 | Female | 1275970 | 1147067 | 1400790 |
| 10-14 years | 1997 | Both | 2405343 | 2186636 | 2619410 |
| 10-14 years | 1998 | Male | 1184331 | 1078123 | 1302909 |
| 10-14 years | 1998 | Female | 1335226 | 1200210 | 1465680 |
| 10-14 years | 1998 | Both | 2519557 | 2290571 | 2744110 |
| 10-14 years | 1999 | Male | 1228775 | 1118509 | 1351831 |
| 10-14 years | 1999 | Female | 1383783 | 1243730 | 1518846 |
| 10-14 years | 1999 | Both | 2612558 | 2375209 | 2845643 |
| 10-14 years | 2000 | Male | 1249036 | 1136899 | 1374123 |
| 10-14 years | 2000 | Female | 1404810 | 1262544 | 1541892 |
| 10-14 years | 2000 | Both | 2653846 | 2412758 | 2890644 |
| 10-14 years | 2001 | Male | 1232789 | 1126077 | 1347361 |
| 10-14 years | 2001 | Female | 1381682 | 1247535 | 1508677 |
| 10-14 years | 2001 | Both | 2614471 | 2381159 | 2838471 |
| 10-14 years | 2002 | Male | 1188481 | 1087646 | 1294589 |
| 10-14 years | 2002 | Female | 1324336 | 1201865 | 1443008 |
| 10-14 years | 2002 | Both | 2512817 | 2295838 | 2723102 |
| 10-14 years | 2003 | Male | 1125718 | 1031514 | 1228672 |
| 10-14 years | 2003 | Female | 1244712 | 1125121 | 1356973 |
| 10-14 years | 2003 | Both | 2370430 | 2165620 | 2567462 |
| 10-14 years | 2004 | Male | 1054837 | 962585.7 | 1153408 |
| 10-14 years | 2004 | Female | 1156029 | 1043268 | 1263619 |
| 10-14 years | 2004 | Both | 2210866 | 2018473 | 2402291 |
| 10-14 years | 2005 | Male | 985428.2 | 895929.2 | 1081834 |
| 10-14 years | 2005 | Female | 1070202 | 962061.8 | 1177997 |
| 10-14 years | 2005 | Both | 2055630 | 1872384 | 2240646 |
| 10-14 years | 2006 | Male | 924555.5 | 843922.9 | 1013514 |
| 10-14 years | 2006 | Female | 996417.6 | 904509.8 | 1088846 |
| 10-14 years | 2006 | Both | 1920973 | 1753382 | 2087100 |
| 10-14 years | 2007 | Male | 871614.9 | 795938.8 | 951693.1 |
| 10-14 years | 2007 | Female | 933563.2 | 847208.3 | 1015683 |
| 10-14 years | 2007 | Both | 1805178 | 1649522 | 1957397 |
| 10-14 years | 2008 | Male | 826742.6 | 754658.2 | 898947.7 |
| 10-14 years | 2008 | Female | 880930.1 | 798633.5 | 957825.1 |
| 10-14 years | 2008 | Both | 1707673 | 1562599 | 1846232 |
| 10-14 years | 2009 | Male | 790600.7 | 718782.5 | 862770.2 |
| 10-14 years | 2009 | Female | 838781.6 | 757926.4 | 916107.7 |
| 10-14 years | 2009 | Both | 1629382 | 1487146 | 1762804 |
| 10-14 years | 2010 | Male | 764824.7 | 693986.8 | 840874.8 |
| 10-14 years | 2010 | Female | 808585.7 | 730958.9 | 888450.5 |
| 10-14 years | 2010 | Both | 1573410 | 1432023 | 1710581 |
| 10-14 years | 2011 | Male | 749855.6 | 680464.3 | 824475.7 |
| 10-14 years | 2011 | Female | 790898.7 | 714866.1 | 869023.2 |
| 10-14 years | 2011 | Both | 1540754 | 1402028 | 1675281 |
| 10-14 years | 2012 | Male | 741096.8 | 672592.3 | 814922.2 |
| 10-14 years | 2012 | Female | 780365.7 | 705230.4 | 857456.8 |
| 10-14 years | 2012 | Both | 1521462 | 1384423 | 1654543 |
| 10-14 years | 2013 | Male | 736033.4 | 667955 | 809401.6 |
| 10-14 years | 2013 | Female | 773849.8 | 699482 | 850302.6 |
| 10-14 years | 2013 | Both | 1509883 | 1373975 | 1642174 |
| 10-14 years | 2014 | Male | 732598.8 | 664763.1 | 805609.8 |
| 10-14 years | 2014 | Female | 768841.2 | 695079.3 | 844801.9 |
| 10-14 years | 2014 | Both | 1501440 | 1366068 | 1633152 |
| 10-14 years | 2015 | Male | 728728.7 | 661203.7 | 801317.3 |
| 10-14 years | 2015 | Female | 762957.1 | 689854.4 | 838335.3 |
| 10-14 years | 2015 | Both | 1491686 | 1357069 | 1622538 |
| 10-14 years | 2016 | Male | 722601.5 | 655632.2 | 794525.6 |
| 10-14 years | 2016 | Female | 754215.9 | 682004.1 | 828723.2 |
| 10-14 years | 2016 | Both | 1476817 | 1343541 | 1606321 |
| 10-14 years | 2017 | Male | 715867.1 | 649532.7 | 787058.4 |
| 10-14 years | 2017 | Female | 744989.8 | 673686.2 | 818515.3 |
| 10-14 years | 2017 | Both | 1460857 | 1329092 | 1588894 |
| 10-14 years | 2018 | Male | 719644 | 654740.5 | 783016.9 |
| 10-14 years | 2018 | Female | 746787.2 | 680585.3 | 814977.9 |
| 10-14 years | 2018 | Both | 1466431 | 1344652 | 1592406 |
| 10-14 years | 2019 | Male | 736207 | 667318.9 | 805849.6 |
| 10-14 years | 2019 | Female | 762156.2 | 687556.8 | 837802.5 |
| 10-14 years | 2019 | Both | 1498363 | 1366337 | 1636636 |
| 15-19 years | 1990 | Male | 924556.7 | 828568.8 | 1032760 |
| 15-19 years | 1990 | Female | 1450721 | 1300209 | 1622788 |
| 15-19 years | 1990 | Both | 2375277 | 2146874 | 2643835 |
| 15-19 years | 1991 | Male | 875322.7 | 789475.5 | 974855 |
| 15-19 years | 1991 | Female | 1381710 | 1241616 | 1543996 |
| 15-19 years | 1991 | Both | 2257033 | 2047067 | 2507501 |
| 15-19 years | 1992 | Male | 826543.2 | 748632.5 | 915965.2 |
| 15-19 years | 1992 | Female | 1310371 | 1180567 | 1464854 |
| 15-19 years | 1992 | Both | 2136914 | 1940777 | 2367361 |
| 15-19 years | 1993 | Male | 783303.4 | 710260.6 | 863756.5 |
| 15-19 years | 1993 | Female | 1244195 | 1121818 | 1394581 |
| 15-19 years | 1993 | Both | 2027499 | 1836587 | 2252090 |
| 15-19 years | 1994 | Male | 749056.2 | 679805.4 | 826966.7 |
| 15-19 years | 1994 | Female | 1189477 | 1070418 | 1333587 |
| 15-19 years | 1994 | Both | 1938533 | 1757652 | 2155563 |
| 15-19 years | 1995 | Male | 726322.6 | 655847.9 | 805019.3 |
| 15-19 years | 1995 | Female | 1152415 | 1032209 | 1292809 |
| 15-19 years | 1995 | Both | 1878738 | 1701200 | 2091123 |
| 15-19 years | 1996 | Male | 716092.4 | 646516.6 | 793562.3 |
| 15-19 years | 1996 | Female | 1138113 | 1020098 | 1276583 |
| 15-19 years | 1996 | Both | 1854205 | 1679333 | 2063678 |
| 15-19 years | 1997 | Male | 715303.4 | 645724.9 | 792494.5 |
| 15-19 years | 1997 | Female | 1138841 | 1021478 | 1277235 |
| 15-19 years | 1997 | Both | 1854145 | 1679593 | 2063470 |
| 15-19 years | 1998 | Male | 722971.2 | 652620.3 | 800841.3 |
| 15-19 years | 1998 | Female | 1151135 | 1032920 | 1290890 |
| 15-19 years | 1998 | Both | 1874107 | 1697948 | 2085585 |
| 15-19 years | 1999 | Male | 739416.4 | 667538.4 | 818963.3 |
| 15-19 years | 1999 | Female | 1175121 | 1054415 | 1317692 |
| 15-19 years | 1999 | Both | 1914537 | 1734797 | 2130522 |
| 15-19 years | 2000 | Male | 766297.1 | 691838.8 | 848706.1 |
| 15-19 years | 2000 | Female | 1214639 | 1089860 | 1361950 |
| 15-19 years | 2000 | Both | 1980936 | 1795097 | 2204396 |
| 15-19 years | 2001 | Male | 806164.2 | 728416.4 | 887997.7 |
| 15-19 years | 2001 | Female | 1275470 | 1144861 | 1425886 |
| 15-19 years | 2001 | Both | 2081634 | 1888722 | 2311611 |
| 15-19 years | 2002 | Male | 853787.6 | 770500.5 | 941915.6 |
| 15-19 years | 2002 | Female | 1349980 | 1217971 | 1508713 |
| 15-19 years | 2002 | Both | 2203767 | 2000228 | 2443757 |
| 15-19 years | 2003 | Male | 901008.3 | 815369 | 996383.2 |
| 15-19 years | 2003 | Female | 1426012 | 1286896 | 1586629 |
| 15-19 years | 2003 | Both | 2327021 | 2116148 | 2575657 |
| 15-19 years | 2004 | Male | 938141.2 | 846129.7 | 1038039 |
| 15-19 years | 2004 | Female | 1487575 | 1343623 | 1656528 |
| 15-19 years | 2004 | Both | 2425716 | 2206694 | 2678716 |
| 15-19 years | 2005 | Male | 954927 | 857135 | 1059476 |
| 15-19 years | 2005 | Female | 1516844 | 1368017 | 1691146 |
| 15-19 years | 2005 | Both | 2471771 | 2247401 | 2738941 |
| 15-19 years | 2006 | Male | 942839.5 | 849742.8 | 1043126 |
| 15-19 years | 2006 | Female | 1498734 | 1354732 | 1667076 |
| 15-19 years | 2006 | Both | 2441573 | 2220404 | 2700258 |
| 15-19 years | 2007 | Male | 908324 | 819443.4 | 1004190 |
| 15-19 years | 2007 | Female | 1443453 | 1304983 | 1606138 |
| 15-19 years | 2007 | Both | 2351777 | 2134899 | 2597113 |
| 15-19 years | 2008 | Male | 859252.2 | 773998.4 | 954986.8 |
| 15-19 years | 2008 | Female | 1362145 | 1232115 | 1505648 |
| 15-19 years | 2008 | Both | 2221397 | 2006914 | 2449596 |
| 15-19 years | 2009 | Male | 803900.3 | 722897.3 | 893759.7 |
| 15-19 years | 2009 | Female | 1267807 | 1145586 | 1407220 |
| 15-19 years | 2009 | Both | 2071707 | 1866447 | 2292865 |
| 15-19 years | 2010 | Male | 749770 | 670247.6 | 837105.1 |
| 15-19 years | 2010 | Female | 1172612 | 1055274 | 1311179 |
| 15-19 years | 2010 | Both | 1922382 | 1739322 | 2124860 |
| 15-19 years | 2011 | Male | 702843.6 | 628263.7 | 784772.4 |
| 15-19 years | 2011 | Female | 1088615 | 979505.1 | 1216959 |
| 15-19 years | 2011 | Both | 1791458 | 1620923 | 1980221 |
| 15-19 years | 2012 | Male | 663320.5 | 592911.7 | 740681.9 |
| 15-19 years | 2012 | Female | 1017954 | 915820.6 | 1137787 |
| 15-19 years | 2012 | Both | 1681274 | 1521217 | 1858483 |
| 15-19 years | 2013 | Male | 630737.4 | 563776.3 | 704318.1 |
| 15-19 years | 2013 | Female | 959719.8 | 863390.8 | 1072635 |
| 15-19 years | 2013 | Both | 1590457 | 1438970 | 1758129 |
| 15-19 years | 2014 | Male | 604836 | 540625.5 | 675394.6 |
| 15-19 years | 2014 | Female | 913507.5 | 821841.5 | 1021043 |
| 15-19 years | 2014 | Both | 1518343 | 1373590 | 1678423 |
| 15-19 years | 2015 | Male | 585925.4 | 523735.2 | 654256.5 |
| 15-19 years | 2015 | Female | 879779.4 | 791589 | 983505.1 |
| 15-19 years | 2015 | Both | 1465705 | 1325785 | 1620220 |
| 15-19 years | 2016 | Male | 574399 | 513456.2 | 641343.7 |
| 15-19 years | 2016 | Female | 858892.9 | 772950.8 | 960410.5 |
| 15-19 years | 2016 | Both | 1433292 | 1296238 | 1584351 |
| 15-19 years | 2017 | Male | 567661.4 | 507463.1 | 633767.3 |
| 15-19 years | 2017 | Female | 845985.2 | 761437 | 946263.3 |
| 15-19 years | 2017 | Both | 1413647 | 1278232 | 1562579 |
| 15-19 years | 2018 | Male | 567273.6 | 512971.5 | 630080.1 |
| 15-19 years | 2018 | Female | 842000.5 | 759021.7 | 931213.6 |
| 15-19 years | 2018 | Both | 1409274 | 1275931 | 1553698 |
| 15-19 years | 2019 | Male | 572241.2 | 516819.7 | 641662 |
| 15-19 years | 2019 | Female | 844903.2 | 756680.5 | 940109.5 |
| 15-19 years | 2019 | Both | 1417144 | 1281574 | 1568992 |

## Supplementary Table 4. Prevalence rates of Chinese children and adolescents with AD in different age groups from 1990 to 2019

| **Age group** | **Year** | **Sex** | **Value*** | **Lower*** | **Upper*** |
| --- | --- | --- | --- | --- | --- |
| <5 years | 1990 | Male | 2491.54 | 2273.876 | 2732.885 |
| <5 years | 1990 | Female | 2184.303 | 1981.254 | 2404.841 |
| <5 years | 1990 | Both | 2346.534 | 2156.294 | 2557.966 |
| <5 years | 1991 | Male | 2484.965 | 2285.123 | 2714.69 |
| <5 years | 1991 | Female | 2178.154 | 1981.342 | 2375.711 |
| <5 years | 1991 | Both | 2340.404 | 2151.342 | 2543.372 |
| <5 years | 1992 | Male | 2475.753 | 2273.448 | 2695.056 |
| <5 years | 1992 | Female | 2170.119 | 1980.28 | 2362.268 |
| <5 years | 1992 | Both | 2332.041 | 2142.338 | 2532.289 |
| <5 years | 1993 | Male | 2464.05 | 2258.02 | 2682.723 |
| <5 years | 1993 | Female | 2159.638 | 1970.293 | 2356.999 |
| <5 years | 1993 | Both | 2321.288 | 2133.288 | 2523.574 |
| <5 years | 1994 | Male | 2452.396 | 2247.269 | 2665.09 |
| <5 years | 1994 | Female | 2148.969 | 1953.351 | 2354.699 |
| <5 years | 1994 | Both | 2310.534 | 2121.68 | 2515.007 |
| <5 years | 1995 | Male | 2445.168 | 2231.417 | 2672.886 |
| <5 years | 1995 | Female | 2141.823 | 1932.787 | 2350.826 |
| <5 years | 1995 | Both | 2303.819 | 2115.497 | 2511.856 |
| <5 years | 1996 | Male | 2442.835 | 2229.036 | 2670.703 |
| <5 years | 1996 | Female | 2138.645 | 1929.834 | 2347.593 |
| <5 years | 1996 | Both | 2301.541 | 2113.559 | 2509.236 |
| <5 years | 1997 | Male | 2441.81 | 2227.887 | 2669.465 |
| <5 years | 1997 | Female | 2137.069 | 1928.355 | 2346.062 |
| <5 years | 1997 | Both | 2300.664 | 2112.891 | 2508.159 |
| <5 years | 1998 | Male | 2442.246 | 2228.115 | 2669.826 |
| <5 years | 1998 | Female | 2137.575 | 1928.784 | 2346.748 |
| <5 years | 1998 | Both | 2301.502 | 2113.777 | 2508.975 |
| <5 years | 1999 | Male | 2443.861 | 2229.475 | 2671.486 |
| <5 years | 1999 | Female | 2139.38 | 1930.418 | 2348.79 |
| <5 years | 1999 | Both | 2303.539 | 2115.74 | 2511.119 |
| <5 years | 2000 | Male | 2445.837 | 2231.219 | 2673.55 |
| <5 years | 2000 | Female | 2141.527 | 1932.397 | 2351.14 |
| <5 years | 2000 | Both | 2305.889 | 2117.85 | 2513.626 |
| <5 years | 2001 | Male | 2450.727 | 2243.842 | 2672.616 |
| <5 years | 2001 | Female | 2149.282 | 1958.172 | 2353 |
| <5 years | 2001 | Both | 2312.371 | 2123.074 | 2521.303 |
| <5 years | 2002 | Male | 2459.896 | 2255.38 | 2681.447 |
| <5 years | 2002 | Female | 2165.44 | 1979.077 | 2367.414 |
| <5 years | 2002 | Both | 2325.013 | 2133.704 | 2531.848 |
| <5 years | 2003 | Male | 2469.896 | 2266.284 | 2689.223 |
| <5 years | 2003 | Female | 2184.005 | 1996.762 | 2396.288 |
| <5 years | 2003 | Both | 2339.19 | 2148.275 | 2543.825 |
| <5 years | 2004 | Male | 2478.148 | 2269.178 | 2704.261 |
| <5 years | 2004 | Female | 2199.329 | 2000.592 | 2416.606 |
| <5 years | 2004 | Both | 2350.915 | 2158.701 | 2564.592 |
| <5 years | 2005 | Male | 2481.89 | 2266.271 | 2725.141 |
| <5 years | 2005 | Female | 2205.897 | 2000.157 | 2432.395 |
| <5 years | 2005 | Both | 2356.177 | 2158.78 | 2574.831 |
| <5 years | 2006 | Male | 2479.122 | 2276.433 | 2707.838 |
| <5 years | 2006 | Female | 2202.563 | 2003.276 | 2420.039 |
| <5 years | 2006 | Both | 2353.374 | 2163.887 | 2567.383 |
| <5 years | 2007 | Male | 2471.208 | 2271.996 | 2689.85 |
| <5 years | 2007 | Female | 2193.474 | 1997.308 | 2410.673 |
| <5 years | 2007 | Both | 2345.101 | 2165.191 | 2548.338 |
| <5 years | 2008 | Male | 2460.118 | 2268.291 | 2671.924 |
| <5 years | 2008 | Female | 2181.002 | 1996.864 | 2400.726 |
| <5 years | 2008 | Both | 2333.477 | 2153.412 | 2538.207 |
| <5 years | 2009 | Male | 2450.867 | 2252.146 | 2660.959 |
| <5 years | 2009 | Female | 2170.142 | 1987.902 | 2394.816 |
| <5 years | 2009 | Both | 2323.489 | 2144.076 | 2527.209 |
| <5 years | 2010 | Male | 2450.86 | 2240.022 | 2676.336 |
| <5 years | 2010 | Female | 2168.035 | 1979.747 | 2404.581 |
| <5 years | 2010 | Both | 2322.413 | 2138.082 | 2525.009 |
| <5 years | 2011 | Male | 2446.876 | 2236.32 | 2671.92 |
| <5 years | 2011 | Female | 2163.527 | 1975.709 | 2399.376 |
| <5 years | 2011 | Both | 2317.976 | 2134.011 | 2520.233 |
| <5 years | 2012 | Male | 2425.223 | 2216.486 | 2648.226 |
| <5 years | 2012 | Female | 2144.14 | 1958.067 | 2377.713 |
| <5 years | 2012 | Both | 2297.075 | 2114.804 | 2497.598 |
| <5 years | 2013 | Male | 2417.974 | 2209.819 | 2640.277 |
| <5 years | 2013 | Female | 2137.866 | 1952.373 | 2370.666 |
| <5 years | 2013 | Both | 2289.957 | 2108.252 | 2489.995 |
| <5 years | 2014 | Male | 2434.204 | 2224.605 | 2657.976 |
| <5 years | 2014 | Female | 2152.645 | 1965.873 | 2387.038 |
| <5 years | 2014 | Both | 2305.204 | 2122.34 | 2506.748 |
| <5 years | 2015 | Male | 2413.614 | 2205.728 | 2635.46 |
| <5 years | 2015 | Female | 2134.768 | 1949.53 | 2367.223 |
| <5 years | 2015 | Both | 2285.566 | 2104.385 | 2485.562 |
| <5 years | 2016 | Male | 2373.081 | 2168.604 | 2591.272 |
| <5 years | 2016 | Female | 2099.453 | 1917.247 | 2328.071 |
| <5 years | 2016 | Both | 2247.177 | 2069.165 | 2443.975 |
| <5 years | 2017 | Male | 2398.59 | 2191.811 | 2619.268 |
| <5 years | 2017 | Female | 2123.33 | 1939.001 | 2354.569 |
| <5 years | 2017 | Both | 2271.737 | 2091.902 | 2470.843 |
| <5 years | 2018 | Male | 2479.793 | 2283.294 | 2695.932 |
| <5 years | 2018 | Female | 2192.463 | 2010.798 | 2395.243 |
| <5 years | 2018 | Both | 2347.237 | 2168.416 | 2558.911 |
| <5 years | 2019 | Male | 2560.789 | 2339.662 | 2832.033 |
| <5 years | 2019 | Female | 2255.859 | 2054.454 | 2480.129 |
| <5 years | 2019 | Both | 2420.025 | 2218.362 | 2649.873 |
| 5-9 years | 1990 | Male | 2928.069 | 2673.297 | 3213.945 |
| 5-9 years | 1990 | Female | 2938.889 | 2675.476 | 3229.574 |
| 5-9 years | 1990 | Both | 2933.262 | 2690.092 | 3191.242 |
| 5-9 years | 1991 | Male | 2894.47 | 2653.768 | 3159.928 |
| 5-9 years | 1991 | Female | 2904.099 | 2649.751 | 3181.876 |
| 5-9 years | 1991 | Both | 2899.077 | 2670.353 | 3146.505 |
| 5-9 years | 1992 | Male | 2863.315 | 2629.723 | 3108.414 |
| 5-9 years | 1992 | Female | 2871.678 | 2623.022 | 3128.819 |
| 5-9 years | 1992 | Both | 2867.303 | 2643.826 | 3096.518 |
| 5-9 years | 1993 | Male | 2837.305 | 2609.044 | 3081.15 |
| 5-9 years | 1993 | Female | 2844.492 | 2599.488 | 3099.705 |
| 5-9 years | 1993 | Both | 2840.723 | 2619.593 | 3066.433 |
| 5-9 years | 1994 | Male | 2819.109 | 2594.097 | 3070.286 |
| 5-9 years | 1994 | Female | 2825.37 | 2567.464 | 3075.578 |
| 5-9 years | 1994 | Both | 2822.08 | 2596.203 | 3058.306 |
| 5-9 years | 1995 | Male | 2811.342 | 2575.627 | 3077.312 |
| 5-9 years | 1995 | Female | 2817.063 | 2539.503 | 3078.636 |
| 5-9 years | 1995 | Both | 2814.051 | 2584.995 | 3061.579 |
| 5-9 years | 1996 | Male | 2809.418 | 2574.022 | 3075.01 |
| 5-9 years | 1996 | Female | 2814.845 | 2537.686 | 3076.393 |
| 5-9 years | 1996 | Both | 2811.981 | 2583.327 | 3059.024 |
| 5-9 years | 1997 | Male | 2807.125 | 2572.179 | 3072.088 |
| 5-9 years | 1997 | Female | 2812.309 | 2535.322 | 3074.174 |
| 5-9 years | 1997 | Both | 2809.565 | 2581.407 | 3055.864 |
| 5-9 years | 1998 | Male | 2804.555 | 2570.165 | 3068.683 |
| 5-9 years | 1998 | Female | 2809.548 | 2532.797 | 3071.999 |
| 5-9 years | 1998 | Both | 2806.895 | 2579.314 | 3052.236 |
| 5-9 years | 1999 | Male | 2801.912 | 2568.132 | 3065.083 |
| 5-9 years | 1999 | Female | 2806.767 | 2530.293 | 3069.885 |
| 5-9 years | 1999 | Both | 2804.177 | 2576.632 | 3048.446 |
| 5-9 years | 2000 | Male | 2799.517 | 2566.311 | 3061.763 |
| 5-9 years | 2000 | Female | 2804.293 | 2528.085 | 3068.043 |
| 5-9 years | 2000 | Both | 2801.735 | 2573.772 | 3044.989 |
| 5-9 years | 2001 | Male | 2801.301 | 2568.802 | 3043.499 |
| 5-9 years | 2001 | Female | 2808.476 | 2551.431 | 3072.373 |
| 5-9 years | 2001 | Both | 2804.621 | 2576.359 | 3041.404 |
| 5-9 years | 2002 | Male | 2808.64 | 2578.797 | 3040.332 |
| 5-9 years | 2002 | Female | 2821.648 | 2579.124 | 3077.303 |
| 5-9 years | 2002 | Both | 2814.641 | 2587.712 | 3047.34 |
| 5-9 years | 2003 | Male | 2818.294 | 2593.06 | 3041.712 |
| 5-9 years | 2003 | Female | 2838.344 | 2592.687 | 3104.806 |
| 5-9 years | 2003 | Both | 2827.523 | 2601.688 | 3067.293 |
| 5-9 years | 2004 | Male | 2827.036 | 2593.027 | 3065.283 |
| 5-9 years | 2004 | Female | 2853.111 | 2599.754 | 3129.501 |
| 5-9 years | 2004 | Both | 2839.016 | 2605.728 | 3083.384 |
| 5-9 years | 2005 | Male | 2831.695 | 2587.06 | 3085.679 |
| 5-9 years | 2005 | Female | 2860.557 | 2600.152 | 3147.421 |
| 5-9 years | 2005 | Both | 2844.939 | 2608.893 | 3097.254 |
| 5-9 years | 2006 | Male | 2829.047 | 2590.217 | 3070.15 |
| 5-9 years | 2006 | Female | 2857.243 | 2615.329 | 3122.47 |
| 5-9 years | 2006 | Both | 2841.975 | 2613.719 | 3087.177 |
| 5-9 years | 2007 | Male | 2820.571 | 2593.972 | 3060.231 |
| 5-9 years | 2007 | Female | 2846.594 | 2605.661 | 3097.885 |
| 5-9 years | 2007 | Both | 2832.495 | 2605.985 | 3069.815 |
| 5-9 years | 2008 | Male | 2810.262 | 2590.062 | 3057.251 |
| 5-9 years | 2008 | Female | 2833.645 | 2589.857 | 3083.492 |
| 5-9 years | 2008 | Both | 2820.969 | 2596.12 | 3052.202 |
| 5-9 years | 2009 | Male | 2802.121 | 2585.529 | 3061.337 |
| 5-9 years | 2009 | Female | 2823.42 | 2569.578 | 3070.574 |
| 5-9 years | 2009 | Both | 2811.867 | 2584.697 | 3042.755 |
| 5-9 years | 2010 | Male | 2800.107 | 2563.174 | 3073.503 |
| 5-9 years | 2010 | Female | 2820.88 | 2545.143 | 3067.628 |
| 5-9 years | 2010 | Both | 2809.603 | 2582.484 | 3041.747 |
| 5-9 years | 2011 | Male | 2802.131 | 2565.125 | 3075.574 |
| 5-9 years | 2011 | Female | 2823.479 | 2547.533 | 3070.276 |
| 5-9 years | 2011 | Both | 2811.876 | 2584.418 | 3044.13 |
| 5-9 years | 2012 | Male | 2803.728 | 2566.722 | 3077.108 |
| 5-9 years | 2012 | Female | 2825.624 | 2549.591 | 3072.396 |
| 5-9 years | 2012 | Both | 2813.71 | 2585.952 | 3046.052 |
| 5-9 years | 2013 | Male | 2805.058 | 2568.094 | 3078.306 |
| 5-9 years | 2013 | Female | 2827.457 | 2551.404 | 3074.149 |
| 5-9 years | 2013 | Both | 2815.26 | 2587.23 | 3047.702 |
| 5-9 years | 2014 | Male | 2806.201 | 2569.294 | 3079.454 |
| 5-9 years | 2014 | Female | 2829.034 | 2552.991 | 3075.623 |
| 5-9 years | 2014 | Both | 2816.599 | 2588.319 | 3049.167 |
| 5-9 years | 2015 | Male | 2807.183 | 2570.327 | 3080.509 |
| 5-9 years | 2015 | Female | 2830.36 | 2554.331 | 3076.852 |
| 5-9 years | 2015 | Both | 2817.746 | 2589.238 | 3050.47 |
| 5-9 years | 2016 | Male | 2808.022 | 2571.205 | 3081.416 |
| 5-9 years | 2016 | Female | 2831.458 | 2555.438 | 3077.869 |
| 5-9 years | 2016 | Both | 2818.718 | 2590.005 | 3051.561 |
| 5-9 years | 2017 | Male | 2808.608 | 2571.823 | 3082.049 |
| 5-9 years | 2017 | Female | 2832.226 | 2556.235 | 3078.582 |
| 5-9 years | 2017 | Both | 2819.408 | 2590.519 | 3052.322 |
| 5-9 years | 2018 | Male | 2846.636 | 2623.25 | 3108.65 |
| 5-9 years | 2018 | Female | 2867.259 | 2629.318 | 3116.324 |
| 5-9 years | 2018 | Both | 2856.088 | 2630.906 | 3090.512 |
| 5-9 years | 2019 | Male | 2929.76 | 2664.5 | 3216.473 |
| 5-9 years | 2019 | Female | 2943.62 | 2674.095 | 3241.822 |
| 5-9 years | 2019 | Both | 2936.127 | 2698.486 | 3200.83 |
| 10-14 years | 1990 | Male | 1919.157 | 1722.776 | 2100.689 |
| 10-14 years | 1990 | Female | 2344.577 | 2131.227 | 2586.345 |
| 10-14 years | 1990 | Both | 2124.98 | 1933.633 | 2322.231 |
| 10-14 years | 1991 | Male | 1899.28 | 1726.578 | 2069.868 |
| 10-14 years | 1991 | Female | 2322.284 | 2111.705 | 2552.1 |
| 10-14 years | 1991 | Both | 2103.906 | 1924.667 | 2293.628 |
| 10-14 years | 1992 | Male | 1880.917 | 1719.109 | 2049.127 |
| 10-14 years | 1992 | Female | 2301.557 | 2083.189 | 2519.549 |
| 10-14 years | 1992 | Both | 2084.427 | 1905.817 | 2265.014 |
| 10-14 years | 1993 | Male | 1865.655 | 1705.526 | 2032.99 |
| 10-14 years | 1993 | Female | 2284.182 | 2074.948 | 2498.433 |
| 10-14 years | 1993 | Both | 2068.048 | 1892.48 | 2247.713 |
| 10-14 years | 1994 | Male | 1855.176 | 1693.218 | 2027.557 |
| 10-14 years | 1994 | Female | 2272.139 | 2056.355 | 2490.949 |
| 10-14 years | 1994 | Both | 2056.522 | 1873.901 | 2237.461 |
| 10-14 years | 1995 | Male | 1851.21 | 1685.496 | 2036.424 |
| 10-14 years | 1995 | Female | 2267.506 | 2038.724 | 2489.628 |
| 10-14 years | 1995 | Both | 2051.799 | 1865.16 | 2234.052 |
| 10-14 years | 1996 | Male | 1851.092 | 1685.319 | 2036.318 |
| 10-14 years | 1996 | Female | 2267.302 | 2038.45 | 2489.325 |
| 10-14 years | 1996 | Both | 2051.176 | 1864.606 | 2233.487 |
| 10-14 years | 1997 | Male | 1850.872 | 1685.01 | 2036.127 |
| 10-14 years | 1997 | Female | 2267.021 | 2037.999 | 2488.79 |
| 10-14 years | 1997 | Both | 2050.548 | 1864.101 | 2233.039 |
| 10-14 years | 1998 | Male | 1850.494 | 1684.546 | 2035.77 |
| 10-14 years | 1998 | Female | 2266.61 | 2037.414 | 2488.062 |
| 10-14 years | 1998 | Both | 2049.932 | 1863.627 | 2232.63 |
| 10-14 years | 1999 | Male | 1849.902 | 1683.897 | 2035.161 |
| 10-14 years | 1999 | Female | 2266 | 2036.657 | 2487.172 |
| 10-14 years | 1999 | Both | 2049.21 | 1863.041 | 2232.035 |
| 10-14 years | 2000 | Male | 1849.015 | 1683.012 | 2034.187 |
| 10-14 years | 2000 | Female | 2265.077 | 2035.691 | 2486.104 |
| 10-14 years | 2000 | Both | 2048.166 | 1862.1 | 2230.919 |
| 10-14 years | 2001 | Male | 1849.477 | 1689.384 | 2021.362 |
| 10-14 years | 2001 | Female | 2265.308 | 2045.371 | 2473.521 |
| 10-14 years | 2001 | Both | 2048.169 | 1865.393 | 2223.65 |
| 10-14 years | 2002 | Male | 1852.014 | 1694.882 | 2017.362 |
| 10-14 years | 2002 | Female | 2267.408 | 2057.725 | 2470.589 |
| 10-14 years | 2002 | Both | 2049.943 | 1872.933 | 2221.493 |
| 10-14 years | 2003 | Male | 1855.064 | 1699.826 | 2024.722 |
| 10-14 years | 2003 | Female | 2269.955 | 2051.859 | 2474.683 |
| 10-14 years | 2003 | Both | 2052.006 | 1874.708 | 2222.57 |
| 10-14 years | 2004 | Male | 1857.15 | 1694.732 | 2030.695 |
| 10-14 years | 2004 | Female | 2271.602 | 2050.026 | 2483.018 |
| 10-14 years | 2004 | Both | 2053.007 | 1874.351 | 2230.764 |
| 10-14 years | 2005 | Male | 1856.868 | 1688.222 | 2038.528 |
| 10-14 years | 2005 | Female | 2271.059 | 2041.577 | 2499.81 |
| 10-14 years | 2005 | Both | 2051.674 | 1868.78 | 2236.333 |
| 10-14 years | 2006 | Male | 1852.292 | 1690.749 | 2030.514 |
| 10-14 years | 2006 | Female | 2266.699 | 2057.623 | 2476.961 |
| 10-14 years | 2006 | Both | 2046.351 | 1867.821 | 2223.321 |
| 10-14 years | 2007 | Male | 1844.216 | 1684.096 | 2013.65 |
| 10-14 years | 2007 | Female | 2259.37 | 2050.378 | 2458.112 |
| 10-14 years | 2007 | Both | 2037.868 | 1862.148 | 2209.708 |
| 10-14 years | 2008 | Male | 1835.382 | 1675.353 | 1995.678 |
| 10-14 years | 2008 | Female | 2251.536 | 2041.197 | 2448.069 |
| 10-14 years | 2008 | Both | 2028.827 | 1856.469 | 2193.444 |
| 10-14 years | 2009 | Male | 1828.517 | 1662.414 | 1995.432 |
| 10-14 years | 2009 | Female | 2245.651 | 2029.179 | 2452.674 |
| 10-14 years | 2009 | Both | 2021.851 | 1845.354 | 2187.409 |
| 10-14 years | 2010 | Male | 1826.385 | 1657.226 | 2007.991 |
| 10-14 years | 2010 | Female | 2244.218 | 2028.766 | 2465.881 |
| 10-14 years | 2010 | Both | 2019.623 | 1838.139 | 2195.694 |
| 10-14 years | 2011 | Male | 1827.909 | 1658.755 | 2009.809 |
| 10-14 years | 2011 | Female | 2246.302 | 2030.355 | 2468.19 |
| 10-14 years | 2011 | Both | 2021.152 | 1839.171 | 2197.623 |
| 10-14 years | 2012 | Male | 1829.875 | 1660.727 | 2012.16 |
| 10-14 years | 2012 | Female | 2248.941 | 2032.408 | 2471.11 |
| 10-14 years | 2012 | Both | 2023.245 | 1841.009 | 2200.216 |
| 10-14 years | 2013 | Male | 1832.039 | 1662.587 | 2014.657 |
| 10-14 years | 2013 | Female | 2251.831 | 2035.427 | 2474.301 |
| 10-14 years | 2013 | Both | 2025.574 | 1843.248 | 2203.047 |
| 10-14 years | 2014 | Male | 1834.201 | 1664.362 | 2016.998 |
| 10-14 years | 2014 | Female | 2254.73 | 2038.414 | 2477.495 |
| 10-14 years | 2014 | Both | 2027.875 | 1845.039 | 2205.768 |
| 10-14 years | 2015 | Male | 1836.156 | 1666.015 | 2019.056 |
| 10-14 years | 2015 | Female | 2257.393 | 2041.101 | 2480.418 |
| 10-14 years | 2015 | Both | 2029.895 | 1846.707 | 2207.96 |
| 10-14 years | 2016 | Male | 1837.699 | 1667.385 | 2020.615 |
| 10-14 years | 2016 | Female | 2259.568 | 2043.228 | 2482.786 |
| 10-14 years | 2016 | Both | 2031.393 | 1848.068 | 2209.528 |
| 10-14 years | 2017 | Male | 1838.918 | 1668.519 | 2021.795 |
| 10-14 years | 2017 | Female | 2261.341 | 2044.906 | 2484.52 |
| 10-14 years | 2017 | Both | 2032.544 | 1849.215 | 2210.687 |
| 10-14 years | 2018 | Male | 1865.041 | 1696.836 | 2029.28 |
| 10-14 years | 2018 | Female | 2291.921 | 2088.744 | 2501.201 |
| 10-14 years | 2018 | Both | 2060.479 | 1889.367 | 2237.486 |
| 10-14 years | 2019 | Male | 1921.152 | 1741.386 | 2102.886 |
| 10-14 years | 2019 | Female | 2357.213 | 2126.49 | 2591.174 |
| 10-14 years | 2019 | Both | 2120.703 | 1933.84 | 2316.407 |
| 15-19 years | 1990 | Male | 1418.902 | 1271.591 | 1584.961 |
| 15-19 years | 1990 | Female | 2350.404 | 2106.551 | 2629.18 |
| 15-19 years | 1990 | Both | 1872.034 | 1692.022 | 2083.692 |
| 15-19 years | 1991 | Male | 1409.777 | 1271.514 | 1570.082 |
| 15-19 years | 1991 | Female | 2341.244 | 2103.86 | 2616.229 |
| 15-19 years | 1991 | Both | 1863.692 | 1690.318 | 2070.51 |
| 15-19 years | 1992 | Male | 1401.531 | 1269.421 | 1553.16 |
| 15-19 years | 1992 | Female | 2333.018 | 2101.913 | 2608.065 |
| 15-19 years | 1992 | Both | 1855.916 | 1685.571 | 2056.061 |
| 15-19 years | 1993 | Male | 1394.977 | 1264.896 | 1538.255 |
| 15-19 years | 1993 | Female | 2326.574 | 2097.735 | 2607.788 |
| 15-19 years | 1993 | Both | 1849.414 | 1675.27 | 2054.278 |
| 15-19 years | 1994 | Male | 1390.82 | 1262.238 | 1535.482 |
| 15-19 years | 1994 | Female | 2322.567 | 2090.093 | 2603.955 |
| 15-19 years | 1994 | Both | 1844.974 | 1672.823 | 2051.529 |
| 15-19 years | 1995 | Male | 1389.729 | 1254.884 | 1540.305 |
| 15-19 years | 1995 | Female | 2321.679 | 2079.509 | 2604.519 |
| 15-19 years | 1995 | Both | 1843.694 | 1669.467 | 2052.117 |
| 15-19 years | 1996 | Male | 1390.373 | 1255.284 | 1540.789 |
| 15-19 years | 1996 | Female | 2322.612 | 2081.771 | 2605.196 |
| 15-19 years | 1996 | Both | 1844.888 | 1670.894 | 2053.307 |
| 15-19 years | 1997 | Male | 1391 | 1255.695 | 1541.108 |
| 15-19 years | 1997 | Female | 2323.512 | 2084.063 | 2605.869 |
| 15-19 years | 1997 | Both | 1846.068 | 1672.277 | 2054.482 |
| 15-19 years | 1998 | Male | 1391.515 | 1256.109 | 1541.392 |
| 15-19 years | 1998 | Female | 2324.249 | 2085.561 | 2606.426 |
| 15-19 years | 1998 | Both | 1846.722 | 1673.137 | 2055.11 |
| 15-19 years | 1999 | Male | 1391.916 | 1256.609 | 1541.659 |
| 15-19 years | 1999 | Female | 2324.937 | 2086.126 | 2607.01 |
| 15-19 years | 1999 | Both | 1846.825 | 1673.442 | 2055.172 |
| 15-19 years | 2000 | Male | 1392.256 | 1256.976 | 1541.982 |
| 15-19 years | 2000 | Female | 2325.787 | 2086.861 | 2607.859 |
| 15-19 years | 2000 | Both | 1846.772 | 1673.519 | 2055.097 |
| 15-19 years | 2001 | Male | 1393.082 | 1258.731 | 1534.493 |
| 15-19 years | 2001 | Female | 2327.105 | 2088.808 | 2601.541 |
| 15-19 years | 2001 | Both | 1847.411 | 1676.205 | 2051.512 |
| 15-19 years | 2002 | Male | 1394.494 | 1258.46 | 1538.433 |
| 15-19 years | 2002 | Female | 2328.731 | 2101.015 | 2602.549 |
| 15-19 years | 2002 | Both | 1848.856 | 1678.097 | 2050.196 |
| 15-19 years | 2003 | Male | 1396.047 | 1263.355 | 1543.823 |
| 15-19 years | 2003 | Female | 2330.509 | 2103.154 | 2593.003 |
| 15-19 years | 2003 | Both | 1850.824 | 1683.104 | 2048.581 |
| 15-19 years | 2004 | Male | 1397.315 | 1260.269 | 1546.107 |
| 15-19 years | 2004 | Female | 2332.357 | 2106.656 | 2597.257 |
| 15-19 years | 2004 | Both | 1852.841 | 1685.545 | 2046.09 |
| 15-19 years | 2005 | Male | 1397.857 | 1254.705 | 1550.899 |
| 15-19 years | 2005 | Female | 2334.192 | 2105.169 | 2602.416 |
| 15-19 years | 2005 | Both | 1854.329 | 1686.006 | 2054.76 |
| 15-19 years | 2006 | Male | 1397.871 | 1259.844 | 1546.557 |
| 15-19 years | 2006 | Female | 2338.71 | 2114.002 | 2601.401 |
| 15-19 years | 2006 | Both | 1856.257 | 1688.109 | 2052.928 |
| 15-19 years | 2007 | Male | 1397.765 | 1260.992 | 1545.287 |
| 15-19 years | 2007 | Female | 2346.783 | 2121.656 | 2611.278 |
| 15-19 years | 2007 | Both | 1859.233 | 1687.777 | 2053.187 |
| 15-19 years | 2008 | Male | 1397.321 | 1258.681 | 1553.005 |
| 15-19 years | 2008 | Female | 2355.367 | 2130.524 | 2603.508 |
| 15-19 years | 2008 | Both | 1861.646 | 1681.898 | 2052.889 |
| 15-19 years | 2009 | Male | 1396.391 | 1255.687 | 1552.478 |
| 15-19 years | 2009 | Female | 2361.526 | 2133.866 | 2621.208 |
| 15-19 years | 2009 | Both | 1862.112 | 1677.618 | 2060.895 |
| 15-19 years | 2010 | Male | 1394.887 | 1246.942 | 1557.367 |
| 15-19 years | 2010 | Female | 2362.426 | 2126.029 | 2641.593 |
| 15-19 years | 2010 | Both | 1859.4 | 1682.337 | 2055.244 |
| 15-19 years | 2011 | Male | 1393.274 | 1245.431 | 1555.684 |
| 15-19 years | 2011 | Female | 2360.047 | 2123.504 | 2638.29 |
| 15-19 years | 2011 | Both | 1855.044 | 1678.455 | 2050.506 |
| 15-19 years | 2012 | Male | 1392.127 | 1244.358 | 1554.487 |
| 15-19 years | 2012 | Female | 2358.364 | 2121.745 | 2635.99 |
| 15-19 years | 2012 | Both | 1851.388 | 1675.136 | 2046.527 |
| 15-19 years | 2013 | Male | 1391.532 | 1243.803 | 1553.866 |
| 15-19 years | 2013 | Female | 2357.543 | 2120.912 | 2634.918 |
| 15-19 years | 2013 | Both | 1848.61 | 1672.534 | 2043.497 |
| 15-19 years | 2014 | Male | 1391.551 | 1243.821 | 1553.885 |
| 15-19 years | 2014 | Female | 2357.706 | 2121.121 | 2635.247 |
| 15-19 years | 2014 | Both | 1846.897 | 1670.821 | 2041.616 |
| 15-19 years | 2015 | Male | 1392.25 | 1244.476 | 1554.615 |
| 15-19 years | 2015 | Female | 2358.979 | 2122.511 | 2637.101 |
| 15-19 years | 2015 | Both | 1846.448 | 1670.181 | 2041.101 |
| 15-19 years | 2016 | Male | 1393.642 | 1245.779 | 1556.067 |
| 15-19 years | 2016 | Female | 2361.392 | 2125.107 | 2640.498 |
| 15-19 years | 2016 | Both | 1847.311 | 1670.668 | 2042.006 |
| 15-19 years | 2017 | Male | 1395.453 | 1247.47 | 1557.957 |
| 15-19 years | 2017 | Female | 2364.491 | 2128.183 | 2644.764 |
| 15-19 years | 2017 | Both | 1848.916 | 1671.806 | 2043.705 |
| 15-19 years | 2018 | Male | 1406.145 | 1271.542 | 1561.829 |
| 15-19 years | 2018 | Female | 2380.387 | 2145.801 | 2632.598 |
| 15-19 years | 2018 | Both | 1861.291 | 1685.178 | 2052.038 |
| 15-19 years | 2019 | Male | 1427.187 | 1288.964 | 1600.325 |
| 15-19 years | 2019 | Female | 2411.221 | 2159.447 | 2682.925 |
| 15-19 years | 2019 | Both | 1886.1 | 1705.668 | 2088.197 |

*Per 100,000

## Supplementary Table 5. DALY cases of Chinese children and adolescents with AD in different age groups from 1990 to 2019

| **Age group** | **Year** | **Sex** | **Value** | **Lower** | **Upper** |
| --- | --- | --- | --- | --- | --- |
| <5 years | 1990 | Male | 68239.71 | 37028.3 | 115306.5 |
| <5 years | 1990 | Female | 53534.19 | 28623.02 | 90876.51 |
| <5 years | 1990 | Both | 121773.9 | 65244.02 | 205664.2 |
| <5 years | 1991 | Male | 68234.88 | 36224.34 | 114774.4 |
| <5 years | 1991 | Female | 53332.84 | 28493.17 | 90495.65 |
| <5 years | 1991 | Both | 121567.7 | 65025.76 | 203882.6 |
| <5 years | 1992 | Male | 67280.04 | 35739.02 | 113162.9 |
| <5 years | 1992 | Female | 52357.76 | 27997.85 | 88960.25 |
| <5 years | 1992 | Both | 119637.8 | 63687.47 | 201768.6 |
| <5 years | 1993 | Male | 65480.68 | 34656.52 | 110755.5 |
| <5 years | 1993 | Female | 50715.42 | 26969.81 | 85429.39 |
| <5 years | 1993 | Both | 116196.1 | 62012.45 | 196506.7 |
| <5 years | 1994 | Male | 63033.18 | 33391.88 | 107051.3 |
| <5 years | 1994 | Female | 48509.43 | 26058.89 | 82290.95 |
| <5 years | 1994 | Both | 111542.6 | 59802.15 | 188726.8 |
| <5 years | 1995 | Male | 60447.88 | 32012.65 | 102882.5 |
| <5 years | 1995 | Female | 46258.14 | 24535.46 | 79006.96 |
| <5 years | 1995 | Both | 106706 | 56997.95 | 181928.5 |
| <5 years | 1996 | Male | 58110.2 | 30753.64 | 99018.46 |
| <5 years | 1996 | Female | 44092.58 | 23594.16 | 75127.11 |
| <5 years | 1996 | Both | 102202.8 | 54476.85 | 175227.8 |
| <5 years | 1997 | Male | 55834.72 | 29555.82 | 95817.23 |
| <5 years | 1997 | Female | 42170.76 | 22898.05 | 71572.69 |
| <5 years | 1997 | Both | 98005.48 | 52203.34 | 167390.9 |
| <5 years | 1998 | Male | 53757.6 | 28772.2 | 90739.45 |
| <5 years | 1998 | Female | 40415.25 | 21623.27 | 68788.15 |
| <5 years | 1998 | Both | 94172.86 | 50285.38 | 159195.4 |
| <5 years | 1999 | Male | 51798.01 | 27557.53 | 87834.41 |
| <5 years | 1999 | Female | 38832.86 | 20474.61 | 65946.02 |
| <5 years | 1999 | Both | 90630.87 | 48406.73 | 154385.4 |
| <5 years | 2000 | Male | 50107.31 | 26312.37 | 86020.38 |
| <5 years | 2000 | Female | 37383.67 | 19938.48 | 63243.62 |
| <5 years | 2000 | Both | 87490.98 | 46627.13 | 149863.6 |
| <5 years | 2001 | Male | 48576.64 | 26079.6 | 83648.04 |
| <5 years | 2001 | Female | 36180.93 | 19230.44 | 60862.57 |
| <5 years | 2001 | Both | 84757.57 | 45353.04 | 144344.5 |
| <5 years | 2002 | Male | 47358.94 | 25184.03 | 80936.56 |
| <5 years | 2002 | Female | 35272.88 | 18944.83 | 60265.59 |
| <5 years | 2002 | Both | 82631.82 | 44016.88 | 142121 |
| <5 years | 2003 | Male | 46373.17 | 24409.25 | 79081.79 |
| <5 years | 2003 | Female | 34564.11 | 18617 | 59344.57 |
| <5 years | 2003 | Both | 80937.28 | 42962.81 | 139139 |
| <5 years | 2004 | Male | 45513.53 | 24217.01 | 77299.4 |
| <5 years | 2004 | Female | 33896.86 | 18105.11 | 58083.65 |
| <5 years | 2004 | Both | 79410.39 | 42249.89 | 136732.8 |
| <5 years | 2005 | Male | 44779.07 | 23489.54 | 76692.93 |
| <5 years | 2005 | Female | 33381.66 | 17756.32 | 57286.67 |
| <5 years | 2005 | Both | 78160.73 | 40920.36 | 133481.2 |
| <5 years | 2006 | Male | 44050.46 | 23337.55 | 74717.33 |
| <5 years | 2006 | Female | 32692.64 | 17343.67 | 56525.33 |
| <5 years | 2006 | Both | 76743.1 | 40819.5 | 131035.3 |
| <5 years | 2007 | Male | 43353.45 | 23183.69 | 73246.67 |
| <5 years | 2007 | Female | 32015.67 | 17178.07 | 55075.83 |
| <5 years | 2007 | Both | 75369.13 | 40350.1 | 127358.6 |
| <5 years | 2008 | Male | 42734.16 | 22770.9 | 72066.2 |
| <5 years | 2008 | Female | 31497.58 | 16707.01 | 53895.88 |
| <5 years | 2008 | Both | 74231.74 | 39385.53 | 128302.9 |
| <5 years | 2009 | Male | 42251.36 | 22439.33 | 70967.67 |
| <5 years | 2009 | Female | 31125.45 | 16595.08 | 53310.53 |
| <5 years | 2009 | Both | 73376.8 | 39194.14 | 124601.2 |
| <5 years | 2010 | Male | 42006.16 | 22461.96 | 71391.7 |
| <5 years | 2010 | Female | 30996.89 | 16286.45 | 52874 |
| <5 years | 2010 | Both | 73003.04 | 39029.02 | 124926.4 |
| <5 years | 2011 | Male | 41831.22 | 22234.58 | 71102.49 |
| <5 years | 2011 | Female | 30931.72 | 16269.07 | 53150.6 |
| <5 years | 2011 | Both | 72762.94 | 38956.78 | 124492.9 |
| <5 years | 2012 | Male | 41857.48 | 22128.53 | 72329.54 |
| <5 years | 2012 | Female | 31007.59 | 16438.9 | 53234.73 |
| <5 years | 2012 | Both | 72865.07 | 38888.74 | 124589.9 |
| <5 years | 2013 | Male | 42273.12 | 22171.69 | 72133.03 |
| <5 years | 2013 | Female | 31472.62 | 16483.8 | 54285.18 |
| <5 years | 2013 | Both | 73745.74 | 39370.84 | 125624.3 |
| <5 years | 2014 | Male | 42973.79 | 22590.8 | 72325.28 |
| <5 years | 2014 | Female | 32161.93 | 16987.43 | 55191 |
| <5 years | 2014 | Both | 75135.72 | 40039.6 | 128055.3 |
| <5 years | 2015 | Male | 43644.08 | 23515.6 | 74658.46 |
| <5 years | 2015 | Female | 32773.88 | 17265.82 | 56552.12 |
| <5 years | 2015 | Both | 76417.96 | 40871.29 | 131685.1 |
| <5 years | 2016 | Male | 44786.68 | 23863.47 | 77157.71 |
| <5 years | 2016 | Female | 33816.97 | 17863.63 | 58244.86 |
| <5 years | 2016 | Both | 78603.65 | 41979.71 | 135813.6 |
| <5 years | 2017 | Male | 46785.28 | 24927.65 | 80120.42 |
| <5 years | 2017 | Female | 35436.34 | 18890.19 | 60371.75 |
| <5 years | 2017 | Both | 82221.62 | 44356.71 | 140328.4 |
| <5 years | 2018 | Male | 48908.16 | 26310.47 | 83277.39 |
| <5 years | 2018 | Female | 37068.9 | 19813.78 | 63712.71 |
| <5 years | 2018 | Both | 85977.06 | 45912.67 | 146533.6 |
| <5 years | 2019 | Male | 50645.4 | 26889.58 | 86344.88 |
| <5 years | 2019 | Female | 38346.39 | 20258.56 | 65444.7 |
| <5 years | 2019 | Both | 88991.79 | 47353.77 | 149608.1 |
| 5-9 years | 1990 | Male | 71657.1 | 37748.44 | 121898 |
| 5-9 years | 1990 | Female | 66332.97 | 35576.02 | 112331.9 |
| 5-9 years | 1990 | Both | 137990.1 | 73536.23 | 234421 |
| 5-9 years | 1991 | Male | 72552.27 | 38278.84 | 122465.9 |
| 5-9 years | 1991 | Female | 66719.87 | 35571.65 | 112704.4 |
| 5-9 years | 1991 | Both | 139272.1 | 74107.55 | 233356.7 |
| 5-9 years | 1992 | Male | 73803.77 | 40092.77 | 124238.9 |
| 5-9 years | 1992 | Female | 67597.23 | 36076.32 | 114042.8 |
| 5-9 years | 1992 | Both | 141401 | 75435.14 | 237927.6 |
| 5-9 years | 1993 | Male | 75320.76 | 40538.15 | 127349.2 |
| 5-9 years | 1993 | Female | 68663.93 | 36596.19 | 115531.6 |
| 5-9 years | 1993 | Both | 143984.7 | 76744.07 | 243346.7 |
| 5-9 years | 1994 | Male | 76855.39 | 41438.57 | 129367.7 |
| 5-9 years | 1994 | Female | 69579.39 | 37519.92 | 116050.9 |
| 5-9 years | 1994 | Both | 146434.8 | 78645.82 | 246032.2 |
| 5-9 years | 1995 | Male | 77624.06 | 41642.7 | 130826.2 |
| 5-9 years | 1995 | Female | 69978.61 | 37194.73 | 117815 |
| 5-9 years | 1995 | Both | 147602.7 | 78996.15 | 248046.4 |
| 5-9 years | 1996 | Male | 76773.24 | 40970.39 | 128429.6 |
| 5-9 years | 1996 | Female | 68890.12 | 36968.18 | 115363.1 |
| 5-9 years | 1996 | Both | 145663.4 | 77383.75 | 245972.4 |
| 5-9 years | 1997 | Male | 74556.1 | 40167.36 | 125621.9 |
| 5-9 years | 1997 | Female | 66405.91 | 35455.49 | 111348.4 |
| 5-9 years | 1997 | Both | 140962 | 75503.24 | 235920.6 |
| 5-9 years | 1998 | Male | 71223.25 | 38238.02 | 119799.8 |
| 5-9 years | 1998 | Female | 63021.55 | 33313.58 | 105941.4 |
| 5-9 years | 1998 | Both | 134244.8 | 71124.52 | 226311.9 |
| 5-9 years | 1999 | Male | 67412.26 | 35986.45 | 113587 |
| 5-9 years | 1999 | Female | 59128.87 | 31398.06 | 99874.21 |
| 5-9 years | 1999 | Both | 126541.1 | 67050.61 | 211866.8 |
| 5-9 years | 2000 | Male | 63637.81 | 34519.45 | 107137.9 |
| 5-9 years | 2000 | Female | 55343.67 | 29323.91 | 93457.67 |
| 5-9 years | 2000 | Both | 118981.5 | 63586.41 | 200583.4 |
| 5-9 years | 2001 | Male | 60728.79 | 32559.07 | 101740.4 |
| 5-9 years | 2001 | Female | 52447.52 | 27719.7 | 89221.28 |
| 5-9 years | 2001 | Both | 113176.3 | 60371.43 | 190160.3 |
| 5-9 years | 2002 | Male | 58355.69 | 31701.87 | 98206.9 |
| 5-9 years | 2002 | Female | 50324.81 | 27266.78 | 85320.03 |
| 5-9 years | 2002 | Both | 108680.5 | 58659.41 | 183405.1 |
| 5-9 years | 2003 | Male | 56548.41 | 30452.68 | 95153.07 |
| 5-9 years | 2003 | Female | 48580.25 | 26209.66 | 82241.35 |
| 5-9 years | 2003 | Both | 105128.7 | 57176.01 | 176041.6 |
| 5-9 years | 2004 | Male | 54933.4 | 29590.71 | 92631.61 |
| 5-9 years | 2004 | Female | 47220.63 | 25256.25 | 79202.93 |
| 5-9 years | 2004 | Both | 102154 | 55462.03 | 171808.9 |
| 5-9 years | 2005 | Male | 53603.57 | 28613.02 | 90814.66 |
| 5-9 years | 2005 | Female | 45997.66 | 24608.03 | 77614.69 |
| 5-9 years | 2005 | Both | 99601.23 | 53621.39 | 168387.8 |
| 5-9 years | 2006 | Male | 52538 | 28023.49 | 89027.13 |
| 5-9 years | 2006 | Female | 45001.86 | 24271.49 | 76189.66 |
| 5-9 years | 2006 | Both | 97539.86 | 52665.03 | 164324.6 |
| 5-9 years | 2007 | Male | 51665.34 | 28072.39 | 86775.58 |
| 5-9 years | 2007 | Female | 44155.2 | 23904.45 | 73954.04 |
| 5-9 years | 2007 | Both | 95820.54 | 51425.89 | 161064.7 |
| 5-9 years | 2008 | Male | 51053.9 | 27595 | 86625.13 |
| 5-9 years | 2008 | Female | 43498.65 | 23478.49 | 73602.55 |
| 5-9 years | 2008 | Both | 94552.55 | 51394.56 | 159102.2 |
| 5-9 years | 2009 | Male | 50500.52 | 27023.13 | 84590.24 |
| 5-9 years | 2009 | Female | 42996.74 | 23121 | 72978.2 |
| 5-9 years | 2009 | Both | 93497.26 | 50262.64 | 157170.2 |
| 5-9 years | 2010 | Male | 50148.86 | 26904.47 | 84179.1 |
| 5-9 years | 2010 | Female | 42603.64 | 22815.04 | 72765.91 |
| 5-9 years | 2010 | Both | 92752.49 | 50218.16 | 157067.8 |
| 5-9 years | 2011 | Male | 49723.99 | 26838.16 | 84085.44 |
| 5-9 years | 2011 | Female | 42129.6 | 22736.74 | 71911.93 |
| 5-9 years | 2011 | Both | 91853.59 | 49528.24 | 154898.8 |
| 5-9 years | 2012 | Male | 49250.29 | 26502.97 | 83558.48 |
| 5-9 years | 2012 | Female | 41651.41 | 22370.79 | 70150.36 |
| 5-9 years | 2012 | Both | 90901.7 | 48959.54 | 153020.1 |
| 5-9 years | 2013 | Male | 48826.03 | 26280.64 | 82534.41 |
| 5-9 years | 2013 | Female | 41261.33 | 22431.02 | 69709.74 |
| 5-9 years | 2013 | Both | 90087.36 | 48877.34 | 152348.2 |
| 5-9 years | 2014 | Male | 48505.46 | 25991.05 | 81878.48 |
| 5-9 years | 2014 | Female | 40952.6 | 22222.37 | 69506.64 |
| 5-9 years | 2014 | Both | 89458.06 | 47937.31 | 151607.3 |
| 5-9 years | 2015 | Male | 48182.56 | 26008.01 | 81294.59 |
| 5-9 years | 2015 | Female | 40726.51 | 22109.31 | 69835.56 |
| 5-9 years | 2015 | Both | 88909.08 | 48169.35 | 151026.2 |
| 5-9 years | 2016 | Male | 48067.32 | 26048.18 | 80300.31 |
| 5-9 years | 2016 | Female | 40778.62 | 22304.3 | 69204.89 |
| 5-9 years | 2016 | Both | 88845.94 | 47723.66 | 150051.5 |
| 5-9 years | 2017 | Male | 48469.51 | 26447.29 | 81839.35 |
| 5-9 years | 2017 | Female | 41250.65 | 22179.52 | 70579.14 |
| 5-9 years | 2017 | Both | 89720.17 | 48718.22 | 152060.1 |
| 5-9 years | 2018 | Male | 49810.37 | 27091.29 | 83246.29 |
| 5-9 years | 2018 | Female | 42513.6 | 22822.14 | 72344.16 |
| 5-9 years | 2018 | Both | 92323.97 | 50396.18 | 155496.2 |
| 5-9 years | 2019 | Male | 51807.66 | 27927.9 | 86657.06 |
| 5-9 years | 2019 | Female | 44283.72 | 23621.77 | 75028.71 |
| 5-9 years | 2019 | Both | 96091.38 | 51849.74 | 161072.5 |
| 10-14 years | 1990 | Male | 45566.49 | 24085.34 | 77184.12 |
| 10-14 years | 1990 | Female | 52089.05 | 27781.17 | 88502.82 |
| 10-14 years | 1990 | Both | 97655.54 | 52009.6 | 166833.4 |
| 10-14 years | 1991 | Male | 44483.48 | 23579.57 | 76032.98 |
| 10-14 years | 1991 | Female | 50882.12 | 27071.71 | 87028.56 |
| 10-14 years | 1991 | Both | 95365.6 | 50786.19 | 163081.6 |
| 10-14 years | 1992 | Male | 44047.35 | 23436.39 | 74707.7 |
| 10-14 years | 1992 | Female | 50459.36 | 27033.14 | 85104.46 |
| 10-14 years | 1992 | Both | 94506.71 | 50405.92 | 161057.6 |
| 10-14 years | 1993 | Male | 44110.62 | 23850.52 | 75423.07 |
| 10-14 years | 1993 | Female | 50548.7 | 26915.11 | 86127.22 |
| 10-14 years | 1993 | Both | 94659.32 | 50344.76 | 162629.5 |
| 10-14 years | 1994 | Male | 44806.27 | 23741 | 75935.12 |
| 10-14 years | 1994 | Female | 51230.04 | 27263.53 | 86115.57 |
| 10-14 years | 1994 | Both | 96036.31 | 51180.07 | 160823.5 |
| 10-14 years | 1995 | Male | 46174.97 | 24835.47 | 77606.69 |
| 10-14 years | 1995 | Female | 52487.04 | 28100.5 | 88020.37 |
| 10-14 years | 1995 | Both | 98662.01 | 53191.21 | 165115.8 |
| 10-14 years | 1996 | Male | 48146.61 | 25906.11 | 81646.69 |
| 10-14 years | 1996 | Female | 54494.62 | 29355.41 | 91791.67 |
| 10-14 years | 1996 | Both | 102641.2 | 55281.81 | 171876.9 |
| 10-14 years | 1997 | Male | 50559.03 | 27231.04 | 87156.41 |
| 10-14 years | 1997 | Female | 57108.7 | 30818.02 | 96275.15 |
| 10-14 years | 1997 | Both | 107667.7 | 58004.72 | 183152.7 |
| 10-14 years | 1998 | Male | 53011.69 | 28325.3 | 89837.72 |
| 10-14 years | 1998 | Female | 59690.53 | 32165.86 | 100193.8 |
| 10-14 years | 1998 | Both | 112702.2 | 60876.74 | 190511.4 |
| 10-14 years | 1999 | Male | 54959.32 | 29912.56 | 93271.15 |
| 10-14 years | 1999 | Female | 61857.24 | 33286.65 | 104135.6 |
| 10-14 years | 1999 | Both | 116816.6 | 63661.61 | 196791.1 |
| 10-14 years | 2000 | Male | 55992.42 | 30180.5 | 94882.95 |
| 10-14 years | 2000 | Female | 62854.43 | 33867.82 | 106141.9 |
| 10-14 years | 2000 | Both | 118846.8 | 64767 | 198419.9 |
| 10-14 years | 2001 | Male | 55230.38 | 29634.64 | 94199.07 |
| 10-14 years | 2001 | Female | 61816.15 | 32986.34 | 103495.1 |
| 10-14 years | 2001 | Both | 117046.5 | 62610 | 197174 |
| 10-14 years | 2002 | Male | 53193.92 | 28468.08 | 90834.04 |
| 10-14 years | 2002 | Female | 59256.56 | 31468.88 | 100772 |
| 10-14 years | 2002 | Both | 112450.5 | 60753.81 | 190534.7 |
| 10-14 years | 2003 | Male | 50483.1 | 26938.92 | 85453.68 |
| 10-14 years | 2003 | Female | 55676.48 | 30175.65 | 94084.36 |
| 10-14 years | 2003 | Both | 106159.6 | 57150.35 | 178756.8 |
| 10-14 years | 2004 | Male | 47206.29 | 25230.16 | 79671.44 |
| 10-14 years | 2004 | Female | 51730.4 | 27819.19 | 88293.1 |
| 10-14 years | 2004 | Both | 98936.7 | 53155.14 | 166773.7 |
| 10-14 years | 2005 | Male | 44176.81 | 24197.46 | 74099.78 |
| 10-14 years | 2005 | Female | 47934.86 | 25670.67 | 80659.22 |
| 10-14 years | 2005 | Both | 92111.67 | 49814.19 | 155135.9 |
| 10-14 years | 2006 | Male | 41469.57 | 22184.18 | 70105.02 |
| 10-14 years | 2006 | Female | 44588.06 | 24177.42 | 75689.02 |
| 10-14 years | 2006 | Both | 86057.63 | 46772.29 | 145398.4 |
| 10-14 years | 2007 | Male | 39078.63 | 20868.02 | 65400.87 |
| 10-14 years | 2007 | Female | 41795.8 | 22306.87 | 70826.46 |
| 10-14 years | 2007 | Both | 80874.42 | 43710.73 | 136226.8 |
| 10-14 years | 2008 | Male | 37047.04 | 20000.46 | 62613.86 |
| 10-14 years | 2008 | Female | 39409.04 | 21169.13 | 67291.07 |
| 10-14 years | 2008 | Both | 76456.08 | 41248.62 | 128372.3 |
| 10-14 years | 2009 | Male | 35463.66 | 19130.31 | 60154.86 |
| 10-14 years | 2009 | Female | 37604.92 | 20192.45 | 63986.28 |
| 10-14 years | 2009 | Both | 73068.57 | 40172.63 | 123940.6 |
| 10-14 years | 2010 | Male | 34319.1 | 18405.64 | 57662.57 |
| 10-14 years | 2010 | Female | 36241.37 | 19414.18 | 61372.14 |
| 10-14 years | 2010 | Both | 70560.47 | 38058.94 | 118666.3 |
| 10-14 years | 2011 | Male | 33605.47 | 18023.92 | 56554.25 |
| 10-14 years | 2011 | Female | 35457.41 | 19074.29 | 59961.31 |
| 10-14 years | 2011 | Both | 69062.89 | 37158.31 | 116072.4 |
| 10-14 years | 2012 | Male | 33272.02 | 18086.55 | 56234.37 |
| 10-14 years | 2012 | Female | 34965.89 | 18915.26 | 59205.61 |
| 10-14 years | 2012 | Both | 68237.91 | 37550.11 | 115451.2 |
| 10-14 years | 2013 | Male | 33019.93 | 17606.18 | 55314.73 |
| 10-14 years | 2013 | Female | 34666.26 | 18628.45 | 58488 |
| 10-14 years | 2013 | Both | 67686.19 | 36198.25 | 113739.2 |
| 10-14 years | 2014 | Male | 32894.91 | 17677.59 | 55085.31 |
| 10-14 years | 2014 | Female | 34417.39 | 18534.64 | 58470.4 |
| 10-14 years | 2014 | Both | 67312.3 | 36548.32 | 113435 |
| 10-14 years | 2015 | Male | 32691.31 | 17334.87 | 54937.42 |
| 10-14 years | 2015 | Female | 34139.92 | 18443.16 | 57329.82 |
| 10-14 years | 2015 | Both | 66831.23 | 36249.53 | 112753.9 |
| 10-14 years | 2016 | Male | 32396.52 | 16982.2 | 54792.89 |
| 10-14 years | 2016 | Female | 33761.34 | 18029.75 | 57082.73 |
| 10-14 years | 2016 | Both | 66157.87 | 35626.16 | 110539 |
| 10-14 years | 2017 | Male | 32112.02 | 17291.87 | 54949.43 |
| 10-14 years | 2017 | Female | 33328.33 | 18078.19 | 56512.73 |
| 10-14 years | 2017 | Both | 65440.35 | 35131.94 | 110240.8 |
| 10-14 years | 2018 | Male | 32271.18 | 17340.31 | 54007.03 |
| 10-14 years | 2018 | Female | 33412.41 | 17737.87 | 56002.63 |
| 10-14 years | 2018 | Both | 65683.59 | 35165.28 | 109561.1 |
| 10-14 years | 2019 | Male | 33014.7 | 17655.46 | 55964.8 |
| 10-14 years | 2019 | Female | 34150.82 | 18212.52 | 56879.29 |
| 10-14 years | 2019 | Both | 67165.52 | 35931 | 111652.3 |
| 15-19 years | 1990 | Male | 41333.37 | 21539.31 | 68833.62 |
| 15-19 years | 1990 | Female | 64437.73 | 33370.95 | 108759.4 |
| 15-19 years | 1990 | Both | 105771.1 | 55286.98 | 178289.8 |
| 15-19 years | 1991 | Male | 39125.92 | 20494.05 | 65521.65 |
| 15-19 years | 1991 | Female | 61293.44 | 31529.56 | 101922.1 |
| 15-19 years | 1991 | Both | 100419.4 | 51985.01 | 169004.7 |
| 15-19 years | 1992 | Male | 36968.66 | 19390.41 | 62376.88 |
| 15-19 years | 1992 | Female | 58219.66 | 30565.86 | 96762.98 |
| 15-19 years | 1992 | Both | 95188.33 | 50224.84 | 159081.1 |
| 15-19 years | 1993 | Male | 35001.89 | 18144.49 | 58162.12 |
| 15-19 years | 1993 | Female | 55278.19 | 28735.38 | 92424.61 |
| 15-19 years | 1993 | Both | 90280.08 | 46445.32 | 150116.7 |
| 15-19 years | 1994 | Male | 33490.16 | 17703.67 | 55941.47 |
| 15-19 years | 1994 | Female | 52870.48 | 27611.56 | 89389.96 |
| 15-19 years | 1994 | Both | 86360.65 | 45193.92 | 145214.5 |
| 15-19 years | 1995 | Male | 32435.5 | 17089.15 | 54055.11 |
| 15-19 years | 1995 | Female | 51220.92 | 26649.59 | 85717.28 |
| 15-19 years | 1995 | Both | 83656.42 | 43644.88 | 139866.9 |
| 15-19 years | 1996 | Male | 31959.09 | 16811.06 | 53518.01 |
| 15-19 years | 1996 | Female | 50616.62 | 26110.89 | 84994.11 |
| 15-19 years | 1996 | Both | 82575.71 | 42742.38 | 138449.8 |
| 15-19 years | 1997 | Male | 31885.4 | 17003.59 | 53923.19 |
| 15-19 years | 1997 | Female | 50655.91 | 26235.49 | 84746.3 |
| 15-19 years | 1997 | Both | 82541.31 | 43091.55 | 137672.5 |
| 15-19 years | 1998 | Male | 32297.38 | 16994.1 | 54123.95 |
| 15-19 years | 1998 | Female | 51200.07 | 26724.19 | 85786.15 |
| 15-19 years | 1998 | Both | 83497.44 | 43998.56 | 139278.4 |
| 15-19 years | 1999 | Male | 33024.45 | 17353.64 | 55891.32 |
| 15-19 years | 1999 | Female | 52216.75 | 27356.38 | 86722.66 |
| 15-19 years | 1999 | Both | 85241.2 | 44578.05 | 142164.4 |
| 15-19 years | 2000 | Male | 34249.59 | 18049.47 | 57669.31 |
| 15-19 years | 2000 | Female | 53983.23 | 27792.87 | 91685.15 |
| 15-19 years | 2000 | Both | 88232.82 | 45781.57 | 148065.3 |
| 15-19 years | 2001 | Male | 36040.66 | 18803.11 | 60021.17 |
| 15-19 years | 2001 | Female | 56756.7 | 29689.47 | 95982.69 |
| 15-19 years | 2001 | Both | 92797.36 | 48359.48 | 156687.8 |
| 15-19 years | 2002 | Male | 38199.9 | 19892.09 | 63370.61 |
| 15-19 years | 2002 | Female | 60093.73 | 31080.84 | 101638.3 |
| 15-19 years | 2002 | Both | 98293.63 | 51133.55 | 163603.4 |
| 15-19 years | 2003 | Male | 40311.91 | 21263.93 | 66563.9 |
| 15-19 years | 2003 | Female | 63457.48 | 33300.82 | 106129.4 |
| 15-19 years | 2003 | Both | 103769.4 | 54125.08 | 172704 |
| 15-19 years | 2004 | Male | 41945.18 | 22392.62 | 70752.98 |
| 15-19 years | 2004 | Female | 66183.9 | 35029.47 | 111202.9 |
| 15-19 years | 2004 | Both | 108129.1 | 57350.96 | 180161.3 |
| 15-19 years | 2005 | Male | 42674.25 | 22550.17 | 72130.75 |
| 15-19 years | 2005 | Female | 67450.57 | 35614.41 | 114732.9 |
| 15-19 years | 2005 | Both | 110124.8 | 57740.51 | 187408.9 |
| 15-19 years | 2006 | Male | 42097.46 | 22028.84 | 71055.46 |
| 15-19 years | 2006 | Female | 66705.32 | 34787.93 | 111386.1 |
| 15-19 years | 2006 | Both | 108802.8 | 56683.9 | 184348.9 |
| 15-19 years | 2007 | Male | 40589.05 | 21281.82 | 67661.5 |
| 15-19 years | 2007 | Female | 64209.73 | 33564.99 | 108423.2 |
| 15-19 years | 2007 | Both | 104798.8 | 55351.37 | 178429.2 |
| 15-19 years | 2008 | Male | 38376.89 | 20103.85 | 64526.38 |
| 15-19 years | 2008 | Female | 60597.6 | 32096.34 | 102688.9 |
| 15-19 years | 2008 | Both | 98974.49 | 51629.25 | 165659.3 |
| 15-19 years | 2009 | Male | 35892.66 | 18827.68 | 60509.68 |
| 15-19 years | 2009 | Female | 56466.61 | 29421.82 | 94984.46 |
| 15-19 years | 2009 | Both | 92359.27 | 47925.21 | 156128.1 |
| 15-19 years | 2010 | Male | 33509.15 | 17363.03 | 56542.09 |
| 15-19 years | 2010 | Female | 52209.92 | 27330.25 | 88426.94 |
| 15-19 years | 2010 | Both | 85719.07 | 45145.53 | 146293.6 |
| 15-19 years | 2011 | Male | 31398.24 | 16513.91 | 52740.43 |
| 15-19 years | 2011 | Female | 48462.8 | 25534.6 | 81112.35 |
| 15-19 years | 2011 | Both | 79861.04 | 42134.08 | 135889.8 |
| 15-19 years | 2012 | Male | 29693.64 | 15430.27 | 49875.45 |
| 15-19 years | 2012 | Female | 45381.67 | 23675.05 | 77126.25 |
| 15-19 years | 2012 | Both | 75075.31 | 39199.19 | 127527.6 |
| 15-19 years | 2013 | Male | 28231.63 | 14806.97 | 48468.71 |
| 15-19 years | 2013 | Female | 42751.97 | 22592.89 | 71310.6 |
| 15-19 years | 2013 | Both | 70983.6 | 37402.88 | 119545.3 |
| 15-19 years | 2014 | Male | 27051.44 | 14435.52 | 45953.13 |
| 15-19 years | 2014 | Female | 40696.13 | 21339.9 | 68851.66 |
| 15-19 years | 2014 | Both | 67747.57 | 35842.77 | 113960.9 |
| 15-19 years | 2015 | Male | 26193.73 | 13750.86 | 44170.96 |
| 15-19 years | 2015 | Female | 39200.69 | 20460.94 | 65558.72 |
| 15-19 years | 2015 | Both | 65394.41 | 34098.56 | 111180 |
| 15-19 years | 2016 | Male | 25688.42 | 13483.43 | 43377.55 |
| 15-19 years | 2016 | Female | 38297.63 | 19908.37 | 64787.19 |
| 15-19 years | 2016 | Both | 63986.05 | 33361.01 | 108087.2 |
| 15-19 years | 2017 | Male | 25363.76 | 13284.5 | 42753.67 |
| 15-19 years | 2017 | Female | 37692.82 | 19739.77 | 63631.98 |
| 15-19 years | 2017 | Both | 63056.58 | 33370.71 | 105786.3 |
| 15-19 years | 2018 | Male | 25391.68 | 13235.58 | 43143.29 |
| 15-19 years | 2018 | Female | 37500.13 | 19501.74 | 63673 |
| 15-19 years | 2018 | Both | 62891.82 | 33175.37 | 105675.5 |
| 15-19 years | 2019 | Male | 25589.03 | 13558.08 | 43058.17 |
| 15-19 years | 2019 | Female | 37612.93 | 19590.34 | 63469.48 |
| 15-19 years | 2019 | Both | 63201.96 | 33281.14 | 106054.6 |

## Supplementary Table 6. DALY rates of Chinese children and adolescents with AD in different age groups from 1990 to 2019

| **Age group** | **Year** | **Sex** | **Value*** | **Lower*** | **Upper*** |
| --- | --- | --- | --- | --- | --- |
| <5 years | 1990 | Male | 111.9946 | 60.77062 | 189.2403 |
| <5 years | 1990 | Female | 98.29708 | 52.5563 | 166.8634 |
| <5 years | 1990 | Both | 105.5298 | 56.54077 | 178.2295 |
| <5 years | 1991 | Male | 111.704 | 59.30109 | 187.8916 |
| <5 years | 1991 | Female | 97.99183 | 52.35233 | 166.2734 |
| <5 years | 1991 | Both | 105.2432 | 56.29388 | 176.5046 |
| <5 years | 1992 | Male | 111.338 | 59.14249 | 187.2669 |
| <5 years | 1992 | Female | 97.62297 | 52.20302 | 165.8697 |
| <5 years | 1992 | Both | 104.8891 | 55.83618 | 176.8949 |
| <5 years | 1993 | Male | 110.9117 | 58.70151 | 187.5987 |
| <5 years | 1993 | Female | 97.26764 | 51.72568 | 163.8459 |
| <5 years | 1993 | Both | 104.513 | 55.77731 | 176.7487 |
| <5 years | 1994 | Male | 110.3593 | 58.46296 | 187.4269 |
| <5 years | 1994 | Female | 96.72701 | 51.961 | 164.0868 |
| <5 years | 1994 | Both | 103.9858 | 55.75066 | 175.9409 |
| <5 years | 1995 | Male | 110.0296 | 58.2707 | 187.2709 |
| <5 years | 1995 | Female | 96.50089 | 51.18437 | 164.8195 |
| <5 years | 1995 | Both | 103.7257 | 55.40599 | 176.8472 |
| <5 years | 1996 | Male | 110.0435 | 58.23826 | 187.5116 |
| <5 years | 1996 | Female | 96.26363 | 51.51115 | 164.0187 |
| <5 years | 1996 | Both | 103.6428 | 55.24443 | 177.6968 |
| <5 years | 1997 | Male | 109.9434 | 58.19797 | 188.6724 |
| <5 years | 1997 | Female | 96.24459 | 52.25928 | 163.3474 |
| <5 years | 1997 | Both | 103.5986 | 55.18253 | 176.9437 |
| <5 years | 1998 | Male | 109.9642 | 58.85513 | 185.6126 |
| <5 years | 1998 | Female | 96.2896 | 51.51758 | 163.8882 |
| <5 years | 1998 | Both | 103.6472 | 55.34437 | 175.2113 |
| <5 years | 1999 | Male | 109.9465 | 58.49363 | 186.4374 |
| <5 years | 1999 | Female | 96.42835 | 50.8418 | 163.7548 |
| <5 years | 1999 | Both | 103.7166 | 55.39591 | 176.6763 |
| <5 years | 2000 | Male | 110.1758 | 57.85557 | 189.1414 |
| <5 years | 2000 | Female | 96.53861 | 51.4886 | 163.3187 |
| <5 years | 2000 | Both | 103.9042 | 55.37436 | 177.978 |
| <5 years | 2001 | Male | 110.3871 | 59.26415 | 190.0845 |
| <5 years | 2001 | Female | 96.91646 | 51.51183 | 163.0302 |
| <5 years | 2001 | Both | 104.2044 | 55.75888 | 177.463 |
| <5 years | 2002 | Male | 110.8753 | 58.96009 | 189.4863 |
| <5 years | 2002 | Female | 97.69633 | 52.47205 | 166.9194 |
| <5 years | 2002 | Both | 104.8384 | 55.84602 | 180.3148 |
| <5 years | 2003 | Male | 111.4051 | 58.63985 | 189.9831 |
| <5 years | 2003 | Female | 98.58591 | 53.10057 | 169.2663 |
| <5 years | 2003 | Both | 105.5443 | 56.02462 | 181.4408 |
| <5 years | 2004 | Male | 111.7153 | 59.4419 | 189.7354 |
| <5 years | 2004 | Female | 99.12641 | 52.94573 | 169.8571 |
| <5 years | 2004 | Both | 105.9706 | 56.38113 | 182.4656 |
| <5 years | 2005 | Male | 111.8911 | 58.69419 | 191.6355 |
| <5 years | 2005 | Female | 99.7131 | 53.03924 | 171.1189 |
| <5 years | 2005 | Both | 106.3441 | 55.67554 | 181.6122 |
| <5 years | 2006 | Male | 111.7901 | 59.2254 | 189.6156 |
| <5 years | 2006 | Female | 99.50227 | 52.78665 | 172.0387 |
| <5 years | 2006 | Both | 106.2029 | 56.48912 | 181.3367 |
| <5 years | 2007 | Male | 111.4373 | 59.59219 | 188.2759 |
| <5 years | 2007 | Female | 98.94768 | 53.09056 | 170.2174 |
| <5 years | 2007 | Both | 105.7663 | 56.62371 | 178.7237 |
| <5 years | 2008 | Male | 110.9155 | 59.10132 | 187.046 |
| <5 years | 2008 | Female | 98.42805 | 52.20841 | 168.4214 |
| <5 years | 2008 | Both | 105.2497 | 55.84287 | 181.9146 |
| <5 years | 2009 | Male | 110.4205 | 58.64335 | 185.4682 |
| <5 years | 2009 | Female | 97.92784 | 52.21194 | 167.7272 |
| <5 years | 2009 | Both | 104.752 | 55.95316 | 177.8794 |
| <5 years | 2010 | Male | 110.4478 | 59.0598 | 187.712 |
| <5 years | 2010 | Female | 97.95394 | 51.46717 | 167.0883 |
| <5 years | 2010 | Both | 104.7736 | 56.01427 | 179.2939 |
| <5 years | 2011 | Male | 110.2574 | 58.6052 | 187.4097 |
| <5 years | 2011 | Female | 97.68931 | 51.38136 | 167.8615 |
| <5 years | 2011 | Both | 104.54 | 55.97 | 178.8616 |
| <5 years | 2012 | Male | 109.4479 | 57.86113 | 189.1255 |
| <5 years | 2012 | Female | 96.76138 | 51.29876 | 166.1227 |
| <5 years | 2012 | Both | 103.664 | 55.32643 | 177.2522 |
| <5 years | 2013 | Male | 109.0544 | 57.19761 | 186.0858 |
| <5 years | 2013 | Female | 96.4598 | 50.52086 | 166.3776 |
| <5 years | 2013 | Both | 103.2983 | 55.14816 | 175.9665 |
| <5 years | 2014 | Male | 109.7453 | 57.69178 | 184.7024 |
| <5 years | 2014 | Female | 97.13545 | 51.30543 | 166.6879 |
| <5 years | 2014 | Both | 103.968 | 55.40423 | 177.1947 |
| <5 years | 2015 | Male | 108.9063 | 58.67916 | 186.2974 |
| <5 years | 2015 | Female | 96.311 | 50.73822 | 166.187 |
| <5 years | 2015 | Both | 103.1225 | 55.15389 | 177.7029 |
| <5 years | 2016 | Male | 106.9309 | 56.9755 | 184.2187 |
| <5 years | 2016 | Female | 94.73357 | 50.04248 | 163.1649 |
| <5 years | 2016 | Both | 101.3186 | 54.11104 | 175.0612 |
| <5 years | 2017 | Male | 108.0499 | 57.57003 | 185.0369 |
| <5 years | 2017 | Female | 95.74538 | 51.03937 | 163.1183 |
| <5 years | 2017 | Both | 102.3794 | 55.23136 | 174.7318 |
| <5 years | 2018 | Male | 111.7982 | 60.14261 | 190.3622 |
| <5 years | 2018 | Female | 98.93767 | 52.88339 | 170.0505 |
| <5 years | 2018 | Both | 105.8652 | 56.53314 | 180.4296 |
| <5 years | 2019 | Male | 115.4377 | 61.2903 | 196.8087 |
| <5 years | 2019 | Female | 101.9353 | 53.85284 | 173.97 |
| <5 years | 2019 | Both | 109.2046 | 58.10931 | 183.5888 |
| 5-9 years | 1990 | Male | 131.5529 | 69.30112 | 223.7886 |
| 5-9 years | 1990 | Female | 131.9485 | 70.76725 | 223.4488 |
| 5-9 years | 1990 | Both | 131.7428 | 70.20698 | 223.8079 |
| 5-9 years | 1991 | Male | 130.1512 | 68.66823 | 219.6909 |
| 5-9 years | 1991 | Female | 130.4948 | 69.57322 | 220.4342 |
| 5-9 years | 1991 | Both | 130.3156 | 69.34171 | 218.3496 |
| 5-9 years | 1992 | Male | 128.5697 | 69.84351 | 216.43 |
| 5-9 years | 1992 | Female | 129.1962 | 68.95141 | 217.9659 |
| 5-9 years | 1992 | Both | 128.8684 | 68.74922 | 216.8397 |
| 5-9 years | 1993 | Male | 127.2634 | 68.49406 | 215.1717 |
| 5-9 years | 1993 | Female | 127.9475 | 68.19285 | 215.2801 |
| 5-9 years | 1993 | Both | 127.5887 | 68.00499 | 215.6361 |
| 5-9 years | 1994 | Male | 126.6293 | 68.27545 | 213.1501 |
| 5-9 years | 1994 | Female | 126.9616 | 68.46262 | 211.7581 |
| 5-9 years | 1994 | Both | 126.787 | 68.09355 | 213.0209 |
| 5-9 years | 1995 | Male | 126.3665 | 67.79139 | 212.9758 |
| 5-9 years | 1995 | Female | 126.6626 | 67.32319 | 213.2474 |
| 5-9 years | 1995 | Both | 126.5067 | 67.70572 | 212.5947 |
| 5-9 years | 1996 | Male | 126.1367 | 67.31344 | 211.0071 |
| 5-9 years | 1996 | Female | 126.4847 | 67.8749 | 211.8107 |
| 5-9 years | 1996 | Both | 126.3011 | 67.09752 | 213.2765 |
| 5-9 years | 1997 | Male | 126.0956 | 67.93446 | 212.4624 |
| 5-9 years | 1997 | Female | 126.3233 | 67.44661 | 211.8169 |
| 5-9 years | 1997 | Both | 126.2028 | 67.59777 | 211.2188 |
| 5-9 years | 1998 | Male | 125.9809 | 67.63608 | 211.904 |
| 5-9 years | 1998 | Female | 126.3768 | 66.80354 | 212.4438 |
| 5-9 years | 1998 | Both | 126.1665 | 66.84452 | 212.6933 |
| 5-9 years | 1999 | Male | 125.9356 | 67.22778 | 212.1966 |
| 5-9 years | 1999 | Female | 126.32 | 67.07727 | 213.3664 |
| 5-9 years | 1999 | Both | 126.115 | 66.82479 | 211.1533 |
| 5-9 years | 2000 | Male | 125.7739 | 68.22433 | 211.7476 |
| 5-9 years | 2000 | Female | 126.1578 | 66.84485 | 213.0399 |
| 5-9 years | 2000 | Both | 125.9522 | 67.31171 | 212.3348 |
| 5-9 years | 2001 | Male | 125.9967 | 67.55176 | 211.0854 |
| 5-9 years | 2001 | Female | 126.3672 | 66.78791 | 214.97 |
| 5-9 years | 2001 | Both | 126.1681 | 67.30164 | 211.9893 |
| 5-9 years | 2002 | Male | 126.2444 | 68.58259 | 212.457 |
| 5-9 years | 2002 | Female | 127.11 | 68.8702 | 215.5006 |
| 5-9 years | 2002 | Both | 126.6438 | 68.35493 | 213.7191 |
| 5-9 years | 2003 | Male | 126.8608 | 68.3176 | 213.4666 |
| 5-9 years | 2003 | Female | 127.789 | 68.94377 | 216.3335 |
| 5-9 years | 2003 | Both | 127.288 | 69.22776 | 213.1482 |
| 5-9 years | 2004 | Male | 127.1782 | 68.50648 | 214.4546 |
| 5-9 years | 2004 | Female | 128.6124 | 68.78911 | 215.7209 |
| 5-9 years | 2004 | Both | 127.8371 | 69.40604 | 215.0043 |
| 5-9 years | 2005 | Male | 127.3727 | 67.99021 | 215.7936 |
| 5-9 years | 2005 | Female | 128.89 | 68.95412 | 217.484 |
| 5-9 years | 2005 | Both | 128.069 | 68.94729 | 216.5159 |
| 5-9 years | 2006 | Male | 127.2747 | 67.88763 | 215.6705 |
| 5-9 years | 2006 | Female | 128.7514 | 69.44129 | 217.9804 |
| 5-9 years | 2006 | Both | 127.9517 | 69.08542 | 215.5592 |
| 5-9 years | 2007 | Male | 126.8934 | 68.94759 | 213.1264 |
| 5-9 years | 2007 | Female | 128.2302 | 69.4204 | 214.7684 |
| 5-9 years | 2007 | Both | 127.5059 | 68.43111 | 214.3246 |
| 5-9 years | 2008 | Male | 126.5778 | 68.41621 | 214.7694 |
| 5-9 years | 2008 | Female | 127.6639 | 68.90688 | 216.0157 |
| 5-9 years | 2008 | Both | 127.0752 | 69.07241 | 213.8276 |
| 5-9 years | 2009 | Male | 126.0512 | 67.45076 | 211.1405 |
| 5-9 years | 2009 | Female | 127.2156 | 68.40871 | 215.9225 |
| 5-9 years | 2009 | Both | 126.584 | 68.04956 | 212.7895 |
| 5-9 years | 2010 | Male | 125.9963 | 67.596 | 211.4954 |
| 5-9 years | 2010 | Female | 127.1192 | 68.07469 | 217.1163 |
| 5-9 years | 2010 | Both | 126.5096 | 68.49496 | 214.2323 |
| 5-9 years | 2011 | Male | 126.114 | 68.06911 | 213.2643 |
| 5-9 years | 2011 | Female | 127.2295 | 68.66393 | 217.1708 |
| 5-9 years | 2011 | Both | 126.6232 | 68.27632 | 213.5331 |
| 5-9 years | 2012 | Male | 126.19 | 67.90639 | 214.0951 |
| 5-9 years | 2012 | Female | 127.3865 | 68.41873 | 214.5475 |
| 5-9 years | 2012 | Both | 126.7354 | 68.25954 | 213.3411 |
| 5-9 years | 2013 | Male | 126.2336 | 67.94532 | 213.3824 |
| 5-9 years | 2013 | Female | 127.5356 | 69.33259 | 215.4675 |
| 5-9 years | 2013 | Both | 126.8266 | 68.81042 | 214.4786 |
| 5-9 years | 2014 | Male | 126.2893 | 67.67055 | 213.1796 |
| 5-9 years | 2014 | Female | 127.5072 | 69.19004 | 216.4111 |
| 5-9 years | 2014 | Both | 126.8439 | 67.97103 | 214.9663 |
| 5-9 years | 2015 | Male | 126.2566 | 68.15082 | 213.0226 |
| 5-9 years | 2015 | Female | 127.4557 | 69.19221 | 218.5539 |
| 5-9 years | 2015 | Both | 126.803 | 68.6996 | 215.3951 |
| 5-9 years | 2016 | Male | 126.3024 | 68.44457 | 210.9983 |
| 5-9 years | 2016 | Female | 127.6269 | 69.8069 | 216.5941 |
| 5-9 years | 2016 | Both | 126.9069 | 68.16813 | 214.3325 |
| 5-9 years | 2017 | Male | 126.3684 | 68.95266 | 213.3693 |
| 5-9 years | 2017 | Female | 127.6334 | 68.62549 | 218.3784 |
| 5-9 years | 2017 | Both | 126.9469 | 68.93239 | 215.1529 |
| 5-9 years | 2018 | Male | 128.135 | 69.69114 | 214.1474 |
| 5-9 years | 2018 | Female | 129.2474 | 69.38255 | 219.9366 |
| 5-9 years | 2018 | Both | 128.6448 | 70.22238 | 216.6694 |
| 5-9 years | 2019 | Male | 131.9397 | 71.12463 | 220.6915 |
| 5-9 years | 2019 | Female | 132.7176 | 70.79409 | 224.8599 |
| 5-9 years | 2019 | Both | 132.2971 | 71.38591 | 221.7621 |
| 10-14 years | 1990 | Male | 85.89128 | 45.40005 | 145.4894 |
| 10-14 years | 1990 | Female | 104.7572 | 55.87117 | 177.9895 |
| 10-14 years | 1990 | Both | 95.01879 | 50.60531 | 162.3288 |
| 10-14 years | 1991 | Male | 84.96931 | 45.04009 | 145.233 |
| 10-14 years | 1991 | Female | 103.7235 | 55.18581 | 177.4082 |
| 10-14 years | 1991 | Both | 94.04152 | 50.08106 | 160.8174 |
| 10-14 years | 1992 | Male | 84.18524 | 44.79267 | 142.7847 |
| 10-14 years | 1992 | Female | 102.8942 | 55.12465 | 173.5408 |
| 10-14 years | 1992 | Both | 93.23686 | 49.72863 | 158.8936 |
| 10-14 years | 1993 | Male | 83.44883 | 45.1206 | 142.686 |
| 10-14 years | 1993 | Female | 102.1207 | 54.3751 | 173.9981 |
| 10-14 years | 1993 | Both | 92.47828 | 49.18477 | 158.8824 |
| 10-14 years | 1994 | Male | 83.0536 | 44.00669 | 140.7545 |
| 10-14 years | 1994 | Female | 101.691 | 54.11778 | 170.9384 |
| 10-14 years | 1994 | Both | 92.05338 | 49.05747 | 154.1536 |
| 10-14 years | 1995 | Male | 82.96336 | 44.62232 | 139.4373 |
| 10-14 years | 1995 | Female | 101.4114 | 54.29359 | 170.0661 |
| 10-14 years | 1995 | Both | 91.85241 | 49.51998 | 153.7196 |
| 10-14 years | 1996 | Male | 82.88274 | 44.59647 | 140.552 |
| 10-14 years | 1996 | Female | 101.3324 | 54.58617 | 170.686 |
| 10-14 years | 1996 | Both | 91.75198 | 49.41694 | 153.6424 |
| 10-14 years | 1997 | Male | 82.85856 | 44.62753 | 142.8361 |
| 10-14 years | 1997 | Female | 101.4653 | 54.75452 | 171.0525 |
| 10-14 years | 1997 | Both | 91.78643 | 49.44886 | 156.1372 |
| 10-14 years | 1998 | Male | 82.82973 | 44.25772 | 140.3697 |
| 10-14 years | 1998 | Female | 101.3275 | 54.6031 | 170.0839 |
| 10-14 years | 1998 | Both | 91.69545 | 49.52981 | 155.0016 |
| 10-14 years | 1999 | Male | 82.74041 | 45.0329 | 140.4183 |
| 10-14 years | 1999 | Female | 101.2937 | 54.50824 | 170.5263 |
| 10-14 years | 1999 | Both | 91.6273 | 49.9342 | 154.3568 |
| 10-14 years | 2000 | Male | 82.88856 | 44.67781 | 140.4603 |
| 10-14 years | 2000 | Female | 101.3448 | 54.60754 | 171.1402 |
| 10-14 years | 2000 | Both | 91.72274 | 49.9854 | 153.135 |
| 10-14 years | 2001 | Male | 82.85872 | 44.45901 | 141.321 |
| 10-14 years | 2001 | Female | 101.3494 | 54.08208 | 169.6833 |
| 10-14 years | 2001 | Both | 91.69391 | 49.04849 | 154.4656 |
| 10-14 years | 2002 | Male | 82.89227 | 44.3619 | 141.547 |
| 10-14 years | 2002 | Female | 101.4537 | 53.87819 | 172.5328 |
| 10-14 years | 2002 | Both | 91.73654 | 49.56265 | 155.4372 |
| 10-14 years | 2003 | Male | 83.19082 | 44.39249 | 140.8187 |
| 10-14 years | 2003 | Female | 101.536 | 55.0307 | 171.5797 |
| 10-14 years | 2003 | Both | 91.89896 | 49.47324 | 154.7441 |
| 10-14 years | 2004 | Male | 83.11157 | 44.42031 | 140.2698 |
| 10-14 years | 2004 | Female | 101.6505 | 54.66485 | 173.4964 |
| 10-14 years | 2004 | Both | 91.87248 | 49.35979 | 154.8658 |
| 10-14 years | 2005 | Male | 83.2435 | 45.5959 | 139.6281 |
| 10-14 years | 2005 | Female | 101.7218 | 54.47533 | 171.1657 |
| 10-14 years | 2005 | Both | 91.93437 | 49.7183 | 154.8373 |
| 10-14 years | 2006 | Male | 83.0818 | 44.44469 | 140.4512 |
| 10-14 years | 2006 | Female | 101.4311 | 54.99999 | 172.1811 |
| 10-14 years | 2006 | Both | 91.67443 | 49.82501 | 154.8883 |
| 10-14 years | 2007 | Male | 82.68494 | 44.15383 | 138.3791 |
| 10-14 years | 2007 | Female | 101.1524 | 53.98614 | 171.4112 |
| 10-14 years | 2007 | Both | 91.29924 | 49.3451 | 153.7866 |
| 10-14 years | 2008 | Male | 82.24501 | 44.40134 | 139.0038 |
| 10-14 years | 2008 | Female | 100.7241 | 54.10538 | 171.9867 |
| 10-14 years | 2008 | Both | 90.83481 | 49.00605 | 152.5146 |
| 10-14 years | 2009 | Male | 82.02104 | 44.24495 | 139.1273 |
| 10-14 years | 2009 | Female | 100.6788 | 54.06079 | 171.309 |
| 10-14 years | 2009 | Both | 90.66857 | 49.849 | 153.7941 |
| 10-14 years | 2010 | Male | 81.95329 | 43.95227 | 137.697 |
| 10-14 years | 2010 | Female | 100.5874 | 53.88376 | 170.3375 |
| 10-14 years | 2010 | Both | 90.57111 | 48.85229 | 152.3195 |
| 10-14 years | 2011 | Male | 81.91944 | 43.93659 | 137.8612 |
| 10-14 years | 2011 | Female | 100.7058 | 54.1746 | 170.3015 |
| 10-14 years | 2011 | Both | 90.59625 | 48.74403 | 152.263 |
| 10-14 years | 2012 | Male | 82.15343 | 44.6583 | 138.8508 |
| 10-14 years | 2012 | Female | 100.7684 | 54.51202 | 170.625 |
| 10-14 years | 2012 | Both | 90.74298 | 49.93424 | 153.5273 |
| 10-14 years | 2013 | Male | 82.18893 | 43.82303 | 137.6822 |
| 10-14 years | 2013 | Female | 100.8756 | 54.20706 | 170.1946 |
| 10-14 years | 2013 | Both | 90.80397 | 48.56152 | 152.5861 |
| 10-14 years | 2014 | Male | 82.35869 | 44.25923 | 137.9166 |
| 10-14 years | 2014 | Female | 100.9336 | 54.35531 | 171.4723 |
| 10-14 years | 2014 | Both | 90.91335 | 49.3629 | 153.2075 |
| 10-14 years | 2015 | Male | 82.37133 | 43.67816 | 138.4242 |
| 10-14 years | 2015 | Female | 101.0112 | 54.56855 | 169.6242 |
| 10-14 years | 2015 | Both | 90.94432 | 49.32857 | 153.4361 |
| 10-14 years | 2016 | Male | 82.38991 | 43.18865 | 139.3477 |
| 10-14 years | 2016 | Female | 101.1462 | 54.01565 | 171.0151 |
| 10-14 years | 2016 | Both | 91.00151 | 49.00451 | 152.0487 |
| 10-14 years | 2017 | Male | 82.48931 | 44.41934 | 141.154 |
| 10-14 years | 2017 | Female | 101.1647 | 54.8745 | 171.5386 |
| 10-14 years | 2017 | Both | 91.04958 | 48.88037 | 153.3821 |
| 10-14 years | 2018 | Male | 83.63453 | 44.93943 | 139.9655 |
| 10-14 years | 2018 | Female | 102.544 | 54.43825 | 171.8744 |
| 10-14 years | 2018 | Both | 92.29187 | 49.41066 | 153.9441 |
| 10-14 years | 2019 | Male | 86.15275 | 46.07239 | 146.0416 |
| 10-14 years | 2019 | Female | 105.6224 | 56.32807 | 175.9175 |
| 10-14 years | 2019 | Both | 95.06251 | 50.85483 | 158.0267 |
| 15-19 years | 1990 | Male | 63.43364 | 33.05602 | 105.6378 |
| 15-19 years | 1990 | Female | 104.3996 | 54.06636 | 176.208 |
| 15-19 years | 1990 | Both | 83.36165 | 43.57347 | 140.516 |
| 15-19 years | 1991 | Male | 63.01544 | 33.00731 | 105.5279 |
| 15-19 years | 1991 | Female | 103.8589 | 53.42538 | 172.7022 |
| 15-19 years | 1991 | Both | 82.91892 | 42.92539 | 139.5516 |
| 15-19 years | 1992 | Male | 62.68605 | 32.87942 | 105.7696 |
| 15-19 years | 1992 | Female | 103.6558 | 54.42025 | 172.2793 |
| 15-19 years | 1992 | Both | 82.67135 | 43.62043 | 138.1624 |
| 15-19 years | 1993 | Male | 62.3345 | 32.31333 | 103.5803 |
| 15-19 years | 1993 | Female | 103.3671 | 53.73352 | 172.8288 |
| 15-19 years | 1993 | Both | 82.35034 | 42.3658 | 136.9312 |
| 15-19 years | 1994 | Male | 62.18332 | 32.87154 | 103.8701 |
| 15-19 years | 1994 | Female | 103.2347 | 53.91421 | 174.5424 |
| 15-19 years | 1994 | Both | 82.19263 | 43.01273 | 138.2061 |
| 15-19 years | 1995 | Male | 62.06134 | 32.69798 | 103.4278 |
| 15-19 years | 1995 | Female | 103.1907 | 53.6888 | 172.6877 |
| 15-19 years | 1995 | Both | 82.09596 | 42.83076 | 137.2579 |
| 15-19 years | 1996 | Male | 62.05211 | 32.64053 | 103.9111 |
| 15-19 years | 1996 | Female | 103.2962 | 53.28599 | 173.4523 |
| 15-19 years | 1996 | Both | 82.16075 | 42.52759 | 137.7541 |
| 15-19 years | 1997 | Male | 62.00527 | 33.06568 | 104.8606 |
| 15-19 years | 1997 | Female | 103.3503 | 53.52676 | 172.903 |
| 15-19 years | 1997 | Both | 82.18178 | 42.90386 | 137.0728 |
| 15-19 years | 1998 | Male | 62.16329 | 32.70882 | 104.1733 |
| 15-19 years | 1998 | Female | 103.3777 | 53.95861 | 173.2102 |
| 15-19 years | 1998 | Both | 82.27735 | 43.35564 | 137.2432 |
| 15-19 years | 1999 | Male | 62.16695 | 32.66741 | 105.2127 |
| 15-19 years | 1999 | Female | 103.3091 | 54.1237 | 171.5779 |
| 15-19 years | 1999 | Both | 82.22647 | 43.00146 | 137.1365 |
| 15-19 years | 2000 | Male | 62.22678 | 32.79341 | 104.7772 |
| 15-19 years | 2000 | Female | 103.3669 | 53.2177 | 175.5585 |
| 15-19 years | 2000 | Both | 82.25701 | 42.68089 | 138.0372 |
| 15-19 years | 2001 | Male | 62.27961 | 32.49248 | 103.7188 |
| 15-19 years | 2001 | Female | 103.5531 | 54.16867 | 175.1212 |
| 15-19 years | 2001 | Both | 82.35594 | 42.91814 | 139.0575 |
| 15-19 years | 2002 | Male | 62.392 | 32.4898 | 103.5034 |
| 15-19 years | 2002 | Female | 103.6624 | 53.61483 | 175.3274 |
| 15-19 years | 2002 | Both | 82.4637 | 42.89862 | 137.2555 |
| 15-19 years | 2003 | Male | 62.46035 | 32.94691 | 103.1359 |
| 15-19 years | 2003 | Female | 103.7075 | 54.42299 | 173.4455 |
| 15-19 years | 2003 | Both | 82.53426 | 43.04905 | 137.3622 |
| 15-19 years | 2004 | Male | 62.47527 | 33.3527 | 105.3831 |
| 15-19 years | 2004 | Female | 103.7692 | 54.92243 | 174.3542 |
| 15-19 years | 2004 | Both | 82.59251 | 43.80653 | 137.6131 |
| 15-19 years | 2005 | Male | 62.46812 | 33.00976 | 105.5876 |
| 15-19 years | 2005 | Female | 103.7962 | 54.80516 | 176.5564 |
| 15-19 years | 2005 | Both | 82.61592 | 43.31707 | 140.5946 |
| 15-19 years | 2006 | Male | 62.41444 | 32.66035 | 105.3481 |
| 15-19 years | 2006 | Female | 104.0908 | 54.28509 | 173.8133 |
| 15-19 years | 2006 | Both | 82.7196 | 43.09513 | 140.1551 |
| 15-19 years | 2007 | Male | 62.46004 | 32.74931 | 104.1202 |
| 15-19 years | 2007 | Female | 104.3929 | 54.57035 | 176.2758 |
| 15-19 years | 2007 | Both | 82.85027 | 43.75887 | 141.0599 |
| 15-19 years | 2008 | Male | 62.40871 | 32.693 | 104.9332 |
| 15-19 years | 2008 | Female | 104.783 | 55.49974 | 177.5656 |
| 15-19 years | 2008 | Both | 82.94575 | 43.26799 | 138.8311 |
| 15-19 years | 2009 | Male | 62.34626 | 32.70406 | 105.1065 |
| 15-19 years | 2009 | Female | 105.1795 | 54.80359 | 176.9262 |
| 15-19 years | 2009 | Both | 83.01523 | 43.0766 | 140.3325 |
| 15-19 years | 2010 | Male | 62.34109 | 32.30253 | 105.192 |
| 15-19 years | 2010 | Female | 105.1858 | 55.06144 | 178.1511 |
| 15-19 years | 2010 | Both | 82.9107 | 43.66645 | 141.5006 |
| 15-19 years | 2011 | Male | 62.24193 | 32.73616 | 104.5494 |
| 15-19 years | 2011 | Female | 105.0642 | 55.35737 | 175.8464 |
| 15-19 years | 2011 | Both | 82.69559 | 43.62956 | 140.713 |
| 15-19 years | 2012 | Male | 62.31877 | 32.38389 | 104.6748 |
| 15-19 years | 2012 | Female | 105.1389 | 54.84963 | 178.6837 |
| 15-19 years | 2012 | Both | 82.67154 | 43.16542 | 140.431 |
| 15-19 years | 2013 | Male | 62.28458 | 32.66712 | 106.9316 |
| 15-19 years | 2013 | Female | 105.0198 | 55.49922 | 175.1739 |
| 15-19 years | 2013 | Both | 82.5052 | 43.47387 | 138.9491 |
| 15-19 years | 2014 | Male | 62.23746 | 33.21191 | 105.7247 |
| 15-19 years | 2014 | Female | 105.0342 | 55.07693 | 177.7018 |
| 15-19 years | 2014 | Both | 82.40744 | 43.59877 | 138.6209 |
| 15-19 years | 2015 | Male | 62.24037 | 32.67417 | 104.9571 |
| 15-19 years | 2015 | Female | 105.11 | 54.86253 | 175.7846 |
| 15-19 years | 2015 | Both | 82.38177 | 42.95626 | 140.0609 |
| 15-19 years | 2016 | Male | 62.32681 | 32.71433 | 105.2453 |
| 15-19 years | 2016 | Female | 105.2933 | 54.73496 | 178.1222 |
| 15-19 years | 2016 | Both | 82.46901 | 42.99765 | 139.3092 |
| 15-19 years | 2017 | Male | 62.35043 | 32.65661 | 105.0991 |
| 15-19 years | 2017 | Female | 105.3497 | 55.17179 | 177.8485 |
| 15-19 years | 2017 | Both | 82.47205 | 43.64573 | 138.3585 |
| 15-19 years | 2018 | Male | 62.94035 | 32.80807 | 106.9426 |
| 15-19 years | 2018 | Female | 106.0152 | 55.13261 | 180.0074 |
| 15-19 years | 2018 | Both | 83.06401 | 43.81618 | 139.5703 |
| 15-19 years | 2019 | Male | 63.81982 | 33.81426 | 107.3884 |
| 15-19 years | 2019 | Female | 107.3414 | 55.90775 | 181.1319 |
| 15-19 years | 2019 | Both | 84.1165 | 44.2944 | 141.1498 |

## Supplementary Table 7. Annual percent change based on joinpoint models for incidence cases of Chinese children and adolescents with AD in different age groups from 1990 to 2019

| **Cohort** | **Segment** | **Lower Endpoint** | **Upper Endpoint** | **APC** | **Lower CI** | **Upper CI** |
| --- | --- | --- | --- | --- | --- | --- |
| "<5 years" / Both - 5 Joinpoints | 1 | 1990 | 1992 | -1.17* | -1.91 | -0.42 |
| "<5 years" / Both - 5 Joinpoints | 2 | 1992 | 2001 | -3.97* | -4.05 | -3.88 |
| "<5 years" / Both - 5 Joinpoints | 3 | 2001 | 2009 | -1.74* | -1.86 | -1.61 |
| "<5 years" / Both - 5 Joinpoints | 4 | 2009 | 2012 | -0.31 | -1.28 | 0.67 |
| "<5 years" / Both - 5 Joinpoints | 5 | 2012 | 2016 | 1.97* | 1.48 | 2.46 |
| "<5 years" / Both - 5 Joinpoints | 6 | 2016 | 2019 | 4.76* | 4.29 | 5.24 |
| "<5 years" / Female - 5 Joinpoints | 1 | 1990 | 1992 | -1.22* | -1.95 | -0.49 |
| "<5 years" / Female - 5 Joinpoints | 2 | 1992 | 2000 | -4.38* | -4.48 | -4.27 |
| "<5 years" / Female - 5 Joinpoints | 3 | 2000 | 2008 | -2.08* | -2.2 | -1.96 |
| "<5 years" / Female - 5 Joinpoints | 4 | 2008 | 2012 | -0.39 | -0.88 | 0.09 |
| "<5 years" / Female - 5 Joinpoints | 5 | 2012 | 2015 | 1.90* | 0.92 | 2.89 |
| "<5 years" / Female - 5 Joinpoints | 6 | 2015 | 2019 | 4.26* | 3.97 | 4.56 |
| "<5 years" / Male - 5 Joinpoints | 1 | 1990 | 1992 | -0.96* | -1.6 | -0.31 |
| "<5 years" / Male - 5 Joinpoints | 2 | 1992 | 2001 | -3.75* | -3.82 | -3.67 |
| "<5 years" / Male - 5 Joinpoints | 3 | 2001 | 2008 | -1.77* | -1.9 | -1.63 |
| "<5 years" / Male - 5 Joinpoints | 4 | 2008 | 2012 | -0.64* | -1.06 | -0.23 |
| "<5 years" / Male - 5 Joinpoints | 5 | 2012 | 2016 | 1.78* | 1.37 | 2.21 |
| "<5 years" / Male - 5 Joinpoints | 6 | 2016 | 2019 | 4.77* | 4.37 | 5.18 |
| "5-9 years" / Both - 4 Joinpoints | 1 | 1990 | 1996 | 1.01* | 0.85 | 1.17 |
| "5-9 years" / Both - 4 Joinpoints | 2 | 1996 | 2002 | -5.24* | -5.45 | -5.03 |
| "5-9 years" / Both - 4 Joinpoints | 3 | 2002 | 2007 | -2.66* | -3 | -2.32 |
| "5-9 years" / Both - 4 Joinpoints | 4 | 2007 | 2016 | -0.96* | -1.09 | -0.84 |
| "5-9 years" / Both - 4 Joinpoints | 5 | 2016 | 2019 | 3.15* | 2.56 | 3.75 |
| "5-9 years" / Female - 4 Joinpoints | 1 | 1990 | 1996 | 0.86* | 0.69 | 1.02 |
| "5-9 years" / Female - 4 Joinpoints | 2 | 1996 | 2002 | -5.58* | -5.8 | -5.36 |
| "5-9 years" / Female - 4 Joinpoints | 3 | 2002 | 2006 | -3.17* | -3.74 | -2.6 |
| "5-9 years" / Female - 4 Joinpoints | 4 | 2006 | 2016 | -1.07* | -1.18 | -0.96 |
| "5-9 years" / Female - 4 Joinpoints | 5 | 2016 | 2019 | 3.36* | 2.72 | 4 |
| "5-9 years" / Male - 4 Joinpoints | 1 | 1990 | 1996 | 1.18* | 1.03 | 1.33 |
| "5-9 years" / Male - 4 Joinpoints | 2 | 1996 | 2002 | -4.82* | -5.01 | -4.62 |
| "5-9 years" / Male - 4 Joinpoints | 3 | 2002 | 2008 | -2.35* | -2.57 | -2.13 |
| "5-9 years" / Male - 4 Joinpoints | 4 | 2008 | 2017 | -0.70* | -0.82 | -0.59 |
| "5-9 years" / Male - 4 Joinpoints | 5 | 2017 | 2019 | 4.83* | 3.74 | 5.94 |
| "10-14 years" / Both - 5 Joinpoints | 1 | 1990 | 1994 | 0.1 | -0.37 | 0.58 |
| "10-14 years" / Both - 5 Joinpoints | 2 | 1994 | 2000 | 4.12* | 3.8 | 4.45 |
| "10-14 years" / Both - 5 Joinpoints | 3 | 2000 | 2003 | -4.33* | -5.63 | -3.02 |
| "10-14 years" / Both - 5 Joinpoints | 4 | 2003 | 2006 | -7.32* | -8.7 | -5.91 |
| "10-14 years" / Both - 5 Joinpoints | 5 | 2006 | 2010 | -4.55* | -5.34 | -3.75 |
| "10-14 years" / Both - 5 Joinpoints | 6 | 2010 | 2019 | -0.86* | -1.02 | -0.7 |
| "10-14 years" / Female - 5 Joinpoints | 1 | 1990 | 1994 | -0.01 | -0.49 | 0.47 |
| "10-14 years" / Female - 5 Joinpoints | 2 | 1994 | 2000 | 4.02* | 3.68 | 4.35 |
| "10-14 years" / Female - 5 Joinpoints | 3 | 2000 | 2003 | -4.53* | -5.84 | -3.19 |
| "10-14 years" / Female - 5 Joinpoints | 4 | 2003 | 2006 | -7.66* | -9.07 | -6.23 |
| "10-14 years" / Female - 5 Joinpoints | 5 | 2006 | 2010 | -4.69* | -5.5 | -3.87 |
| "10-14 years" / Female - 5 Joinpoints | 6 | 2010 | 2019 | -0.94* | -1.11 | -0.77 |
| "10-14 years" / Male - 5 Joinpoints | 1 | 1990 | 1993 | -0.80* | -1.52 | -0.08 |
| "10-14 years" / Male - 5 Joinpoints | 2 | 1993 | 2000 | 4.00* | 3.76 | 4.24 |
| "10-14 years" / Male - 5 Joinpoints | 3 | 2000 | 2003 | -3.84* | -5.08 | -2.57 |
| "10-14 years" / Male - 5 Joinpoints | 4 | 2003 | 2006 | -6.80* | -8.12 | -5.46 |
| "10-14 years" / Male - 5 Joinpoints | 5 | 2006 | 2010 | -4.34* | -5.08 | -3.58 |
| "10-14 years" / Male - 5 Joinpoints | 6 | 2010 | 2019 | -0.74* | -0.89 | -0.59 |
| "15-19 years" / Both - 5 Joinpoints | 1 | 1990 | 1995 | -4.45* | -4.7 | -4.19 |
| "15-19 years" / Both - 5 Joinpoints | 2 | 1995 | 1999 | 0.39 | -0.25 | 1.02 |
| "15-19 years" / Both - 5 Joinpoints | 3 | 1999 | 2004 | 5.37* | 4.98 | 5.77 |
| "15-19 years" / Both - 5 Joinpoints | 4 | 2004 | 2007 | -0.8 | -1.89 | 0.3 |
| "15-19 years" / Both - 5 Joinpoints | 5 | 2007 | 2014 | -6.38* | -6.58 | -6.19 |
| "15-19 years" / Both - 5 Joinpoints | 6 | 2014 | 2019 | -1.64* | -1.95 | -1.32 |
| "15-19 years" / Female - 5 Joinpoints | 1 | 1990 | 1995 | -4.44* | -4.69 | -4.19 |
| "15-19 years" / Female - 5 Joinpoints | 2 | 1995 | 1999 | 0.37 | -0.26 | 1 |
| "15-19 years" / Female - 5 Joinpoints | 3 | 1999 | 2004 | 5.41* | 5.02 | 5.8 |
| "15-19 years" / Female - 5 Joinpoints | 4 | 2004 | 2007 | -0.69 | -1.77 | 0.39 |
| "15-19 years" / Female - 5 Joinpoints | 5 | 2007 | 2014 | -6.61* | -6.81 | -6.41 |
| "15-19 years" / Female - 5 Joinpoints | 6 | 2014 | 2019 | -1.77* | -2.09 | -1.46 |
| "15-19 years" / Male - 5 Joinpoints | 1 | 1990 | 1995 | -4.45* | -4.72 | -4.18 |
| "15-19 years" / Male - 5 Joinpoints | 2 | 1995 | 1999 | 0.43 | -0.24 | 1.11 |
| "15-19 years" / Male - 5 Joinpoints | 3 | 1999 | 2004 | 5.29* | 4.87 | 5.7 |
| "15-19 years" / Male - 5 Joinpoints | 4 | 2004 | 2007 | -1.06 | -2.22 | 0.1 |
| "15-19 years" / Male - 5 Joinpoints | 5 | 2007 | 2014 | -5.84* | -6.05 | -5.63 |
| "15-19 years" / Male - 5 Joinpoints | 6 | 2014 | 2019 | -1.33* | -1.66 | -1 |

APC, Annual percent change

**P*<0.05

## Supplementary Table 8. Annual percent change based on joinpoint models for incidence rates of Chinese children and adolescents with AD in different age groups from 1990 to 2019

| **Cohort** | **Segment** | **Lower Endpoint** | **Upper Endpoint** | **APC** | **Lower CI** | **Upper CI** | ***P*** |
| --- | --- | --- | --- | --- | --- | --- | --- |
| "<5 years" / Both - 3 Joinpoints | 1 | 1990 | 1997 | -0.49* | -0.65 | -0.34 | < 0.001 |
| "<5 years" / Both - 3 Joinpoints | 2 | 1997 | 2005 | 0.39* | 0.24 | 0.55 | < 0.001 |
| "<5 years" / Both - 3 Joinpoints | 3 | 2005 | 2017 | -0.37* | -0.44 | -0.29 | < 0.001 |
| "<5 years" / Both - 3 Joinpoints | 4 | 2017 | 2019 | 3.99* | 2.79 | 5.2 | < 0.001 |
| "<5 years" / Female - 4 Joinpoints | 1 | 1990 | 1995 | -0.64* | -0.86 | -0.41 | < 0.001 |
| "<5 years" / Female - 4 Joinpoints | 2 | 1995 | 2000 | 0.02 | -0.32 | 0.35 | 0.922 |
| "<5 years" / Female - 4 Joinpoints | 3 | 2000 | 2005 | 0.67* | 0.35 | 1 | < 0.001 |
| "<5 years" / Female - 4 Joinpoints | 4 | 2005 | 2017 | -0.40* | -0.47 | -0.33 | < 0.001 |
| "<5 years" / Female - 4 Joinpoints | 5 | 2017 | 2019 | 3.87* | 2.8 | 4.96 | < 0.001 |
| "<5 years" / Male - 3 Joinpoints | 1 | 1990 | 1996 | -0.57* | -0.76 | -0.39 | < 0.001 |
| "<5 years" / Male - 3 Joinpoints | 2 | 1996 | 2005 | 0.25* | 0.13 | 0.37 | < 0.001 |
| "<5 years" / Male - 3 Joinpoints | 3 | 2005 | 2017 | -0.35* | -0.42 | -0.28 | < 0.001 |
| "<5 years" / Male - 3 Joinpoints | 4 | 2017 | 2019 | 4.12* | 3 | 5.25 | < 0.001 |
| "5-9 years" / Both - 5 Joinpoints | 1 | 1990 | 1994 | -1.23* | -1.28 | -1.18 | < 0.001 |
| "5-9 years" / Both - 5 Joinpoints | 2 | 1994 | 2001 | -0.09* | -0.12 | -0.07 | < 0.001 |
| "5-9 years" / Both - 5 Joinpoints | 3 | 2001 | 2005 | 0.23* | 0.15 | 0.3 | < 0.001 |
| "5-9 years" / Both - 5 Joinpoints | 4 | 2005 | 2009 | -0.51* | -0.58 | -0.43 | < 0.001 |
| "5-9 years" / Both - 5 Joinpoints | 5 | 2009 | 2017 | -0.03* | -0.06 | -0.01 | 0.004 |
| "5-9 years" / Both - 5 Joinpoints | 6 | 2017 | 2019 | 2.57* | 2.41 | 2.73 | < 0.001 |
| "5-9 years" / Female - 4 Joinpoints | 1 | 1990 | 1994 | -1.11* | -1.19 | -1.03 | < 0.001 |
| "5-9 years" / Female - 4 Joinpoints | 2 | 1994 | 2006 | -0.02 | -0.03 | 0 | 0.063 |
| "5-9 years" / Female - 4 Joinpoints | 3 | 2006 | 2009 | -0.43* | -0.68 | -0.18 | 0.002 |
| "5-9 years" / Female - 4 Joinpoints | 4 | 2009 | 2017 | -0.02 | -0.05 | 0.02 | 0.36 |
| "5-9 years" / Female - 4 Joinpoints | 5 | 2017 | 2019 | 2.27* | 2 | 2.54 | < 0.001 |
| "5-9 years" / Male - 5 Joinpoints | 1 | 1990 | 1994 | -1.35* | -1.4 | -1.29 | < 0.001 |
| "5-9 years" / Male - 5 Joinpoints | 2 | 1994 | 2001 | -0.03* | -0.06 | -0.01 | 0.023 |
| "5-9 years" / Male - 5 Joinpoints | 3 | 2001 | 2005 | 0.44* | 0.36 | 0.52 | < 0.001 |
| "5-9 years" / Male - 5 Joinpoints | 4 | 2005 | 2009 | -0.62* | -0.7 | -0.54 | < 0.001 |
| "5-9 years" / Male - 5 Joinpoints | 5 | 2009 | 2017 | -0.04* | -0.06 | -0.02 | 0.002 |
| "5-9 years" / Male - 5 Joinpoints | 6 | 2017 | 2019 | 2.78* | 2.6 | 2.96 | < 0.001 |
| "10-14 years" / Both - 5 Joinpoints | 1 | 1990 | 1994 | -0.33* | -0.36 | -0.31 | < 0.001 |
| "10-14 years" / Both - 5 Joinpoints | 2 | 1994 | 2001 | -0.05* | -0.06 | -0.04 | < 0.001 |
| "10-14 years" / Both - 5 Joinpoints | 3 | 2001 | 2005 | -0.27* | -0.31 | -0.24 | < 0.001 |
| "10-14 years" / Both - 5 Joinpoints | 4 | 2005 | 2011 | 0.11* | 0.09 | 0.13 | < 0.001 |
| "10-14 years" / Both - 5 Joinpoints | 5 | 2011 | 2017 | 0 | -0.02 | 0.01 | 0.729 |
| "10-14 years" / Both - 5 Joinpoints | 6 | 2017 | 2019 | 0.84* | 0.76 | 0.91 | < 0.001 |
| "10-14 years" / Female - 5 Joinpoints | 1 | 1990 | 1994 | -0.36* | -0.38 | -0.34 | < 0.001 |
| "10-14 years" / Female - 5 Joinpoints | 2 | 1994 | 2001 | -0.01* | -0.02 | 0 | 0.036 |
| "10-14 years" / Female - 5 Joinpoints | 3 | 2001 | 2005 | -0.21* | -0.23 | -0.18 | < 0.001 |
| "10-14 years" / Female - 5 Joinpoints | 4 | 2005 | 2010 | 0.29* | 0.27 | 0.3 | < 0.001 |
| "10-14 years" / Female - 5 Joinpoints | 5 | 2010 | 2017 | 0.04* | 0.03 | 0.05 | < 0.001 |
| "10-14 years" / Female - 5 Joinpoints | 6 | 2017 | 2019 | 0.90* | 0.85 | 0.96 | < 0.001 |
| "10-14 years" / Male - 5 Joinpoints | 1 | 1990 | 1994 | -0.23* | -0.26 | -0.2 | < 0.001 |
| "10-14 years" / Male - 5 Joinpoints | 2 | 1994 | 2000 | 0 | -0.02 | 0.02 | 0.96 |
| "10-14 years" / Male - 5 Joinpoints | 3 | 2000 | 2005 | -0.08* | -0.11 | -0.05 | < 0.001 |
| "10-14 years" / Male - 5 Joinpoints | 4 | 2005 | 2014 | 0.08* | 0.07 | 0.09 | < 0.001 |
| "10-14 years" / Male - 5 Joinpoints | 5 | 2014 | 2017 | -0.02 | -0.12 | 0.09 | 0.724 |
| "10-14 years" / Male - 5 Joinpoints | 6 | 2017 | 2019 | 0.83* | 0.73 | 0.93 | < 0.001 |
| "15-19 years" / Both - 5 Joinpoints | 1 | 1990 | 1992 | 0.13 | -0.04 | 0.31 | 0.128 |
| "15-19 years" / Both - 5 Joinpoints | 2 | 1992 | 2005 | 0.02* | 0.01 | 0.03 | 0.001 |
| "15-19 years" / Both - 5 Joinpoints | 3 | 2005 | 2009 | 0.54* | 0.46 | 0.62 | < 0.001 |
| "15-19 years" / Both - 5 Joinpoints | 4 | 2009 | 2014 | -0.22* | -0.27 | -0.17 | < 0.001 |
| "15-19 years" / Both - 5 Joinpoints | 5 | 2014 | 2017 | -0.03 | -0.2 | 0.14 | 0.716 |
| "15-19 years" / Both - 5 Joinpoints | 6 | 2017 | 2019 | -0.28* | -0.45 | -0.11 | 0.003 |
| "15-19 years" / Female - 4 Joinpoints | 1 | 1990 | 2002 | 0.02* | 0.01 | 0.02 | < 0.001 |
| "15-19 years" / Female - 4 Joinpoints | 2 | 2002 | 2006 | 0.22* | 0.17 | 0.28 | < 0.001 |
| "15-19 years" / Female - 4 Joinpoints | 3 | 2006 | 2009 | 0.80* | 0.69 | 0.9 | < 0.001 |
| "15-19 years" / Female - 4 Joinpoints | 4 | 2009 | 2017 | 0.02* | 0 | 0.03 | 0.022 |
| "15-19 years" / Female - 4 Joinpoints | 5 | 2017 | 2019 | -0.21* | -0.31 | -0.1 | 0.001 |
| "15-19 years" / Male - 3 Joinpoints | 1 | 1990 | 1995 | 0.11* | 0.06 | 0.15 | < 0.001 |
| "15-19 years" / Male - 3 Joinpoints | 2 | 1995 | 2006 | -0.03* | -0.05 | -0.02 | < 0.001 |
| "15-19 years" / Male - 3 Joinpoints | 3 | 2006 | 2009 | 0.48* | 0.29 | 0.68 | < 0.001 |
| "15-19 years" / Male - 3 Joinpoints | 4 | 2009 | 2019 | 0 | -0.02 | 0.01 | 0.741 |

APC, Annual percent change

**P*<0.05

## Supplementary Table 9. Annual percent change based on joinpoint models for prevalence cases of Chinese children and adolescents with AD in different age groups from 1990 to 2019

| **Cohort** | **Segment** | **Lower Endpoint** | **Upper Endpoint** | **APC** | **Lower CI** | **Upper CI** | ***P*** |
| --- | --- | --- | --- | --- | --- | --- | --- |
| "<5 years" / Both - 5 Joinpoints | 1 | 1990 | 1992 | -0.75 | -1.54 | 0.04 | 0.061 |
| "<5 years" / Both - 5 Joinpoints | 2 | 1992 | 2001 | -3.92* | -4.02 | -3.83 | < 0.001 |
| "<5 years" / Both - 5 Joinpoints | 3 | 2001 | 2008 | -1.82* | -1.98 | -1.66 | < 0.001 |
| "<5 years" / Both - 5 Joinpoints | 4 | 2008 | 2012 | -0.46 | -0.97 | 0.06 | 0.077 |
| "<5 years" / Both - 5 Joinpoints | 5 | 2012 | 2015 | 1.61* | 0.58 | 2.66 | 0.005 |
| "<5 years" / Both - 5 Joinpoints | 6 | 2015 | 2019 | 3.99* | 3.67 | 4.30 | < 0.001 |
| "<5 years" / Female - 5 Joinpoints | 1 | 1990 | 1992 | -1.00* | -1.95 | -0.05 | 0.041 |
| "<5 years" / Female - 5 Joinpoints | 2 | 1992 | 2001 | -4.20* | -4.31 | -4.09 | < 0.001 |
| "<5 years" / Female - 5 Joinpoints | 3 | 2001 | 2009 | -1.80* | -1.96 | -1.64 | < 0.001 |
| "<5 years" / Female - 5 Joinpoints | 4 | 2009 | 2012 | -0.02 | -1.28 | 1.25 | 0.969 |
| "<5 years" / Female - 5 Joinpoints | 5 | 2012 | 2015 | 1.84* | 0.58 | 3.12 | 0.007 |
| "<5 years" / Female - 5 Joinpoints | 6 | 2015 | 2019 | 4.10* | 3.71 | 4.48 | < 0.001 |
| "<5 years" / Male - 5 Joinpoints | 1 | 1990 | 1992 | -0.54 | -1.23 | 0.16 | 0.12 |
| "<5 years" / Male - 5 Joinpoints | 2 | 1992 | 2001 | -3.72* | -3.8 | -3.64 | < 0.001 |
| "<5 years" / Male - 5 Joinpoints | 3 | 2001 | 2008 | -1.78* | -1.92 | -1.64 | < 0.001 |
| "<5 years" / Male - 5 Joinpoints | 4 | 2008 | 2012 | -0.47* | -0.91 | -0.02 | 0.041 |
| "<5 years" / Male - 5 Joinpoints | 5 | 2012 | 2015 | 1.35* | 0.45 | 2.25 | 0.006 |
| "<5 years" / Male - 5 Joinpoints | 6 | 2015 | 2019 | 3.92* | 3.64 | 4.2 | < 0.001 |
| "5-9 years" / Both - 4 Joinpoints | 1 | 1990 | 1996 | 1.17* | 1.02 | 1.32 | < 0.001 |
| "5-9 years" / Both - 4 Joinpoints | 2 | 1996 | 2002 | -5.21* | -5.41 | -5.02 | < 0.001 |
| "5-9 years" / Both - 4 Joinpoints | 3 | 2002 | 2007 | -2.45* | -2.77 | -2.14 | < 0.001 |
| "5-9 years" / Both - 4 Joinpoints | 4 | 2007 | 2016 | -0.87* | -0.99 | -0.76 | < 0.001 |
| "5-9 years" / Both - 4 Joinpoints | 5 | 2016 | 2019 | 2.74* | 2.19 | 3.3 | < 0.001 |
| "5-9 years" / Female - 4 Joinpoints | 1 | 1990 | 1996 | 0.89* | 0.73 | 1.04 | < 0.001 |
| "5-9 years" / Female - 4 Joinpoints | 2 | 1996 | 2002 | -5.56* | -5.77 | -5.36 | < 0.001 |
| "5-9 years" / Female - 4 Joinpoints | 3 | 2002 | 2007 | -2.54* | -2.88 | -2.19 | < 0.001 |
| "5-9 years" / Female - 4 Joinpoints | 4 | 2007 | 2016 | -0.94* | -1.07 | -0.82 | < 0.001 |
| "5-9 years" / Female - 4 Joinpoints | 5 | 2016 | 2019 | 3.02* | 2.42 | 3.62 | < 0.001 |
| "5-9 years" / Male - 4 Joinpoints | 1 | 1990 | 1996 | 1.43* | 1.29 | 1.58 | < 0.001 |
| "5-9 years" / Male - 4 Joinpoints | 2 | 1996 | 2002 | -4.91* | -5.1 | -4.72 | < 0.001 |
| "5-9 years" / Male - 4 Joinpoints | 3 | 2002 | 2007 | -2.38* | -2.69 | -2.08 | < 0.001 |
| "5-9 years" / Male - 4 Joinpoints | 4 | 2007 | 2016 | -0.82* | -0.93 | -0.7 | < 0.001 |
| "5-9 years" / Male - 4 Joinpoints | 5 | 2016 | 2019 | 2.51* | 1.98 | 3.04 | < 0.001 |
| "10-14 years" / Both - 5 Joinpoints | 1 | 1990 | 1994 | -0.39 | -1.1 | 0.32 | 0.258 |
| "10-14 years" / Both - 5 Joinpoints | 2 | 1994 | 2000 | 4.12* | 3.63 | 4.63 | < 0.001 |
| "10-14 years" / Both - 5 Joinpoints | 3 | 2000 | 2003 | -4.11* | -6.08 | -2.1 | 0.001 |
| "10-14 years" / Both - 5 Joinpoints | 4 | 2003 | 2007 | -6.99* | -8.06 | -5.91 | < 0.001 |
| "10-14 years" / Both - 5 Joinpoints | 5 | 2007 | 2011 | -3.93* | -5.17 | -2.67 | < 0.001 |
| "10-14 years" / Both - 5 Joinpoints | 6 | 2011 | 2019 | -0.43* | -0.72 | -0.13 | 0.008 |
| "10-14 years" / Female - 5 Joinpoints | 1 | 1990 | 1994 | -0.44 | -1.13 | 0.26 | 0.199 |
| "10-14 years" / Female - 5 Joinpoints | 2 | 1994 | 2000 | 3.99* | 3.5 | 4.48 | < 0.001 |
| "10-14 years" / Female - 5 Joinpoints | 3 | 2000 | 2003 | -4.34* | -6.26 | -2.38 | < 0.001 |
| "10-14 years" / Female - 5 Joinpoints | 4 | 2003 | 2007 | -7.38* | -8.43 | -6.32 | < 0.001 |
| "10-14 years" / Female - 5 Joinpoints | 5 | 2007 | 2011 | -4.08* | -5.3 | -2.83 | < 0.001 |
| "10-14 years" / Female - 5 Joinpoints | 6 | 2011 | 2019 | -0.54* | -0.83 | -0.25 | 0.002 |
| "10-14 years" / Male - 5 Joinpoints | 1 | 1990 | 1994 | -0.55 | -1.26 | 0.18 | 0.126 |
| "10-14 years" / Male - 5 Joinpoints | 2 | 1994 | 1999 | 4.77* | 4.05 | 5.49 | < 0.001 |
| "10-14 years" / Male - 5 Joinpoints | 3 | 1999 | 2002 | -1.13 | -3.14 | 0.92 | 0.251 |
| "10-14 years" / Male - 5 Joinpoints | 4 | 2002 | 2008 | -6.20* | -6.68 | -5.72 | < 0.001 |
| "10-14 years" / Male - 5 Joinpoints | 5 | 2008 | 2011 | -3.19* | -5.67 | -0.65 | 0.018 |
| "10-14 years" / Male - 5 Joinpoints | 6 | 2011 | 2019 | -0.37* | -0.66 | -0.08 | 0.017 |
| "15-19 years" / Both - 5 Joinpoints | 1 | 1990 | 1995 | -4.80* | -5.17 | -4.42 | < 0.001 |
| "15-19 years" / Both - 5 Joinpoints | 2 | 1995 | 1999 | 0.47 | -0.46 | 1.42 | 0.298 |
| "15-19 years" / Both - 5 Joinpoints | 3 | 1999 | 2004 | 5.42* | 4.84 | 6 | < 0.001 |
| "15-19 years" / Both - 5 Joinpoints | 4 | 2004 | 2007 | -1.13 | -2.73 | 0.49 | 0.155 |
| "15-19 years" / Both - 5 Joinpoints | 5 | 2007 | 2014 | -6.52* | -6.81 | -6.22 | < 0.001 |
| "15-19 years" / Both - 5 Joinpoints | 6 | 2014 | 2019 | -1.14* | -1.61 | -0.67 | < 0.001 |
| "15-19 years" / Female - 5 Joinpoints | 1 | 1990 | 1995 | -4.71* | -5.07 | -4.35 | < 0.001 |
| "15-19 years" / Female - 5 Joinpoints | 2 | 1995 | 1999 | 0.44 | -0.45 | 1.35 | 0.305 |
| "15-19 years" / Female - 5 Joinpoints | 3 | 1999 | 2004 | 5.39* | 4.84 | 5.95 | < 0.001 |
| "15-19 years" / Female - 5 Joinpoints | 4 | 2004 | 2007 | -1.02 | -2.56 | 0.54 | 0.179 |
| "15-19 years" / Female - 5 Joinpoints | 5 | 2007 | 2014 | -6.80* | -7.08 | -6.52 | < 0.001 |
| "15-19 years" / Female - 5 Joinpoints | 6 | 2014 | 2019 | -1.33* | -1.79 | -0.88 | < 0.001 |
| "15-19 years" / Male - 5 Joinpoints | 1 | 1990 | 1995 | -4.93* | -5.33 | -4.52 | < 0.001 |
| "15-19 years" / Male - 5 Joinpoints | 2 | 1995 | 1999 | 0.48 | -0.53 | 1.51 | 0.321 |
| "15-19 years" / Male - 5 Joinpoints | 3 | 1999 | 2003 | 5.64* | 4.63 | 6.65 | < 0.001 |
| "15-19 years" / Male - 5 Joinpoints | 4 | 2003 | 2006 | 1.95* | 0.16 | 3.76 | 0.035 |
| "15-19 years" / Male - 5 Joinpoints | 5 | 2006 | 2014 | -5.87* | -6.12 | -5.62 | < 0.001 |
| "15-19 years" / Male - 5 Joinpoints | 6 | 2014 | 2019 | -0.98* | -1.48 | -0.47 | 0.001 |

APC, Annual percent change

**P*<0.05

## Supplementary Table 10. Annual percent change based on joinpoint models for prevalence rates of Chinese children and adolescents with AD in different age groups from 1990 to 2019

| **Cohort** | **Segment** | **Lower Endpoint** | **Upper Endpoint** | **APC** | **Lower CI** | **Upper CI** | ***P*** |
| --- | --- | --- | --- | --- | --- | --- | --- |
| "<5 years" / Both - 3 Joinpoints | 1 | 1990 | 1998 | -0.29* | -0.4 | -0.17 | < 0.001 |
| "<5 years" / Both - 3 Joinpoints | 2 | 1998 | 2005 | 0.42* | 0.24 | 0.6 | < 0.001 |
| "<5 years" / Both - 3 Joinpoints | 3 | 2005 | 2017 | -0.34* | -0.41 | -0.27 | < 0.001 |
| "<5 years" / Both - 3 Joinpoints | 4 | 2017 | 2019 | 3.43* | 2.32 | 4.56 | < 0.001 |
| "<5 years" / Female - 3 Joinpoints | 1 | 1990 | 1999 | -0.27* | -0.36 | -0.17 | < 0.001 |
| "<5 years" / Female - 3 Joinpoints | 2 | 1999 | 2005 | 0.62* | 0.38 | 0.85 | < 0.001 |
| "<5 years" / Female - 3 Joinpoints | 3 | 2005 | 2017 | -0.36* | -0.43 | -0.29 | < 0.001 |
| "<5 years" / Female - 3 Joinpoints | 4 | 2017 | 2019 | 3.40* | 2.38 | 4.43 | < 0.001 |
| "<5 years" / Male - 3 Joinpoints | 1 | 1990 | 1997 | -0.32* | -0.46 | -0.18 | < 0.001 |
| "<5 years" / Male - 3 Joinpoints | 2 | 1997 | 2006 | 0.21* | 0.1 | 0.33 | 0.001 |
| "<5 years" / Male - 3 Joinpoints | 3 | 2006 | 2017 | -0.34* | -0.42 | -0.26 | < 0.001 |
| "<5 years" / Male - 3 Joinpoints | 4 | 2017 | 2019 | 3.55* | 2.47 | 4.65 | < 0.001 |
| "5-9 years" / Both - 5 Joinpoints | 1 | 1990 | 1994 | -0.98* | -1.02 | -0.94 | < 0.001 |
| "5-9 years" / Both - 5 Joinpoints | 2 | 1994 | 2001 | -0.07* | -0.09 | -0.05 | < 0.001 |
| "5-9 years" / Both - 5 Joinpoints | 3 | 2001 | 2005 | 0.42* | 0.36 | 0.48 | < 0.001 |
| "5-9 years" / Both - 5 Joinpoints | 4 | 2005 | 2009 | -0.33* | -0.39 | -0.27 | < 0.001 |
| "5-9 years" / Both - 5 Joinpoints | 5 | 2009 | 2017 | 0.02* | 0.01 | 0.04 | 0.01 |
| "5-9 years" / Both - 5 Joinpoints | 6 | 2017 | 2019 | 1.94* | 1.82 | 2.06 | < 0.001 |
| "5-9 years" / Female - 5 Joinpoints | 1 | 1990 | 1994 | -1.00* | -1.04 | -0.96 | < 0.001 |
| "5-9 years" / Female - 5 Joinpoints | 2 | 1994 | 2001 | -0.07* | -0.09 | -0.05 | < 0.001 |
| "5-9 years" / Female - 5 Joinpoints | 3 | 2001 | 2005 | 0.54* | 0.48 | 0.6 | < 0.001 |
| "5-9 years" / Female - 5 Joinpoints | 4 | 2005 | 2009 | -0.38* | -0.44 | -0.32 | < 0.001 |
| "5-9 years" / Female - 5 Joinpoints | 5 | 2009 | 2017 | 0.03* | 0.01 | 0.04 | 0.003 |
| "5-9 years" / Female - 5 Joinpoints | 6 | 2017 | 2019 | 1.84* | 1.72 | 1.96 | < 0.001 |
| "5-9 years" / Male - 5 Joinpoints | 1 | 1990 | 1994 | -0.96* | -0.99 | -0.92 | < 0.001 |
| "5-9 years" / Male - 5 Joinpoints | 2 | 1994 | 2001 | -0.07* | -0.09 | -0.05 | < 0.001 |
| "5-9 years" / Male - 5 Joinpoints | 3 | 2001 | 2005 | 0.32* | 0.27 | 0.37 | < 0.001 |
| "5-9 years" / Male - 5 Joinpoints | 4 | 2005 | 2009 | -0.29* | -0.35 | -0.24 | < 0.001 |
| "5-9 years" / Male - 5 Joinpoints | 5 | 2009 | 2017 | 0.01 | 0 | 0.03 | 0.089 |
| "5-9 years" / Male - 5 Joinpoints | 6 | 2017 | 2019 | 2.02* | 1.9 | 2.14 | < 0.001 |
| "10-14 years" / Both - 4 Joinpoints | 1 | 1990 | 1994 | -0.86* | -0.92 | -0.8 | < 0.001 |
| "10-14 years" / Both - 4 Joinpoints | 2 | 1994 | 2005 | 0 | -0.01 | 0.02 | 0.828 |
| "10-14 years" / Both - 4 Joinpoints | 3 | 2005 | 2009 | -0.37* | -0.47 | -0.28 | < 0.001 |
| "10-14 years" / Both - 4 Joinpoints | 4 | 2009 | 2017 | 0.06* | 0.03 | 0.08 | < 0.001 |
| "10-14 years" / Both - 4 Joinpoints | 5 | 2017 | 2019 | 2.05* | 1.85 | 2.25 | < 0.001 |
| "10-14 years" / Female - 4 Joinpoints | 1 | 1990 | 1994 | -0.81* | -0.87 | -0.75 | < 0.001 |
| "10-14 years" / Female - 4 Joinpoints | 2 | 1994 | 2006 | 0.01 | -0.01 | 0.02 | 0.445 |
| "10-14 years" / Female - 4 Joinpoints | 3 | 2006 | 2009 | -0.36* | -0.55 | -0.17 | 0.001 |
| "10-14 years" / Female - 4 Joinpoints | 4 | 2009 | 2017 | 0.08* | 0.06 | 0.11 | < 0.001 |
| "10-14 years" / Female - 4 Joinpoints | 5 | 2017 | 2019 | 1.99* | 1.8 | 2.19 | < 0.001 |
| "10-14 years" / Male - 4 Joinpoints | 1 | 1990 | 1994 | -0.88* | -0.97 | -0.79 | < 0.001 |
| "10-14 years" / Male - 4 Joinpoints | 2 | 1994 | 2006 | 0.02 | 0 | 0.04 | 0.071 |
| "10-14 years" / Male - 4 Joinpoints | 3 | 2006 | 2009 | -0.49* | -0.76 | -0.23 | 0.001 |
| "10-14 years" / Male - 4 Joinpoints | 4 | 2009 | 2017 | 0.07* | 0.03 | 0.11 | 0.002 |
| "10-14 years" / Male - 4 Joinpoints | 5 | 2017 | 2019 | 2.12* | 1.83 | 2.4 | < 0.001 |
| "15-19 years" / Both - 5 Joinpoints | 1 | 1990 | 1994 | -0.37* | -0.39 | -0.34 | < 0.001 |
| "15-19 years" / Both - 5 Joinpoints | 2 | 1994 | 2002 | 0.03* | 0.02 | 0.05 | < 0.001 |
| "15-19 years" / Both - 5 Joinpoints | 3 | 2002 | 2009 | 0.11* | 0.1 | 0.12 | < 0.001 |
| "15-19 years" / Both - 5 Joinpoints | 4 | 2009 | 2013 | -0.20* | -0.25 | -0.16 | < 0.001 |
| "15-19 years" / Both - 5 Joinpoints | 5 | 2013 | 2017 | -0.01 | -0.06 | 0.03 | 0.477 |
| "15-19 years" / Both - 5 Joinpoints | 6 | 2017 | 2019 | 1.01* | 0.92 | 1.09 | < 0.001 |
| "15-19 years" / Female - 5 Joinpoints | 1 | 1990 | 1994 | -0.31* | -0.34 | -0.28 | < 0.001 |
| "15-19 years" / Female - 5 Joinpoints | 2 | 1994 | 2005 | 0.05* | 0.04 | 0.06 | < 0.001 |
| "15-19 years" / Female - 5 Joinpoints | 3 | 2005 | 2009 | 0.32* | 0.28 | 0.36 | < 0.001 |
| "15-19 years" / Female - 5 Joinpoints | 4 | 2009 | 2013 | -0.05* | -0.1 | -0.01 | 0.021 |
| "15-19 years" / Female - 5 Joinpoints | 5 | 2013 | 2017 | 0.05* | 0 | 0.09 | 0.034 |
| "15-19 years" / Female - 5 Joinpoints | 6 | 2017 | 2019 | 0.98* | 0.9 | 1.07 | < 0.001 |
| "15-19 years" / Male - 4 Joinpoints | 1 | 1990 | 1994 | -0.51* | -0.55 | -0.47 | < 0.001 |
| "15-19 years" / Male - 4 Joinpoints | 2 | 1994 | 2007 | 0.05* | 0.05 | 0.06 | < 0.001 |
| "15-19 years" / Male - 4 Joinpoints | 3 | 2007 | 2013 | -0.09* | -0.12 | -0.06 | < 0.001 |
| "15-19 years" / Male - 4 Joinpoints | 4 | 2013 | 2017 | 0.05 | -0.02 | 0.12 | 0.17 |
| "15-19 years" / Male - 4 Joinpoints | 5 | 2017 | 2019 | 1.13* | 0.99 | 1.26 | < 0.001 |

APC, Annual percent change

**P*<0.05

## Supplementary Table 11. Annual percent change based on joinpoint models for DALY cases of Chinese children and adolescents with AD in different age groups from 1990 to 2019

| **Cohort** | **Segment** | **Lower Endpoint** | **Upper Endpoint** | **APC** | **Lower CI** | **Upper CI** | ***P*** |
| --- | --- | --- | --- | --- | --- | --- | --- |
| "<5 years" / Both - 5 Joinpoints | 1 | 1990 | 1992 | -0.73 | -1.52 | 0.06 | 0.068 |
| "<5 years" / Both - 5 Joinpoints | 2 | 1992 | 2001 | -3.90* | -4 | -3.81 | < 0.001 |
| "<5 years" / Both - 5 Joinpoints | 3 | 2001 | 2008 | -1.81* | -1.97 | -1.64 | < 0.001 |
| "<5 years" / Both - 5 Joinpoints | 4 | 2008 | 2012 | -0.46 | -0.97 | 0.05 | 0.075 |
| "<5 years" / Both - 5 Joinpoints | 5 | 2012 | 2015 | 1.60* | 0.57 | 2.64 | 0.005 |
| "<5 years" / Both - 5 Joinpoints | 6 | 2015 | 2019 | 3.99* | 3.68 | 4.31 | < 0.001 |
| "<5 years" / Female - 5 Joinpoints | 1 | 1990 | 1992 | -0.8 | -1.61 | 0.01 | 0.053 |
| "<5 years" / Female - 5 Joinpoints | 2 | 1992 | 2000 | -4.33* | -4.44 | -4.21 | < 0.001 |
| "<5 years" / Female - 5 Joinpoints | 3 | 2000 | 2008 | -2.08* | -2.22 | -1.95 | < 0.001 |
| "<5 years" / Female - 5 Joinpoints | 4 | 2008 | 2012 | -0.31 | -0.84 | 0.23 | 0.233 |
| "<5 years" / Female - 5 Joinpoints | 5 | 2012 | 2015 | 1.89* | 0.82 | 2.98 | 0.002 |
| "<5 years" / Female - 5 Joinpoints | 6 | 2015 | 2019 | 4.12* | 3.79 | 4.44 | < 0.001 |
| "<5 years" / Male - 5 Joinpoints | 1 | 1990 | 1992 | -0.49 | -1.15 | 0.17 | 0.13 |
| "<5 years" / Male - 5 Joinpoints | 2 | 1992 | 2001 | -3.71* | -3.78 | -3.63 | < 0.001 |
| "<5 years" / Male - 5 Joinpoints | 3 | 2001 | 2008 | -1.76* | -1.9 | -1.63 | < 0.001 |
| "<5 years" / Male - 5 Joinpoints | 4 | 2008 | 2012 | -0.55* | -0.97 | -0.13 | 0.014 |
| "<5 years" / Male - 5 Joinpoints | 5 | 2012 | 2016 | 1.79* | 1.37 | 2.22 | < 0.001 |
| "<5 years" / Male - 5 Joinpoints | 6 | 2016 | 2019 | 4.40* | 3.99 | 4.81 | < 0.001 |
| "5-9 years" / Both - 4 Joinpoints | 1 | 1990 | 1996 | 1.17* | 1.02 | 1.31 | < 0.001 |
| "5-9 years" / Both - 4 Joinpoints | 2 | 1996 | 2002 | -5.19* | -5.38 | -5 | < 0.001 |
| "5-9 years" / Both - 4 Joinpoints | 3 | 2002 | 2007 | -2.44* | -2.74 | -2.13 | < 0.001 |
| "5-9 years" / Both - 4 Joinpoints | 4 | 2007 | 2016 | -0.88* | -0.99 | -0.76 | < 0.001 |
| "5-9 years" / Both - 4 Joinpoints | 5 | 2016 | 2019 | 2.77* | 2.24 | 3.3 | < 0.001 |
| "5-9 years" / Female - 4 Joinpoints | 1 | 1990 | 1996 | 0.89* | 0.74 | 1.04 | < 0.001 |
| "5-9 years" / Female - 4 Joinpoints | 2 | 1996 | 2002 | -5.54* | -5.73 | -5.34 | < 0.001 |
| "5-9 years" / Female - 4 Joinpoints | 3 | 2002 | 2007 | -2.52* | -2.85 | -2.2 | < 0.001 |
| "5-9 years" / Female - 4 Joinpoints | 4 | 2007 | 2016 | -0.94* | -1.06 | -0.82 | < 0.001 |
| "5-9 years" / Female - 4 Joinpoints | 5 | 2016 | 2019 | 3.03* | 2.46 | 3.6 | < 0.001 |
| "5-9 years" / Male - 4 Joinpoints | 1 | 1990 | 1996 | 1.42* | 1.28 | 1.56 | < 0.001 |
| "5-9 years" / Male - 4 Joinpoints | 2 | 1996 | 2002 | -4.88* | -5.07 | -4.7 | < 0.001 |
| "5-9 years" / Male - 4 Joinpoints | 3 | 2002 | 2007 | -2.37* | -2.66 | -2.07 | < 0.001 |
| "5-9 years" / Male - 4 Joinpoints | 4 | 2007 | 2016 | -0.82* | -0.93 | -0.71 | < 0.001 |
| "5-9 years" / Male - 4 Joinpoints | 5 | 2016 | 2019 | 2.54* | 2.03 | 3.06 | < 0.001 |
| "10-14 years" / Both - 5 Joinpoints | 1 | 1990 | 1994 | -0.37 | -1.1 | 0.36 | 0.292 |
| "10-14 years" / Both - 5 Joinpoints | 2 | 1994 | 2000 | 4.14* | 3.63 | 4.65 | < 0.001 |
| "10-14 years" / Both - 5 Joinpoints | 3 | 2000 | 2003 | -4.23* | -6.25 | -2.17 | 0.001 |
| "10-14 years" / Both - 5 Joinpoints | 4 | 2003 | 2008 | -6.61* | -7.32 | -5.89 | < 0.001 |
| "10-14 years" / Both - 5 Joinpoints | 5 | 2008 | 2011 | -3.27* | -5.84 | -0.63 | 0.019 |
| "10-14 years" / Both - 5 Joinpoints | 6 | 2011 | 2019 | -0.50* | -0.81 | -0.2 | 0.003 |
| "10-14 years" / Female - 5 Joinpoints | 1 | 1990 | 1994 | -0.4 | -1.12 | 0.32 | 0.251 |
| "10-14 years" / Female - 5 Joinpoints | 2 | 1994 | 2000 | 3.99* | 3.48 | 4.49 | < 0.001 |
| "10-14 years" / Female - 5 Joinpoints | 3 | 2000 | 2003 | -4.32* | -6.32 | -2.28 | 0.001 |
| "10-14 years" / Female - 5 Joinpoints | 4 | 2003 | 2007 | -7.37* | -8.46 | -6.27 | < 0.001 |
| "10-14 years" / Female - 5 Joinpoints | 5 | 2007 | 2011 | -4.05* | -5.32 | -2.76 | < 0.001 |
| "10-14 years" / Female - 5 Joinpoints | 6 | 2011 | 2019 | -0.56* | -0.86 | -0.26 | 0.002 |
| "10-14 years" / Male - 5 Joinpoints | 1 | 1990 | 1993 | -1.57* | -2.69 | -0.43 | 0.011 |
| "10-14 years" / Male - 5 Joinpoints | 2 | 1993 | 2000 | 3.94* | 3.56 | 4.33 | < 0.001 |
| "10-14 years" / Male - 5 Joinpoints | 3 | 2000 | 2003 | -3.76* | -5.76 | -1.73 | 0.002 |
| "10-14 years" / Male - 5 Joinpoints | 4 | 2003 | 2008 | -6.24* | -6.94 | -5.54 | < 0.001 |
| "10-14 years" / Male - 5 Joinpoints | 5 | 2008 | 2011 | -3.16* | -5.65 | -0.6 | 0.02 |
| "10-14 years" / Male - 5 Joinpoints | 6 | 2011 | 2019 | -0.38* | -0.67 | -0.08 | 0.016 |
| "15-19 years" / Both - 5 Joinpoints | 1 | 1990 | 1995 | -4.79* | -5.16 | -4.42 | < 0.001 |
| "15-19 years" / Both - 5 Joinpoints | 2 | 1995 | 1999 | 0.48 | -0.46 | 1.42 | 0.293 |
| "15-19 years" / Both - 5 Joinpoints | 3 | 1999 | 2004 | 5.44* | 4.86 | 6.03 | < 0.001 |
| "15-19 years" / Both - 5 Joinpoints | 4 | 2004 | 2007 | -1.16 | -2.76 | 0.46 | 0.145 |
| "15-19 years" / Both - 5 Joinpoints | 5 | 2007 | 2014 | -6.49* | -6.78 | -6.19 | < 0.001 |
| "15-19 years" / Both - 5 Joinpoints | 6 | 2014 | 2019 | -1.16* | -1.63 | -0.69 | < 0.001 |
| "15-19 years" / Female - 5 Joinpoints | 1 | 1990 | 1995 | -4.68* | -5.03 | -4.33 | < 0.001 |
| "15-19 years" / Female - 5 Joinpoints | 2 | 1995 | 1999 | 0.45 | -0.43 | 1.33 | 0.293 |
| "15-19 years" / Female - 5 Joinpoints | 3 | 1999 | 2004 | 5.41* | 4.87 | 5.96 | < 0.001 |
| "15-19 years" / Female - 5 Joinpoints | 4 | 2004 | 2007 | -1.03 | -2.53 | 0.49 | 0.166 |
| "15-19 years" / Female - 5 Joinpoints | 5 | 2007 | 2014 | -6.78* | -7.05 | -6.5 | < 0.001 |
| "15-19 years" / Female - 5 Joinpoints | 6 | 2014 | 2019 | -1.35* | -1.79 | -0.9 | < 0.001 |
| "15-19 years" / Male - 5 Joinpoints | 1 | 1990 | 1995 | -4.95* | -5.37 | -4.54 | < 0.001 |
| "15-19 years" / Male - 5 Joinpoints | 2 | 1995 | 1999 | 0.48 | -0.56 | 1.54 | 0.335 |
| "15-19 years" / Male - 5 Joinpoints | 3 | 1999 | 2003 | 5.70* | 4.66 | 6.74 | < 0.001 |
| "15-19 years" / Male - 5 Joinpoints | 4 | 2003 | 2006 | 1.87* | 0.04 | 3.74 | 0.046 |
| "15-19 years" / Male - 5 Joinpoints | 5 | 2006 | 2014 | -5.85* | -6.11 | -5.59 | < 0.001 |
| "15-19 years" / Male - 5 Joinpoints | 6 | 2014 | 2019 | -0.98* | -1.5 | -0.47 | 0.001 |

APC, Annual percent change

**P*<0.05

## Supplementary Table 12. Annual percent change based on joinpoint models for DALY rates of Chinese children and adolescents with AD in different age groups from 1990 to 2019

| **Cohort** | **Segment** | **Lower Endpoint** | **Upper Endpoint** | **APC** | **Lower CI** | **Upper CI** | ***P*** |
| --- | --- | --- | --- | --- | --- | --- | --- |
| "<5 years" / Both - 3 Joinpoints | 1 | 1990 | 1998 | -0.27* | -0.38 | -0.15 | < 0.001 |
| "<5 years" / Both - 3 Joinpoints | 2 | 1998 | 2005 | 0.44* | 0.26 | 0.62 | < 0.001 |
| "<5 years" / Both - 3 Joinpoints | 3 | 2005 | 2017 | -0.34* | -0.41 | -0.27 | < 0.001 |
| "<5 years" / Both - 3 Joinpoints | 4 | 2017 | 2019 | 3.45* | 2.38 | 4.53 | < 0.001 |
| "<5 years" / Female - 3 Joinpoints | 1 | 1990 | 1998 | -0.32* | -0.43 | -0.2 | < 0.001 |
| "<5 years" / Female - 3 Joinpoints | 2 | 1998 | 2005 | 0.56* | 0.38 | 0.74 | < 0.001 |
| "<5 years" / Female - 3 Joinpoints | 3 | 2005 | 2017 | -0.36* | -0.43 | -0.29 | < 0.001 |
| "<5 years" / Female - 3 Joinpoints | 4 | 2017 | 2019 | 3.44* | 2.35 | 4.55 | < 0.001 |
| "<5 years" / Male - 3 Joinpoints | 1 | 1990 | 1998 | -0.26* | -0.38 | -0.15 | < 0.001 |
| "<5 years" / Male - 3 Joinpoints | 2 | 1998 | 2005 | 0.33* | 0.15 | 0.51 | 0.001 |
| "<5 years" / Male - 3 Joinpoints | 3 | 2005 | 2017 | -0.32* | -0.39 | -0.25 | < 0.001 |
| "<5 years" / Male - 3 Joinpoints | 4 | 2017 | 2019 | 3.49* | 2.39 | 4.6 | < 0.001 |
| "5-9 years" / Both - 5 Joinpoints | 1 | 1990 | 1994 | -0.97* | -1.01 | -0.92 | < 0.001 |
| "5-9 years" / Both - 5 Joinpoints | 2 | 1994 | 2000 | -0.09* | -0.12 | -0.06 | < 0.001 |
| "5-9 years" / Both - 5 Joinpoints | 3 | 2000 | 2005 | 0.37* | 0.33 | 0.41 | < 0.001 |
| "5-9 years" / Both - 5 Joinpoints | 4 | 2005 | 2009 | -0.31* | -0.37 | -0.24 | < 0.001 |
| "5-9 years" / Both - 5 Joinpoints | 5 | 2009 | 2017 | 0.02 | 0 | 0.03 | 0.062 |
| "5-9 years" / Both - 5 Joinpoints | 6 | 2017 | 2019 | 2.00* | 1.87 | 2.14 | < 0.001 |
| "5-9 years" / Female - 5 Joinpoints | 1 | 1990 | 1994 | -0.97* | -1.02 | -0.91 | < 0.001 |
| "5-9 years" / Female - 5 Joinpoints | 2 | 1994 | 2000 | -0.10* | -0.14 | -0.06 | < 0.001 |
| "5-9 years" / Female - 5 Joinpoints | 3 | 2000 | 2005 | 0.48* | 0.42 | 0.53 | < 0.001 |
| "5-9 years" / Female - 5 Joinpoints | 4 | 2005 | 2009 | -0.35* | -0.44 | -0.27 | < 0.001 |
| "5-9 years" / Female - 5 Joinpoints | 5 | 2009 | 2017 | 0.03* | 0.01 | 0.05 | 0.021 |
| "5-9 years" / Female - 5 Joinpoints | 6 | 2017 | 2019 | 1.88* | 1.7 | 2.06 | < 0.001 |
| "5-9 years" / Male - 5 Joinpoints | 1 | 1990 | 1993 | -1.21* | -1.3 | -1.11 | < 0.001 |
| "5-9 years" / Male - 5 Joinpoints | 2 | 1993 | 2000 | -0.15* | -0.18 | -0.12 | < 0.001 |
| "5-9 years" / Male - 5 Joinpoints | 3 | 2000 | 2005 | 0.30* | 0.25 | 0.36 | < 0.001 |
| "5-9 years" / Male - 5 Joinpoints | 4 | 2005 | 2009 | -0.28* | -0.37 | -0.18 | < 0.001 |
| "5-9 years" / Male - 5 Joinpoints | 5 | 2009 | 2017 | 0.01 | -0.02 | 0.03 | 0.487 |
| "5-9 years" / Male - 5 Joinpoints | 6 | 2017 | 2019 | 2.10* | 1.91 | 2.28 | < 0.001 |
| "10-14 years" / Both - 4 Joinpoints | 1 | 1990 | 1994 | -0.84* | -0.91 | -0.76 | < 0.001 |
| "10-14 years" / Both - 4 Joinpoints | 2 | 1994 | 2005 | 0.01 | -0.01 | 0.03 | 0.361 |
| "10-14 years" / Both - 4 Joinpoints | 3 | 2005 | 2009 | -0.34* | -0.45 | -0.22 | < 0.001 |
| "10-14 years" / Both - 4 Joinpoints | 4 | 2009 | 2017 | 0.05* | 0.02 | 0.08 | 0.006 |
| "10-14 years" / Both - 4 Joinpoints | 5 | 2017 | 2019 | 2.08* | 1.85 | 2.32 | < 0.001 |
| "10-14 years" / Female - 4 Joinpoints | 1 | 1990 | 1994 | -0.79* | -0.87 | -0.72 | < 0.001 |
| "10-14 years" / Female - 4 Joinpoints | 2 | 1994 | 2005 | 0.02* | 0 | 0.04 | 0.029 |
| "10-14 years" / Female - 4 Joinpoints | 3 | 2005 | 2009 | -0.24* | -0.35 | -0.12 | < 0.001 |
| "10-14 years" / Female - 4 Joinpoints | 4 | 2009 | 2017 | 0.06* | 0.02 | 0.09 | 0.001 |
| "10-14 years" / Female - 4 Joinpoints | 5 | 2017 | 2019 | 2.08* | 1.86 | 2.31 | < 0.001 |
| "10-14 years" / Male - 4 Joinpoints | 1 | 1990 | 1994 | -0.87* | -0.99 | -0.76 | < 0.001 |
| "10-14 years" / Male - 4 Joinpoints | 2 | 1994 | 2006 | 0.03* | 0 | 0.05 | 0.029 |
| "10-14 years" / Male - 4 Joinpoints | 3 | 2006 | 2009 | -0.47* | -0.84 | -0.1 | 0.017 |
| "10-14 years" / Male - 4 Joinpoints | 4 | 2009 | 2017 | 0.07* | 0.02 | 0.12 | 0.008 |
| "10-14 years" / Male - 4 Joinpoints | 5 | 2017 | 2019 | 2.09* | 1.71 | 2.47 | < 0.001 |
| "15-19 years" / Both - 5 Joinpoints | 1 | 1990 | 1994 | -0.36* | -0.41 | -0.31 | < 0.001 |
| "15-19 years" / Both - 5 Joinpoints | 2 | 1994 | 2000 | 0.03 | 0 | 0.07 | 0.06 |
| "15-19 years" / Both - 5 Joinpoints | 3 | 2000 | 2009 | 0.10* | 0.08 | 0.12 | < 0.001 |
| "15-19 years" / Both - 5 Joinpoints | 4 | 2009 | 2014 | -0.15* | -0.2 | -0.09 | < 0.001 |
| "15-19 years" / Both - 5 Joinpoints | 5 | 2014 | 2017 | 0.01 | -0.15 | 0.17 | 0.908 |
| "15-19 years" / Both - 5 Joinpoints | 6 | 2017 | 2019 | 0.97* | 0.81 | 1.14 | < 0.001 |
| "15-19 years" / Female - 4 Joinpoints | 1 | 1990 | 1994 | -0.27* | -0.35 | -0.2 | < 0.001 |
| "15-19 years" / Female - 4 Joinpoints | 2 | 1994 | 2005 | 0.06* | 0.04 | 0.08 | < 0.001 |
| "15-19 years" / Female - 4 Joinpoints | 3 | 2005 | 2009 | 0.31* | 0.19 | 0.44 | < 0.001 |
| "15-19 years" / Female - 4 Joinpoints | 4 | 2009 | 2017 | 0.01 | -0.02 | 0.04 | 0.503 |
| "15-19 years" / Female - 4 Joinpoints | 5 | 2017 | 2019 | 0.97* | 0.72 | 1.23 | < 0.001 |
| "15-19 years" / Male - 5 Joinpoints | 1 | 1990 | 1993 | -0.59* | -0.68 | -0.5 | < 0.001 |
| "15-19 years" / Male - 5 Joinpoints | 2 | 1993 | 1996 | -0.17 | -0.34 | 0.01 | 0.06 |
| "15-19 years" / Male - 5 Joinpoints | 3 | 1996 | 2004 | 0.10* | 0.08 | 0.12 | < 0.001 |
| "15-19 years" / Male - 5 Joinpoints | 4 | 2004 | 2014 | -0.04* | -0.06 | -0.02 | < 0.001 |
| "15-19 years" / Male - 5 Joinpoints | 5 | 2014 | 2017 | 0.04 | -0.13 | 0.22 | 0.603 |
| "15-19 years" / Male - 5 Joinpoints | 6 | 2017 | 2019 | 1.16* | 0.98 | 1.34 | < 0.001 |

APC, Annual percent change

**P*<0.05

## Supplementary Table 13. Predicted incidence cases from 2020 to 2030 based on BAPC model

| **Age group** | **Year** | **Sex** | **Value** | **Upper** | **Lower** |
| --- | --- | --- | --- | --- | --- |
| <5 years | 2020 | Male | 339503.6 | 352502.3 | 326504.9 |
| <5 years | 2021 | Male | 333861.4 | 351994.2 | 315728.6 |
| <5 years | 2022 | Male | 318012.4 | 340179 | 295845.8 |
| <5 years | 2023 | Male | 310592.2 | 336471.6 | 284712.7 |
| <5 years | 2024 | Male | 304351.2 | 333673.1 | 275029.2 |
| <5 years | 2025 | Male | 298660.1 | 331445 | 265875.2 |
| <5 years | 2026 | Male | 293252.4 | 329666.3 | 256838.5 |
| <5 years | 2027 | Male | 288052.8 | 328319.6 | 247785.9 |
| <5 years | 2028 | Male | 283096.4 | 327458.7 | 238734.2 |
| <5 years | 2029 | Male | 278550.7 | 327271.9 | 229829.5 |
| <5 years | 2030 | Male | 274560 | 327925 | 221194.9 |
| <5 years | 2020 | Female | 273801 | 284226.9 | 263375.1 |
| <5 years | 2021 | Female | 268717.4 | 282574.2 | 254860.7 |
| <5 years | 2022 | Female | 255304.9 | 272175.2 | 238434.6 |
| <5 years | 2023 | Female | 248787.5 | 268671.7 | 228903.4 |
| <5 years | 2024 | Female | 243243.4 | 265972.5 | 220514.3 |
| <5 years | 2025 | Female | 238159.3 | 263701.1 | 212617.6 |
| <5 years | 2026 | Female | 233320.4 | 261734 | 204906.8 |
| <5 years | 2027 | Female | 228667.8 | 260061.8 | 197273.7 |
| <5 years | 2028 | Female | 224226.7 | 258735.2 | 189718.2 |
| <5 years | 2029 | Female | 220130 | 257914.4 | 182345.6 |
| <5 years | 2030 | Female | 216485.1 | 257730.7 | 175239.5 |
| <5 years | 2020 | Both | 613304.7 | 636729.3 | 589880.1 |
| <5 years | 2021 | Both | 602578.9 | 634568.4 | 570589.3 |
| <5 years | 2022 | Both | 573317.3 | 612354.2 | 534280.4 |
| <5 years | 2023 | Both | 559379.7 | 605143.3 | 513616.1 |
| <5 years | 2024 | Both | 547594.5 | 599645.6 | 495543.5 |
| <5 years | 2025 | Both | 536819.4 | 595146.1 | 478492.7 |
| <5 years | 2026 | Both | 526572.8 | 591400.4 | 461745.2 |
| <5 years | 2027 | Both | 516720.6 | 588381.4 | 445059.7 |
| <5 years | 2028 | Both | 507323.1 | 586193.9 | 428452.3 |
| <5 years | 2029 | Both | 498680.7 | 585186.2 | 412175.1 |
| <5 years | 2030 | Both | 491045 | 585655.7 | 396434.4 |
| 5-9 years | 2020 | Male | 111225.8 | 120460.1 | 101991.4 |
| 5-9 years | 2021 | Male | 114643.8 | 130553.6 | 98733.96 |
| 5-9 years | 2022 | Male | 120933.2 | 152855.5 | 89010.94 |
| 5-9 years | 2023 | Male | 123969.5 | 171156.8 | 76782.21 |
| 5-9 years | 2024 | Male | 126019.7 | 181106.4 | 70932.96 |
| 5-9 years | 2025 | Male | 126325.9 | 183033.5 | 69618.33 |
| 5-9 years | 2026 | Male | 124251.9 | 179050.6 | 69453.21 |
| 5-9 years | 2027 | Male | 118373.2 | 168886.1 | 67860.37 |
| 5-9 years | 2028 | Male | 115639.5 | 163258.7 | 68020.36 |
| 5-9 years | 2029 | Male | 113345.2 | 158526.5 | 68163.99 |
| 5-9 years | 2030 | Male | 111262.5 | 154449.8 | 68075.11 |
| 5-9 years | 2020 | Female | 123251.1 | 130749.4 | 115752.8 |
| 5-9 years | 2021 | Female | 127575.4 | 140133.1 | 115017.8 |
| 5-9 years | 2022 | Female | 134954.5 | 159817.5 | 110091.5 |
| 5-9 years | 2023 | Female | 138357.5 | 175342.7 | 101372.4 |
| 5-9 years | 2024 | Female | 140458.8 | 184632 | 96285.6 |
| 5-9 years | 2025 | Female | 140541.4 | 187184.8 | 93898.02 |
| 5-9 years | 2026 | Female | 137955.9 | 184037.7 | 91874.14 |
| 5-9 years | 2027 | Female | 131086.6 | 174341 | 87832.24 |
| 5-9 years | 2028 | Female | 127767.4 | 169204.7 | 86330.04 |
| 5-9 years | 2029 | Female | 124947.7 | 164851 | 85044.42 |
| 5-9 years | 2030 | Female | 122372.9 | 161068.7 | 83677.09 |
| 5-9 years | 2020 | Both | 234476.9 | 251209.5 | 217744.3 |
| 5-9 years | 2021 | Both | 242219.2 | 270686.7 | 213751.7 |
| 5-9 years | 2022 | Both | 255887.7 | 312673 | 199102.5 |
| 5-9 years | 2023 | Both | 262327 | 346499.5 | 178154.6 |
| 5-9 years | 2024 | Both | 266478.4 | 365738.3 | 167218.6 |
| 5-9 years | 2025 | Both | 266867.3 | 370218.3 | 163516.4 |
| 5-9 years | 2026 | Both | 262207.8 | 363088.3 | 161327.4 |
| 5-9 years | 2027 | Both | 249459.9 | 343227.1 | 155692.6 |
| 5-9 years | 2028 | Both | 243406.9 | 332463.4 | 154350.4 |
| 5-9 years | 2029 | Both | 238292.9 | 323377.5 | 153208.4 |
| 5-9 years | 2030 | Both | 233635.4 | 315518.5 | 151752.2 |
| 10-14 years | 2020 | Male | 69918.35 | 79349.59 | 60487.12 |
| 10-14 years | 2021 | Male | 69822.82 | 86761.56 | 52884.09 |
| 10-14 years | 2022 | Male | 70093.92 | 102100.9 | 38086.91 |
| 10-14 years | 2023 | Male | 70647.85 | 117282 | 24013.72 |
| 10-14 years | 2024 | Male | 71451.77 | 129047.5 | 13856.01 |
| 10-14 years | 2025 | Male | 72739.23 | 138343.6 | 7134.897 |
| 10-14 years | 2026 | Male | 74993.48 | 147264.5 | 2722.437 |
| 10-14 years | 2027 | Male | 79134.72 | 158426.7 | NA |
| 10-14 years | 2028 | Male | 81148.93 | 163613.5 | NA |
| 10-14 years | 2029 | Male | 82520.22 | 165645.4 | NA |
| 10-14 years | 2030 | Male | 82751.93 | 164073.4 | 1430.465 |
| 10-14 years | 2020 | Female | 106680 | 113924 | 99435.97 |
| 10-14 years | 2021 | Female | 106460.1 | 118719 | 94201.15 |
| 10-14 years | 2022 | Female | 106882.6 | 130016 | 83749.19 |
| 10-14 years | 2023 | Female | 107895.4 | 142304.9 | 73485.82 |
| 10-14 years | 2024 | Female | 109509.5 | 152777.2 | 66241.82 |
| 10-14 years | 2025 | Female | 112019.5 | 162024.8 | 62014.16 |
| 10-14 years | 2026 | Female | 115977.2 | 171686.2 | 60268.17 |
| 10-14 years | 2027 | Female | 122725.1 | 184275.8 | 61174.35 |
| 10-14 years | 2028 | Female | 125858.8 | 190215.7 | 61501.93 |
| 10-14 years | 2029 | Female | 127812.1 | 193198.4 | 62425.8 |
| 10-14 years | 2030 | Female | 127932.5 | 192588 | 63276.9 |
| 10-14 years | 2020 | Both | 176598.3 | 193273.6 | 159923.1 |
| 10-14 years | 2021 | Both | 176282.9 | 205480.6 | 147085.2 |
| 10-14 years | 2022 | Both | 176976.5 | 232116.9 | 121836.1 |
| 10-14 years | 2023 | Both | 178543.2 | 259586.9 | 97499.54 |
| 10-14 years | 2024 | Both | 180961.3 | 281824.7 | 80097.83 |
| 10-14 years | 2025 | Both | 184758.7 | 300368.4 | 69149.06 |
| 10-14 years | 2026 | Both | 190970.7 | 318950.7 | 62990.6 |
| 10-14 years | 2027 | Both | 201859.8 | 342702.5 | 61174.35 |
| 10-14 years | 2028 | Both | 207007.7 | 353829.2 | 61501.93 |
| 10-14 years | 2029 | Both | 210332.3 | 358843.7 | 62425.8 |
| 10-14 years | 2030 | Both | 210684.4 | 356661.4 | 64707.37 |
| 15-19 years | 2020 | Male | 72781 | 82386.05 | 63175.95 |
| 15-19 years | 2021 | Male | 72751.97 | 90216.91 | 55287.03 |
| 15-19 years | 2022 | Male | 72628.53 | 105588.3 | 39668.75 |
| 15-19 years | 2023 | Male | 72321.37 | 119782.7 | 24860.06 |
| 15-19 years | 2024 | Male | 71953.9 | 129572 | 14335.78 |
| 15-19 years | 2025 | Male | 71637.61 | 135772.3 | 7502.956 |
| 15-19 years | 2026 | Male | 71548.18 | 140005.7 | 3090.717 |
| 15-19 years | 2027 | Male | 71844.32 | 143439.7 | 248.9523 |
| 15-19 years | 2028 | Male | 72437.14 | 146404.5 | NA |
| 15-19 years | 2029 | Male | 73290.85 | 149246.9 | NA |
| 15-19 years | 2030 | Male | 74642.99 | 152724.5 | NA |
| 15-19 years | 2020 | Female | 162821.7 | 170257 | 155386.4 |
| 15-19 years | 2021 | Female | 161596.3 | 173945 | 149247.6 |
| 15-19 years | 2022 | Female | 160383.6 | 183186.9 | 137580.3 |
| 15-19 years | 2023 | Female | 159022.8 | 192188.1 | 125857.5 |
| 15-19 years | 2024 | Female | 157757.7 | 198474.7 | 117040.8 |
| 15-19 years | 2025 | Female | 156800.2 | 202579.2 | 111021.3 |
| 15-19 years | 2026 | Female | 156497 | 205831.5 | 107162.6 |
| 15-19 years | 2027 | Female | 157156.9 | 209278.4 | 105035.4 |
| 15-19 years | 2028 | Female | 158696.5 | 213207.7 | 104185.3 |
| 15-19 years | 2029 | Female | 161129.5 | 217937.4 | 104321.6 |
| 15-19 years | 2030 | Female | 164888.3 | 224271.1 | 105505.6 |
| 15-19 years | 2020 | Both | 235602.7 | 252643.1 | 218562.4 |
| 15-19 years | 2021 | Both | 234348.2 | 264161.9 | 204534.6 |
| 15-19 years | 2022 | Both | 233012.2 | 288775.2 | 177249.1 |
| 15-19 years | 2023 | Both | 231344.2 | 311970.8 | 150717.6 |
| 15-19 years | 2024 | Both | 229711.6 | 328046.7 | 131376.5 |
| 15-19 years | 2025 | Both | 228437.8 | 338351.4 | 118524.3 |
| 15-19 years | 2026 | Both | 228045.2 | 345837.1 | 110253.3 |
| 15-19 years | 2027 | Both | 229001.2 | 352718 | 105284.4 |
| 15-19 years | 2028 | Both | 231133.6 | 359612.3 | 104185.3 |
| 15-19 years | 2029 | Both | 234420.4 | 367184.3 | 104321.6 |
| 15-19 years | 2030 | Both | 239531.3 | 376995.6 | 105505.6 |

## Supplementary Table 14. Predicted incidence rates from 2020 to 2030 based on BAPC model

| **Age group** | **Year** | **Sex** | **Value** | **Upper** | **Lower** |
| --- | --- | --- | --- | --- | --- |
| <5 years | 2020 | Male | 783.0799 | 802.6028 | 764.1445 |
| <5 years | 2021 | Male | 795.1961 | 822.0971 | 769.6677 |
| <5 years | 2022 | Male | 807.537 | 844.2047 | 773.3 |
| <5 years | 2023 | Male | 820.1178 | 868.586 | 775.426 |
| <5 years | 2024 | Male | 832.9544 | 895.0842 | 776.3049 |
| <5 years | 2025 | Male | 846.0637 | 923.6486 | 776.1064 |
| <5 years | 2026 | Male | 859.4634 | 954.2871 | 774.9481 |
| <5 years | 2027 | Male | 873.1721 | 987.0431 | 772.9175 |
| <5 years | 2028 | Male | 887.2092 | 1021.984 | 770.0841 |
| <5 years | 2029 | Male | 901.5953 | 1059.196 | 766.506 |
| <5 years | 2030 | Male | 916.3519 | 1098.78 | 762.2336 |
| <5 years | 2020 | Female | 737.4083 | 756.0721 | 719.221 |
| <5 years | 2021 | Female | 747.1861 | 772.2302 | 723.1876 |
| <5 years | 2022 | Female | 757.1263 | 790.7365 | 725.3642 |
| <5 years | 2023 | Female | 767.2411 | 811.2768 | 726.1089 |
| <5 years | 2024 | Female | 777.5438 | 833.6807 | 725.6768 |
| <5 years | 2025 | Female | 788.0478 | 857.8762 | 724.2387 |
| <5 years | 2026 | Female | 798.7675 | 883.8482 | 721.9138 |
| <5 years | 2027 | Female | 809.7178 | 911.6164 | 718.7911 |
| <5 years | 2028 | Female | 820.9143 | 941.2236 | 714.9408 |
| <5 years | 2029 | Female | 832.3733 | 972.7293 | 710.4213 |
| <5 years | 2030 | Female | 844.1119 | 1006.207 | 705.2832 |
| <5 years | 2020 | Both | 760.2441 | 779.3374 | 741.6827 |
| <5 years | 2021 | Both | 771.1911 | 797.1636 | 746.4277 |
| <5 years | 2022 | Both | 782.3317 | 817.4706 | 749.3321 |
| <5 years | 2023 | Both | 793.6795 | 839.9314 | 750.7674 |
| <5 years | 2024 | Both | 805.2491 | 864.3825 | 750.9908 |
| <5 years | 2025 | Both | 817.0558 | 890.7624 | 750.1725 |
| <5 years | 2026 | Both | 829.1155 | 919.0676 | 748.431 |
| <5 years | 2027 | Both | 841.4449 | 949.3298 | 745.8543 |
| <5 years | 2028 | Both | 854.0617 | 981.6039 | 742.5125 |
| <5 years | 2029 | Both | 866.9843 | 1015.963 | 738.4637 |
| <5 years | 2030 | Both | 880.2319 | 1052.493 | 733.7584 |
| 5-9 years | 2020 | Male | 275.896 | 281.1442 | 270.7213 |
| 5-9 years | 2021 | Male | 277.258 | 283.8062 | 270.8479 |
| 5-9 years | 2022 | Male | 279.8264 | 288.3705 | 271.5424 |
| 5-9 years | 2023 | Male | 283.5846 | 294.8074 | 272.7961 |
| 5-9 years | 2024 | Male | 287.9871 | 302.5991 | 274.0513 |
| 5-9 years | 2025 | Male | 292.4768 | 311.1875 | 274.7962 |
| 5-9 years | 2026 | Male | 297.0587 | 320.5418 | 275.1088 |
| 5-9 years | 2027 | Male | 301.7385 | 330.6495 | 275.0432 |
| 5-9 years | 2028 | Male | 306.5222 | 341.5128 | 274.6384 |
| 5-9 years | 2029 | Male | 311.4164 | 353.1452 | 273.9243 |
| 5-9 years | 2030 | Male | 316.428 | 365.5685 | 272.9249 |
| 5-9 years | 2020 | Female | 360.5221 | 367.6565 | 353.4877 |
| 5-9 years | 2021 | Female | 362.4362 | 371.1977 | 353.8564 |
| 5-9 years | 2022 | Female | 365.5856 | 376.9138 | 354.5909 |
| 5-9 years | 2023 | Female | 369.9009 | 384.6959 | 355.6618 |
| 5-9 years | 2024 | Female | 374.8272 | 393.9723 | 356.5523 |
| 5-9 years | 2025 | Female | 379.8421 | 404.2014 | 356.8043 |
| 5-9 years | 2026 | Female | 384.9518 | 415.3412 | 356.512 |
| 5-9 years | 2027 | Female | 390.1627 | 427.3722 | 355.7436 |
| 5-9 years | 2028 | Female | 395.4818 | 440.2929 | 354.5497 |
| 5-9 years | 2029 | Female | 400.9162 | 454.1148 | 352.97 |
| 5-9 years | 2030 | Female | 406.4738 | 468.8595 | 351.0369 |
| 5-9 years | 2020 | Both | 318.2091 | 324.4004 | 312.1045 |
| 5-9 years | 2021 | Both | 319.8471 | 327.502 | 312.3521 |
| 5-9 years | 2022 | Both | 322.706 | 332.6422 | 313.0666 |
| 5-9 years | 2023 | Both | 326.7427 | 339.7517 | 314.2289 |
| 5-9 years | 2024 | Both | 331.4072 | 348.2857 | 315.3018 |
| 5-9 years | 2025 | Both | 336.1595 | 357.6945 | 315.8002 |
| 5-9 years | 2026 | Both | 341.0053 | 367.9415 | 315.8104 |
| 5-9 years | 2027 | Both | 345.9506 | 379.0109 | 315.3934 |
| 5-9 years | 2028 | Both | 351.002 | 390.9029 | 314.5941 |
| 5-9 years | 2029 | Both | 356.1663 | 403.63 | 313.4471 |
| 5-9 years | 2030 | Both | 361.4509 | 417.214 | 311.9809 |
| 10-14 years | 2020 | Male | 178.7936 | 182.1181 | 175.5125 |
| 10-14 years | 2021 | Male | 179.0478 | 183.1884 | 174.9942 |
| 10-14 years | 2022 | Male | 179.4368 | 184.8547 | 174.1852 |
| 10-14 years | 2023 | Male | 179.9817 | 187.0556 | 173.1889 |
| 10-14 years | 2024 | Male | 180.557 | 189.5993 | 171.951 |
| 10-14 years | 2025 | Male | 181.0592 | 192.3443 | 170.4153 |
| 10-14 years | 2026 | Male | 181.9868 | 195.7774 | 169.1001 |
| 10-14 years | 2027 | Male | 183.7151 | 200.2965 | 168.3691 |
| 10-14 years | 2028 | Male | 186.2338 | 205.9365 | 168.1804 |
| 10-14 years | 2029 | Male | 189.1845 | 212.3725 | 168.1606 |
| 10-14 years | 2030 | Male | 192.2015 | 219.2509 | 167.9553 |
| 10-14 years | 2020 | Female | 324.9352 | 331.2334 | 318.7211 |
| 10-14 years | 2021 | Female | 325.1832 | 332.9097 | 317.6154 |
| 10-14 years | 2022 | Female | 325.687 | 335.6746 | 315.9939 |
| 10-14 years | 2023 | Female | 326.5188 | 339.4619 | 314.0692 |
| 10-14 years | 2024 | Female | 327.5558 | 344.0323 | 311.8452 |
| 10-14 years | 2025 | Female | 328.6807 | 349.1986 | 309.2911 |
| 10-14 years | 2026 | Female | 330.488 | 355.5282 | 307.0445 |
| 10-14 years | 2027 | Female | 333.4369 | 363.5115 | 305.5524 |
| 10-14 years | 2028 | Female | 337.4652 | 373.1443 | 304.719 |
| 10-14 years | 2029 | Female | 342.0663 | 383.9603 | 304.0282 |
| 10-14 years | 2030 | Female | 346.7643 | 395.505 | 303.0193 |
| 10-14 years | 2020 | Both | 251.8644 | 256.6758 | 247.1168 |
| 10-14 years | 2021 | Both | 252.1155 | 258.0491 | 246.3048 |
| 10-14 years | 2022 | Both | 252.5619 | 260.2646 | 245.0895 |
| 10-14 years | 2023 | Both | 253.2502 | 263.2587 | 243.629 |
| 10-14 years | 2024 | Both | 254.0564 | 266.8158 | 241.8981 |
| 10-14 years | 2025 | Both | 254.87 | 270.7714 | 239.8532 |
| 10-14 years | 2026 | Both | 256.2374 | 275.6528 | 238.0723 |
| 10-14 years | 2027 | Both | 258.576 | 281.904 | 236.9607 |
| 10-14 years | 2028 | Both | 261.8495 | 289.5404 | 236.4497 |
| 10-14 years | 2029 | Both | 265.6254 | 298.1664 | 236.0944 |
| 10-14 years | 2030 | Both | 269.4829 | 307.378 | 235.4873 |
| 15-19 years | 2020 | Male | 182.5321 | 185.8983 | 179.2092 |
| 15-19 years | 2021 | Male | 182.9096 | 187.1107 | 178.7962 |
| 15-19 years | 2022 | Male | 183.2533 | 188.7593 | 177.9158 |
| 15-19 years | 2023 | Male | 183.5555 | 190.7444 | 176.652 |
| 15-19 years | 2024 | Male | 183.8076 | 192.9862 | 175.071 |
| 15-19 years | 2025 | Male | 184.0161 | 195.4516 | 173.2279 |
| 15-19 years | 2026 | Male | 184.3122 | 198.2434 | 171.2908 |
| 15-19 years | 2027 | Male | 184.7565 | 201.4111 | 169.34 |
| 15-19 years | 2028 | Male | 185.3702 | 204.9754 | 167.4073 |
| 15-19 years | 2029 | Male | 186.0231 | 208.7988 | 165.3807 |
| 15-19 years | 2030 | Male | 186.608 | 212.7683 | 163.1707 |
| 15-19 years | 2020 | Female | 476.2953 | 485.4441 | 467.2651 |
| 15-19 years | 2021 | Female | 477.2416 | 488.4947 | 466.216 |
| 15-19 years | 2022 | Female | 477.9852 | 492.5598 | 463.8354 |
| 15-19 years | 2023 | Female | 478.5493 | 497.4382 | 460.3728 |
| 15-19 years | 2024 | Female | 478.9386 | 502.9478 | 456.0337 |
| 15-19 years | 2025 | Female | 479.1877 | 509.013 | 450.9868 |
| 15-19 years | 2026 | Female | 479.645 | 515.9067 | 445.6776 |
| 15-19 years | 2027 | Female | 480.5007 | 523.7899 | 440.3479 |
| 15-19 years | 2028 | Female | 481.8615 | 532.7706 | 435.1282 |
| 15-19 years | 2029 | Female | 483.5452 | 542.6644 | 429.8675 |
| 15-19 years | 2030 | Female | 485.3782 | 553.2989 | 424.4213 |
| 15-19 years | 2020 | Both | 329.4137 | 335.6712 | 323.2372 |
| 15-19 years | 2021 | Both | 330.0756 | 337.8027 | 322.5061 |
| 15-19 years | 2022 | Both | 330.6193 | 340.6596 | 320.8756 |
| 15-19 years | 2023 | Both | 331.0524 | 344.0913 | 318.5124 |
| 15-19 years | 2024 | Both | 331.3731 | 347.967 | 315.5524 |
| 15-19 years | 2025 | Both | 331.6019 | 352.2323 | 312.1074 |
| 15-19 years | 2026 | Both | 331.9786 | 357.075 | 308.4842 |
| 15-19 years | 2027 | Both | 332.6286 | 362.6005 | 304.844 |
| 15-19 years | 2028 | Both | 333.6159 | 368.873 | 301.2678 |
| 15-19 years | 2029 | Both | 334.7842 | 375.7316 | 297.6241 |
| 15-19 years | 2030 | Both | 335.9931 | 383.0336 | 293.796 |

## Supplementary Table 15. Predicted prevalence cases from 2020 to 2030 based on BAPC model

| **Age group** | **Year** | **Sex** | **Value** | **Upper** | **Lower** |
| --- | --- | --- | --- | --- | --- |
| <5 years | 2020 | Male | 1102884 | 1128755 | 1077013 |
| <5 years | 2021 | Male | 1083115 | 1117619 | 1048611 |
| <5 years | 2022 | Male | 1030323 | 1074035 | 986610.3 |
| <5 years | 2023 | Male | 1004936 | 1059821 | 950050.3 |
| <5 years | 2024 | Male | 983418.2 | 1050521 | 916315.6 |
| <5 years | 2025 | Male | 963723.9 | 1043868 | 883579.3 |
| <5 years | 2026 | Male | 944986.2 | 1038821 | 851151.4 |
| <5 years | 2027 | Male | 926958.5 | 1035004 | 818913 |
| <5 years | 2028 | Male | 909750.5 | 1032455 | 787046 |
| <5 years | 2029 | Male | 893895.6 | 1031730 | 756060.7 |
| <5 years | 2030 | Male | 879850.8 | 1033358 | 726343.7 |
| <5 years | 2020 | Female | 831104.1 | 851445 | 810763.2 |
| <5 years | 2021 | Female | 814824.5 | 841396.8 | 788252.3 |
| <5 years | 2022 | Female | 773346.6 | 806589.3 | 740103.8 |
| <5 years | 2023 | Female | 752816.3 | 794214.7 | 711417.9 |
| <5 years | 2024 | Female | 735267.2 | 785567.2 | 684967.3 |
| <5 years | 2025 | Female | 719140.6 | 778918.2 | 659363 |
| <5 years | 2026 | Female | 703783 | 773483.4 | 634082.6 |
| <5 years | 2027 | Female | 689015 | 768988.6 | 609041.3 |
| <5 years | 2028 | Female | 674910.3 | 765452.8 | 584367.7 |
| <5 years | 2029 | Female | 661866.5 | 763289.2 | 560443.8 |
| <5 years | 2030 | Female | 650202.3 | 762863.7 | 537540.8 |
| <5 years | 2020 | Both | 1933988 | 1980200 | 1887776 |
| <5 years | 2021 | Both | 1897939 | 1959016 | 1836863 |
| <5 years | 2022 | Both | 1803669 | 1880625 | 1726714 |
| <5 years | 2023 | Both | 1757752 | 1854036 | 1661468 |
| <5 years | 2024 | Both | 1718685 | 1836088 | 1601283 |
| <5 years | 2025 | Both | 1682864 | 1822787 | 1542942 |
| <5 years | 2026 | Both | 1648769 | 1812304 | 1485234 |
| <5 years | 2027 | Both | 1615973 | 1803993 | 1427954 |
| <5 years | 2028 | Both | 1584661 | 1797908 | 1371414 |
| <5 years | 2029 | Both | 1555762 | 1795020 | 1316504 |
| <5 years | 2030 | Both | 1530053 | 1796222 | 1263885 |
| 5-9 years | 2020 | Male | 1140914 | 1161454 | 1120373 |
| 5-9 years | 2021 | Male | 1175356 | 1202714 | 1147998 |
| 5-9 years | 2022 | Male | 1238896 | 1279038 | 1198753 |
| 5-9 years | 2023 | Male | 1268632 | 1323131 | 1214133 |
| 5-9 years | 2024 | Male | 1287907 | 1356641 | 1219173 |
| 5-9 years | 2025 | Male | 1289317 | 1372408 | 1206226 |
| 5-9 years | 2026 | Male | 1266451 | 1363855 | 1169046 |
| 5-9 years | 2027 | Male | 1204906 | 1314456 | 1095356 |
| 5-9 years | 2028 | Male | 1175485 | 1300536 | 1050433 |
| 5-9 years | 2029 | Male | 1150592 | 1292302 | 1008881 |
| 5-9 years | 2030 | Male | 1127898 | 1287086 | 968709.3 |
| 5-9 years | 2020 | Female | 975407.5 | 993801.7 | 957013.3 |
| 5-9 years | 2021 | Female | 1008485 | 1032457 | 984512.9 |
| 5-9 years | 2022 | Female | 1065691 | 1100180 | 1031202 |
| 5-9 years | 2023 | Female | 1091460 | 1138061 | 1044860 |
| 5-9 years | 2024 | Female | 1106886 | 1165866 | 1047905 |
| 5-9 years | 2025 | Female | 1106380 | 1177863 | 1034896 |
| 5-9 years | 2026 | Female | 1084885 | 1168664 | 1001106 |
| 5-9 years | 2027 | Female | 1029777 | 1123774 | 935780.4 |
| 5-9 years | 2028 | Female | 1002638 | 1109636 | 895639.4 |
| 5-9 years | 2029 | Female | 979465.5 | 1100358 | 858573.1 |
| 5-9 years | 2030 | Female | 958253.4 | 1093657 | 822849.9 |
| 5-9 years | 2020 | Both | 2116321 | 2155255 | 2077387 |
| 5-9 years | 2021 | Both | 2183841 | 2235171 | 2132511 |
| 5-9 years | 2022 | Both | 2304587 | 2379218 | 2229956 |
| 5-9 years | 2023 | Both | 2360093 | 2461192 | 2258993 |
| 5-9 years | 2024 | Both | 2394792 | 2522507 | 2267078 |
| 5-9 years | 2025 | Both | 2395697 | 2550271 | 2241123 |
| 5-9 years | 2026 | Both | 2351336 | 2532519 | 2170153 |
| 5-9 years | 2027 | Both | 2234683 | 2438230 | 2031136 |
| 5-9 years | 2028 | Both | 2178122 | 2410172 | 1946072 |
| 5-9 years | 2029 | Both | 2130057 | 2392660 | 1867454 |
| 5-9 years | 2030 | Both | 2086151 | 2380743 | 1791559 |
| 10-14 years | 2020 | Male | 731016.3 | 744748.7 | 717284 |
| 10-14 years | 2021 | Male | 730650.7 | 749742.8 | 711558.5 |
| 10-14 years | 2022 | Male | 733706 | 762844.3 | 704567.6 |
| 10-14 years | 2023 | Male | 739427.8 | 779272.4 | 699583.1 |
| 10-14 years | 2024 | Male | 747608.1 | 797206 | 698010.3 |
| 10-14 years | 2025 | Male | 760795.9 | 819948.4 | 701643.5 |
| 10-14 years | 2026 | Male | 783957.6 | 853691.4 | 714223.9 |
| 10-14 years | 2027 | Male | 826610.3 | 909610.4 | 743610.2 |
| 10-14 years | 2028 | Male | 846718.5 | 941820 | 751616.9 |
| 10-14 years | 2029 | Male | 859868 | 967333.5 | 752402.6 |
| 10-14 years | 2030 | Male | 861114.2 | 980599.9 | 741628.4 |
| 10-14 years | 2020 | Female | 754787.4 | 769058.7 | 740516.1 |
| 10-14 years | 2021 | Female | 753860.9 | 772348.2 | 735373.6 |
| 10-14 years | 2022 | Female | 757045.7 | 783463.4 | 730628 |
| 10-14 years | 2023 | Female | 764032.9 | 799855.2 | 728210.6 |
| 10-14 years | 2024 | Female | 774928.8 | 820281.1 | 729576.6 |
| 10-14 years | 2025 | Female | 791854.7 | 847251.4 | 736458.1 |
| 10-14 years | 2026 | Female | 818896.2 | 885726.8 | 752065.6 |
| 10-14 years | 2027 | Female | 865617.2 | 946756.5 | 784477.9 |
| 10-14 years | 2028 | Female | 886808.8 | 981256.9 | 792360.6 |
| 10-14 years | 2029 | Female | 899618.9 | 1007701 | 791536.6 |
| 10-14 years | 2030 | Female | 899508.1 | 1020830 | 778186.2 |
| 10-14 years | 2020 | Both | 1485804 | 1513807 | 1457800 |
| 10-14 years | 2021 | Both | 1484512 | 1522091 | 1446932 |
| 10-14 years | 2022 | Both | 1490752 | 1546308 | 1435196 |
| 10-14 years | 2023 | Both | 1503461 | 1579128 | 1427794 |
| 10-14 years | 2024 | Both | 1522537 | 1617487 | 1427587 |
| 10-14 years | 2025 | Both | 1552651 | 1667200 | 1438102 |
| 10-14 years | 2026 | Both | 1602854 | 1739418 | 1466289 |
| 10-14 years | 2027 | Both | 1692227 | 1856367 | 1528088 |
| 10-14 years | 2028 | Both | 1733527 | 1923077 | 1543978 |
| 10-14 years | 2029 | Both | 1759487 | 1975035 | 1543939 |
| 10-14 years | 2030 | Both | 1760622 | 2001430 | 1519815 |
| 15-19 years | 2020 | Male | 562475.4 | 573962.2 | 550988.6 |
| 15-19 years | 2021 | Male | 563423.8 | 580569.9 | 546277.7 |
| 15-19 years | 2022 | Male | 563891.6 | 591661.6 | 536121.7 |
| 15-19 years | 2023 | Male | 563161.9 | 601183.3 | 525140.5 |
| 15-19 years | 2024 | Male | 561824.1 | 607880.7 | 515767.4 |
| 15-19 years | 2025 | Male | 560273.8 | 612917.3 | 507630.2 |
| 15-19 years | 2026 | Male | 560056.2 | 618807.7 | 501304.7 |
| 15-19 years | 2027 | Male | 562531.5 | 627623 | 497439.9 |
| 15-19 years | 2028 | Male | 567098.7 | 639061.9 | 495135.4 |
| 15-19 years | 2029 | Male | 573585.2 | 653215.6 | 493954.8 |
| 15-19 years | 2030 | Male | 583932.4 | 672522.8 | 495342 |
| 15-19 years | 2020 | Female | 814801.2 | 830035.5 | 799566.9 |
| 15-19 years | 2021 | Female | 809876.1 | 829516.1 | 790236.2 |
| 15-19 years | 2022 | Female | 805361.7 | 833092 | 777631.5 |
| 15-19 years | 2023 | Female | 800407.6 | 837351.9 | 763463.2 |
| 15-19 years | 2024 | Female | 795836.3 | 841660.4 | 750012.3 |
| 15-19 years | 2025 | Female | 792191.4 | 846782.6 | 737600.2 |
| 15-19 years | 2026 | Female | 791313.2 | 855087 | 727539.3 |
| 15-19 years | 2027 | Female | 794841 | 868679.7 | 721002.3 |
| 15-19 years | 2028 | Female | 802416.5 | 887438 | 717395 |
| 15-19 years | 2029 | Female | 814139.6 | 911715.3 | 716563.9 |
| 15-19 years | 2030 | Female | 832234.7 | 944270.6 | 720198.9 |
| 15-19 years | 2020 | Both | 1377277 | 1403998 | 1350555 |
| 15-19 years | 2021 | Both | 1373300 | 1410086 | 1336514 |
| 15-19 years | 2022 | Both | 1369253 | 1424754 | 1313753 |
| 15-19 years | 2023 | Both | 1363569 | 1438535 | 1288604 |
| 15-19 years | 2024 | Both | 1357660 | 1449541 | 1265780 |
| 15-19 years | 2025 | Both | 1352465 | 1459700 | 1245230 |
| 15-19 years | 2026 | Both | 1351369 | 1473895 | 1228844 |
| 15-19 years | 2027 | Both | 1357372 | 1496303 | 1218442 |
| 15-19 years | 2028 | Both | 1369515 | 1526500 | 1212530 |
| 15-19 years | 2029 | Both | 1387725 | 1564931 | 1210519 |
| 15-19 years | 2030 | Both | 1416167 | 1616793 | 1215541 |

## Supplementary Table 16. Predicted prevalence rates from 2020 to 2030 based on BAPC model

| **Age group** | **Year** | **Sex** | **Value** | **Upper** | **Lower** |
| --- | --- | --- | --- | --- | --- |
| <5 years | 2020 | Male | 2543.849 | 2601.726 | 2487.54 |
| <5 years | 2021 | Male | 2579.779 | 2659.982 | 2503.267 |
| <5 years | 2022 | Male | 2616.325 | 2726.254 | 2512.978 |
| <5 years | 2023 | Male | 2653.53 | 2799.422 | 2517.937 |
| <5 years | 2024 | Male | 2691.439 | 2878.953 | 2518.983 |
| <5 years | 2025 | Male | 2730.1 | 2964.648 | 2516.656 |
| <5 years | 2026 | Male | 2769.563 | 3056.494 | 2511.332 |
| <5 years | 2027 | Male | 2809.882 | 3154.587 | 2503.289 |
| <5 years | 2028 | Male | 2851.11 | 3259.096 | 2492.748 |
| <5 years | 2029 | Male | 2893.305 | 3370.246 | 2479.894 |
| <5 years | 2030 | Male | 2936.528 | 3488.304 | 2464.889 |
| <5 years | 2020 | Female | 2238.352 | 2291.064 | 2186.935 |
| <5 years | 2021 | Female | 2265.672 | 2337.135 | 2197.112 |
| <5 years | 2022 | Female | 2293.419 | 2390.069 | 2201.93 |
| <5 years | 2023 | Female | 2321.626 | 2448.878 | 2202.503 |
| <5 years | 2024 | Female | 2350.331 | 2513.036 | 2199.603 |
| <5 years | 2025 | Female | 2379.571 | 2582.314 | 2193.741 |
| <5 years | 2026 | Female | 2409.386 | 2656.65 | 2185.27 |
| <5 years | 2027 | Female | 2439.818 | 2736.086 | 2174.454 |
| <5 years | 2028 | Female | 2470.908 | 2820.728 | 2161.504 |
| <5 years | 2029 | Female | 2502.703 | 2910.729 | 2146.595 |
| <5 years | 2030 | Female | 2535.248 | 3006.282 | 2129.877 |
| <5 years | 2020 | Both | 2391.101 | 2446.395 | 2337.237 |
| <5 years | 2021 | Both | 2422.726 | 2498.559 | 2350.189 |
| <5 years | 2022 | Both | 2454.872 | 2558.161 | 2357.454 |
| <5 years | 2023 | Both | 2487.578 | 2624.15 | 2360.22 |
| <5 years | 2024 | Both | 2520.885 | 2695.995 | 2359.293 |
| <5 years | 2025 | Both | 2554.836 | 2773.481 | 2355.199 |
| <5 years | 2026 | Both | 2589.475 | 2856.572 | 2348.301 |
| <5 years | 2027 | Both | 2624.85 | 2945.337 | 2338.872 |
| <5 years | 2028 | Both | 2661.009 | 3039.912 | 2327.126 |
| <5 years | 2029 | Both | 2698.004 | 3140.488 | 2313.245 |
| <5 years | 2030 | Both | 2735.888 | 3247.293 | 2297.383 |
| 5-9 years | 2020 | Male | 2830.041 | 2879.271 | 2781.433 |
| 5-9 years | 2021 | Male | 2842.517 | 2904.722 | 2781.599 |
| 5-9 years | 2022 | Male | 2866.672 | 2948.869 | 2786.968 |
| 5-9 years | 2023 | Male | 2902.04 | 3010.901 | 2797.359 |
| 5-9 years | 2024 | Male | 2943.196 | 3085.462 | 2807.436 |
| 5-9 years | 2025 | Male | 2985.099 | 3167.482 | 2812.584 |
| 5-9 years | 2026 | Male | 3027.802 | 3256.664 | 2813.526 |
| 5-9 years | 2027 | Male | 3071.357 | 3352.883 | 2810.775 |
| 5-9 years | 2028 | Male | 3115.822 | 3456.144 | 2804.707 |
| 5-9 years | 2029 | Male | 3161.254 | 3566.553 | 2795.614 |
| 5-9 years | 2030 | Male | 3207.717 | 3684.29 | 2783.735 |
| 5-9 years | 2020 | Female | 2853.167 | 2905.442 | 2801.559 |
| 5-9 years | 2021 | Female | 2865.061 | 2929.946 | 2801.458 |
| 5-9 years | 2022 | Female | 2886.908 | 2971.609 | 2804.614 |
| 5-9 years | 2023 | Female | 2918.036 | 3029.353 | 2810.752 |
| 5-9 years | 2024 | Female | 2953.826 | 3098.404 | 2815.579 |
| 5-9 years | 2025 | Female | 2990.219 | 3174.576 | 2815.507 |
| 5-9 years | 2026 | Female | 3027.261 | 3257.535 | 2811.256 |
| 5-9 years | 2027 | Female | 3065.002 | 3347.12 | 2803.351 |
| 5-9 years | 2028 | Female | 3103.491 | 3443.299 | 2792.182 |
| 5-9 years | 2029 | Female | 3142.784 | 3546.138 | 2778.056 |
| 5-9 years | 2030 | Female | 3182.935 | 3655.776 | 2761.227 |
| 5-9 years | 2020 | Both | 2841.604 | 2892.357 | 2791.496 |
| 5-9 years | 2021 | Both | 2853.789 | 2917.334 | 2791.528 |
| 5-9 years | 2022 | Both | 2876.79 | 2960.239 | 2795.791 |
| 5-9 years | 2023 | Both | 2910.038 | 3020.127 | 2804.056 |
| 5-9 years | 2024 | Both | 2948.511 | 3091.933 | 2811.507 |
| 5-9 years | 2025 | Both | 2987.659 | 3171.029 | 2814.046 |
| 5-9 years | 2026 | Both | 3027.532 | 3257.1 | 2812.391 |
| 5-9 years | 2027 | Both | 3068.179 | 3350.002 | 2807.063 |
| 5-9 years | 2028 | Both | 3109.656 | 3449.722 | 2798.444 |
| 5-9 years | 2029 | Both | 3152.019 | 3556.346 | 2786.835 |
| 5-9 years | 2030 | Both | 3195.326 | 3670.033 | 2772.481 |
| 10-14 years | 2020 | Male | 1869.338 | 1901.066 | 1837.982 |
| 10-14 years | 2021 | Male | 1873.619 | 1913.732 | 1834.332 |
| 10-14 years | 2022 | Male | 1878.249 | 1931.469 | 1826.641 |
| 10-14 years | 2023 | Male | 1883.758 | 1953.876 | 1816.357 |
| 10-14 years | 2024 | Male | 1889.189 | 1979.312 | 1803.278 |
| 10-14 years | 2025 | Male | 1893.739 | 2006.601 | 1787.08 |
| 10-14 years | 2026 | Male | 1902.432 | 2040.65 | 1772.985 |
| 10-14 years | 2027 | Male | 1919.016 | 2085.427 | 1764.627 |
| 10-14 years | 2028 | Male | 1943.188 | 2141.023 | 1761.437 |
| 10-14 years | 2029 | Male | 1971.32 | 2204.072 | 1759.697 |
| 10-14 years | 2030 | Male | 2000.044 | 2271.327 | 1756.131 |
| 10-14 years | 2020 | Female | 2298.998 | 2340.19 | 2258.302 |
| 10-14 years | 2021 | Female | 2302.674 | 2353.835 | 2252.514 |
| 10-14 years | 2022 | Female | 2306.83 | 2373.75 | 2241.806 |
| 10-14 years | 2023 | Female | 2312.158 | 2399.572 | 2227.94 |
| 10-14 years | 2024 | Female | 2317.904 | 2429.699 | 2211.086 |
| 10-14 years | 2025 | Female | 2323.411 | 2462.991 | 2191.198 |
| 10-14 years | 2026 | Female | 2333.522 | 2504.104 | 2173.407 |
| 10-14 years | 2027 | Female | 2351.831 | 2556.847 | 2161.218 |
| 10-14 years | 2028 | Female | 2377.8 | 2621.062 | 2153.871 |
| 10-14 years | 2029 | Female | 2407.67 | 2693.24 | 2147.559 |
| 10-14 years | 2030 | Female | 2438.14 | 2770.226 | 2139.076 |
| 10-14 years | 2020 | Both | 2084.168 | 2120.628 | 2048.142 |
| 10-14 years | 2021 | Both | 2088.147 | 2133.783 | 2043.423 |
| 10-14 years | 2022 | Both | 2092.539 | 2152.609 | 2034.224 |
| 10-14 years | 2023 | Both | 2097.958 | 2176.724 | 2022.148 |
| 10-14 years | 2024 | Both | 2103.546 | 2204.506 | 2007.182 |
| 10-14 years | 2025 | Both | 2108.575 | 2234.796 | 1989.139 |
| 10-14 years | 2026 | Both | 2117.977 | 2272.377 | 1973.196 |
| 10-14 years | 2027 | Both | 2135.424 | 2321.137 | 1962.923 |
| 10-14 years | 2028 | Both | 2160.494 | 2381.043 | 1957.654 |
| 10-14 years | 2029 | Both | 2189.495 | 2448.656 | 1953.628 |
| 10-14 years | 2030 | Both | 2219.092 | 2520.776 | 1947.603 |
| 15-19 years | 2020 | Male | 1410.668 | 1434.427 | 1387.194 |
| 15-19 years | 2021 | Male | 1416.534 | 1446.678 | 1387.017 |
| 15-19 years | 2022 | Male | 1422.788 | 1462.928 | 1383.869 |
| 15-19 years | 2023 | Male | 1429.335 | 1482.368 | 1378.361 |
| 15-19 years | 2024 | Male | 1435.19 | 1503.471 | 1370.098 |
| 15-19 years | 2025 | Male | 1439.18 | 1524.715 | 1358.333 |
| 15-19 years | 2026 | Male | 1442.737 | 1547.31 | 1344.784 |
| 15-19 years | 2027 | Male | 1446.619 | 1571.912 | 1330.366 |
| 15-19 years | 2028 | Male | 1451.233 | 1598.912 | 1315.573 |
| 15-19 years | 2029 | Male | 1455.844 | 1627.522 | 1299.801 |
| 15-19 years | 2030 | Male | 1459.835 | 1657.094 | 1282.549 |
| 15-19 years | 2020 | Female | 2383.502 | 2425.873 | 2341.647 |
| 15-19 years | 2021 | Female | 2391.804 | 2444.601 | 2340.043 |
| 15-19 years | 2022 | Female | 2400.189 | 2469.488 | 2332.857 |
| 15-19 years | 2023 | Female | 2408.676 | 2499.417 | 2321.25 |
| 15-19 years | 2024 | Female | 2416.09 | 2532.282 | 2305.056 |
| 15-19 years | 2025 | Female | 2420.968 | 2566.02 | 2283.545 |
| 15-19 years | 2026 | Female | 2425.282 | 2602.199 | 2259.188 |
| 15-19 years | 2027 | Female | 2430.194 | 2641.804 | 2233.429 |
| 15-19 years | 2028 | Female | 2436.435 | 2685.53 | 2207.148 |
| 15-19 years | 2029 | Female | 2443.211 | 2732.533 | 2179.734 |
| 15-19 years | 2030 | Female | 2449.831 | 2782.11 | 2150.655 |
| 15-19 years | 2020 | Both | 1897.085 | 1930.15 | 1864.421 |
| 15-19 years | 2021 | Both | 1904.169 | 1945.64 | 1863.53 |
| 15-19 years | 2022 | Both | 1911.488 | 1966.208 | 1858.363 |
| 15-19 years | 2023 | Both | 1919.006 | 1990.892 | 1849.806 |
| 15-19 years | 2024 | Both | 1925.64 | 2017.876 | 1837.577 |
| 15-19 years | 2025 | Both | 1930.074 | 2045.367 | 1820.939 |
| 15-19 years | 2026 | Both | 1934.01 | 2074.755 | 1801.986 |
| 15-19 years | 2027 | Both | 1938.406 | 2106.858 | 1781.898 |
| 15-19 years | 2028 | Both | 1943.834 | 2142.221 | 1761.36 |
| 15-19 years | 2029 | Both | 1949.528 | 2180.027 | 1739.768 |
| 15-19 years | 2030 | Both | 1954.833 | 2219.602 | 1716.602 |

## Supplementary Table 17. Predicted DALY cases from 2020 to 2030 based on BAPC model

| **Age group** | **Year** | **Sex** | **Value** | **Upper** | **Lower** |
| --- | --- | --- | --- | --- | --- |
| <5 years | 2020 | Male | 49525.66 | 63057.14 | 35994.17 |
| <5 years | 2021 | Male | 48547.93 | 67345.43 | 29750.43 |
| <5 years | 2022 | Male | 46096.47 | 68851.63 | 23341.3 |
| <5 years | 2023 | Male | 44878.08 | 70649.61 | 19106.54 |
| <5 years | 2024 | Male | 43836.96 | 71521.56 | 16152.37 |
| <5 years | 2025 | Male | 42881.13 | 71667.34 | 14094.91 |
| <5 years | 2026 | Male | 41971.68 | 71287.17 | 12656.18 |
| <5 years | 2027 | Male | 41097.45 | 70546.78 | 11648.13 |
| <5 years | 2028 | Male | 40263.16 | 69582.05 | 10944.27 |
| <5 years | 2029 | Male | 39492.18 | 68523.25 | 10461.1 |
| <5 years | 2030 | Male | 38804.37 | 67469.26 | 10139.47 |
| <5 years | 2020 | Female | 37395.45 | 48922.36 | 25868.54 |
| <5 years | 2021 | Female | 36607.86 | 52160.35 | 21055.37 |
| <5 years | 2022 | Female | 34692.3 | 53296.65 | 16087.95 |
| <5 years | 2023 | Female | 33720.92 | 54711.58 | 12730.26 |
| <5 years | 2024 | Female | 32885.96 | 55411.35 | 10360.57 |
| <5 years | 2025 | Female | 32117.22 | 55537.37 | 8697.077 |
| <5 years | 2026 | Female | 31385.3 | 55241.75 | 7528.848 |
| <5 years | 2027 | Female | 30682.04 | 54653.81 | 6710.271 |
| <5 years | 2028 | Female | 30010.63 | 53879.66 | 6141.602 |
| <5 years | 2029 | Female | 29388.59 | 53021.84 | 5755.334 |
| <5 years | 2030 | Female | 28829.84 | 52156.67 | 5503.012 |
| <5 years | 2020 | Both | 86921.11 | 111979.5 | 61862.72 |
| <5 years | 2021 | Both | 85155.79 | 119505.8 | 50805.8 |
| <5 years | 2022 | Both | 80788.77 | 122148.3 | 39429.25 |
| <5 years | 2023 | Both | 78598.99 | 125361.2 | 31836.79 |
| <5 years | 2024 | Both | 76722.92 | 126932.9 | 26512.93 |
| <5 years | 2025 | Both | 74998.35 | 127204.7 | 22791.99 |
| <5 years | 2026 | Both | 73356.98 | 126528.9 | 20185.03 |
| <5 years | 2027 | Both | 71779.49 | 125200.6 | 18358.4 |
| <5 years | 2028 | Both | 70273.79 | 123461.7 | 17085.87 |
| <5 years | 2029 | Both | 68880.76 | 121545.1 | 16216.43 |
| <5 years | 2030 | Both | 67634.21 | 119625.9 | 15642.48 |
| 5-9 years | 2020 | Male | 51444.82 | 62084.32 | 40805.31 |
| 5-9 years | 2021 | Male | 53026.43 | 73967.98 | 32084.87 |
| 5-9 years | 2022 | Male | 55866.79 | 98536.36 | 13197.23 |
| 5-9 years | 2023 | Male | 57126.27 | 119258.6 | NA |
| 5-9 years | 2024 | Male | 57886.65 | 130757.6 | NA |
| 5-9 years | 2025 | Male | 57843.91 | 133737.8 | NA |
| 5-9 years | 2026 | Male | 56715.03 | 130731.7 | NA |
| 5-9 years | 2027 | Male | 53861.98 | 122345 | NA |
| 5-9 years | 2028 | Male | 52453.23 | 116847.9 | NA |
| 5-9 years | 2029 | Male | 51251.88 | 111795.9 | NA |
| 5-9 years | 2030 | Male | 50153.26 | 107118.2 | NA |
| 5-9 years | 2020 | Female | 43980.66 | 52693.6 | 35267.72 |
| 5-9 years | 2021 | Female | 45479.34 | 61523.83 | 29434.84 |
| 5-9 years | 2022 | Female | 48031.99 | 80482.06 | 15581.93 |
| 5-9 years | 2023 | Female | 49132.85 | 96797.94 | 1467.75 |
| 5-9 years | 2024 | Female | 49751.98 | 106181.4 | NA |
| 5-9 years | 2025 | Female | 49655 | 108885.4 | NA |
| 5-9 years | 2026 | Female | 48618.36 | 106734.8 | NA |
| 5-9 years | 2027 | Female | 46081.08 | 100081.6 | NA |
| 5-9 years | 2028 | Female | 44801.39 | 95767.24 | NA |
| 5-9 years | 2029 | Female | 43702.85 | 91770.87 | NA |
| 5-9 years | 2030 | Female | 42695.25 | 88043.95 | NA |
| 5-9 years | 2020 | Both | 95425.48 | 114777.9 | 76073.03 |
| 5-9 years | 2021 | Both | 98505.76 | 135491.8 | 61519.71 |
| 5-9 years | 2022 | Both | 103898.8 | 179018.4 | 28779.16 |
| 5-9 years | 2023 | Both | 106259.1 | 216056.5 | 1467.75 |
| 5-9 years | 2024 | Both | 107638.6 | 236939 | NA |
| 5-9 years | 2025 | Both | 107498.9 | 242623.2 | NA |
| 5-9 years | 2026 | Both | 105333.4 | 237466.5 | NA |
| 5-9 years | 2027 | Both | 99943.07 | 222426.6 | NA |
| 5-9 years | 2028 | Both | 97254.62 | 212615.2 | NA |
| 5-9 years | 2029 | Both | 94954.73 | 203566.8 | NA |
| 5-9 years | 2030 | Both | 92848.5 | 195162.2 | NA |
| 10-14 years | 2020 | Male | 32784.46 | 43900.43 | 21668.48 |
| 10-14 years | 2021 | Male | 32785.38 | 54898.55 | 10672.22 |
| 10-14 years | 2022 | Male | 32938.97 | 75360.86 | NA |
| 10-14 years | 2023 | Male | 33209.25 | 94514.36 | NA |
| 10-14 years | 2024 | Male | 33595.61 | 108749 | NA |
| 10-14 years | 2025 | Male | 34220.92 | 119434.3 | NA |
| 10-14 years | 2026 | Male | 35282.17 | 128929.4 | NA |
| 10-14 years | 2027 | Male | 37185.83 | 139616.2 | NA |
| 10-14 years | 2028 | Male | 38038.69 | 144385.7 | NA |
| 10-14 years | 2029 | Male | 38560.59 | 145912.4 | NA |
| 10-14 years | 2030 | Male | 38548.55 | 143893.9 | NA |
| 10-14 years | 2020 | Female | 33775.21 | 42579.67 | 24970.75 |
| 10-14 years | 2021 | Female | 33736.75 | 49924.03 | 17549.47 |
| 10-14 years | 2022 | Female | 33881.32 | 64902.76 | 2859.879 |
| 10-14 years | 2023 | Female | 34194.64 | 79855.88 | NA |
| 10-14 years | 2024 | Female | 34687.36 | 91558.13 | NA |
| 10-14 years | 2025 | Female | 35460.07 | 100747.4 | NA |
| 10-14 years | 2026 | Female | 36677.04 | 109030.9 | NA |
| 10-14 years | 2027 | Female | 38748.79 | 118267.5 | NA |
| 10-14 years | 2028 | Female | 39650.38 | 122342 | NA |
| 10-14 years | 2029 | Female | 40164.44 | 123695.9 | NA |
| 10-14 years | 2030 | Female | 40101.53 | 122117.7 | NA |
| 10-14 years | 2020 | Both | 66559.67 | 86480.1 | 46639.24 |
| 10-14 years | 2021 | Both | 66522.13 | 104822.6 | 28221.68 |
| 10-14 years | 2022 | Both | 66820.29 | 140263.6 | 2859.879 |
| 10-14 years | 2023 | Both | 67403.89 | 174370.2 | NA |
| 10-14 years | 2024 | Both | 68282.97 | 200307.2 | NA |
| 10-14 years | 2025 | Both | 69680.99 | 220181.7 | NA |
| 10-14 years | 2026 | Both | 71959.21 | 237960.3 | NA |
| 10-14 years | 2027 | Both | 75934.62 | 257883.7 | NA |
| 10-14 years | 2028 | Both | 77689.07 | 266727.7 | NA |
| 10-14 years | 2029 | Both | 78725.04 | 269608.3 | NA |
| 10-14 years | 2030 | Both | 78650.08 | 266011.5 | NA |
| 15-19 years | 2020 | Male | 25170.68 | 37062.88 | 13278.48 |
| 15-19 years | 2021 | Male | 25217.86 | 49105.29 | 1330.43 |
| 15-19 years | 2022 | Male | 25237.72 | 70863.19 | NA |
| 15-19 years | 2023 | Male | 25198.88 | 90233.98 | NA |
| 15-19 years | 2024 | Male | 25135.89 | 103454 | NA |
| 15-19 years | 2025 | Male | 25075.77 | 111866 | NA |
| 15-19 years | 2026 | Male | 25079.45 | 117476.9 | NA |
| 15-19 years | 2027 | Male | 25204.18 | 121666.9 | NA |
| 15-19 years | 2028 | Male | 25421.18 | 124953.7 | NA |
| 15-19 years | 2029 | Male | 25728.83 | 127785 | NA |
| 15-19 years | 2030 | Male | 26220.38 | 130929.5 | NA |
| 15-19 years | 2020 | Female | 36244.1 | 45512.97 | 26975.22 |
| 15-19 years | 2021 | Female | 36015.58 | 53114.95 | 18916.22 |
| 15-19 years | 2022 | Female | 35803.23 | 68165.59 | 3440.864 |
| 15-19 years | 2023 | Female | 35569.92 | 82138.71 | NA |
| 15-19 years | 2024 | Female | 35357.13 | 91866.6 | NA |
| 15-19 years | 2025 | Female | 35195.65 | 98165.49 | NA |
| 15-19 years | 2026 | Female | 35160.06 | 102483 | NA |
| 15-19 years | 2027 | Female | 35320.2 | 105862.8 | NA |
| 15-19 years | 2028 | Female | 35659.52 | 108750 | NA |
| 15-19 years | 2029 | Female | 36188.09 | 111518.5 | NA |
| 15-19 years | 2030 | Female | 37010.44 | 114720.3 | NA |
| 15-19 years | 2020 | Both | 61414.78 | 82575.85 | 40253.7 |
| 15-19 years | 2021 | Both | 61233.44 | 102220.2 | 20246.65 |
| 15-19 years | 2022 | Both | 61040.94 | 139028.8 | 3440.864 |
| 15-19 years | 2023 | Both | 60768.8 | 172372.7 | NA |
| 15-19 years | 2024 | Both | 60493.01 | 195320.6 | NA |
| 15-19 years | 2025 | Both | 60271.42 | 210031.5 | NA |
| 15-19 years | 2026 | Both | 60239.51 | 219959.9 | NA |
| 15-19 years | 2027 | Both | 60524.39 | 227529.8 | NA |
| 15-19 years | 2028 | Both | 61080.71 | 233703.7 | NA |
| 15-19 years | 2029 | Both | 61916.92 | 239303.5 | NA |
| 15-19 years | 2030 | Both | 63230.82 | 245649.8 | NA |

## Supplementary Table 18. Predicted DALY rates from 2020 to 2030 based on BAPC model

| **Age group** | **Year** | **Sex** | **Value** | **Upper** | **Lower** |
| --- | --- | --- | --- | --- | --- |
| <5 years | 2020 | Male | 114.2331 | 117.1714 | 111.3753 |
| <5 years | 2021 | Male | 115.6322 | 119.6367 | 111.8067 |
| <5 years | 2022 | Male | 117.0539 | 122.4729 | 111.9525 |
| <5 years | 2023 | Male | 118.5004 | 125.6285 | 111.8723 |
| <5 years | 2024 | Male | 119.9739 | 129.0771 | 111.6065 |
| <5 years | 2025 | Male | 121.4765 | 132.8078 | 111.1821 |
| <5 years | 2026 | Male | 123.0105 | 136.8188 | 110.6182 |
| <5 years | 2027 | Male | 124.5784 | 141.1135 | 109.9292 |
| <5 years | 2028 | Male | 126.1826 | 145.6991 | 109.1267 |
| <5 years | 2029 | Male | 127.8258 | 150.5851 | 108.2204 |
| <5 years | 2030 | Male | 129.5107 | 155.7834 | 107.2188 |
| <5 years | 2020 | Female | 100.7144 | 103.3453 | 98.15411 |
| <5 years | 2021 | Female | 101.7905 | 105.3202 | 98.40959 |
| <5 years | 2022 | Female | 102.8827 | 107.6065 | 98.41881 |
| <5 years | 2023 | Female | 103.9927 | 110.1605 | 98.23215 |
| <5 years | 2024 | Female | 105.1222 | 112.9581 | 97.88524 |
| <5 years | 2025 | Female | 106.273 | 115.9881 | 97.40221 |
| <5 years | 2026 | Female | 107.4469 | 119.2472 | 96.80019 |
| <5 years | 2027 | Female | 108.6458 | 122.7367 | 96.09215 |
| <5 years | 2028 | Female | 109.8717 | 126.461 | 95.28849 |
| <5 years | 2029 | Female | 111.1265 | 130.4267 | 94.39795 |
| <5 years | 2030 | Female | 112.4124 | 134.6422 | 93.42812 |
| <5 years | 2020 | Both | 107.4738 | 110.2583 | 104.7647 |
| <5 years | 2021 | Both | 108.7113 | 112.4784 | 105.1081 |
| <5 years | 2022 | Both | 109.9683 | 115.0397 | 105.1857 |
| <5 years | 2023 | Both | 111.2466 | 117.8945 | 105.0522 |
| <5 years | 2024 | Both | 112.548 | 121.0176 | 104.7459 |
| <5 years | 2025 | Both | 113.8747 | 124.398 | 104.2922 |
| <5 years | 2026 | Both | 115.2287 | 128.033 | 103.7092 |
| <5 years | 2027 | Both | 116.6121 | 131.9251 | 103.0107 |
| <5 years | 2028 | Both | 118.0271 | 136.08 | 102.2076 |
| <5 years | 2029 | Both | 119.4761 | 140.5059 | 101.3092 |
| <5 years | 2030 | Both | 120.9616 | 145.2128 | 100.3235 |
| 5-9 years | 2020 | Male | 127.6091 | 130.1301 | 125.1246 |
| 5-9 years | 2021 | Male | 128.2408 | 131.425 | 125.1374 |
| 5-9 years | 2022 | Male | 129.2698 | 133.4665 | 125.2337 |
| 5-9 years | 2023 | Male | 130.6783 | 136.2111 | 125.4158 |
| 5-9 years | 2024 | Male | 132.2858 | 139.4691 | 125.5149 |
| 5-9 years | 2025 | Male | 133.9234 | 143.0672 | 125.3834 |
| 5-9 years | 2026 | Male | 135.593 | 146.9916 | 125.0555 |
| 5-9 years | 2027 | Male | 137.2965 | 151.2363 | 124.5559 |
| 5-9 years | 2028 | Male | 139.0362 | 155.8017 | 123.9034 |
| 5-9 years | 2029 | Male | 140.8147 | 160.6925 | 123.1129 |
| 5-9 years | 2030 | Male | 142.6348 | 165.9169 | 122.1965 |
| 5-9 years | 2020 | Female | 128.6479 | 131.2145 | 126.1176 |
| 5-9 years | 2021 | Female | 129.2048 | 132.3946 | 126.0887 |
| 5-9 years | 2022 | Female | 130.1165 | 134.276 | 126.0995 |
| 5-9 years | 2023 | Female | 131.3574 | 136.8093 | 126.146 |
| 5-9 years | 2024 | Female | 132.7678 | 139.8203 | 126.0867 |
| 5-9 years | 2025 | Female | 134.2029 | 143.1553 | 125.8007 |
| 5-9 years | 2026 | Female | 135.6645 | 146.7982 | 125.3223 |
| 5-9 years | 2027 | Female | 137.1545 | 150.741 | 124.6767 |
| 5-9 years | 2028 | Female | 138.6749 | 154.982 | 123.883 |
| 5-9 years | 2029 | Female | 140.2281 | 159.5238 | 122.9561 |
| 5-9 years | 2030 | Female | 141.8165 | 164.3725 | 121.9087 |
| 5-9 years | 2020 | Both | 128.1285 | 130.6723 | 125.6211 |
| 5-9 years | 2021 | Both | 128.7228 | 131.9098 | 125.613 |
| 5-9 years | 2022 | Both | 129.6931 | 133.8712 | 125.6666 |
| 5-9 years | 2023 | Both | 131.0179 | 136.5102 | 125.7809 |
| 5-9 years | 2024 | Both | 132.5268 | 139.6447 | 125.8008 |
| 5-9 years | 2025 | Both | 134.0632 | 143.1113 | 125.592 |
| 5-9 years | 2026 | Both | 135.6288 | 146.8949 | 125.1889 |
| 5-9 years | 2027 | Both | 137.2255 | 150.9887 | 124.6163 |
| 5-9 years | 2028 | Both | 138.8556 | 155.3918 | 123.8932 |
| 5-9 years | 2029 | Both | 140.5214 | 160.1081 | 123.0345 |
| 5-9 years | 2030 | Both | 142.2257 | 165.1447 | 122.0526 |
| 10-14 years | 2020 | Male | 83.83566 | 85.45819 | 82.23654 |
| 10-14 years | 2021 | Male | 84.07208 | 86.1254 | 82.07247 |
| 10-14 years | 2022 | Male | 84.32205 | 87.03508 | 81.71712 |
| 10-14 years | 2023 | Male | 84.6035 | 88.16086 | 81.22977 |
| 10-14 years | 2024 | Male | 84.89535 | 89.44889 | 80.62153 |
| 10-14 years | 2025 | Male | 85.1812 | 90.86507 | 79.89761 |
| 10-14 years | 2026 | Male | 85.61931 | 92.56069 | 79.22828 |
| 10-14 years | 2027 | Male | 86.32872 | 94.66318 | 78.72984 |
| 10-14 years | 2028 | Male | 87.2974 | 97.17578 | 78.38141 |
| 10-14 years | 2029 | Male | 88.4034 | 99.98774 | 78.05833 |
| 10-14 years | 2030 | Male | 89.53375 | 102.9932 | 77.65117 |
| 10-14 years | 2020 | Female | 102.8755 | 104.8823 | 100.8972 |
| 10-14 years | 2021 | Female | 103.0492 | 105.5473 | 100.6099 |
| 10-14 years | 2022 | Female | 103.2414 | 106.5028 | 100.0944 |
| 10-14 years | 2023 | Female | 103.4817 | 107.7288 | 99.42855 |
| 10-14 years | 2024 | Female | 103.754 | 109.1699 | 98.63599 |
| 10-14 years | 2025 | Female | 104.0447 | 110.7899 | 97.73007 |
| 10-14 years | 2026 | Female | 104.5147 | 112.7401 | 96.88676 |
| 10-14 years | 2027 | Female | 105.2782 | 115.143 | 96.21889 |
| 10-14 years | 2028 | Female | 106.3145 | 117.9932 | 95.69796 |
| 10-14 years | 2029 | Female | 107.493 | 121.1712 | 95.19153 |
| 10-14 years | 2030 | Female | 108.6962 | 124.5674 | 94.58632 |
| 10-14 years | 2020 | Both | 93.35558 | 95.17023 | 91.56686 |
| 10-14 years | 2021 | Both | 93.56063 | 95.83637 | 91.34119 |
| 10-14 years | 2022 | Both | 93.78171 | 96.76893 | 90.90577 |
| 10-14 years | 2023 | Both | 94.04259 | 97.94483 | 90.32916 |
| 10-14 years | 2024 | Both | 94.32467 | 99.30937 | 89.62876 |
| 10-14 years | 2025 | Both | 94.61297 | 100.8275 | 88.81384 |
| 10-14 years | 2026 | Both | 95.06701 | 102.6504 | 88.05752 |
| 10-14 years | 2027 | Both | 95.80346 | 104.9031 | 87.47436 |
| 10-14 years | 2028 | Both | 96.80597 | 107.5845 | 87.03968 |
| 10-14 years | 2029 | Both | 97.9482 | 110.5795 | 86.62493 |
| 10-14 years | 2030 | Both | 99.115 | 113.7803 | 86.11875 |
| 15-19 years | 2020 | Male | 63.12715 | 64.34062 | 61.93156 |
| 15-19 years | 2021 | Male | 63.40157 | 64.94137 | 61.90306 |
| 15-19 years | 2022 | Male | 63.67877 | 65.72009 | 61.72182 |
| 15-19 years | 2023 | Male | 63.95612 | 66.63959 | 61.41692 |
| 15-19 years | 2024 | Male | 64.21011 | 67.64972 | 60.98979 |
| 15-19 years | 2025 | Male | 64.41235 | 68.70613 | 60.43054 |
| 15-19 years | 2026 | Male | 64.6061 | 69.84162 | 59.79638 |
| 15-19 years | 2027 | Male | 64.81565 | 71.07595 | 59.11975 |
| 15-19 years | 2028 | Male | 65.05405 | 72.42151 | 58.41838 |
| 15-19 years | 2029 | Male | 65.3036 | 73.86025 | 57.67964 |
| 15-19 years | 2030 | Male | 65.55111 | 75.37977 | 56.8944 |
| 15-19 years | 2020 | Female | 106.0233 | 108.0702 | 104.0058 |
| 15-19 years | 2021 | Female | 106.3647 | 108.9221 | 103.8682 |
| 15-19 years | 2022 | Female | 106.703 | 110.054 | 103.4704 |
| 15-19 years | 2023 | Female | 107.041 | 111.4155 | 102.8666 |
| 15-19 years | 2024 | Female | 107.3411 | 112.926 | 102.0631 |
| 15-19 years | 2025 | Female | 107.5593 | 114.5136 | 101.0478 |
| 15-19 years | 2026 | Female | 107.7615 | 116.2262 | 99.91026 |
| 15-19 years | 2027 | Female | 107.9901 | 118.0982 | 98.70634 |
| 15-19 years | 2028 | Female | 108.2756 | 120.1588 | 97.47362 |
| 15-19 years | 2029 | Female | 108.5995 | 122.3895 | 96.20063 |
| 15-19 years | 2030 | Female | 108.9468 | 124.777 | 94.87795 |
| 15-19 years | 2020 | Both | 84.57521 | 86.20543 | 82.96867 |
| 15-19 years | 2021 | Both | 84.88312 | 86.93172 | 82.88564 |
| 15-19 years | 2022 | Both | 85.19088 | 87.88702 | 82.59609 |
| 15-19 years | 2023 | Both | 85.49855 | 89.02757 | 82.14175 |
| 15-19 years | 2024 | Both | 85.77563 | 90.28784 | 81.52643 |
| 15-19 years | 2025 | Both | 85.98582 | 91.60985 | 80.73917 |
| 15-19 years | 2026 | Both | 86.18379 | 93.03392 | 79.85332 |
| 15-19 years | 2027 | Both | 86.40287 | 94.58707 | 78.91305 |
| 15-19 years | 2028 | Both | 86.66481 | 96.29018 | 77.946 |
| 15-19 years | 2029 | Both | 86.95152 | 98.12486 | 76.94013 |
| 15-19 years | 2030 | Both | 87.24897 | 100.0784 | 75.88617 |


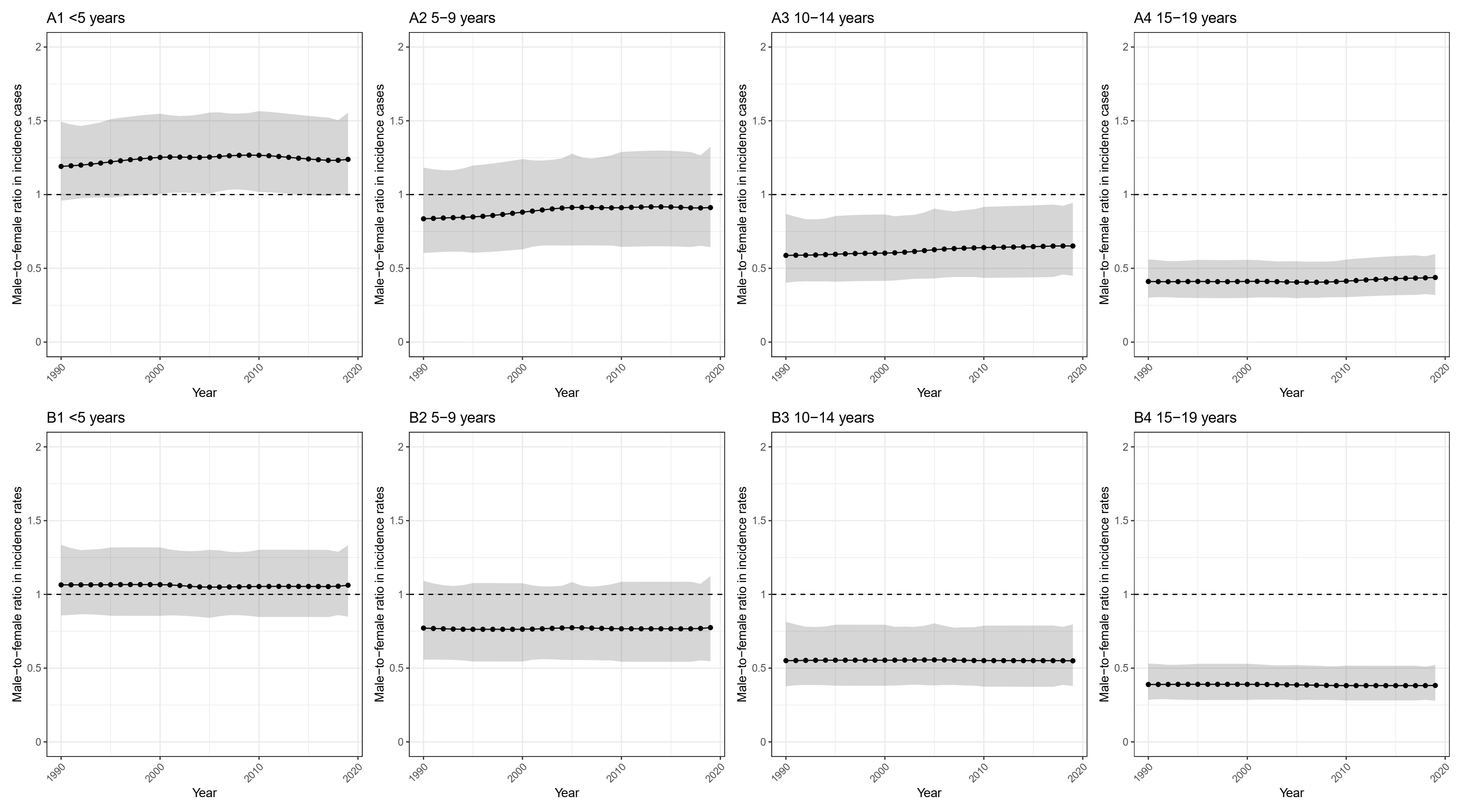


## Supplementary Figure 1. Temporal trend of male-to-female ratio in incidence of Chinese children and adolescents with AD in different age groups from 1990 to 2019

(A1) Temporal trend of male-to-female ratio in incidence cases of Chinese AD patients aged < 5 years from 1990 to 2019; (A2) Temporal trend of male-to-female ratio in incidence cases of Chinese AD patients aged 5-9 years from 1990 to 2019; (A3) Temporal trend of male-to-female ratio in incidence cases of Chinese AD patients aged 10-14 years from 1990 to 2019; (A4) Temporal trend of male-to-female ratio in incidence cases of Chinese AD patients aged 15-19 years from 1990 to 2019; (B1) Temporal trend of incidence rates of Chinese AD patients aged < 5 years from 1990 to 2019; (B2) Temporal trend of incidence rates of Chinese AD patients aged 5-9 years from 1990 to 2019; (B3) Temporal trend of incidence rates of Chinese AD patients aged 10-14 years from 1990 to 2019; (B4) Temporal trend of incidence rates of Chinese AD patients aged 15-19 years from 1990 to 2019.

AD, atopic dermatitis.


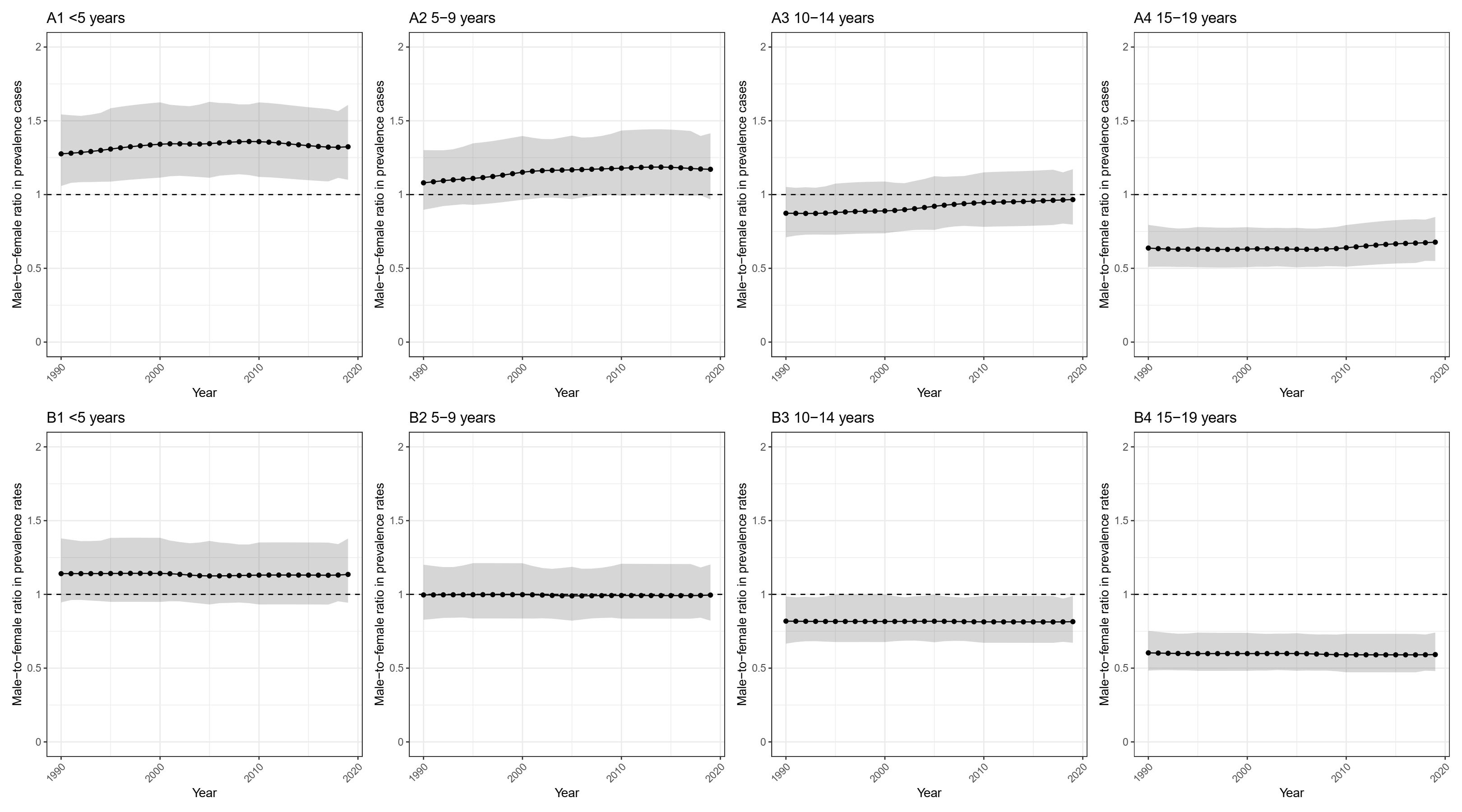


## Supplementary Figure 2. Temporal trend of male-to-female ratio in prevalence of Chinese children and adolescents with AD in different age groups from 1990 to 2019

(A1) Temporal trend of male-to-female ratio in prevalence cases of Chinese AD patients aged < 5 years from 1990 to 2019; (A2) Temporal trend of male-to-female ratio in prevalence cases of Chinese AD patients aged 5-9 years from 1990 to 2019; (A3) Temporal trend of male-to-female ratio in prevalence cases of Chinese AD patients aged 10-14 years from 1990 to 2019; (A4) Temporal trend of male-to-female ratio in prevalence cases of Chinese AD patients aged 15-19 years from 1990 to 2019; (B1) Temporal trend of prevalence rates of Chinese AD patients aged < 5 years from 1990 to 2019; (B2) Temporal trend of prevalence rates of Chinese AD patients aged 5-9 years from 1990 to 2019; (B3) Temporal trend of prevalence rates of Chinese AD patients aged 10-14 years from 1990 to 2019; (B4) Temporal trend of prevalence rates of Chinese AD patients aged 15-19 years from 1990 to 2019.

AD, atopic dermatitis.


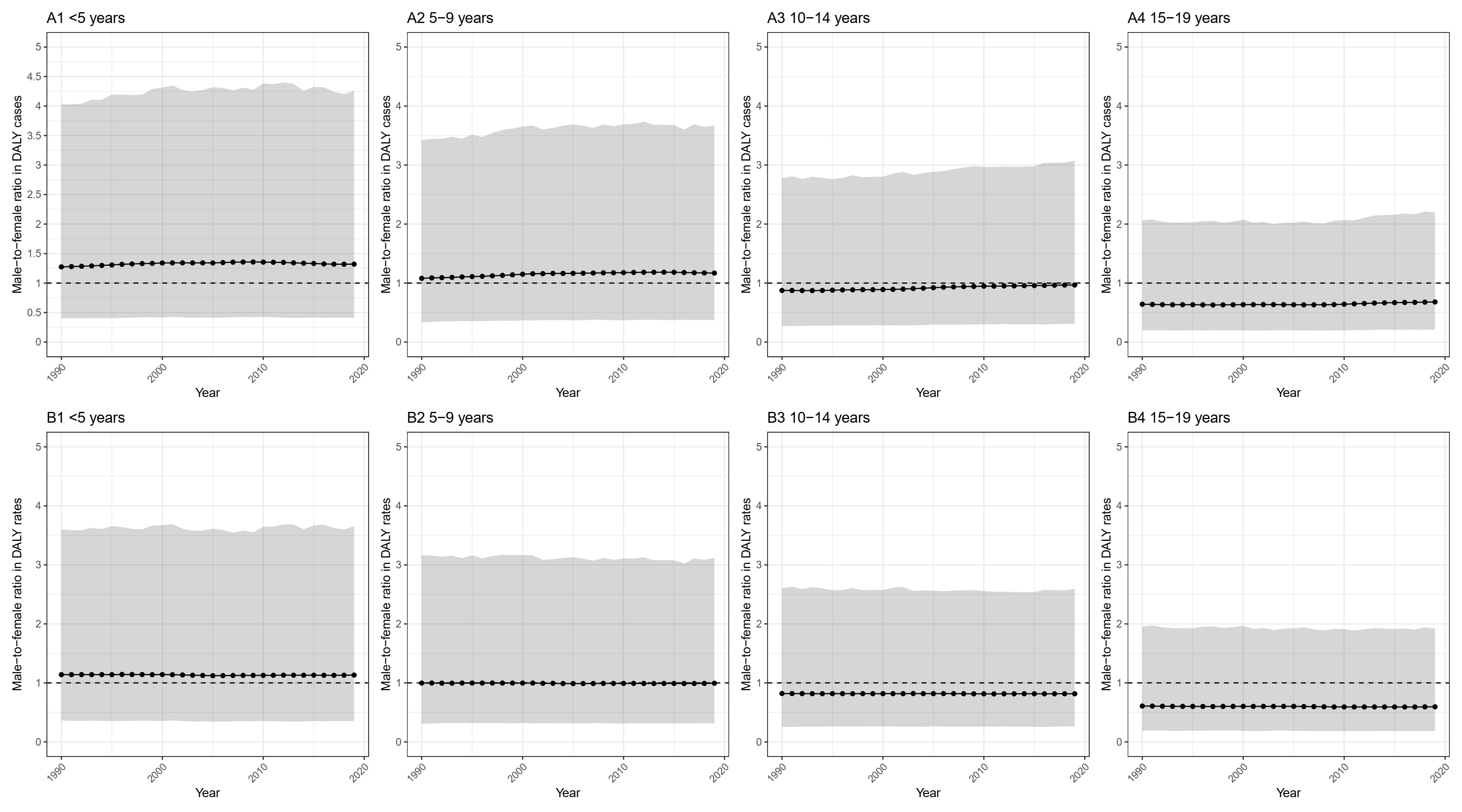


## Supplementary Figure 3. Temporal trend of male-to-female ratio in DALY of Chinese children and adolescents with AD in different age groups from 1990 to 2019

(A1) Temporal trend of male-to-female ratio in DALY cases of Chinese AD patients aged < 5 years from 1990 to 2019; (A2) Temporal trend of male-to-female ratio in DALY cases of Chinese AD patients aged 5-9 years from 1990 to 2019; (A3) Temporal trend of male-to-female ratio in DALY cases of Chinese AD patients aged 10-14 years from 1990 to 2019; (A4) Temporal trend of male-to-female ratio in DALY cases of Chinese AD patients aged 15-19 years from 1990 to 2019; (B1) Temporal trend of DALY rates of Chinese AD patients aged < 5 years from 1990 to 2019; (B2) Temporal trend of DALY rates of Chinese AD patients aged 5-9 years from 1990 to 2019; (B3) Temporal trend of DALY rates of Chinese AD patients aged 10-14 years from 1990 to 2019; (B4) Temporal trend of DALY rates of Chinese AD patients aged 15-19 years from 1990 to 2019.

AD, atopic dermatitis; DALY, disability-adjusted life year.


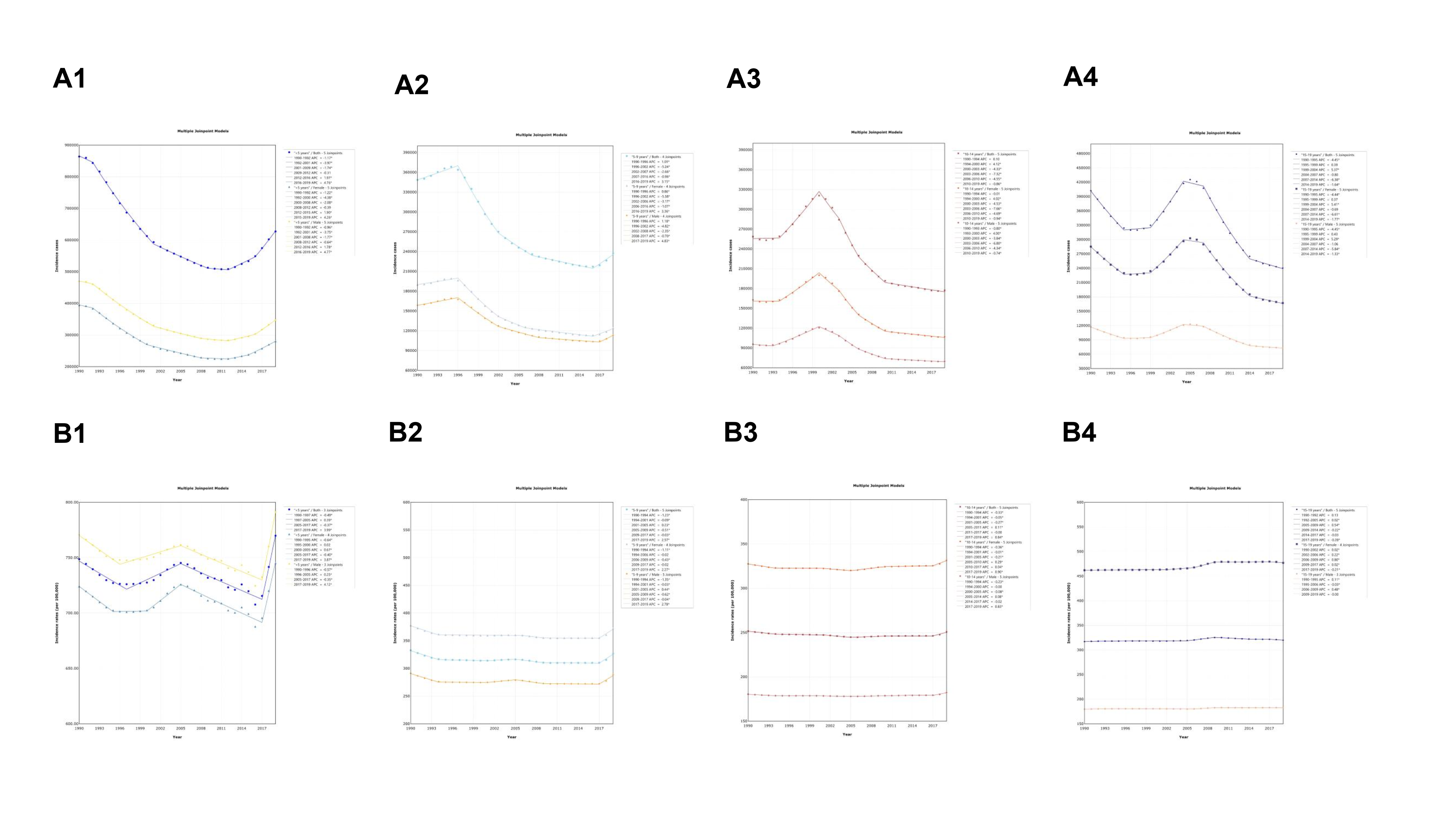


## Supplementary Figure 4. Joinpoint models for incidence of Chinese children and adolescents with AD in different age groups from 1990 to 2019

(A1) Joinpoint models for incidence cases of Chinese AD patients aged < 5 years from 1990 to 2019; (A2) Joinpoint models for incidence cases of Chinese AD patients aged 5-9 years from 1990 to 2019; (A3) Joinpoint models for incidence cases of Chinese AD patients aged 10-14 years from 1990 to 2019; (A4) Joinpoint models for incidence cases of Chinese AD patients aged 15-19 years from 1990 to 2019; (B1) Joinpoint models for incidence rates of Chinese AD patients aged < 5 years from 1990 to 2019; (B2) Joinpoint models for incidence rates of Chinese AD patients aged 5-9 years from 1990 to 2019; (B3) Joinpoint models for incidence rates of Chinese AD patients aged 10-14 years from 1990 to 2019; (B4) Joinpoint models for incidence rates of Chinese AD patients aged 15-19 years from 1990 to 2019.

AD, atopic dermatitis.


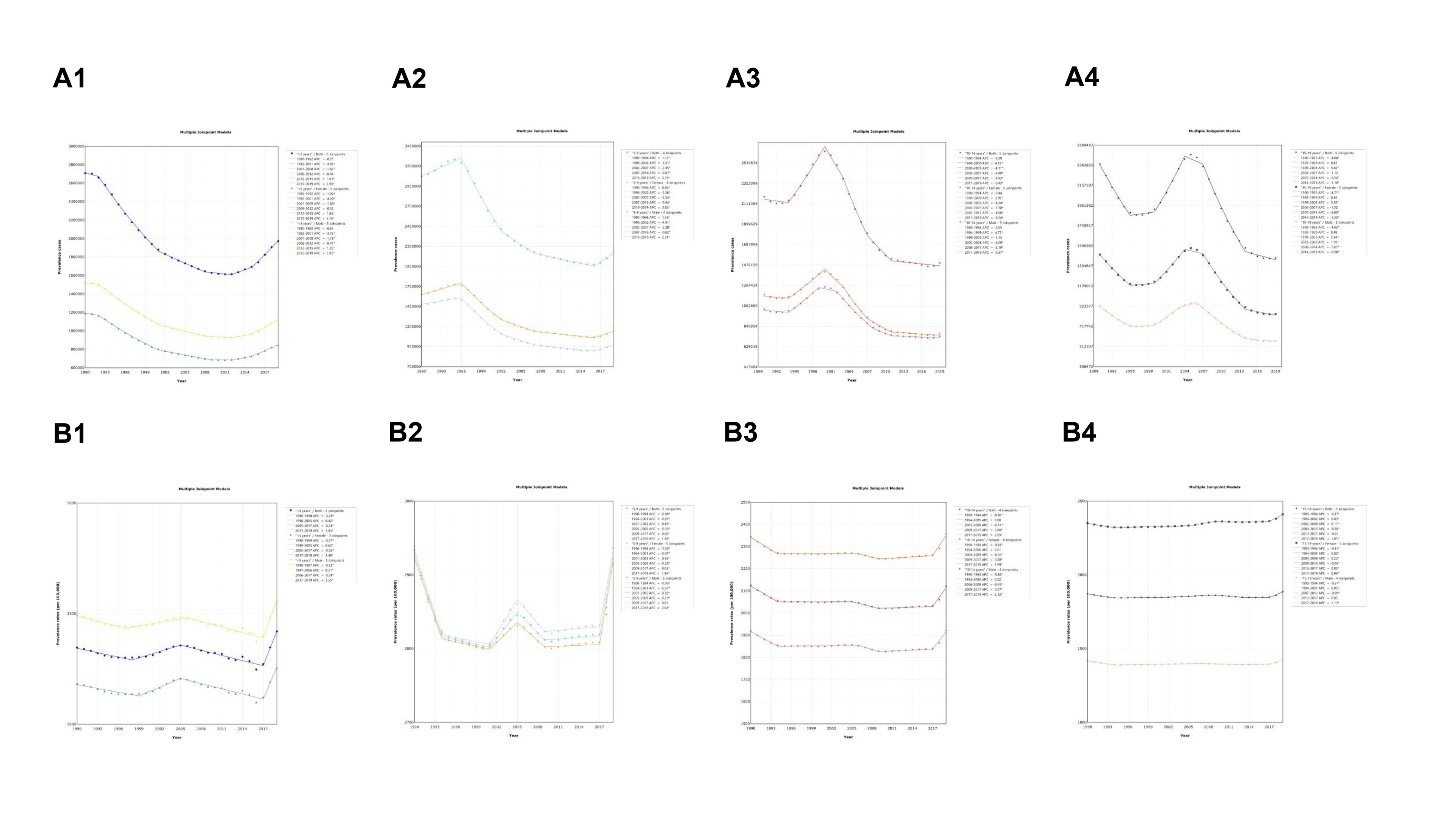


## Supplementary Figure 5. Joinpoint models for prevalence of Chinese children and adolescents with AD in different age groups from 1990 to 2019

(A1) Joinpoint models for prevalence cases of Chinese AD patients aged < 5 years from 1990 to 2019; (A2) Joinpoint models for prevalence cases of Chinese AD patients aged 5-9 years from 1990 to 2019; (A3) Joinpoint models for prevalence cases of Chinese AD patients aged 10-14 years from 1990 to 2019; (A4) Joinpoint models for prevalence cases of Chinese AD patients aged 15-19 years from 1990 to 2019; (B1) Joinpoint models for prevalence rates of Chinese AD patients aged < 5 years from 1990 to 2019; (B2) Joinpoint models for prevalence rates of Chinese AD patients aged 5-9 years from 1990 to 2019; (B3) Joinpoint models for prevalence rates of Chinese AD patients aged 10-14 years from 1990 to 2019; (B4) Joinpoint models for prevalence rates of Chinese AD patients aged 15-19 years from 1990 to 2019.

AD, atopic dermatitis.


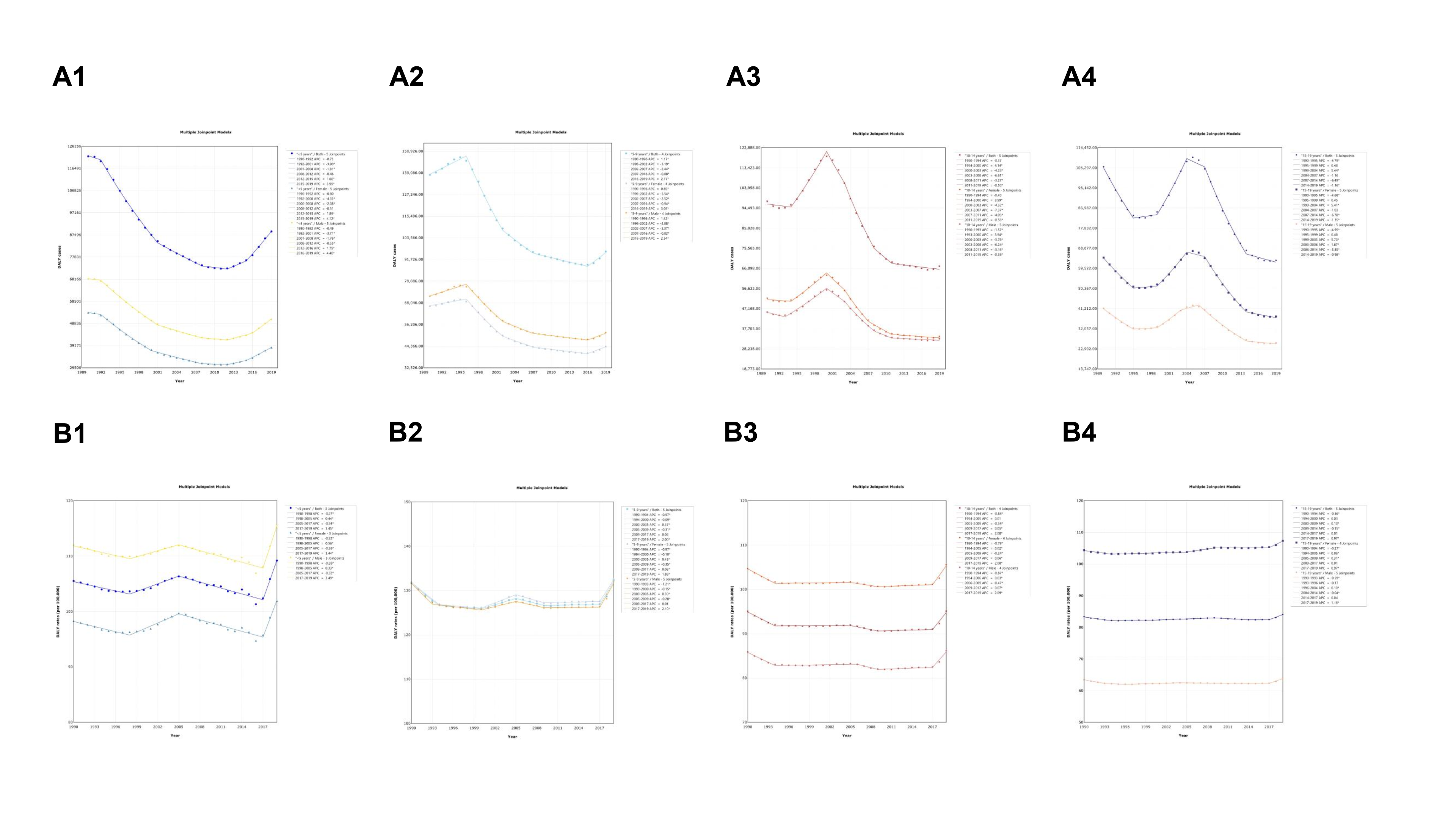


## Supplementary Figure 6. Joinpoint models for DALY of Chinese children and adolescents with AD in different age groups from 1990 to 2019

(A1) Joinpoint models for DALY cases of Chinese AD patients aged < 5 years from 1990 to 2019; (A2) Joinpoint models for DALY cases of Chinese AD patients aged 5-9 years from 1990 to 2019; (A3) Joinpoint models for DALY cases of Chinese AD patients aged 10-14 years from 1990 to 2019; (A4) Joinpoint models for DALY cases of Chinese AD patients aged 15-19 years from 1990 to 2019; (B1) Joinpoint models for DALY rates of Chinese AD patients aged < 5 years from 1990 to 2019; (B2) Joinpoint models for DALY rates of Chinese AD patients aged 5-9 years from 1990 to 2019; (B3) Joinpoint models for DALY rates of Chinese AD patients aged 10-14 years from 1990 to 2019; (B4) Joinpoint models for DALY rates of Chinese AD patients aged 15-19 years from 1990 to 2019.

AD, atopic dermatitis; DALY, disability-adjusted life year.
